# Dynamic isolation forest for anomaly detection in post-PCI myocardial infarction patients

**DOI:** 10.1038/s41598-026-54390-7

**Published:** 2026-05-22

**Authors:** Yu Zhang, Shan Gao, He Xu, Yuan Liu, Yaqiong Guo, XueLing Wei

**Affiliations:** 1 Medical Laboratory, The Second People’s Hospital of Gansu Province (Affiliated Hospital of Northwest Minzu University), Lanzhou, 743000 China; 2 Medical Laboratory, Xinjiang Baoshihua Hospital, Urumqi, 830000 China

**Keywords:** Dynamic isolation forest, Anomaly detection, Post-PCI, Myocardial infarction, Biochemistry, Computational biology and bioinformatics, Cardiology

## Abstract

Patients with myocardial infarction require continuous monitoring of multiple laboratory parameters after percutaneous coronary intervention (PCI), but assessment of their dynamic changes and of whether they deviate from the typical recovery trajectory still relies largely on clinical experience, and objective methods remain lacking. This study included 183 patients with myocardial infarction who underwent PCI, constructed fixed three-time-point windows, used blinded expert review as the reference, compared dynamic isolation forest (DIF) with other unsupervised methods, and conducted an external supportive prognostic analysis in MIMIC-IV. The results showed that DIF had the best agreement with expert ratings (Spearman’s ρ = 0.585; Kendall’s τ = 0.452), with an AUC of 0.859, and overall outperformed the other methods; the MIMIC-IV analysis showed that higher DIF anomaly scores were associated with an increased risk of death. DIF can be used to identify abnormal recovery windows after PCI that deviate from the typical recovery trajectory and may have potential for risk stratification, although its clinical utility still requires further validation.

**Supplementary Information:**

The online version contains supplementary material available at 10.1038/s41598-026-54390-7.

## Introduction

 Myocardial infarction (MI) is an acute coronary syndrome caused by myocardial ischemia and represents one of the most severe clinical manifestations of coronary artery disease (CAD)^1^. According to data from the US National Inpatient Sample from 2004 to 2018, mortality among patients with acute myocardial infarction gradually declined; however, it still remained as high as 37% in 2018^2^. Percutaneous coronary intervention (PCI) is the standard reperfusion strategy for patients with MI, but during the early postprocedural period, patients may experience worsening heart failure, reperfusion injury, persistent inflammatory responses, and other adverse changes, contributing to a relatively high mortality rate^3–5^. These observations highlight the importance of perioperative monitoring. Dynamic changes in perioperative laboratory parameters may reflect patients’ postoperative physiological status and potential risk. However, assessment of dynamic changes in laboratory data after PCI still relies largely on clinical experience, and objective dynamic identification methods remain lacking.

In recent years, with the rapid development of machine learning, unsupervised learning methods have been increasingly applied to medical data and have shown considerable potential for anomaly detection^6^. Isolation forest (IF) is a classic unsupervised anomaly detection method with high computational efficiency, makes no assumptions about the underlying data distribution, and can be applied to different types of datasets. It has therefore been widely used in cybersecurity, finance, industrial monitoring, and environmental surveillance, and in medicine primarily for the detection of rare diseases in biomedical data^7–9^. In addition, one study evaluated five unsupervised machine-learning algorithms for anomaly detection and found that IF outperformed the other algorithms in balancing precision and recall^10^. Traditional IF is mainly designed for relatively independent sample points and is therefore not well suited to directly capture short-term dynamic changes in temporal data. In patients after PCI, greater attention should be paid to the joint patterns of serial laboratory measurements, the direction of change, and the magnitude of fluctuation.

To capture such short-term dynamic changes, this study used windows composed of three consecutive observations as the basic unit of analysis and, on this basis, developed a Dynamic Isolation Forest (DIF) framework. Compared with approaches that focus on a single test value, DIF places greater emphasis on the overall pattern of change across serial observations. For example, a given indicator may remain within an acceptable range at a single measurement, but a persistent increase or greater fluctuation across consecutive observations may suggest that the window deviates from the typical postoperative recovery trajectory. We reiterate that the term “anomaly” in this study does not refer to a single laboratory indicator exceeding its reference range, nor does it directly correspond to a subsequent clinical endpoint. Rather, it denotes a marked deviation in the joint pattern of laboratory changes within a fixed three-time-point window from the typical postoperative recovery trajectory observed in the local cohort, with abnormal recovery windows as the primary analytical focus.

This study used inpatient laboratory data from patients with MI who underwent PCI to evaluate the ability of DIF to identify abnormal recovery windows that deviate from the typical postoperative recovery trajectory. We used fixed three-time-point windows as the unit of analysis and blinded expert review as the primary clinical reference, while comparing DIF with standard isolation forest, local outlier factor (LOF), one-class support vector machine (OCSVM), and a z-score/change-based approach. In addition, we conducted an external supportive prognostic analysis in the MIMIC-IV database to explore the association between anomaly scores and subsequent adverse outcomes.

## Methods

### Database introduction

This study was a retrospective analysis based on existing inpatient medical records. The original data were derived from inpatient laboratory testing records of patients admitted for myocardial infarction (MI) who underwent percutaneous coronary intervention (PCI) between 2021 and 2026. The dataset included multidimensional laboratory data, including inflammatory markers, myocardial injury biomarkers, blood cell parameters, liver and kidney function indicators, electrolytes, and coagulation-related measures. The original database corresponded to 305 unique hospital admission IDs. All data were de-identified before analysis, and only the clinical variables and laboratory testing time points required for the study were retained.

According to China’s *Measures for the Ethical Review of Life Science and Medical Research Involving Human Beings* and the relevant institutional requirements of our hospital, retrospective studies using de-identified data generated during prior routine clinical care may be exempt from ethics review and the requirement for informed consent, provided that the subjects cannot be identified, no additional intervention is involved, and no personal privacy or commercial interests are implicated.

The external validation data used in this study were obtained from the Medical Information Mart for Intensive Care IV (MIMIC-IV), a publicly available database derived from the electronic health records of Beth Israel Deaconess Medical Center^11^.The corresponding author, Yu Zhang, completed the National Institutes of Health online training course and passed the Protecting Human Research Participants examination (certification no. 66963714). Access to this database was approved by the institutional review board of the Massachusetts Institute of Technology. Because this study used anonymized data provided by the database, the requirement for informed consent was waived. The study was conducted in accordance with the ethical standards of the Declaration of Helsinki.

### Study population

The original database included 305 patients hospitalized for MI who underwent PCI. The raw laboratory records were first organized by admission ID and testing date, and a chronologically ordered monitoring-day sequence was generated for each patient. The primary unit of analysis in this study was the patient-window, with each window consisting of three consecutive observations. It should be noted that, if a patient had only three total observations, although a fixed three-time-point window could still be formed, that window would represent the endpoint of all inpatient monitoring information for that patient and would lack the contextual basis indicating that monitoring was still ongoing. It would therefore be difficult to determine whether such a window belonged to a continuing postoperative recovery process. On this basis, to improve the interpretability of the fixed three-time-point windows and to reduce potential selection bias arising from early discharge, early outcomes, or insufficient monitoring, the main analysis included only patients with a monitoring-day sequence of at least 4. Ultimately, 183 patients were included in the main analysis.

### Baseline characteristics

The baseline characteristics of the development cohort included demographic information, MI subtype, vital signs on admission, Killip class, major comorbidities, readmission status, and infarct location. Continuous variables are presented as the median and interquartile range, and categorical variables as counts and percentages. Because the baseline table for the development cohort was intended for overall description rather than between-group comparison, no between-group hypothesis testing was performed. Some variables had missing data, and the corresponding extent of missingness is reported in Table 1.

### Data preprocessing and feature engineering

For repeated records from the same patient on the same testing date, data were merged at the “admission ID–testing date” level: numeric variables were averaged, and the first non-missing value was retained for text variables. The records were then sorted by admission ID and testing date to generate a consecutive monitoring-day sequence for each patient, and sex was coded numerically.

In the original dataset, the six coagulation tests had a high degree of missingness in the retrospective inpatient data. Given the clinical importance of coagulation-related indicators after PCI, directly including all of them in the model could increase imputation instability and amplify the impact of variables with substantial missingness on model construction. Therefore, the original six coagulation indicators were not directly included in the final model input. Instead, two coagulation summary variables were constructed: ‘coag_panel_done’ and ‘coag_burden_score’. ‘coag_panel_done’ was used to indicate whether any coagulation test had been performed at a given observation; it was coded as 0 when all six coagulation indicators were missing and as 1 when at least one indicator was available. ‘coag_burden_score’ was used to reflect the overall burden of coagulation abnormality and was calculated as the mean of six indicators—prothrombin activity, thrombin time, activated partial thromboplastin time, D-dimer, prothrombin time, and fibrinogen—after standardization according to the direction of abnormality. If no coagulation test was performed at a given observation, ‘coag_burden_score’ was set to 0. In the final model, the original six coagulation indicators were removed, and only these two coagulation summary variables were retained.

For variable retention, the missingness rate of N-terminal pro-B-type natriuretic peptide (NT-proBNP) (32.29%) was used as the threshold for the main analysis, and only candidate numeric variables with missingness not exceeding this threshold were retained; ‘coag_panel_done’ and ‘coag_burden_score’ were retained regardless of missingness. For right-skewed variables, a ‘log1p’ transformation was first applied, followed by robust standardization based on the median and interquartile range. Age, sex coding, and monitoring-day sequence were retained as metadata columns in their original scales and were not included in the log transformation, standardization, or k-nearest neighbors imputation procedures. The main analysis used k-nearest neighbors imputation with k = 5. To assess the robustness of the results to the choice of imputation parameter, additional imputed versions with k = 3 and k = 7 were constructed and used for sensitivity analyses under the same fixed-window framework and expert rating reference.

### Fixed-window construction

This study used the patient–window as the unit of analysis. A window was defined as three consecutive observations arranged in chronological order. Although, in theory, multiple overlapping three-time-point candidate windows could be generated within a single patient, the main formal analysis prespecified a fixed-window rule to ensure fair comparison across models and to avoid selection bias introduced by model-driven window selection. Specifically, only the first complete set of three consecutive observations, namely T1–T2–T3, was included for each patient. All models were scored on the same set of fixed windows, and the experts performed a single blinded review of that same set of windows.

### Model construction and comparison

The aim of this study was not to develop a conventional supervised learning prediction model, but rather to evaluate a dynamic anomaly identification workflow based on windows formed by three consecutive observations, designed to identify abnormal recovery windows that markedly deviate from the typical postoperative recovery pattern observed in the local cohort. The window-level anomaly score does not indicate whether a given window is abnormal relative to the patient’s own prior history; rather, it indicates whether the joint pattern of change in that window deviates markedly from the typical postoperative recovery trajectory across all fixed windows in the cohort.

Within each fixed window, three types of features were extracted for each laboratory variable: the mean within the window, the difference between the last and first observations, and the variance within the window, to characterize the overall level, direction of change, and magnitude of fluctuation of that window. Based on a unified window-level feature vector, five anomaly-scoring methods were implemented: Dynamic Isolation Forest (DIF), Standard Isolation Forest (Standard IF), Local Outlier Factor (LOF), One-Class Support Vector Machine (OCSVM), and a z-score/change-based baseline method. All models generated continuous anomaly scores, with higher scores indicating greater deviation from the typical postoperative recovery trajectory observed in the local cohort. In the subsequent external analysis, we further examined the association between the continuous DIF anomaly score and mortality outcomes.

**Fig. 1:**
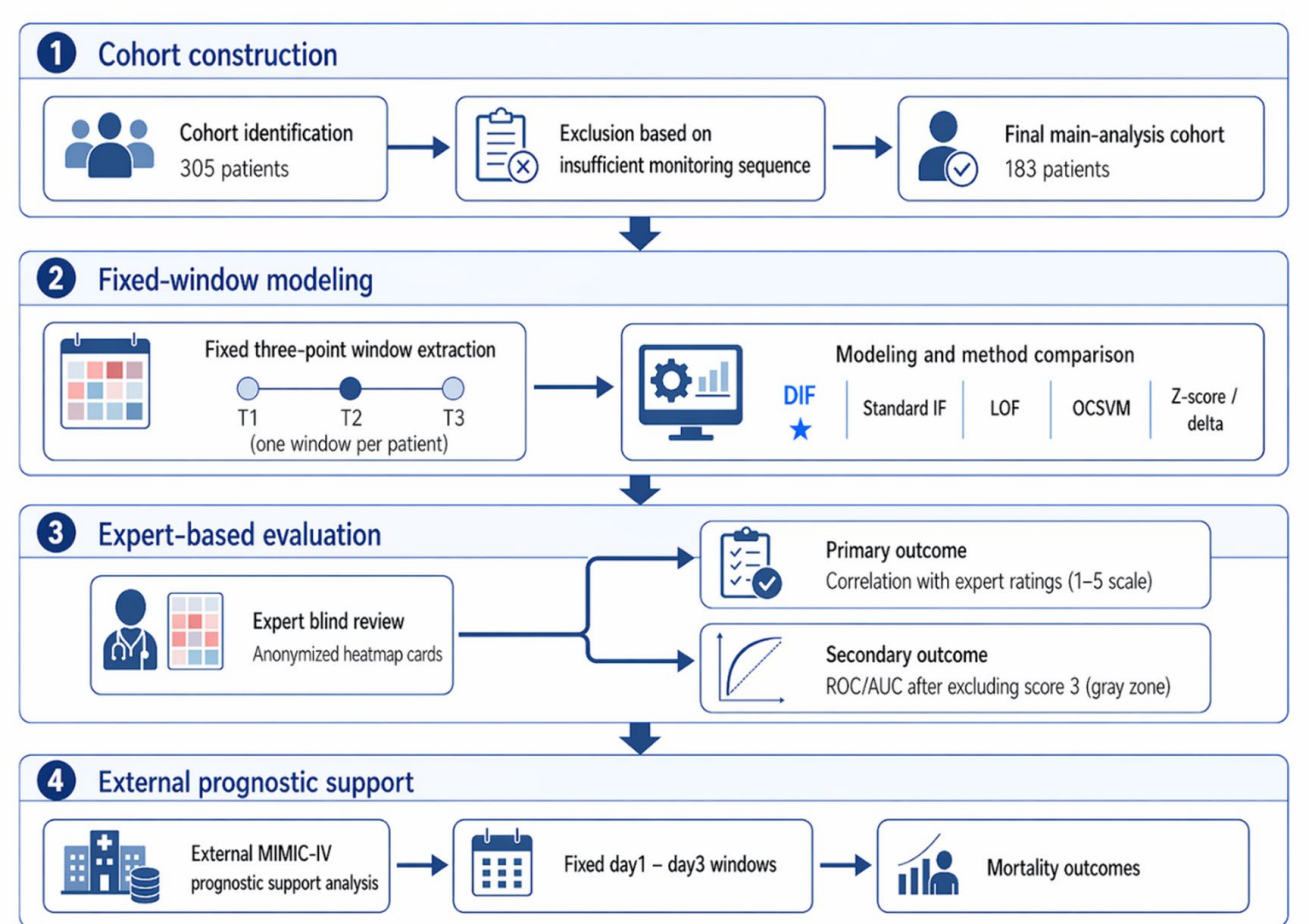
Schematic overview of the overall study workflow. This figure illustrates the overall workflow of the study, from development cohort selection, fixed three-time-point window construction, and window-level feature extraction to model comparison, blinded expert review, and the external supportive survival association analysis in MIMIC-IV.

To facilitate understanding of the overall analytical workflow of this study, Fig. 1 provides an overview of the complete DIF workflow, from development cohort selection, fixed three-time-point window construction, and window-level feature representation to model comparison, blinded expert evaluation, and external supportive prognostic analysis in MIMIC-IV.

### Blinded expert review and reference standard

The objects of expert evaluation were fixed-window heatmap cards. Each card displayed a full-panel heatmap arranged in a fixed order together with a separate response area, and the experts reviewed the cards under blinded conditions without access to the model scores. The blinded expert panel consisted of two physicians with more than 10 years of professional experience, from the fields of laboratory medicine and cardiology, respectively. When disagreement arose between the two experts, adjudication was performed by a third physician with relevant clinical experience.

The rating system used a 5-point ordered scale: a score of 1 indicated very typical recovery, 2 indicated relatively typical recovery, 3 indicated a gray zone or uncertainty, 4 indicated clearly abnormal recovery, and 5 indicated very clearly abnormal recovery. For windows rated 4–5, the experts additionally recorded the three variables showing the most abnormal changes.

In this study, “anomaly” was primarily defined as a marked deviation in the joint pattern of laboratory changes within a fixed three-time-point window from the typical postoperative recovery trajectory observed in the local cohort, with the primary clinical reference standard being an “abnormal recovery window” determined by blinded expert review. Subsequent clinical events and external database analyses were used only as secondary supportive evidence. In the main formal analysis, the primary evaluation framework was the agreement between the model’s continuous scores and the experts’ 1–5ratings. In the secondary analysis, expert ratings were dichotomized as “scores1–2 = non-anomalous” and “scores4–5 = anomalous,” with score 3 excluded as a gray zone for ROC and AUC calculation.

**Fig. 2:**
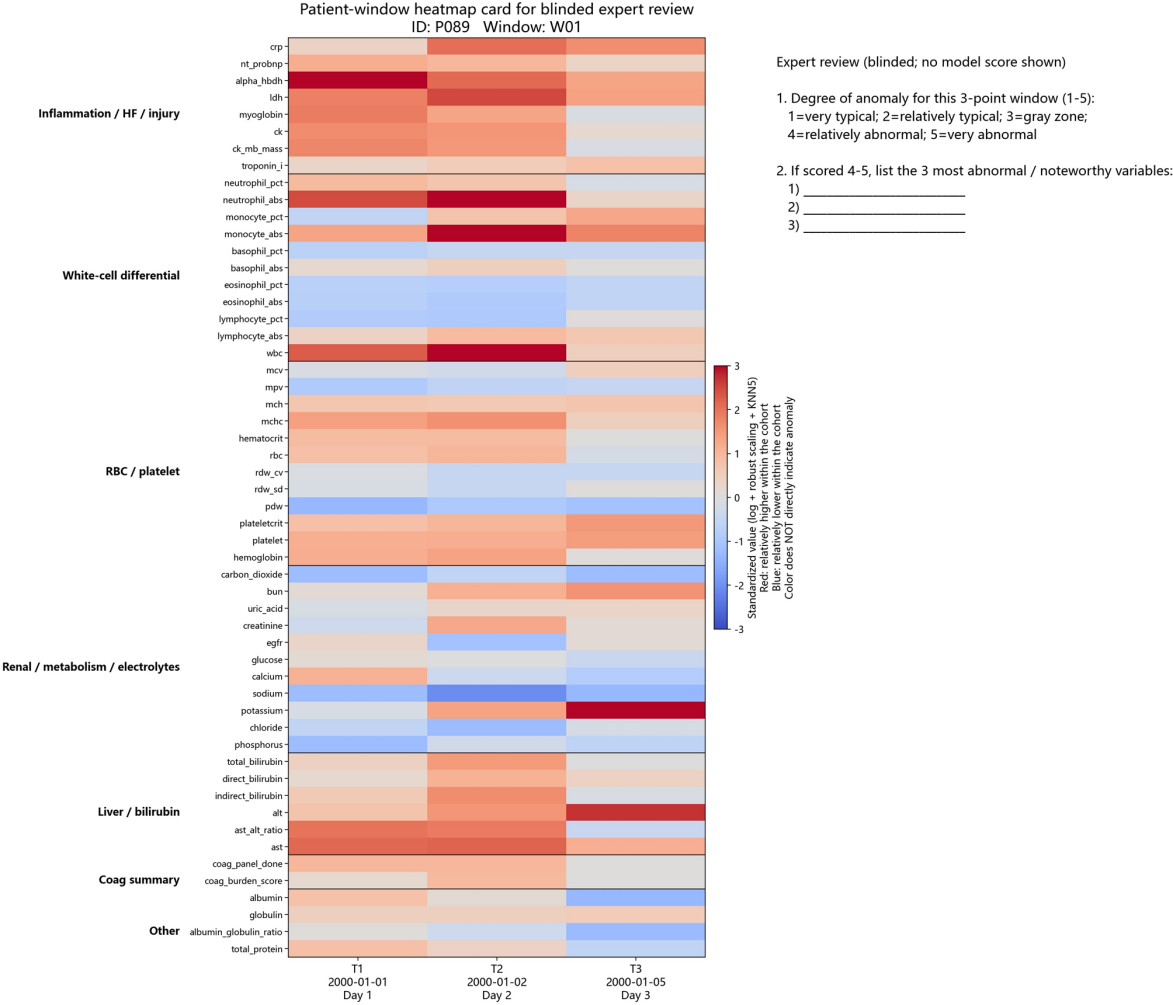
Example of the fixed-window heatmap card used for blinded expert review. This figure shows the structure of the fixed-window heatmap card used for blinded expert review. Under blinded conditions, the experts rated the cards solely on the basis of standardized heatmaps of the three-time-point windows, without access to model scores or model-derived classifications.

To improve the transparency of the evaluation process, Fig. 2 presents a structural example of the fixed-window heatmap card used for blinded expert review. Expert ratings were based on standardized heatmaps of windows composed of three consecutive monitoring time points, and the model-derived scores for the cards were not displayed at any stage of the review process. Because card examples in the main text are constrained by layout and resolution limitations, Fig. 2 shows only the structure of the card. To further enhance the transparency of the evaluation process, all anonymized fixed-window cards used for blinded review, the corresponding window list, and the final expert rating results used in the main analysis are provided as supplementary materials.

### External supportive survival association analysis in MIMIC-IV

To evaluate the relationship between the continuous DIF anomaly score, calculated from fixed three-time-point windows, and adverse prognosis after PCI in the MIMIC-IV cohort, we conducted an external supportive survival association analysis. The external cohort was constructed from the MIMIC-IV hospital module. First, hospital admissions for acute myocardial infarction were identified in the ‘diagnoses_icd’ table, retaining ICD-9 codes 410% and ICD-10 codes I21%. PCI-related procedures were then identified in the ‘procedures_icd’ table, retaining ICD-9 procedure codes 0066, 1755, 3606, 3607, and 3609, as well as ICD-10-PCS code 02703%. When multiple PCI-related dates were present within the same hospital admission, the earliest PCI date was defined as ‘first_pci_date’ and used as the starting point for the post-PCI laboratory observation window and survival time calculation.

We retained hospital admissions with complete admission and discharge times and a hospital stay longer than 3 days. Laboratory data were obtained from ‘labevents’, and only numeric laboratory records within 3 days after PCI were retained. Day 1, day 2, and day 3 were defined according to ‘day_number = floor((charttime - first_pci_date)/1 day) + 1’. Because not all laboratory variables in MIMIC-IV could form a stable 3-day continuous observation sequence after PCI, the external analysis did not fully reproduce all modeling variables used in the development cohort. Instead, it was conducted using a subset of overlapping laboratory variables that were available in both the development and external cohorts and showed relatively good continuous coverage within 3 days after PCI. Ultimately, 15 laboratory variables were included: potassium, sodium, chloride, platelet count, creatinine, urea nitrogen, bicarbonate, anion gap, hematocrit, glucose, magnesium, white blood cell count, hemoglobin, phosphate, and total calcium.

When the same laboratory variable was measured multiple times for the same patient within the same ‘day_number’, the mean value was calculated at the ‘hadm_id + day_number’ level. Only hospital admissions with records for all three time points—day 1, day 2, and day 3—were then retained, thereby forming complete fixed three-time-point windows. The final external cohort included 1,618 hospital admissions corresponding to 1,550 patients, with each admission contributing exactly one fixed three-time-point window. The MIMIC-IV cohort was processed using the same preprocessing framework as the development cohort: missing values were handled using k-nearest neighbors imputation (‘k = 5’), right-skewed variables were log-transformed, and robust standardization was subsequently applied. Window-level features were extracted from the 15 laboratory variables within each fixed three-time-point window, including the mean, the difference between the last and first observations, and the within-window variance, and the continuous DIF anomaly score was calculated accordingly. The outcomes were 30-day, 90-day, and 365-day mortality.

### Statistical analysis

The primary outcomes for model comparison included the Spearman rank correlation coefficient and Kendall rank correlation coefficient between the models’ continuous anomaly scores and the experts’ 1–5 ratings, as well as the area under the receiver operating characteristic curve (AUC), calculated based on expert-derived binary classification after exclusion of the gray-zone windows with a score of 3. In addition, to describe the trend of model scores across different expert rating groups, the sample size, mean, and median were calculated for each group, and the Kruskal–Wallis test was used to assess overall distributional differences across the five rating groups. Accuracy, sensitivity, specificity, and the F1 score were reported only as secondary outcomes.

To evaluate the impact of the imputation parameter on the robustness of the results, the primary model comparisons were repeated using modeling matrices based on KNN3, KNN5, and KNN7 under the same fixed-window framework and expert rating reference.

In the external MIMIC-IV cohort, the continuous DIF anomaly score was entered into Cox proportional hazards models per 1-standard-deviation increase to evaluate its associations with 30-day, 90-day, and 365-day mortality, respectively. Three levels of models were constructed: Model 1 was a univariable Cox model; Model 2 was adjusted for age, sex, and the Charlson Comorbidity Index; and Model 3 further included day 1 creatinine, urea nitrogen, white blood cell count, and hemoglobin in addition to Model 2. In addition, stratified analyses were performed according to quartiles of the DIF score to help illustrate the risk gradient. To evaluate the additional prognostic information provided by DIF beyond the baseline model, likelihood ratio tests were used to compare model fit before and after inclusion of the DIF score. All statistical tests were two-sided, and *P* < 0.05 was considered statistically significant.

## Results

### Baseline characteristics of the development cohort

The development cohort included 183 patients with MI who underwent PCI, with a median age of 62.0 years (IQR, 53.5–69.0); 84.2% were male, and 89.1% had STEMI. All patients underwent emergency PCI, and 85.2% had at least one comorbidity, with hypertension and diabetes being the most common. Killip class II was the most frequent, and the most common infarct locations were the inferior wall, anterior wall, and extensive anterior wall. Detailed results are presented in Table 1.

**Table 1:** Baseline characteristics of the development cohort.

Variable	Overall (*n* = 183)
Age, years	62.0 [53.5, 69.0]
Male sex, n (%)	154 (84.2%)
STEMI, n (%)	163 (89.1%)
NSTEMI, n (%)	19 (10.4%)
Missing AMI subtype, n (%)	1 (0.5%)
Body temperature, °C	36.3 [36.3, 36.5]
Pulse rate, beats/min	80.0 [70.0, 91.0]
Respiratory rate, breaths/min	20.0 [20.0, 20.0]
Systolic blood pressure, mmHg	130.0 [110.8, 145.0]
Diastolic blood pressure, mmHg	70.0 [70.0, 85.2]
Killip class I, n (%)	52 (28.4%)
Killip class II, n (%)	80 (43.7%)
Killip class III, n (%)	13 (7.1%)
Killip class IV, n (%)	8 (4.4%)
Missing Killip class, n (%)	30 (16.4%)
Emergency PCI, n (%)	183 (100.0%)
Any comorbidity, n (%)	156 (85.2%)
Hypertension, n (%)	105 (57.4%)
Diabetes mellitus, n (%)	61 (33.3%)
Cerebral infarction/stroke, n (%)	13 (7.1%)
Prior coronary artery disease/PCI/myocardial infarction, n (%)	51 (27.9%)
COPD/bronchiectasis/chronic lung disease, n (%)	6 (3.3%)
Repeat hospitalization, n (%)	67 (36.6%)
Cardiovascular readmission, n (%)	67 (36.6%)
Infarct location, n (%)	
Inferior wall	45 (24.6%)
Anterior wall	39 (21.3%)
Extensive anterior wall	33 (18.0%)
Posterior wall	10 (5.5%)
Inferoposterior wall	4 (2.2%)
Anteroseptal wall	3 (1.6%)
High lateral wall	1 (0.5%)
Missing	48 (26.2%)

Continuous variables are presented as median [interquartile range], and categorical variables as *n* (%). Some variables had missing data, and the extent of missingness is shown in the table. STEMI, ST-segment elevation myocardial infarction; NSTEMI, non-ST-segment elevation myocardial infarction; PCI, percutaneous coronary intervention; COPD, chronic obstructive pulmonary disease.

### Fixed-window construction and overview of blinded expert review

In the main analysis, the first complete set of three consecutive observations was extracted for each patient according to the prespecified rule to form a fixed window (T1–T2–T3). A total of 183 fixed windows were ultimately generated, with one window per patient. After completion of the blinded expert review, the distribution of ratings across the 183 windows was as follows: 65 windows were rated 1, 49 were rated 2, 22 were rated 3, 29 were rated 4, and 18 were rated 5. Overall, lower-rated windows were more common, whereas higher-rated windows were relatively fewer, and the proportion of gray-zone windows with a score of 3 was low, suggesting that most windows could be judged by the experts as either relatively typical recovery windows or relatively abnormal recovery windows. All anonymized fixed-window cards used for blinded review are provided in Supplementary File 1, and the corresponding window-level expert ratings and detailed model anomaly scores are provided in Supplementary Data 1.

### Comparison of the five models under the fixed-window framework

**Fig. 3:**
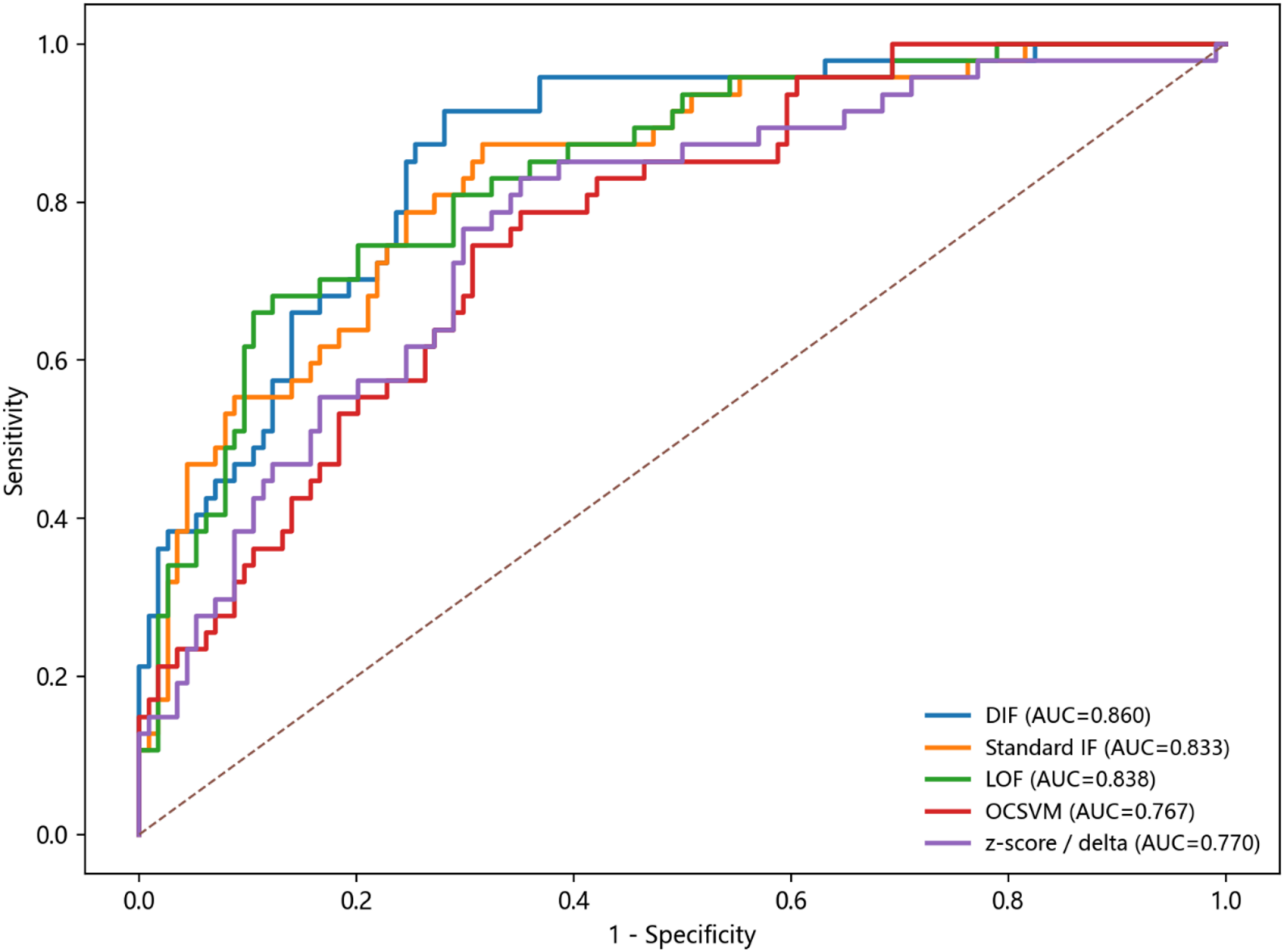
ROC curves of different models for identifying abnormal recovery windows under the fixed-window framework. This figure compares the discriminative performance of DIF, Standard IF, LOF, OCSVM, and the z-score/change-based approach in identifying abnormal recovery windows after exclusion of gray-zone windows with a score of 3.

Under the fixed-window framework and the unified expert rating reference, all five models showed trends consistent with expert judgment, with DIF performing best overall. Compared with the other methods, DIF ranked highest in both correlation metrics and AUC; LOF performed most similarly to DIF, followed by Standard IF, whereas OCSVM and the z-score/change-based approach were relatively weaker. Overall, these results suggest that DIF, which is based on the joint pattern of change across consecutive observations, achieved better overall performance in identifying abnormal recovery windows in laboratory data after PCI (Table 2; Fig. 3).

**Table 2:** Comparison of model performance under the fixed-window framework.

Model	ρ	τ	KW *p*	AUC	Acc	Sens	Spec	F1
DIF	0.586	0.453	< 0.001	0.860	0.776	0.915	0.719	0.705
StandardIF	0.548	0.421	< 0.001	0.833	0.739	0.872	0.684	0.661
LOF	0.553	0.414	< 0.001	0.838	0.820	0.681	0.877	0.688
OCSVM	0.446	0.334	< 0.001	0.767	0.708	0.745	0.693	0.598
ZDelta	0.467	0.358	< 0.001	0.770	0.702	0.830	0.649	0.619

ρ denotes Spearman’s rank correlation coefficient, τ denotes Kendall’s rank correlation coefficient, KW *p* denotes the *P* value from the Kruskal–Wallis test, AUC denotes the area under the receiver operating characteristic curve, Acc denotes accuracy, Sens denotes sensitivity, Spec denotes specificity, and F1 denotes the F1 score. AUC was calculated based on expert-derived binary classification results (scores 1–2 = non-anomalous, scores 4–5 = anomalous, with score 3 excluded as the gray zone).

### Model scores increased with increasing expert ratings

**Fig. 4:**
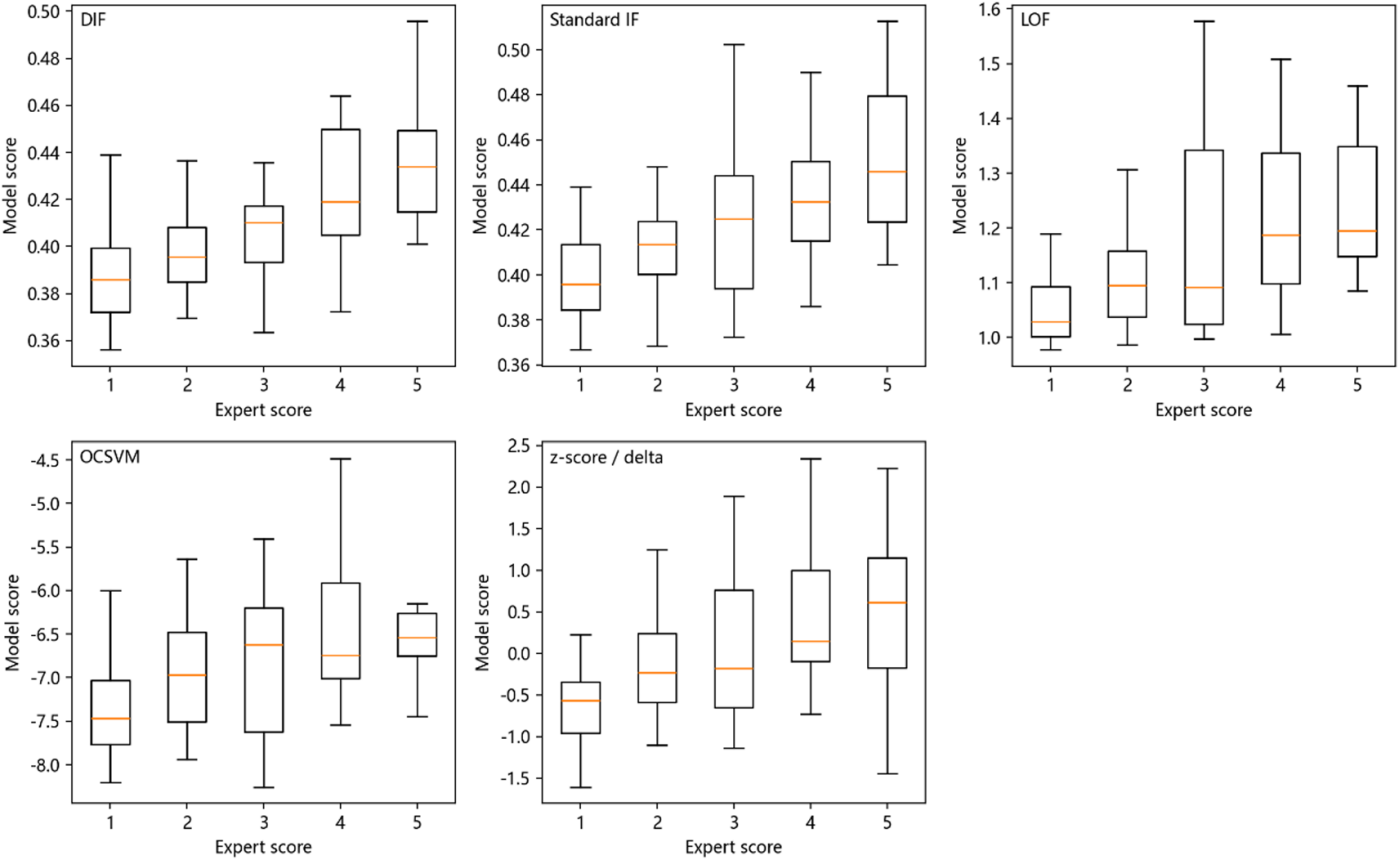
Distribution of model scores across expert rating groups. This figure shows the distribution of model scores across expert rating groups from 1 to 5 and allows comparison of how model scores change as the degree of abnormality judged by the experts increases.

After stratification by expert ratings from 1 to 5, the anomaly scores of all models showed an overall increasing trend, with DIF exhibiting the most stable and clearest pattern. As the degree of abnormality judged by the experts increased, DIF scores increased accordingly, suggesting that its continuous anomaly score better reflected the extent to which a window deviated from the typical postoperative recovery trajectory. The other models showed trends in the same direction, although with varying strength. Among them, LOF and Standard IF displayed relatively clear trends, whereas the z-score/change-based approach showed relatively weaker discrimination across the higher-rating groups. The distributions and trend patterns of each model across the different expert rating groups are shown in Fig. 4. To provide a more concise summary of the overall trend of score changes across expert rating groups for different models, a plot of standardized mean trends across rating groups is additionally provided in Supplementary Fig. S1.

### Robustness analyses

To assess the robustness of the main analysis results, we conducted two supplementary analyses. First, under the same fixed-window framework and expert rating reference, the primary model comparisons were repeated using modeling matrices based on KNN3, KNN5, and KNN7. The results showed that DIF’s correlation metrics and AUC remained generally stable, and the relative ranking of the models did not change materially. Second, in the patient-level bootstrap resampling analysis, the agreement between DIF and expert ratings, as well as the AUC, also remained generally stable across repeated resampling. The results of the KNN sensitivity analysis are presented in Supplementary Table S1, the summary results of the bootstrap stability analysis are shown in Supplementary Table S2, and the results from each resampling iteration are provided in Supplementary Data 2. These findings further support the robustness of the main analysis results.

### Baseline characteristics of the external MIMIC-IV cohort

The external MIMIC-IV cohort included 1,618 hospital admissions corresponding to 1,550 patients, and each admission contributed one complete fixed three-time-point window after PCI. The patients were older and had a substantial burden of comorbidity, with a 365-day mortality rate of 22.1%, suggesting that this cohort overall represented a high-risk post-PCI population. The baseline characteristics are shown in Supplementary Table S3.

### Association between continuous DIF score and mortality

The external MIMIC-IV cohort included 1,618 hospital admissions corresponding to 1,550 patients, and each admission contributed one complete fixed three-time-point window after PCI. The cohort was characterized by older age and a substantial comorbidity burden, with a 365-day mortality rate of 22.1%, suggesting that it represented an overall high-risk post-PCI population. The results of the Cox regression analysis examining the association between the continuous DIF anomaly score and mortality risk are shown in Table 3.

**Table 3:** Cox regression results for the association between the continuous DIF anomaly score and mortality risk.

Outcome	Model 1, HR (95% CI), *P* value	Model 2, HR (95% CI), *P* value	Model 3, HR (95% CI), *P* value
30-day mortality	1.70(1.50–1.93), 8.95 × 10⁻^17^	1.80(1.58–2.06), 3.76 × 10⁻^18^	1.61(1.36–1.90), 1.83 × 10⁻^8^
90-day mortality	1.55(1.39–1.73), 1.02 × 10⁻^15^	1.63(1.45–1.82), 5.24 × 10⁻^17^	1.44(1.25–1.66), 5.75 × 10⁻^7^
365-day mortality	1.48(1.36–1.61), 2.10 × 10⁻^19^	1.52(1.39–1.66), 7.81 × 10⁻^20^	1.37(1.22–1.53), 4.55 × 10⁻^8^

Model 1 was a univariable Cox regression model; Model 2 was further adjusted for age, sex, and the Charlson Comorbidity Index on the basis of Model 1; Model 3 was further adjusted for day 1 laboratory proxy indicators on the basis of Model 2. HR denotes hazard ratio, and CI denotes confidence interval.

### Incremental prognostic value of DIF score

Likelihood ratio tests showed that model fit improved significantly after inclusion of the DIF score in the baseline models. Even after further adjustment for day 1 laboratory surrogate indicators reflecting baseline disease severity, the DIF score still provided additional prognostic information. Detailed results are presented in Supplementary Table S4.

### Risk gradient across DIF score quartiles

**Fig. 5:**
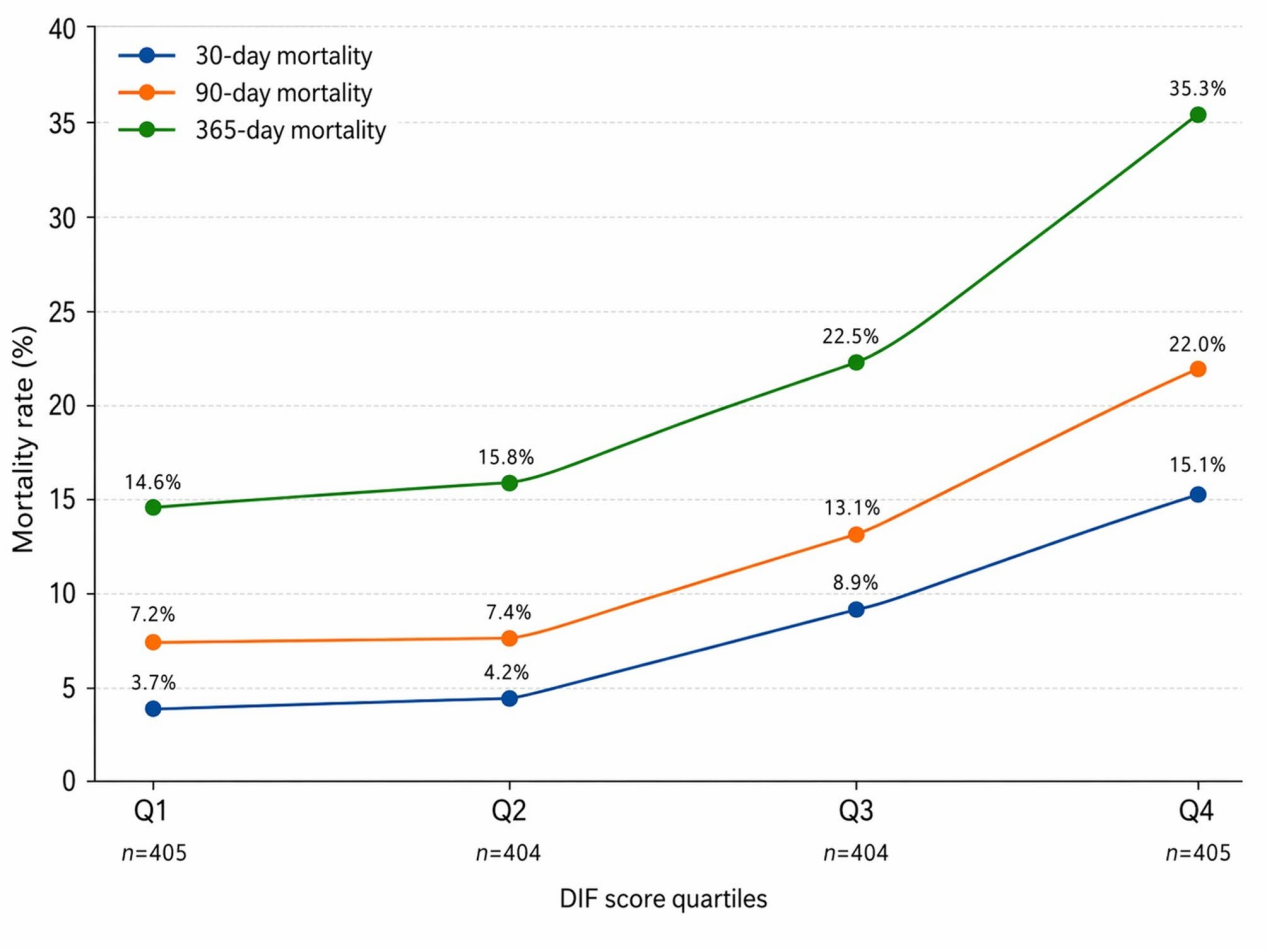
Mortality stratified by quartiles of the DIF score. This figure shows the trends in 30-day, 90-day, and 365-day mortality across quartiles of the DIF score in the external MIMIC-IV cohort.

After stratification by quartiles of the DIF anomaly score, 30-day, 90-day, and 365-day mortality all showed increasing trends, consistent with a higher risk of death at higher DIF anomaly scores. The corresponding stratified results are shown in Fig. 5 as supportive evidence.

## Discussion

This study evaluated the feasibility of monitoring dynamic laboratory abnormalities during hospitalization in patients with myocardial infarction who underwent PCI under a fixed three-time-point window framework and a blinded expert review framework. The results showed that, under a unified window definition and reference standard, DIF generally outperformed Standard IF, LOF, OCSVM, and the z-score/change-based approach in major metrics, including correlation and AUC, suggesting that DIF showed relatively strong overall performance in identifying abnormal windows. After stratification by expert rating, the scores of all models generally increased with higher ratings, with DIF showing the most stable and clearest trend, indicating that its continuous anomaly score was directionally consistent with expert judgment of abnormality severity.

The advantage of DIF may lie in the way it integrates short-term dynamic changes. In this study, an anomaly did not refer to a single indicator exceeding its reference range at one time point, but rather to a marked deviation in the joint pattern of laboratory changes within a fixed three-time-point window from the typical postoperative recovery trajectory. Compared with methods that focus on single values or simple changes, DIF simultaneously encodes the overall level, direction of change, and magnitude of fluctuation within the window, and may therefore be better suited to identifying early dynamic laboratory abnormalities after PCI. In addition, the sensitivity analyses based on KNN3, KNN5, and KNN7 were generally consistent, indicating that the main findings were relatively robust to the choice of imputation parameter.

The MIMIC-IV analysis provided external support for the survival association observed in this study. We found that higher DIF anomaly scores were associated with increased risks of 30-day, 90-day, and 365-day mortality, and this association remained after adjustment for age, sex, the Charlson Comorbidity Index, and selected baseline laboratory indicators. This suggests that the DIF score may reflect not only short-term dynamic laboratory abnormalities but also show prognostic relevance. It should be emphasized that the MIMIC-IV findings support only an association between higher anomaly scores and poorer prognosis and cannot serve as independent validation of anomaly detection accuracy.

This study still has several limitations. The development cohort was derived from single-center retrospective data, and the sample size was limited; therefore, the findings still require validation in larger, multicenter populations. In addition, we did not further include supervised learning baseline models because the current study lacked a natural, stable, and uncontroversial window-level gold-standard label; training supervised models on the same expert-derived ratings that served as the primary reference standard could introduce methodological circularity and thereby weaken the independence of the comparison. Window length may affect the performance of dynamic anomaly detection, but in the current study, we did not conduct a systematic comparison of different window lengths. This was because the main formal analysis of this study had been prespecified as a fixed three-time-point window framework, with blinded expert review under that window definition serving as the primary clinical reference. Further comparison of longer windows would require redefining the unit of analysis, adjusting the study population, and reconstructing the expert evaluation framework, and therefore fell beyond the scope of the present study. Future studies may systematically evaluate, under independent designs, the impact of different window lengths on model performance and clinical interpretability. In addition, the external MIMIC-IV analysis was conducted using 15 overlapping laboratory variables available in both cohorts and did not fully reproduce all modeling variables used in the development cohort. Its findings should therefore be interpreted as external support rather than strict external validation.

## Conclusion

Under the fixed three-time-point window and blinded expert review framework, DIF was able to identify abnormal windows in the dynamic inpatient laboratory changes of patients with myocardial infarction who underwent PCI that deviated from the typical recovery trajectory, and it showed prognostic relevance in the external MIMIC-IV cohort. Based on the current evidence, DIF is more appropriately regarded as an exploratory tool for monitoring dynamic postoperative laboratory abnormalities rather than a clinically validated decision-making model. Its clinical utility still requires further validation in larger, multicenter, and prospective studies.

## Supplementary Information

Below is the link to the electronic supplementary material.

Supplementary Material 1

Supplementary Material 2

Supplementary Material 3

Supplementary Material 4

## Data Availability

The de-identified data and analysis code used in this study have been organized and archived in Zenodo and are publicly available at https://doi.org/10.5281/zenodo.19766746. The public archive provides a de-identified release version together with the materials necessary to reproduce the main analytical workflow of this study.

## Code availability

The custom code used to generate the main analytical results in this study has been archived in Zenodo and is publicly available at 10.5281/zenodo.19766746. This code package contains the core scripts required for fixed-window construction, five-model comparison, KNN sensitivity analysis, and patient-level bootstrap stability analysis. Because the original clinical data involve patient privacy, the public archive provides a de-identified release version together with the materials necessary to reproduce the main analytical workflow of this study.
