# Supplementary material for: Dynamic isolation forest for anomaly detection in post-PCI myocardial infarction patients
Source: Sci Rep. 2026 May 22;16:23457. doi: 10.1038/s41598-026-54390-7 (PMC13407892; doi:10.1038/s41598-026-54390-7)
Supplement: Supplementary file 1 — Supplementary Material 1 [file 41598_2026_54390_MOESM1_ESM.docx]

**Dynamic Isolation Forest for Anomaly Detection in Post-PCI Myocardial Infarction Patients**

**Yu Zhang^a,*^ Shan Gao^a,1^ He Xu^b,1^ Yuan Liu^a,2^ Yaqiong Guo^a,3^ XueLing Wei^a,4^**

a The Second People's Hospital of Gansu Province（Affiliated Hospital of Northwest Minzu University）, Medical laboratory ,Lanzhou, 743000, China

b Xinjiang Baoshihua Hospital, Medical laboratory, Urumqi, 830000, China

***Corresponding Author**  Email: [01585@xbmu.edu.cn](mailto:01585@xbmu.edu.cn)

| **KNN** | **Model** | **Spearman ρ** | **Kendall τ** | **AUC** |
| --- | --- | --- | --- | --- |
| KNN3 | DIF | 0.541894777 | 0.419546426 | 0.835572975 |
| KNN3 | Standard IF | 0.552771813 | 0.426033902 | 0.82941396 |
| KNN3 | z-score/delta | 0.44547914 | 0.339350185 | 0.762224711 |
| KNN5 | DIF | 0.585524795 | 0.45253593 | 0.860209033 |
| KNN5 | Standard IF | 0.547886795 | 0.420512646 | 0.83296006 |
| KNN5 | z-score/delta | 0.466785335 | 0.357708361 | 0.77043673 |
| KNN7 | DIF | 0.56918385 | 0.440941292 | 0.849757372 |
| KNN7 | Standard IF | 0.537291079 | 0.408365883 | 0.831466965 |
| KNN7 | z-score/delta | 0.470190629 | 0.360054895 | 0.773982829 |

Supplementary Table S1 Sensitivity analyses using KNN3, KNN5, and KNN7

**Table legend:** This table presents the main performance results of each model under different KNN imputation settings (k = 3, 5, and 7), using a common fixed-window framework, expert rating reference standard, and model evaluation framework. Spearman’s ρ and Kendall’s τ were used to assess the agreement between the model’s continuous anomaly scores and the experts’ ordered 1–5 ratings. AUC was calculated after excluding windows with an expert rating of 3 as the gray zone, with expert ratings of 1–2 defined as non-anomalous recovery windows and ratings of 4–5 defined as anomalous recovery windows. KNN5 was used as the primary imputation strategy, whereas KNN3 and KNN7 were used to assess the robustness of the results to the choice of imputation parameter.

| **Model** | **Original Spearman ρ** | **Bootstrap Spearman ρ, mean (95% CI)** | **Original Kendall τ** | **Bootstrap Kendall τ, mean (95% CI)** | **Original AUC** | **Bootstrap AUC, mean (95% CI)** |
| --- | --- | --- | --- | --- | --- | --- |
| DIF | 0.586 | 0.585 (0.494–0.672) | 0.453 | 0.454 (0.378–0.527) | 0.860 | 0.860 (0.802–0.916) |
| Standard IF | 0.548 | 0.548 (0.447–0.645) | 0.421 | 0.422 (0.340–0.502) | 0.833 | 0.831 (0.763–0.898) |
| LOF | 0.553 | 0.554 (0.463–0.640) | 0.414 | 0.417 (0.344–0.490) | 0.838 | 0.837 (0.769–0.901) |
| OCSVM | 0.446 | 0.445 (0.334–0.543) | 0.334 | 0.335 (0.249–0.413) | 0.767 | 0.765 (0.691–0.833) |
| z-score / delta | 0.467 | 0.466 (0.336–0.579) | 0.358 | 0.358 (0.262–0.447) | 0.770 | 0.769 (0.683–0.835) |

Supplementary Table S2 Patient-level bootstrap resampling results

**Table legend:** This table presents the results of the model performance stability analysis based on patient-level bootstrap resampling. In each resampling iteration, sampling was performed at the patient level to reduce the impact of within-patient correlation on stability assessment. The bootstrap results were based on 500 valid resampling iterations. Bootstrap estimates are presented as the mean and percentile-based 95% confidence interval, with the interval defined by the 2.5th and 97.5th percentiles of the bootstrap distribution. Original denotes the model performance obtained from the original sample. The full bootstrap output, including the bootstrap median, standard deviation, and number of valid resampling iterations, is provided in Supplementary Data 2.

| **Variable** | **Value** |
| --- | --- |
| Hospital admissions (hadm_id), n | 1,618 |
| Patients (subject_id), n | 1,550 |
| Age, years | 71.5 ± 13.1 |
| Age, median (IQR), years | 73.0 (63.0–82.0) |
| Male sex, n (%) | 949 (58.7) |
| Length of hospital stay, median (IQR), days | 5.95 (4.04–9.71) |
| In-hospital mortality, n (%) | 88 (5.4) |
| 30-day mortality, n (%) | 129 (8.0) |
| 90-day mortality, n (%) | 201 (12.4) |
| 365-day mortality, n (%) | 357 (22.1) |
| Charlson Comorbidity Index, mean | 6.46 |
| Congestive heart failure, n (%) | 993 (61.4) |
| Renal disease, n (%) | 535 (33.1) |
| Diabetes without chronic complications, n (%) | 505 (31.2) |
| Chronic pulmonary disease, n (%) | 438 (27.1) |
| Diabetes with chronic complications, n (%) | 311 (19.2) |

Supplementary Table S3 Baseline characteristics of the external MIMIC-IV cohort

**Table legend:** This table presents the baseline characteristics of the MIMIC-IV cohort used for the external supportive prognostic analysis. The external cohort included 1,618 hospital admissions corresponding to 1,550 patients, with each admission contributing one complete fixed three-time-point laboratory observation window after PCI. Continuous variables are presented as mean ± standard deviation or median (interquartile range), as appropriate to their distribution, and categorical variables are presented as counts and percentages. The Charlson Comorbidity Index was used to characterize comorbidity burden. Mortality outcomes included in-hospital mortality, 30-day mortality, 90-day mortality, and 365-day mortality.

| **Outcome** | **Base model + DIF score, LRT P value** | **Extended model + DIF score, LRT P value** |
| --- | --- | --- |
| 30-day mortality | 3.11 × 10⁻¹⁵ | 1.85 × 10⁻⁷ |
| 90-day mortality | 1.35 × 10⁻¹⁴ | 2.32 × 10⁻⁶ |
| 365-day mortality | 2.69 × 10⁻¹⁷ | 1.86 × 10⁻⁷ |

Supplementary Table S4 Likelihood ratio test results for improvement in Cox model fit after adding the DIF anomaly score

**Table legend:** This table presents the improvement in model fit after adding the continuous DIF anomaly score to Cox proportional hazards models in the external MIMIC-IV cohort. The baseline model was adjusted for age, sex, and the Charlson Comorbidity Index. The extended model further included day 1 creatinine, urea nitrogen, white blood cell count, and hemoglobin in addition to the baseline model variables, to partially reflect early disease severity after PCI. Likelihood ratio tests were used to assess whether model fit improved significantly after inclusion of the DIF anomaly score. Smaller LRT P values suggest that the DIF anomaly score may provide additional prognostic information beyond the corresponding adjusted model.

**
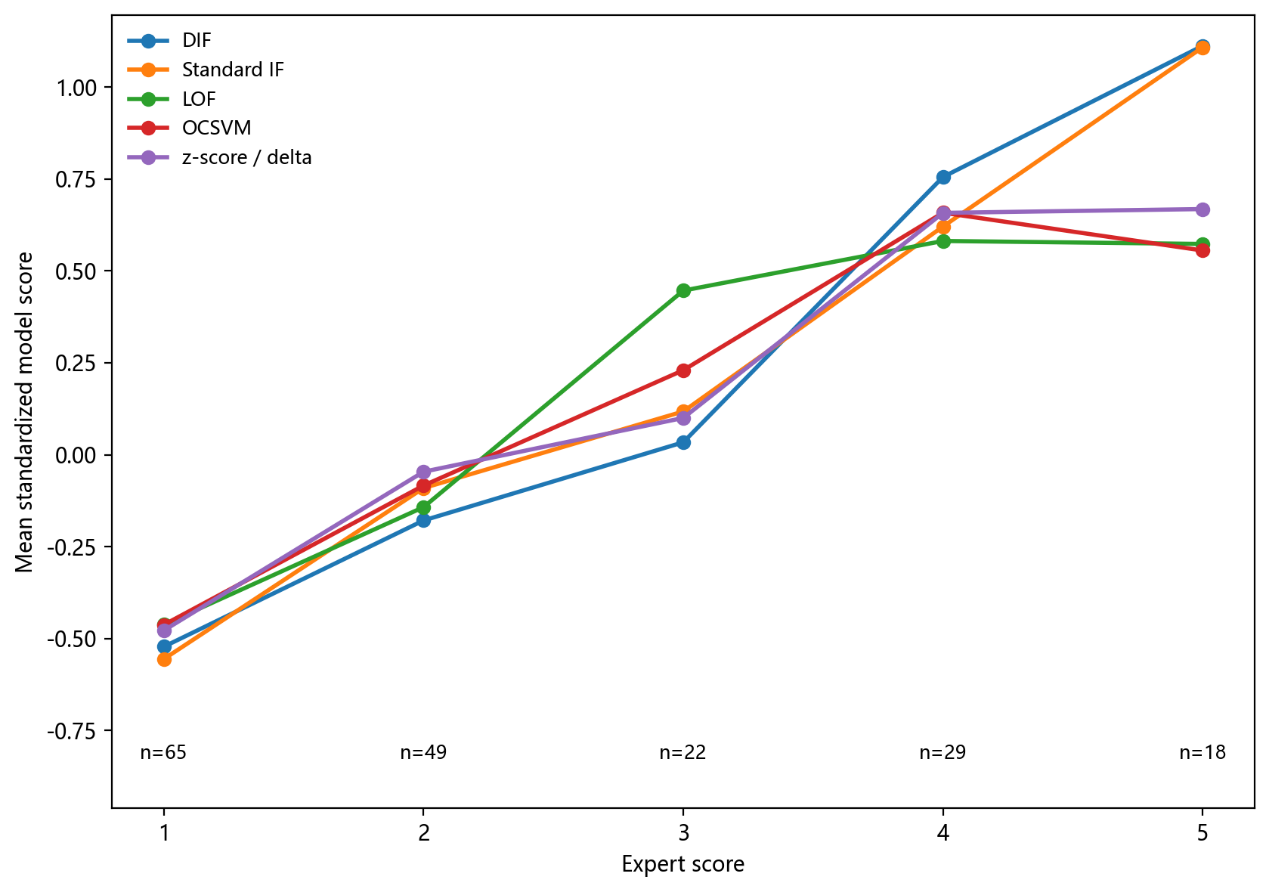
**

Supplementary Figure S1 Standardized mean trends across expert rating groups

**Figure legend:** This figure shows the mean trends of standardized anomaly scores from different models across expert rating groups from 1 to 5. Higher expert ratings indicate a greater deviation of the joint laboratory change pattern within a window from the typical postoperative recovery trajectory observed in the local PCI cohort. To facilitate comparison across models, the anomaly scores from all models were standardized before plotting. The overall trend is intended to illustrate the directional consistency between the models’ continuous anomaly scores and the degree of abnormality judged by the experts. This figure is provided as a supplementary illustration of the model score distribution results presented in the main text.
