# Supplementary material for: Dynamic isolation forest for anomaly detection in post-PCI myocardial infarction patients
Source: Sci Rep. 2026 May 22;16:23457. doi: 10.1038/s41598-026-54390-7 (PMC13407892; doi:10.1038/s41598-026-54390-7)
Supplement: Supplementary file 4 — Supplementary Material 4 [file 41598_2026_54390_MOESM4_ESM.pdf]

Patient-window heatmap card for blinded expert review
ID: P001 Window: W01

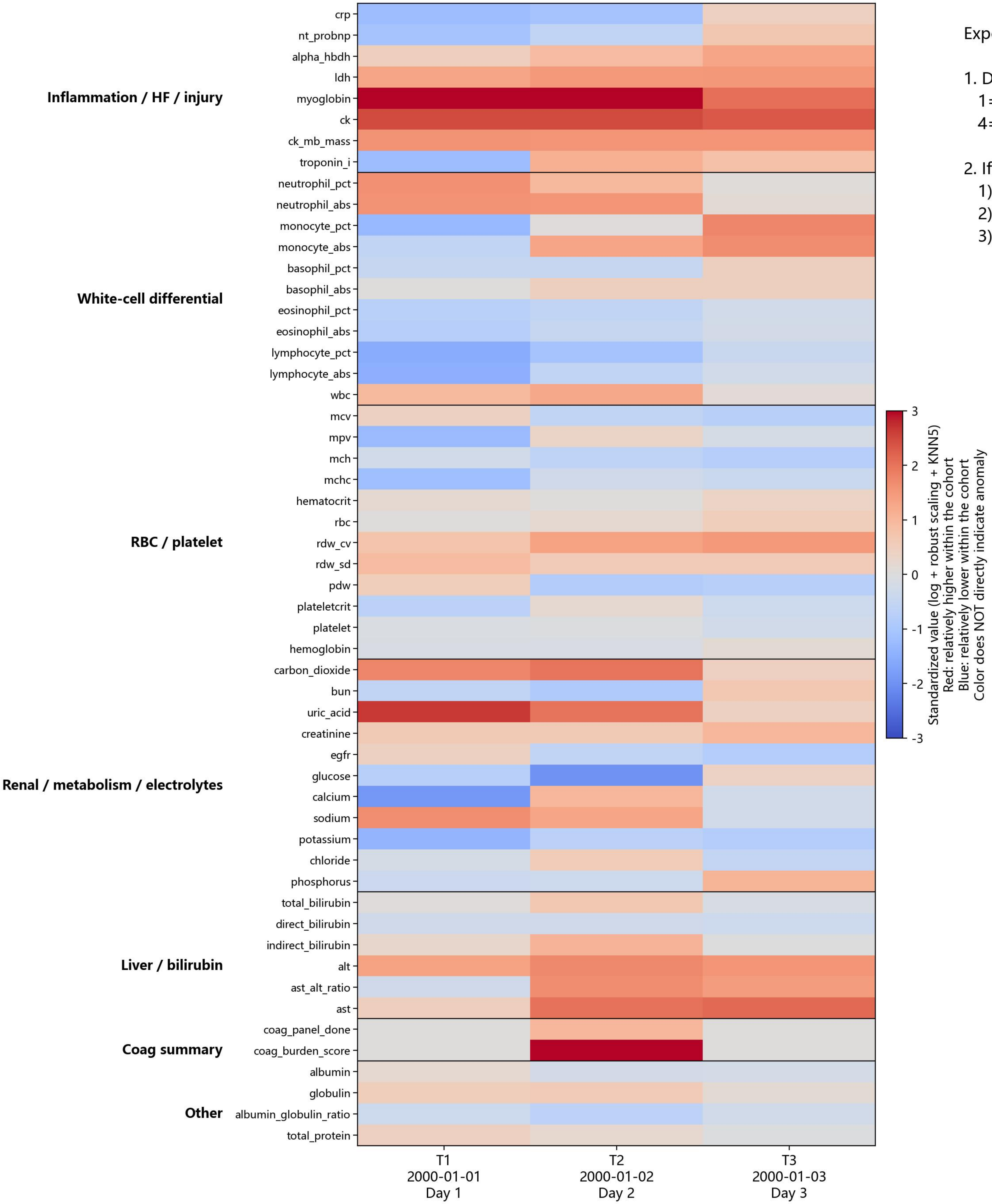

Expert review (blinded; no model score shown)

1. Degree of anomaly for this 3-point window (1-5):
1=very typical; 2=relatively typical; 3=gray zone;
4=relatively abnormal; 5=very abnormal

2. If scored 4-5, list the 3 most abnormal / noteworthy variables:
1) ____________________________
2) ____________________________
3) ____________________________

Patient-window heatmap card for blinded expert review
ID: P002   Window: W01

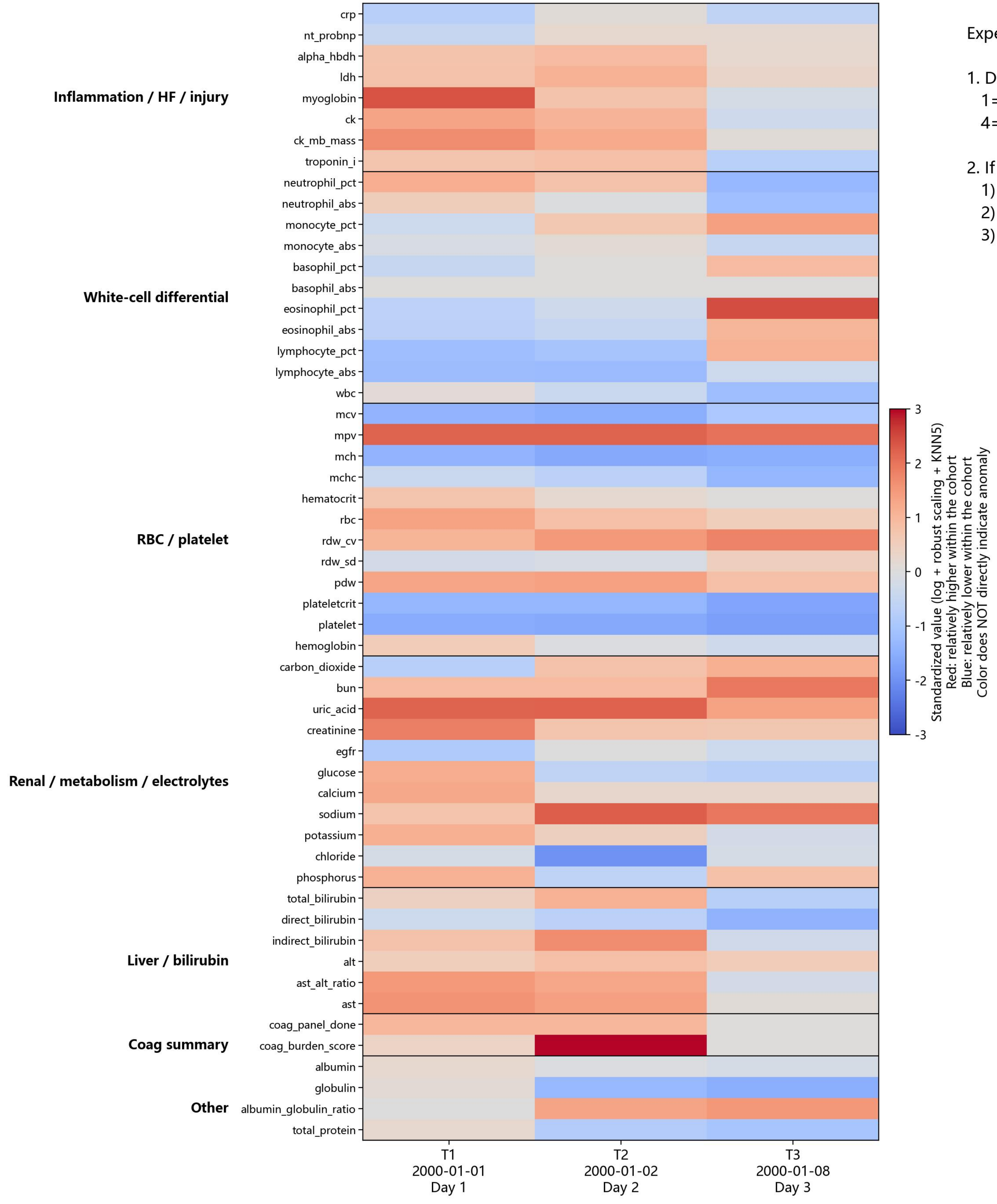

Expert review (blinded; no model score shown)

1. Degree of anomaly for this 3-point window (1-5):
   1=very typical; 2=relatively typical; 3=gray zone;
   4=relatively abnormal; 5=very abnormal

2. If scored 4-5, list the 3 most abnormal / noteworthy variables:
   1) ________________________
   2) ________________________
   3) ________________________

Patient-window heatmap card for blinded expert review
ID: P003   Window: W01

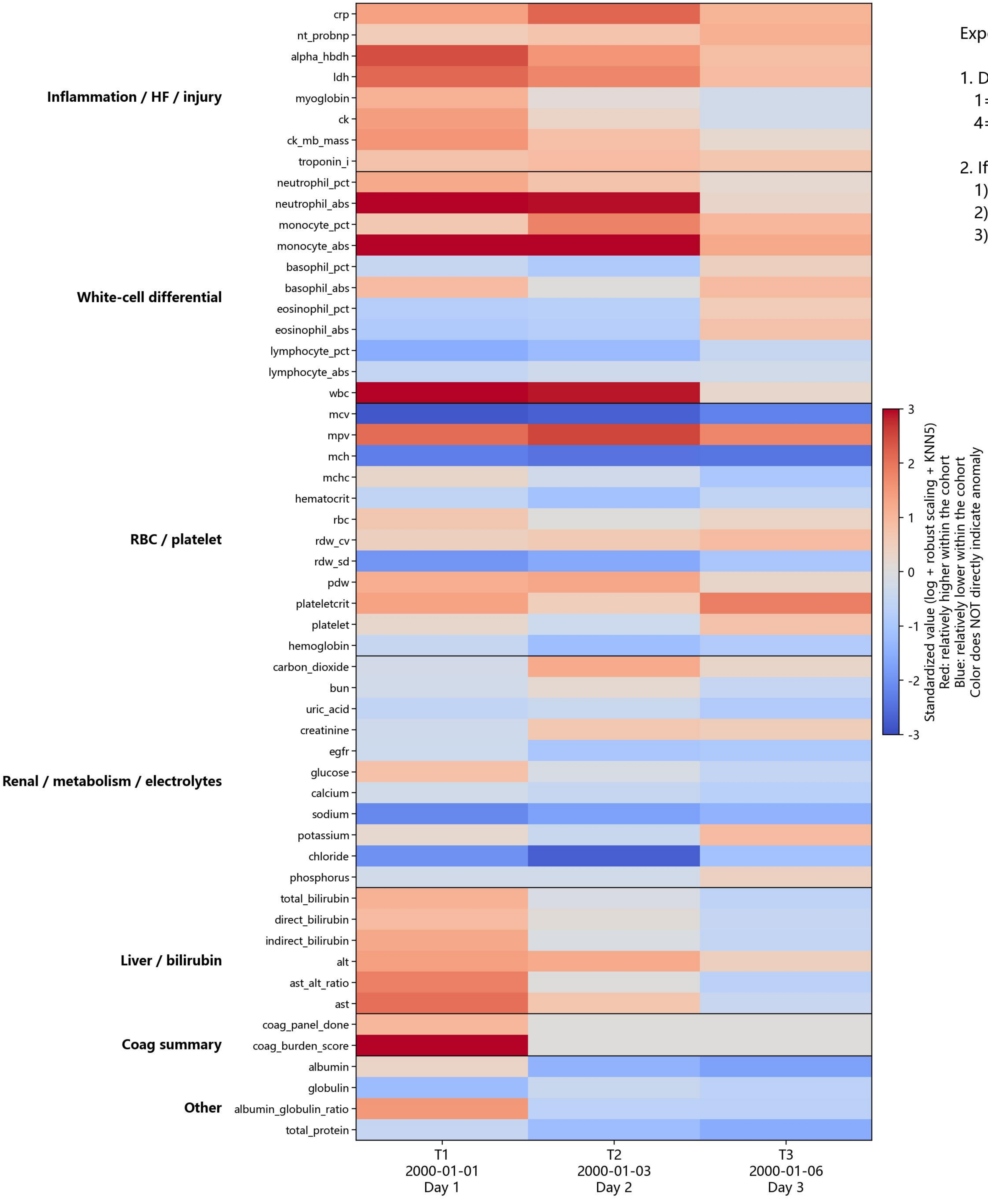

Expert review (blinded; no model score shown)

1. Degree of anomaly for this 3-point window (1-5):
   1=very typical; 2=relatively typical; 3=gray zone;
   4=relatively abnormal; 5=very abnormal

2. If scored 4-5, list the 3 most abnormal / noteworthy variables:
   1) ________________________
   2) ________________________
   3) ________________________

Patient-window heatmap card for blinded expert review
ID: P004   Window: W01

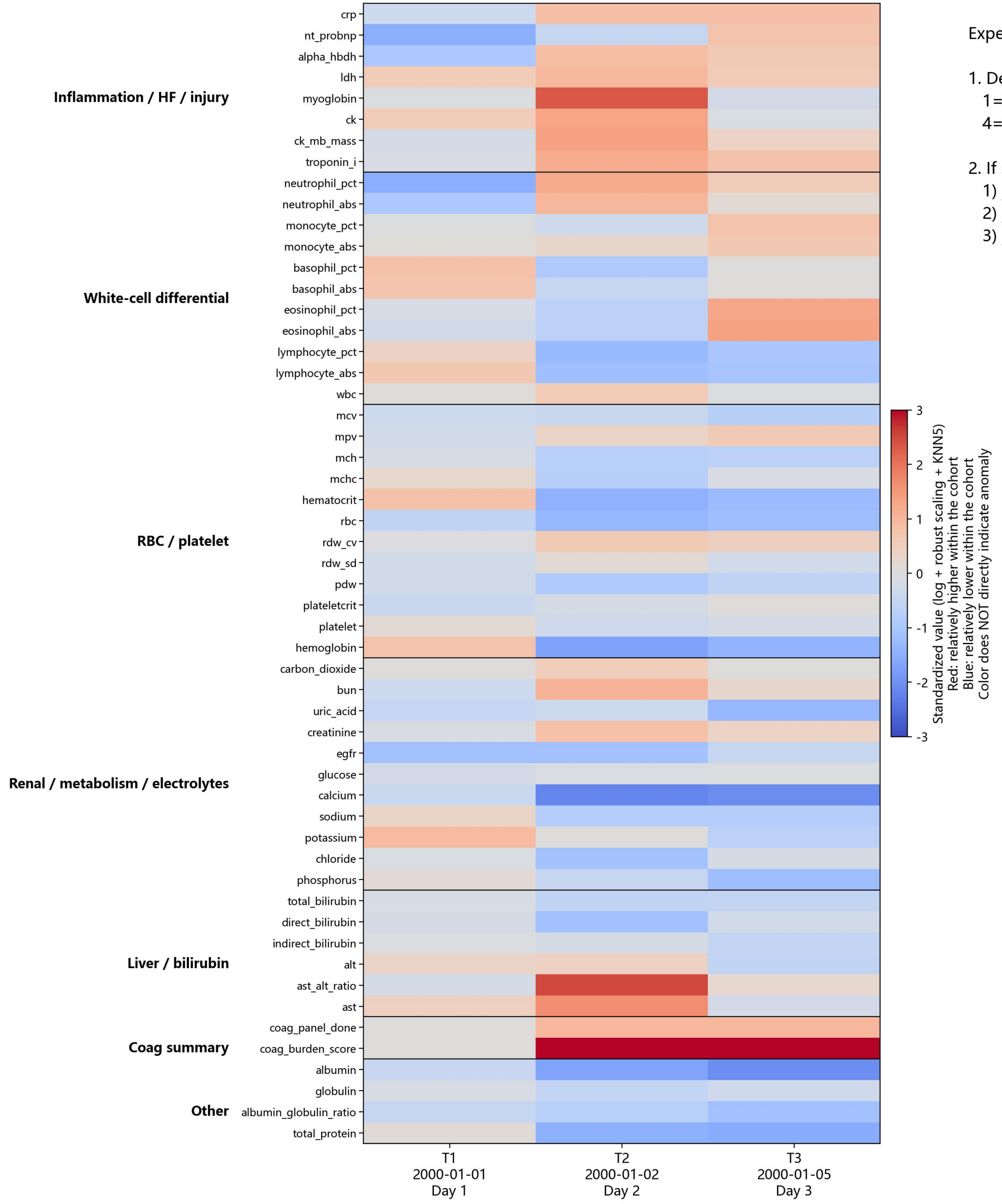

Expert review (blinded; no model score shown)

1. Degree of anomaly for this 3-point window (1-5):
   1=very typical; 2=relatively typical; 3=gray zone;
   4=relatively abnormal; 5=very abnormal

2. If scored 4-5, list the 3 most abnormal / noteworthy variables:
   1) ________________________
   2) ________________________
   3) ________________________

Patient-window heatmap card for blinded expert review
ID: P005   Window: W01

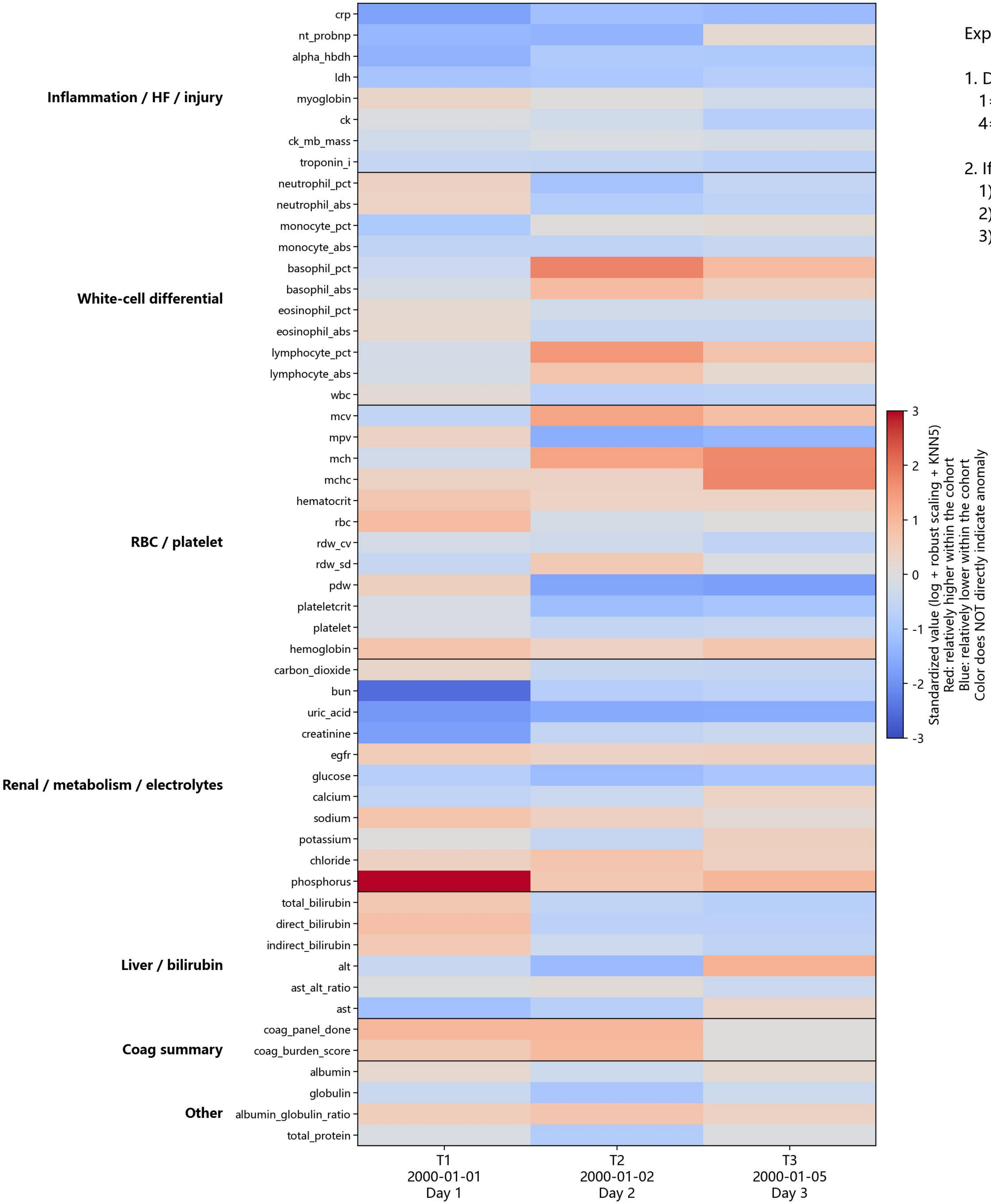

Expert review (blinded; no model score shown)

1. Degree of anomaly for this 3-point window (1-5):
   1=very typical; 2=relatively typical; 3=gray zone;
   4=relatively abnormal; 5=very abnormal

2. If scored 4-5, list the 3 most abnormal / noteworthy variables:
   1) ________________________
   2) ________________________
   3) ________________________

Patient-window heatmap card for blinded expert review
ID: P006 Window: W01

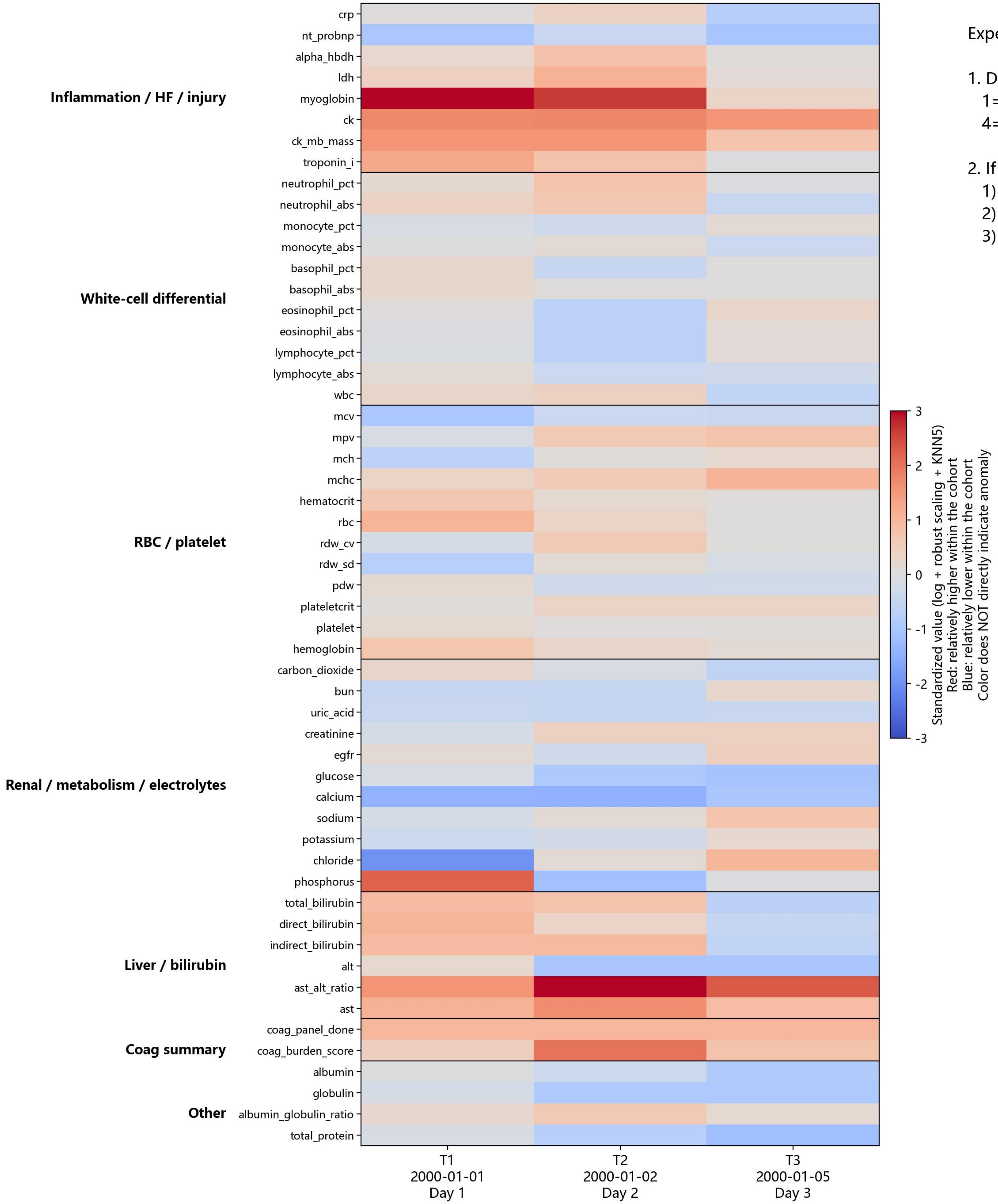

Expert review (blinded; no model score shown)

1. Degree of anomaly for this 3-point window (1-5):
   1=very typical; 2=relatively typical; 3=gray zone;
   4=relatively abnormal; 5=very abnormal

2. If scored 4-5, list the 3 most abnormal / noteworthy variables:
   1) ________________________
   2) ________________________
   3) ________________________

Patient-window heatmap card for blinded expert review
ID: P007   Window: W01

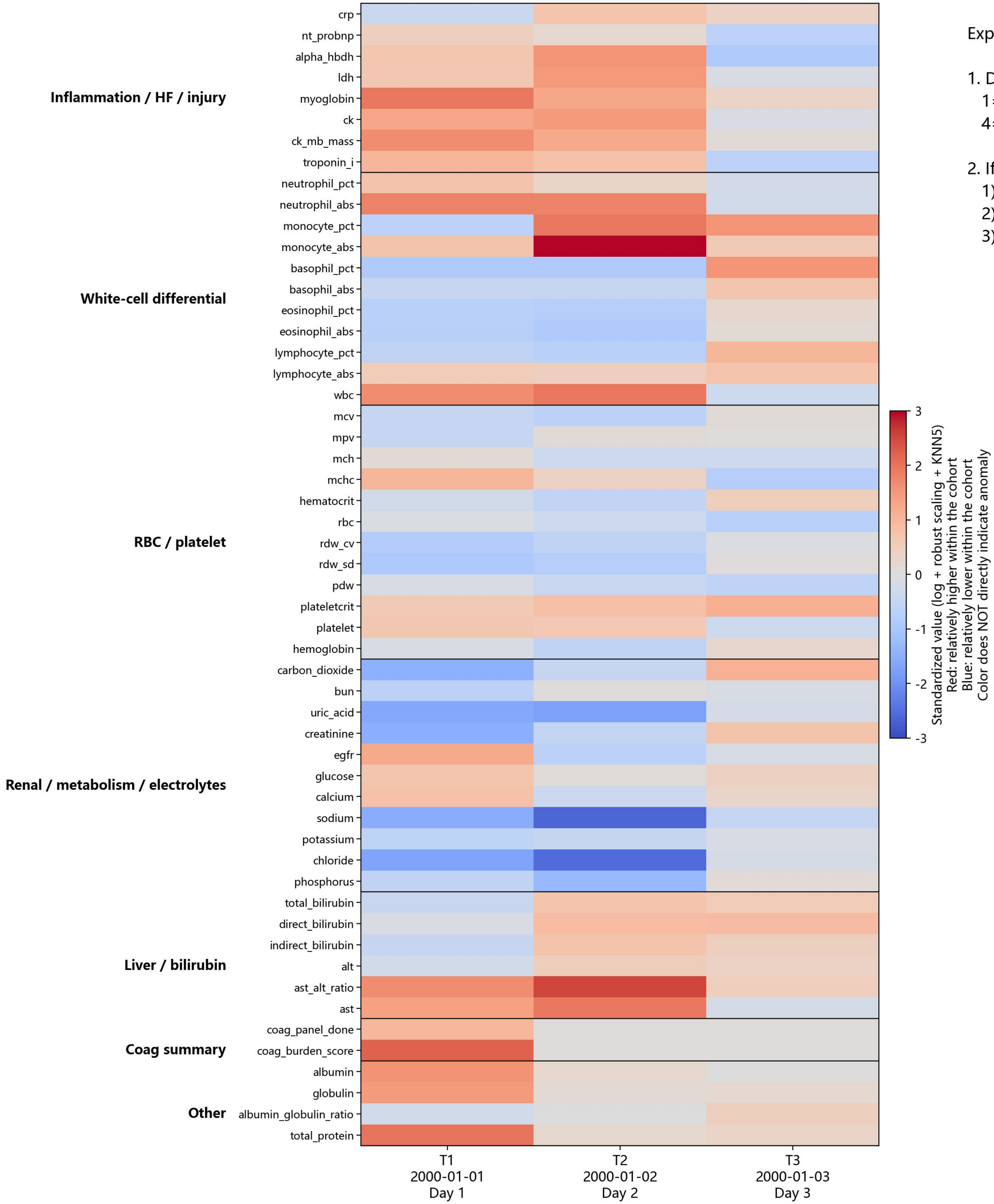

Expert review (blinded; no model score shown)

1. Degree of anomaly for this 3-point window (1-5):
   1=very typical; 2=relatively typical; 3=gray zone;
   4=relatively abnormal; 5=very abnormal

2. If scored 4-5, list the 3 most abnormal / noteworthy variables:
   1) ________________________
   2) ________________________
   3) ________________________

Patient-window heatmap card for blinded expert review
ID: P008   Window: W01

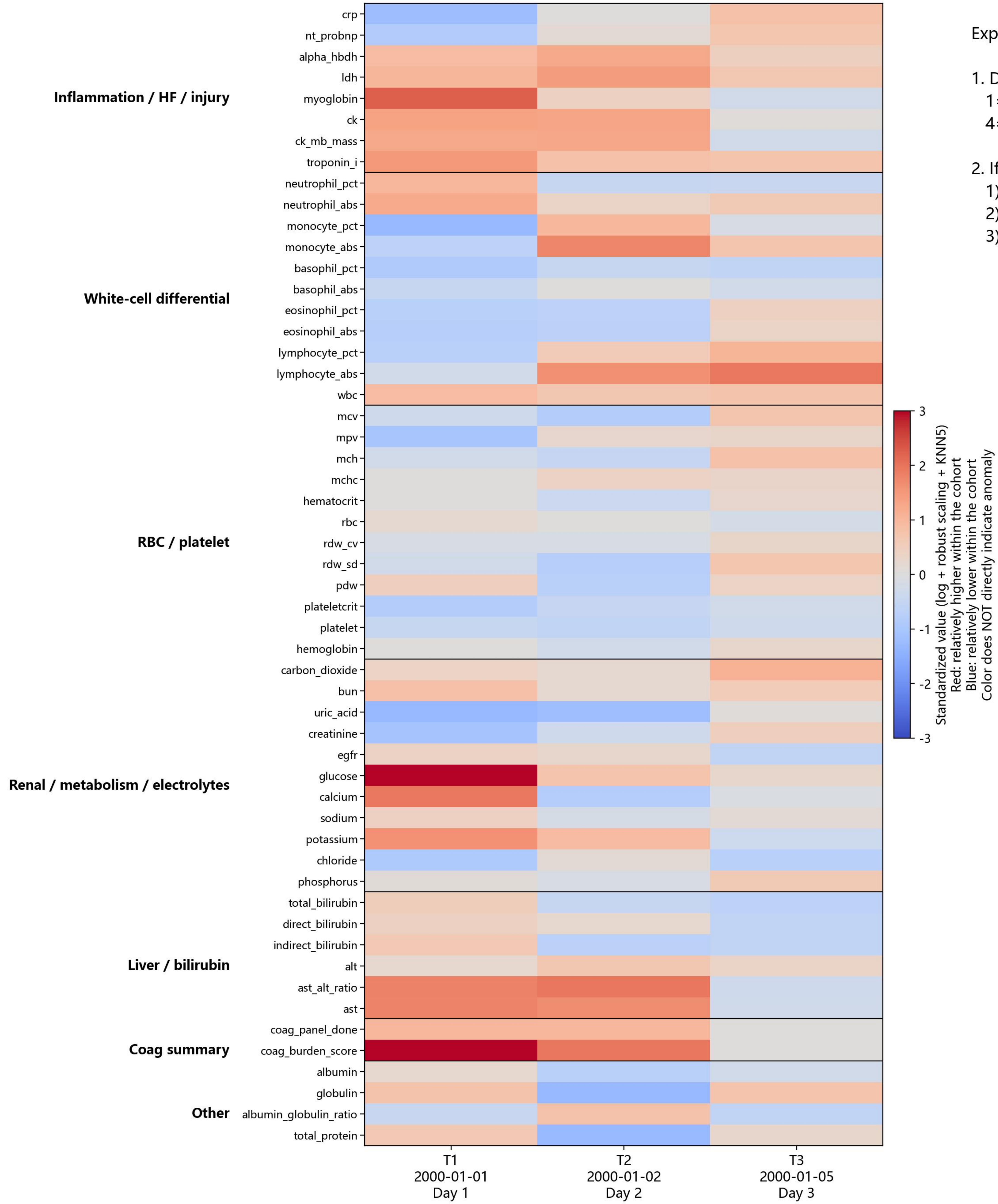

Expert review (blinded; no model score shown)

1. Degree of anomaly for this 3-point window (1-5):
   1=very typical; 2=relatively typical; 3=gray zone;
   4=relatively abnormal; 5=very abnormal

2. If scored 4-5, list the 3 most abnormal / noteworthy variables:
   1) ________________________
   2) ________________________
   3) ________________________

Patient-window heatmap card for blinded expert review
ID: P009 Window: W01

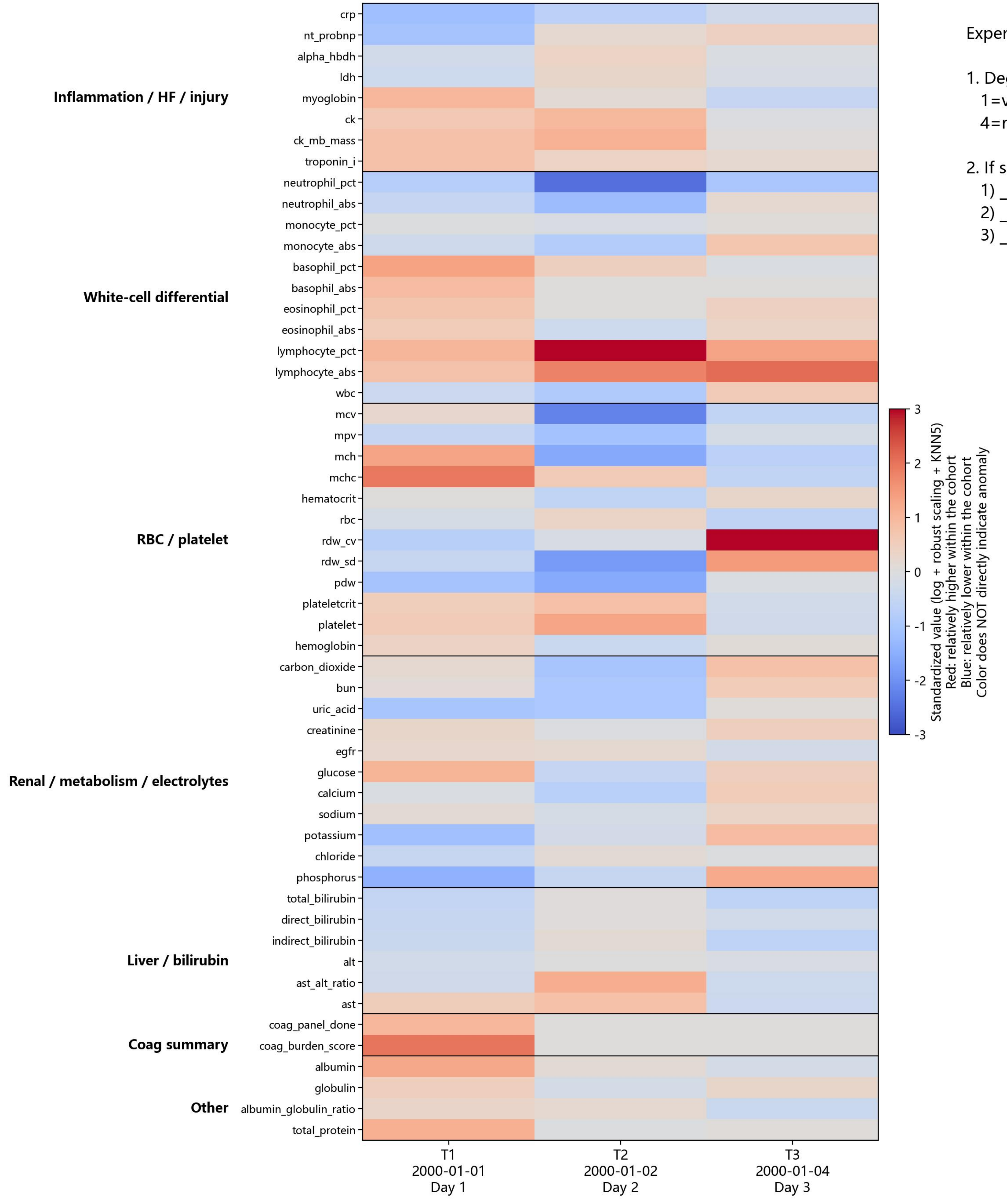

Expert review (blinded; no model score shown)

1. Degree of anomaly for this 3-point window (1-5):
   1=very typical; 2=relatively typical; 3=gray zone;
   4=relatively abnormal; 5=very abnormal

2. If scored 4-5, list the 3 most abnormal / noteworthy variables:
   1) ______________________
   2) ______________________
   3) ______________________

Patient-window heatmap card for blinded expert review
ID: P010   Window: W01

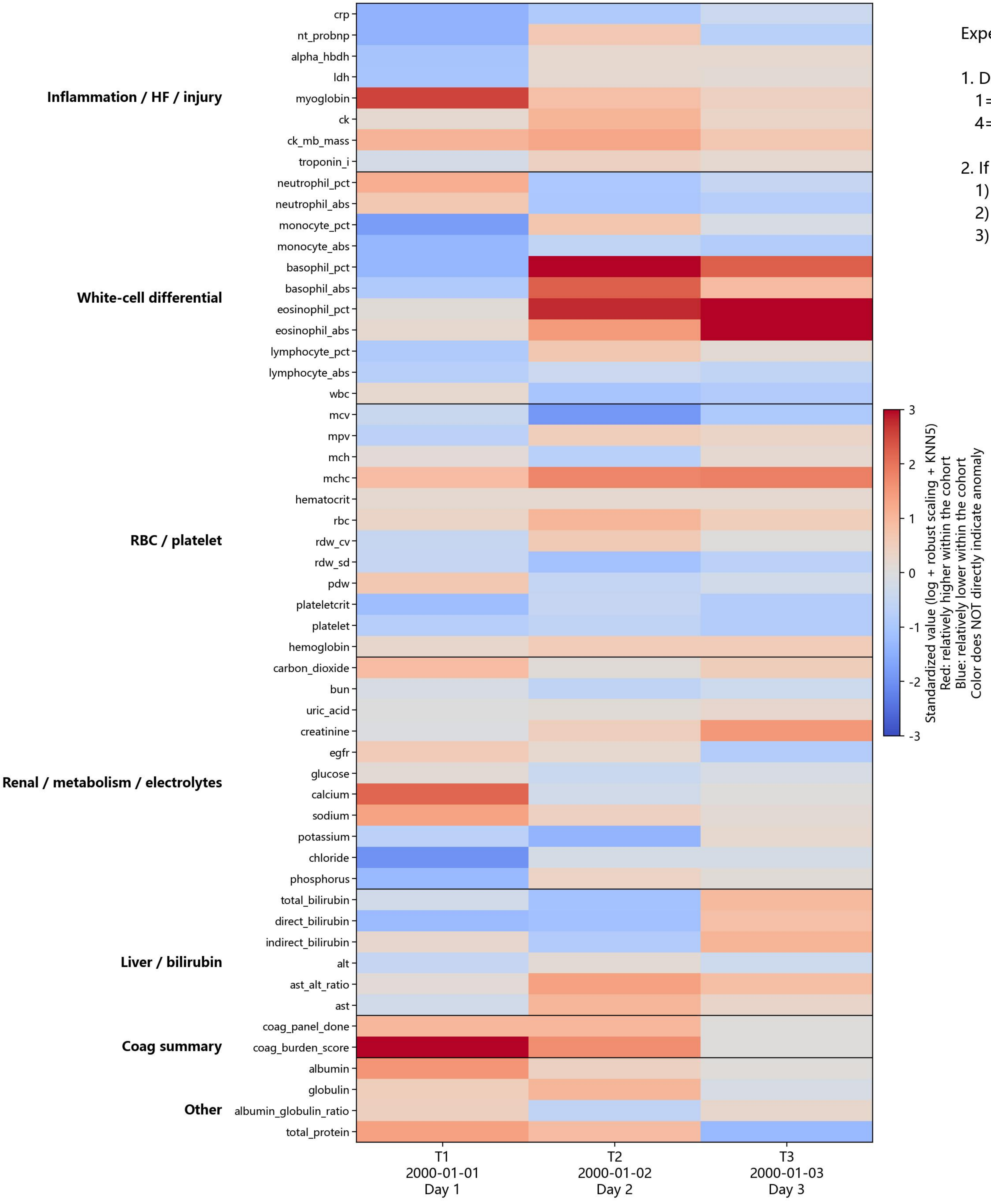

Expert review (blinded; no model score shown)

1. Degree of anomaly for this 3-point window (1-5):
   1=very typical; 2=relatively typical; 3=gray zone;
   4=relatively abnormal; 5=very abnormal

2. If scored 4-5, list the 3 most abnormal / noteworthy variables:
   1) ________________________
   2) ________________________
   3) ________________________

Patient-window heatmap card for blinded expert review
ID: P011   Window: W01

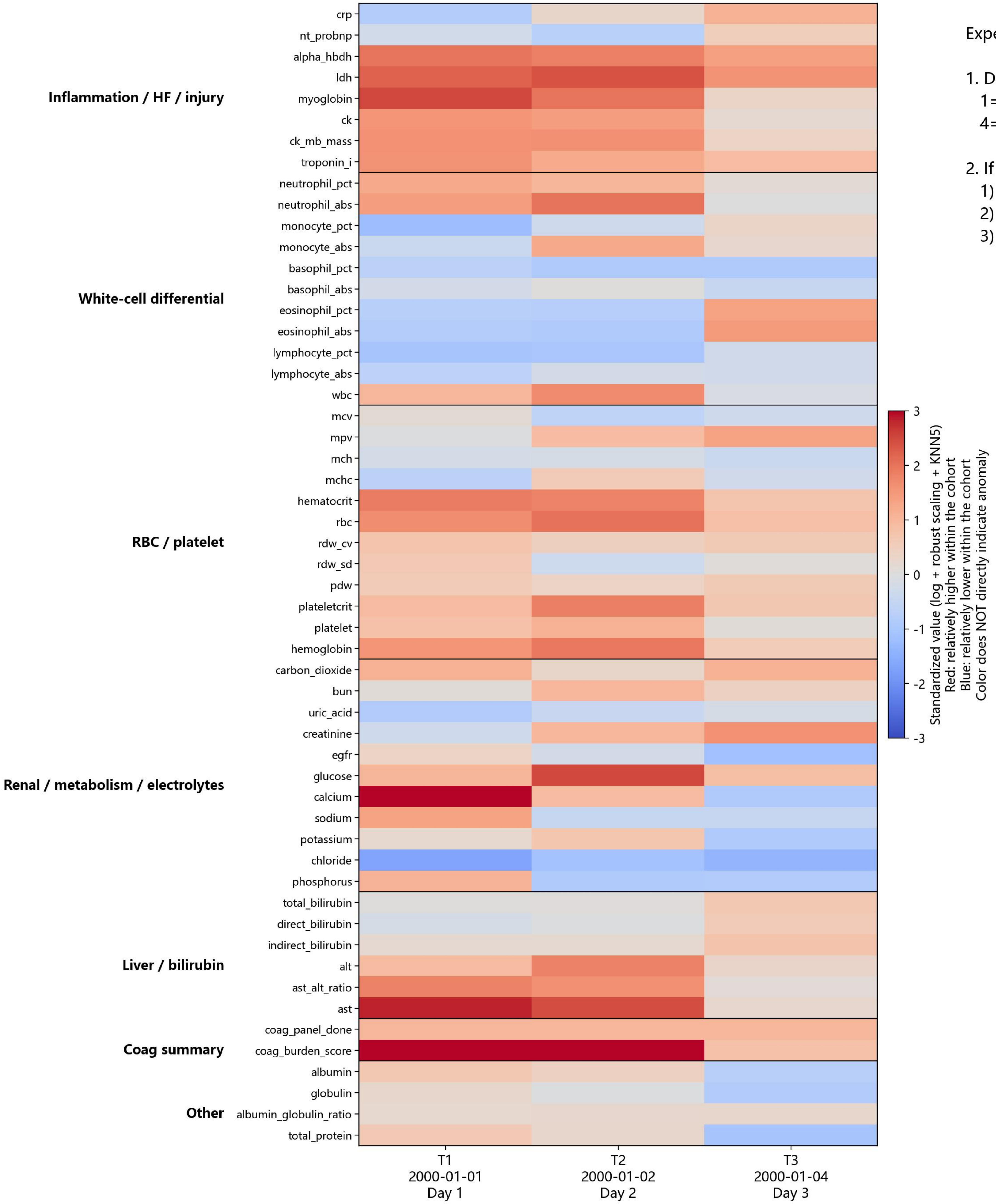

Expert review (blinded; no model score shown)

1. Degree of anomaly for this 3-point window (1-5):
   1=very typical; 2=relatively typical; 3=gray zone;
   4=relatively abnormal; 5=very abnormal

2. If scored 4-5, list the 3 most abnormal / noteworthy variables:
   1) ________________________
   2) ________________________
   3) ________________________

Patient-window heatmap card for blinded expert review
ID: P012   Window: W01

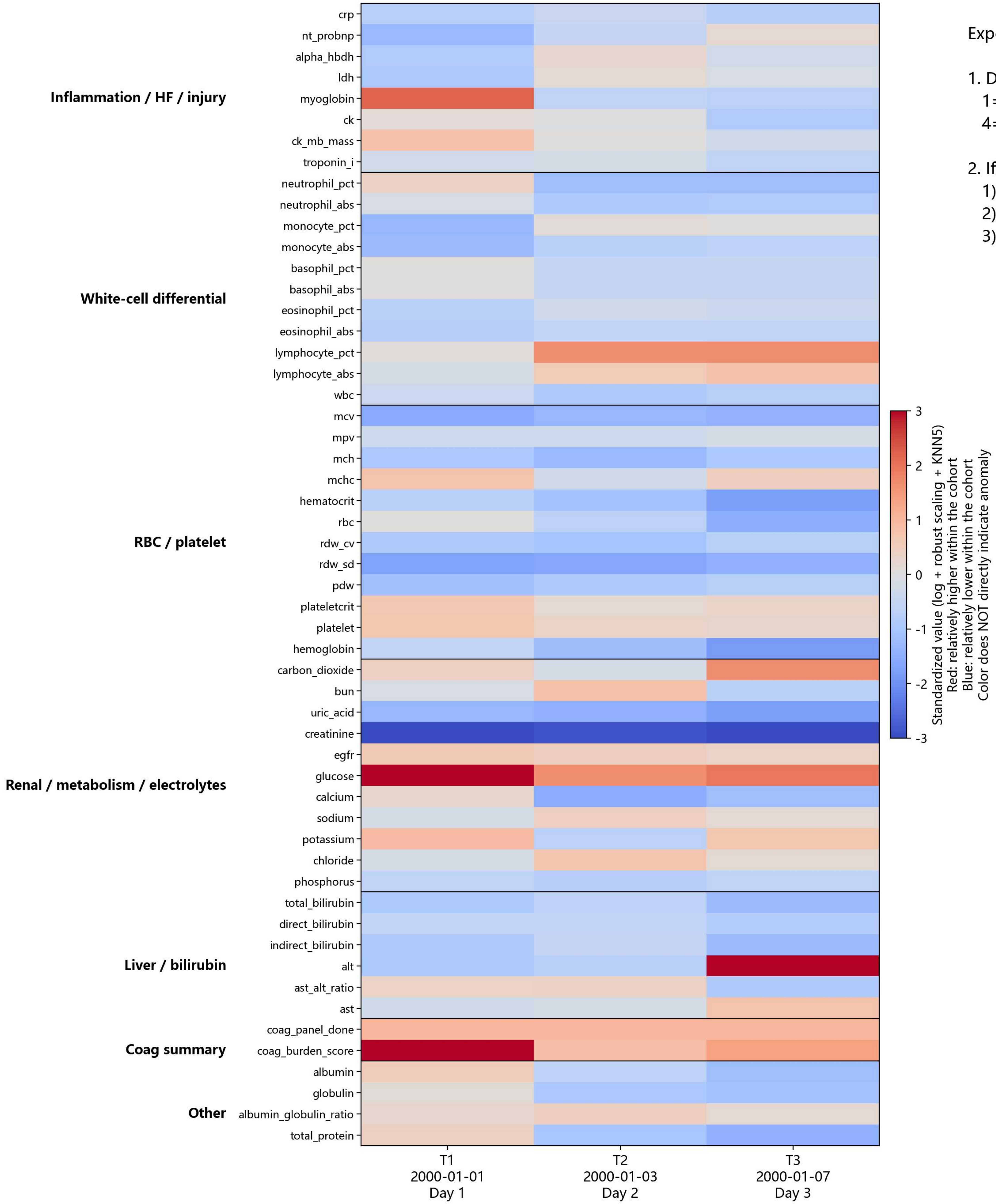

Expert review (blinded; no model score shown)

1. Degree of anomaly for this 3-point window (1-5):
   1=very typical; 2=relatively typical; 3=gray zone;
   4=relatively abnormal; 5=very abnormal

2. If scored 4-5, list the 3 most abnormal / noteworthy variables:
   1) ________________________
   2) ________________________
   3) ________________________

Patient-window heatmap card for blinded expert review
ID: P013   Window: W01

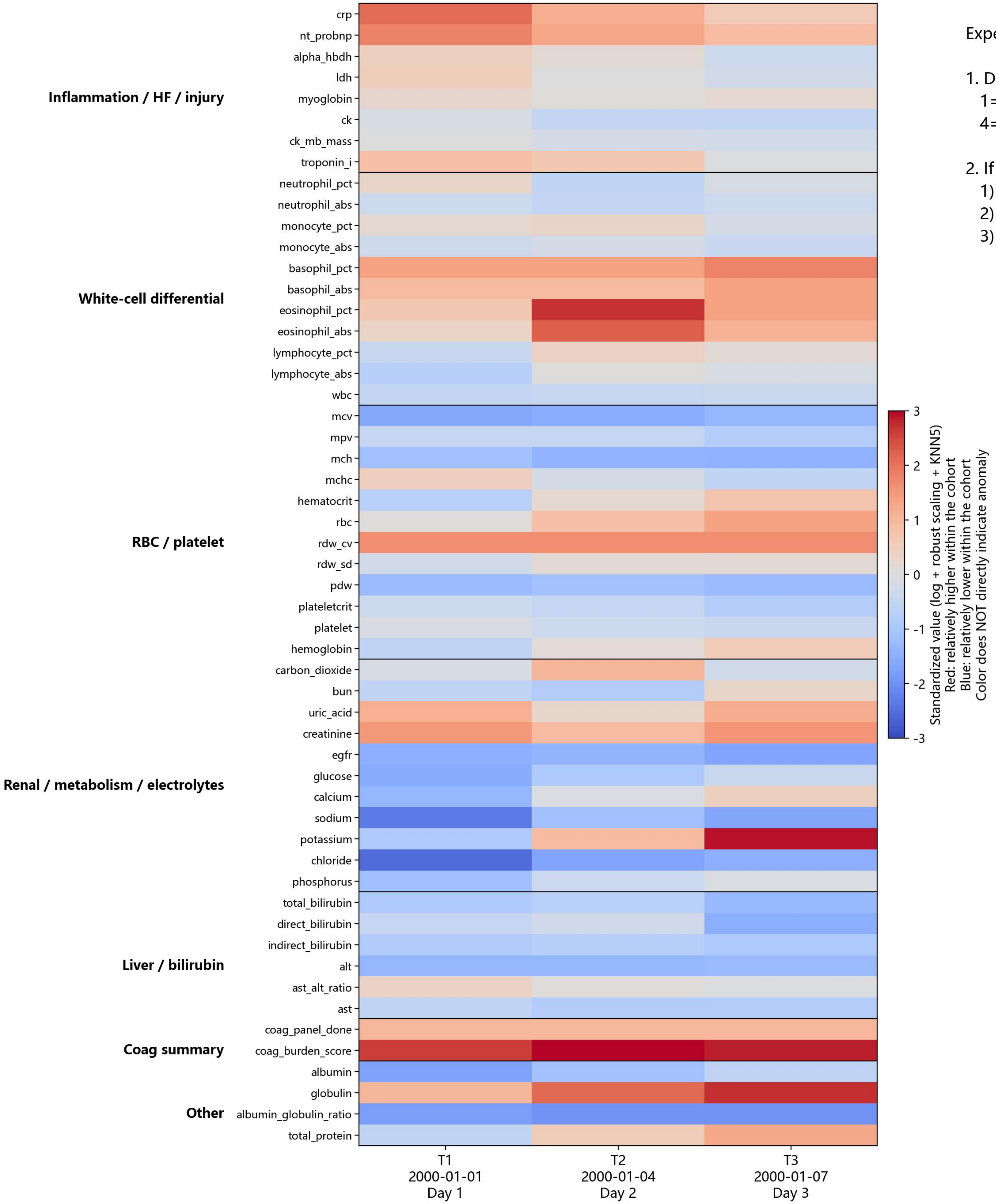

Expert review (blinded; no model score shown)

1. Degree of anomaly for this 3-point window (1-5):
   1=very typical; 2=relatively typical; 3=gray zone;
   4=relatively abnormal; 5=very abnormal

2. If scored 4-5, list the 3 most abnormal / noteworthy variables:
   1) ___________________________
   2) ___________________________
   3) ___________________________

Patient-window heatmap card for blinded expert review
ID: P014   Window: W01

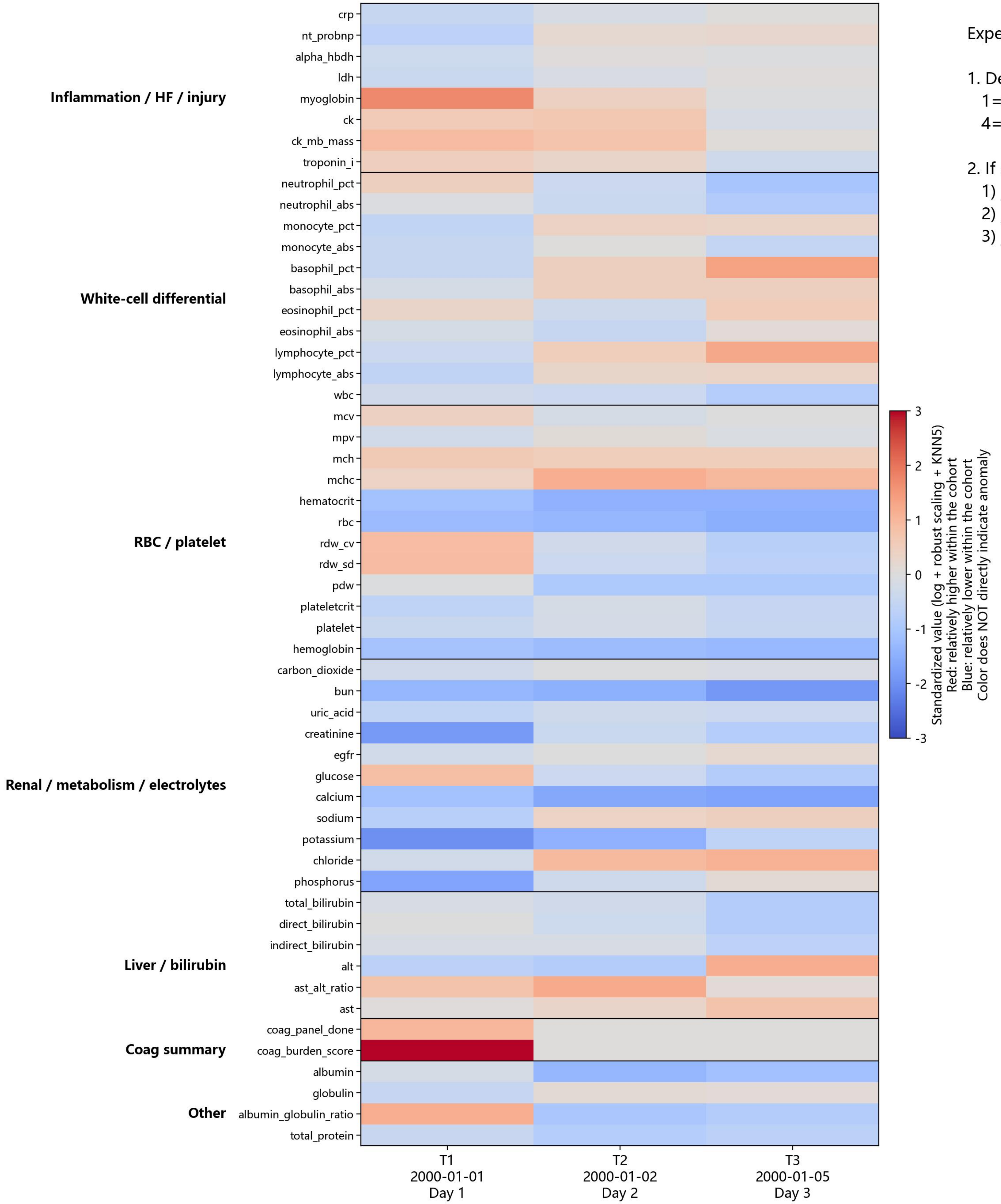

Expert review (blinded; no model score shown)

1. Degree of anomaly for this 3-point window (1-5):
   1=very typical; 2=relatively typical; 3=gray zone;
   4=relatively abnormal; 5=very abnormal

2. If scored 4-5, list the 3 most abnormal / noteworthy variables:
   1) ______________________
   2) ______________________
   3) ______________________

Patient-window heatmap card for blinded expert review
ID: P015 Window: W01

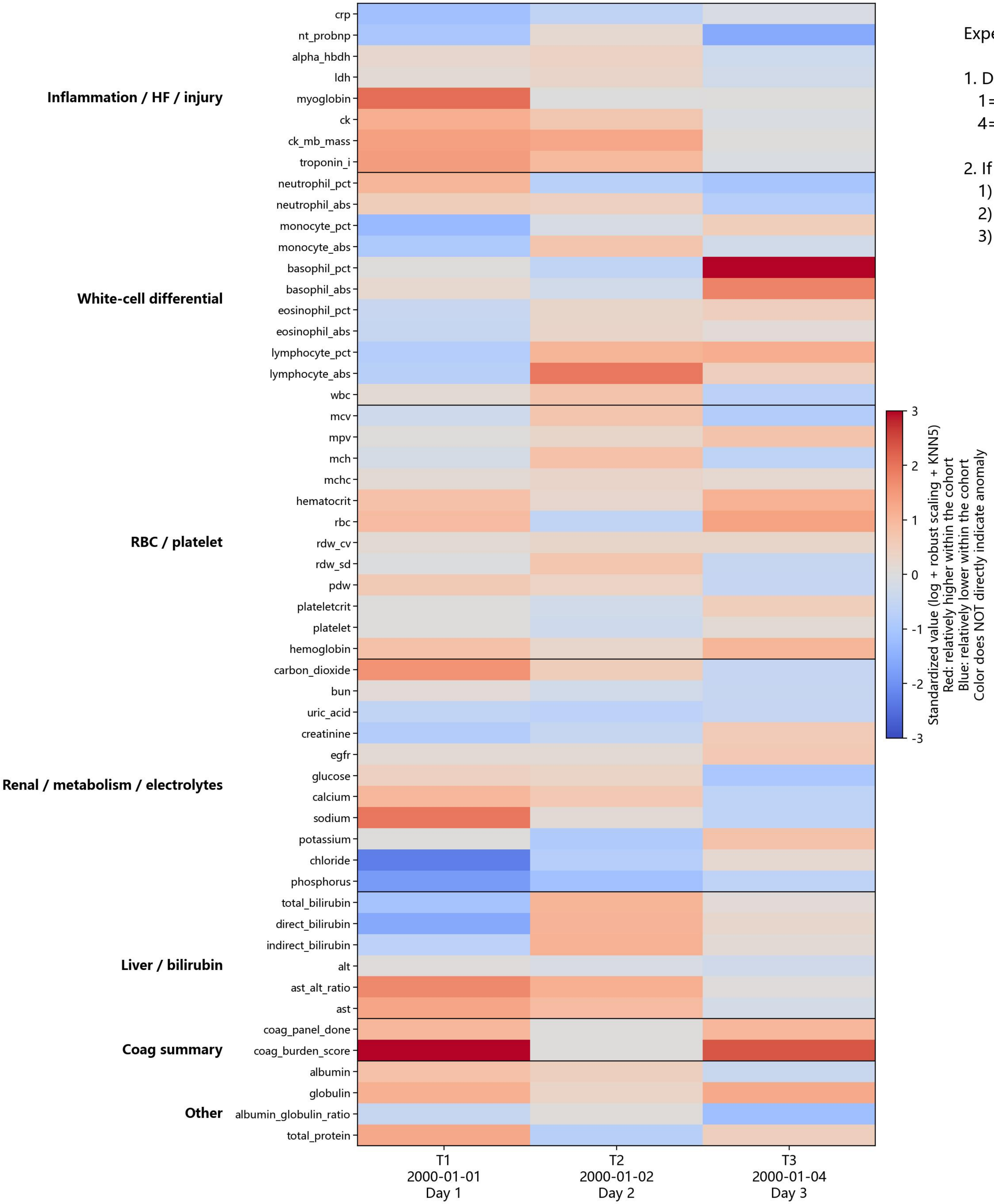

Expert review (blinded; no model score shown)

1. Degree of anomaly for this 3-point window (1-5):
1=very typical; 2=relatively typical; 3=gray zone;
4=relatively abnormal; 5=very abnormal

2. If scored 4-5, list the 3 most abnormal / noteworthy variables:
1) ________________________
2) ________________________
3) ________________________

Patient-window heatmap card for blinded expert review
ID: P016   Window: W01

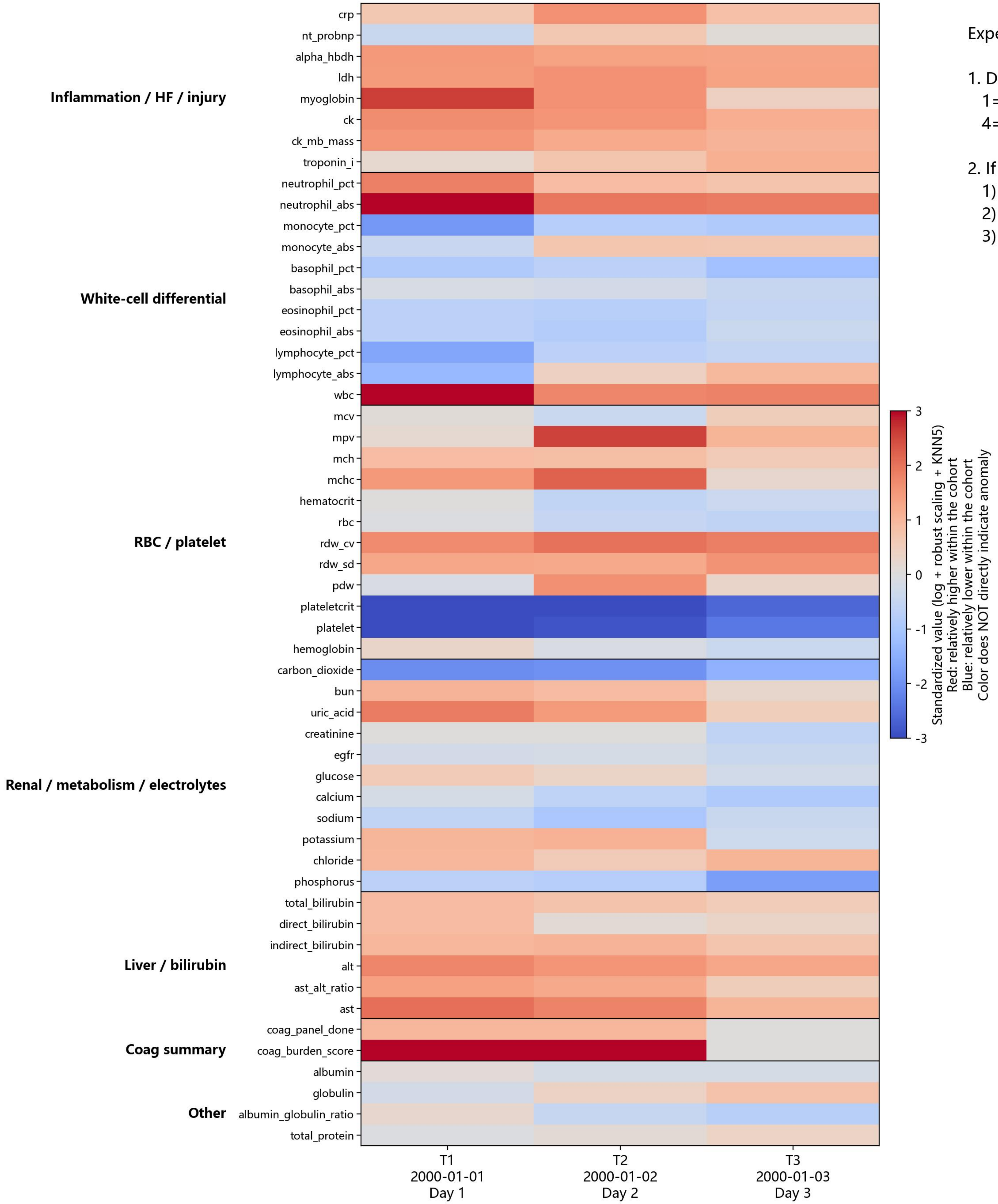

Expert review (blinded; no model score shown)

1. Degree of anomaly for this 3-point window (1-5):
   1=very typical; 2=relatively typical; 3=gray zone;
   4=relatively abnormal; 5=very abnormal

2. If scored 4-5, list the 3 most abnormal / noteworthy variables:
   1) ________________________
   2) ________________________
   3) ________________________

Patient-window heatmap card for blinded expert review
ID: P017   Window: W01

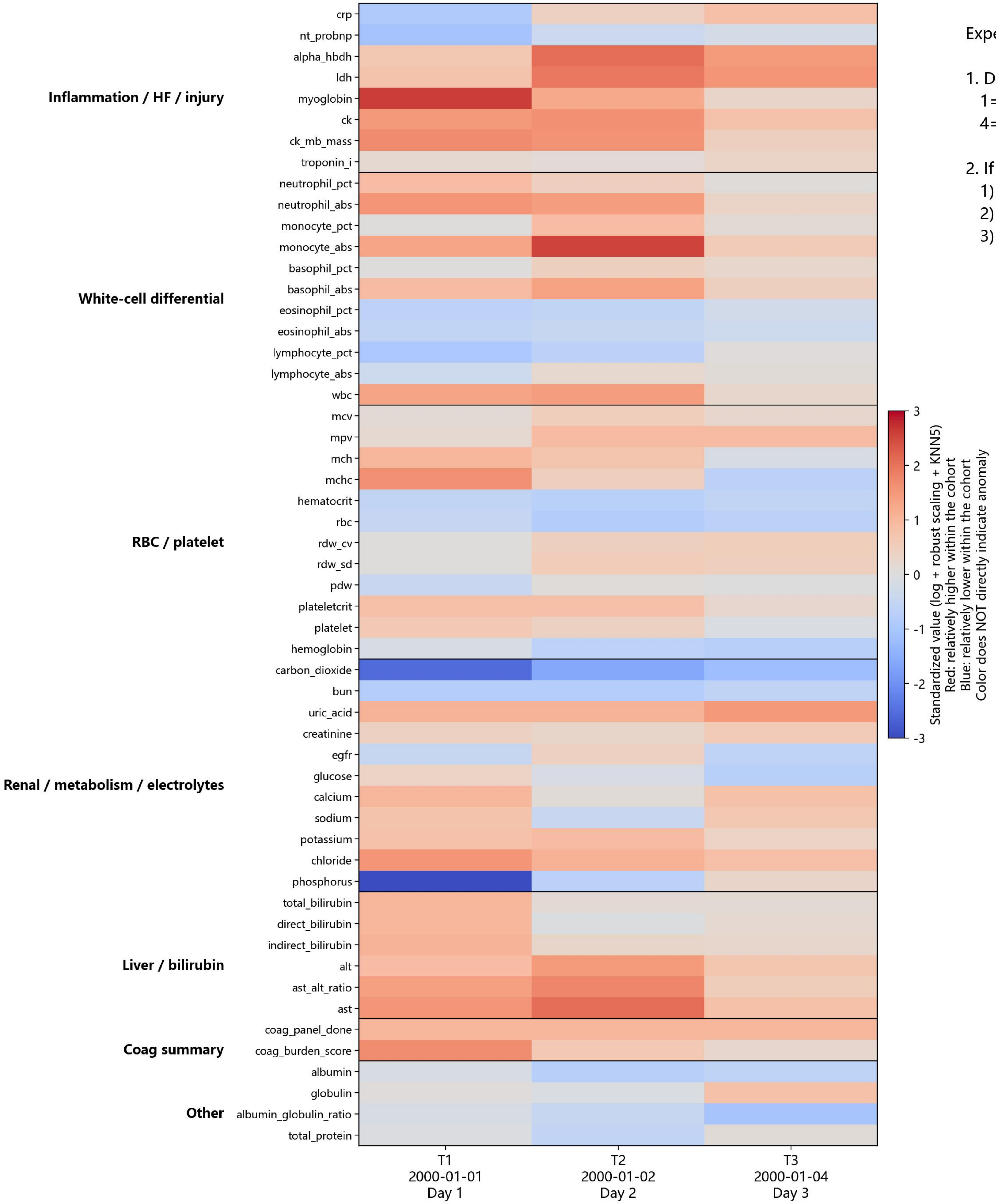

Expert review (blinded; no model score shown)

1. Degree of anomaly for this 3-point window (1-5):
   1=very typical; 2=relatively typical; 3=gray zone;
   4=relatively abnormal; 5=very abnormal

2. If scored 4-5, list the 3 most abnormal / noteworthy variables:
   1) ________________________
   2) ________________________
   3) ________________________

Patient-window heatmap card for blinded expert review
ID: P018 Window: W01

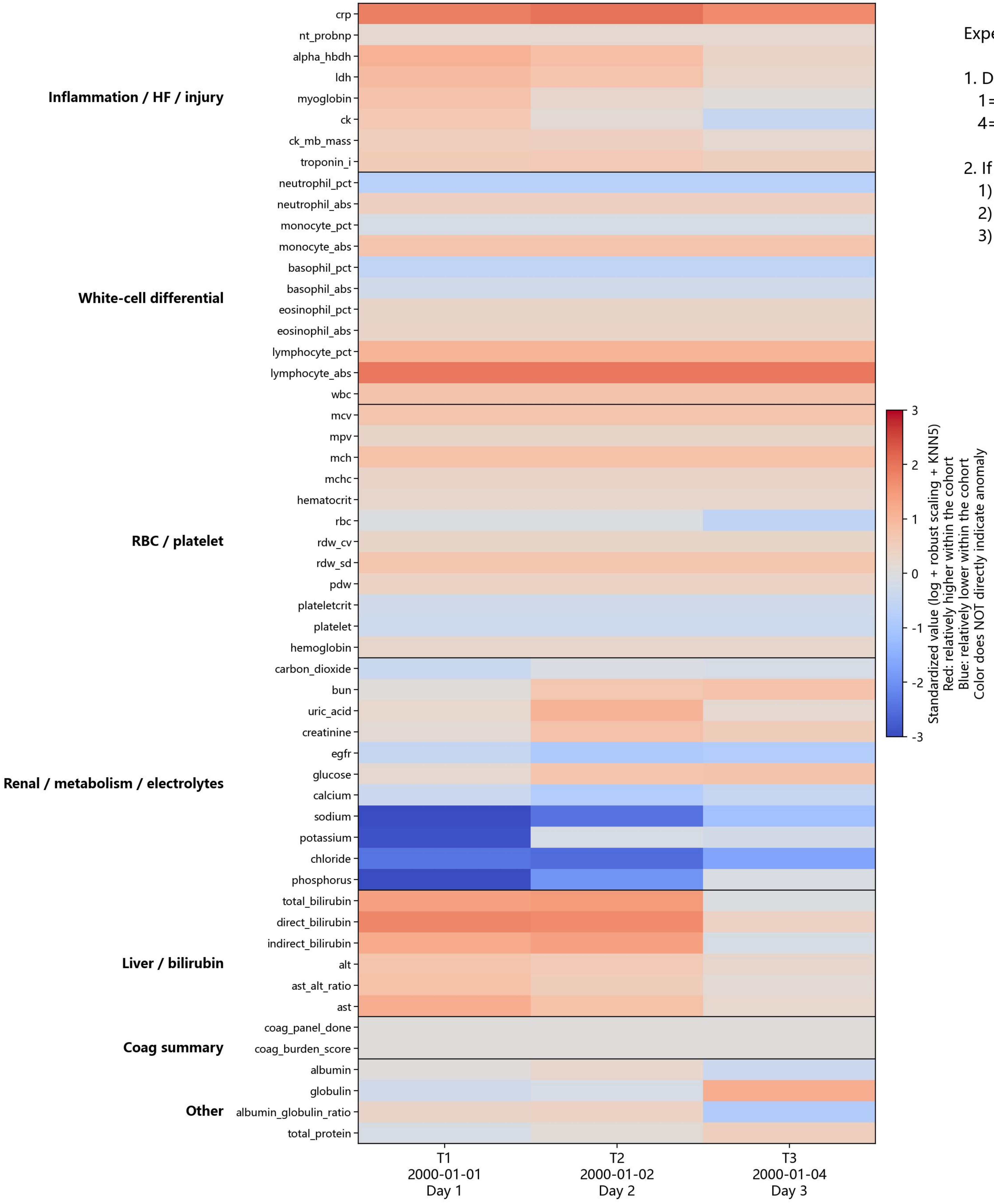

Expert review (blinded; no model score shown)

1. Degree of anomaly for this 3-point window (1-5):
1=very typical; 2=relatively typical; 3=gray zone;
4=relatively abnormal; 5=very abnormal

2. If scored 4-5, list the 3 most abnormal / noteworthy variables:
1) ____________________
2) ____________________
3) ____________________

Patient-window heatmap card for blinded expert review
ID: P019   Window: W01

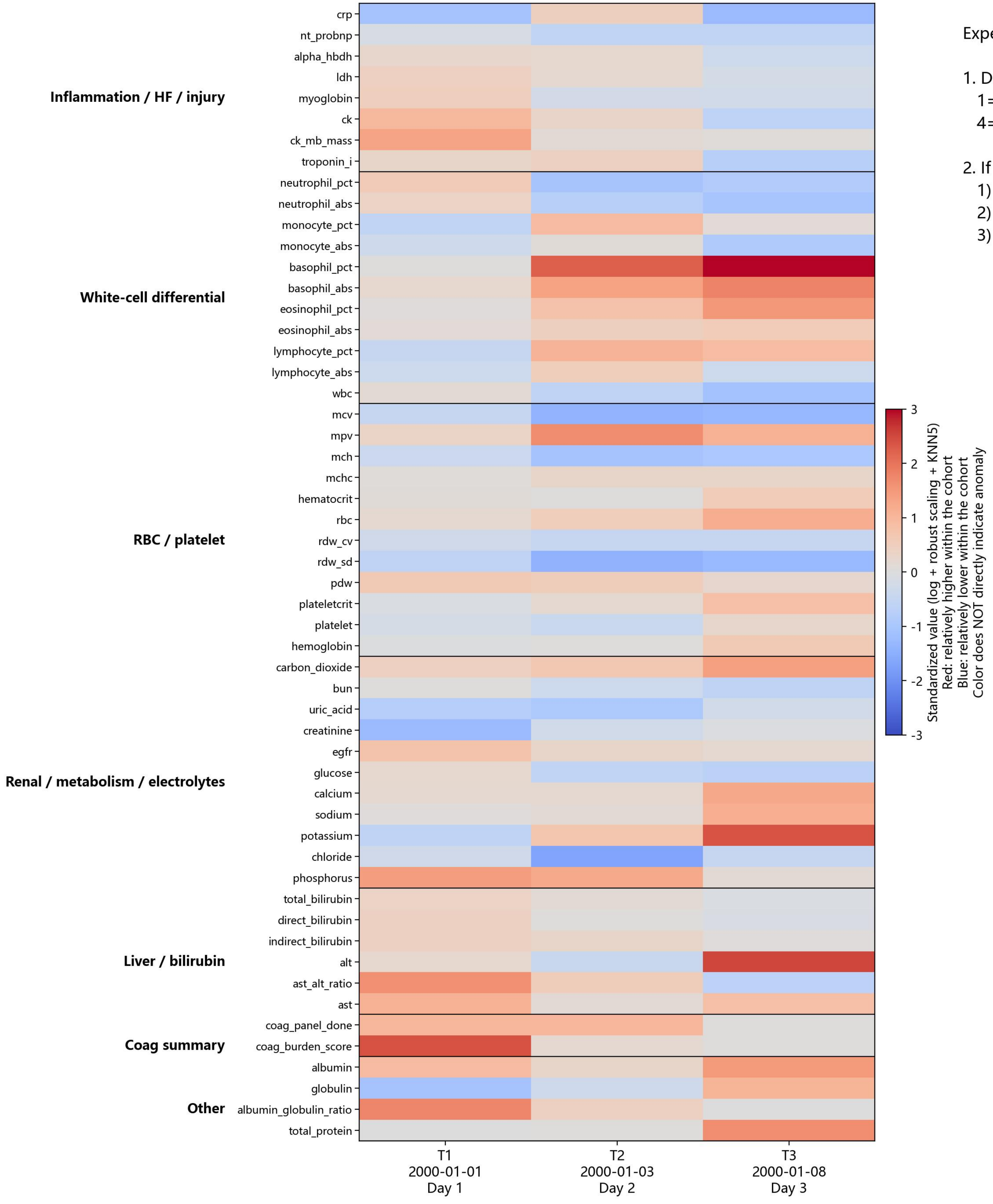

Expert review (blinded; no model score shown)

1. Degree of anomaly for this 3-point window (1-5):
   1=very typical; 2=relatively typical; 3=gray zone;
   4=relatively abnormal; 5=very abnormal

2. If scored 4-5, list the 3 most abnormal / noteworthy variables:
   1) ________________________
   2) ________________________
   3) ________________________

Patient-window heatmap card for blinded expert review
ID: P020 Window: W01

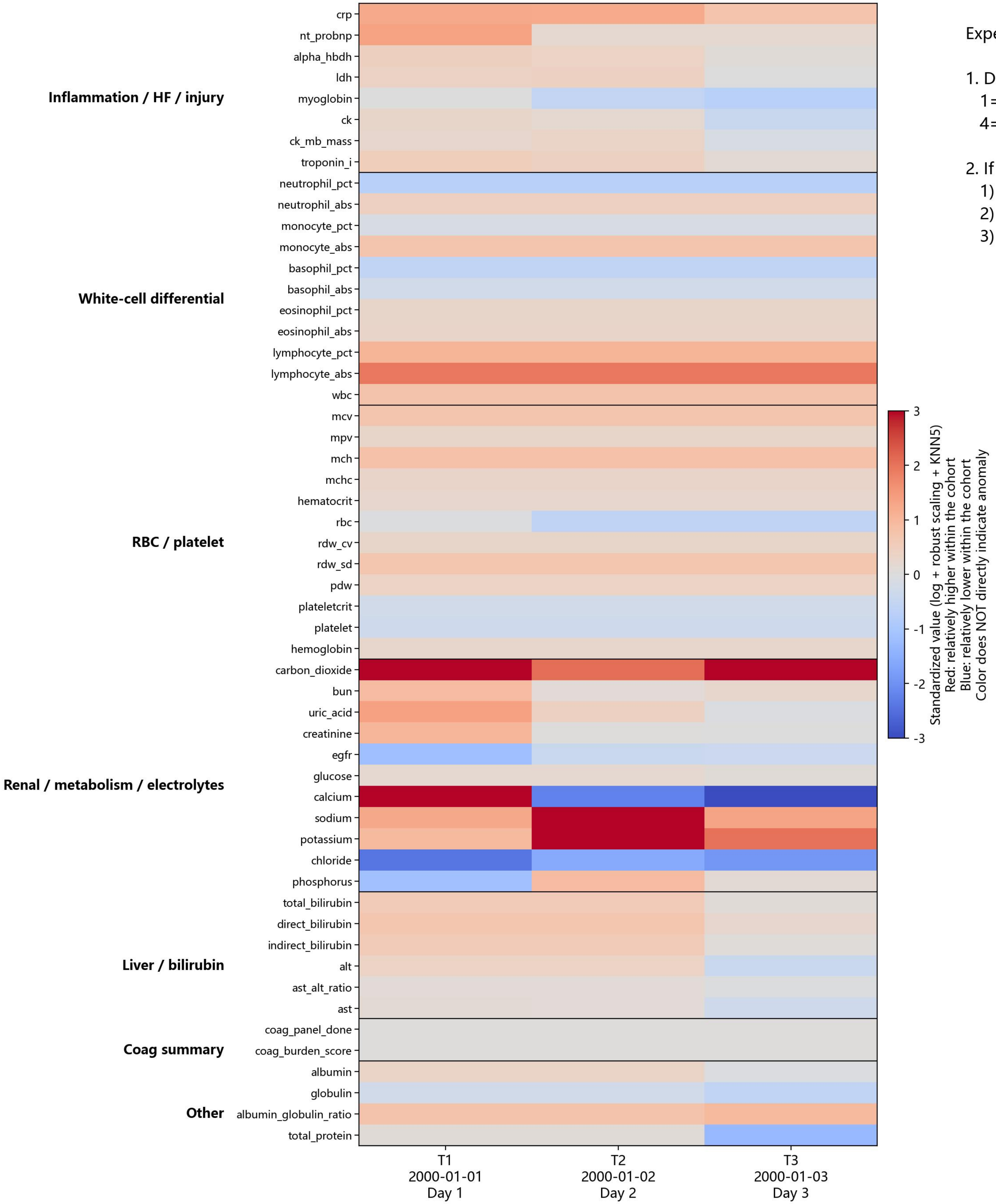

Expert review (blinded; no model score shown)

1. Degree of anomaly for this 3-point window (1-5):
   1=very typical; 2=relatively typical; 3=gray zone;
   4=relatively abnormal; 5=very abnormal

2. If scored 4-5, list the 3 most abnormal / noteworthy variables:
   1) ________________________
   2) ________________________
   3) ________________________

Patient-window heatmap card for blinded expert review
ID: P021 Window: W01

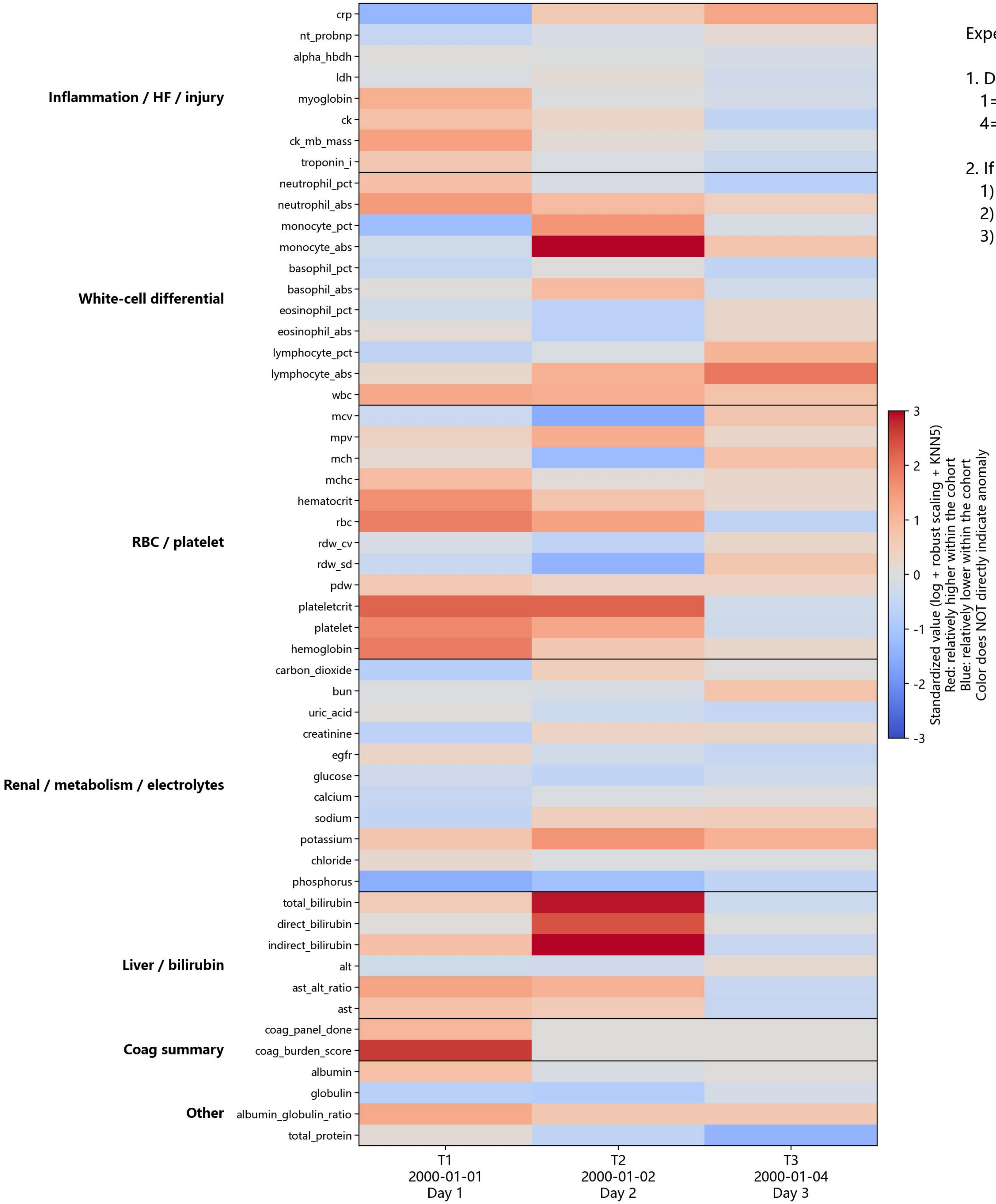

Expert review (blinded; no model score shown)

1. Degree of anomaly for this 3-point window (1-5):
   1=very typical; 2=relatively typical; 3=gray zone;
   4=relatively abnormal; 5=very abnormal

2. If scored 4-5, list the 3 most abnormal / noteworthy variables:
   1) ________________________
   2) ________________________
   3) ________________________

Patient-window heatmap card for blinded expert review
ID: P022   Window: W01

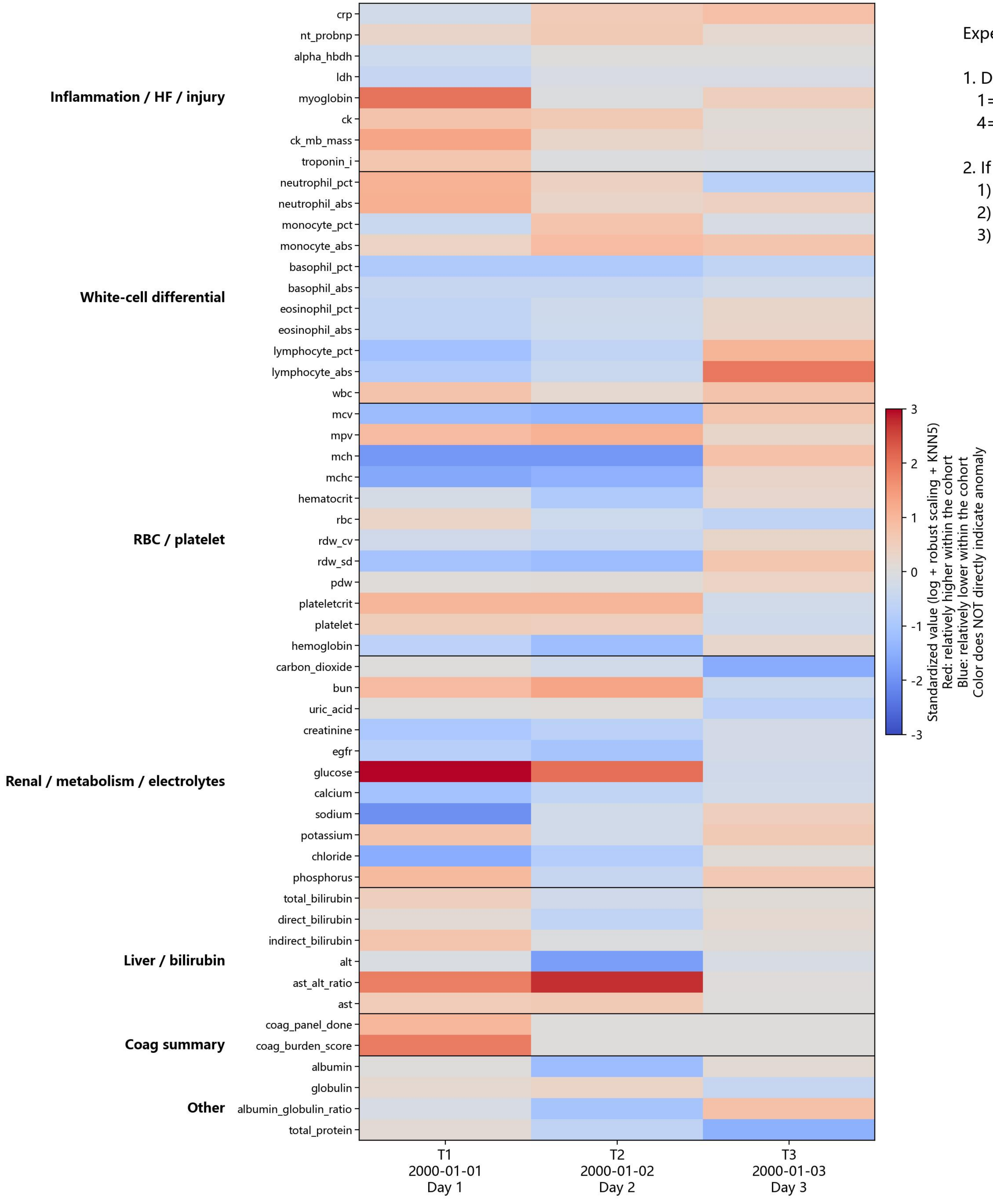

Expert review (blinded; no model score shown)

1. Degree of anomaly for this 3-point window (1-5):
   1=very typical; 2=relatively typical; 3=gray zone;
   4=relatively abnormal; 5=very abnormal

2. If scored 4-5, list the 3 most abnormal / noteworthy variables:
   1) ________________________
   2) ________________________
   3) ________________________

Patient-window heatmap card for blinded expert review
ID: P023   Window: W01

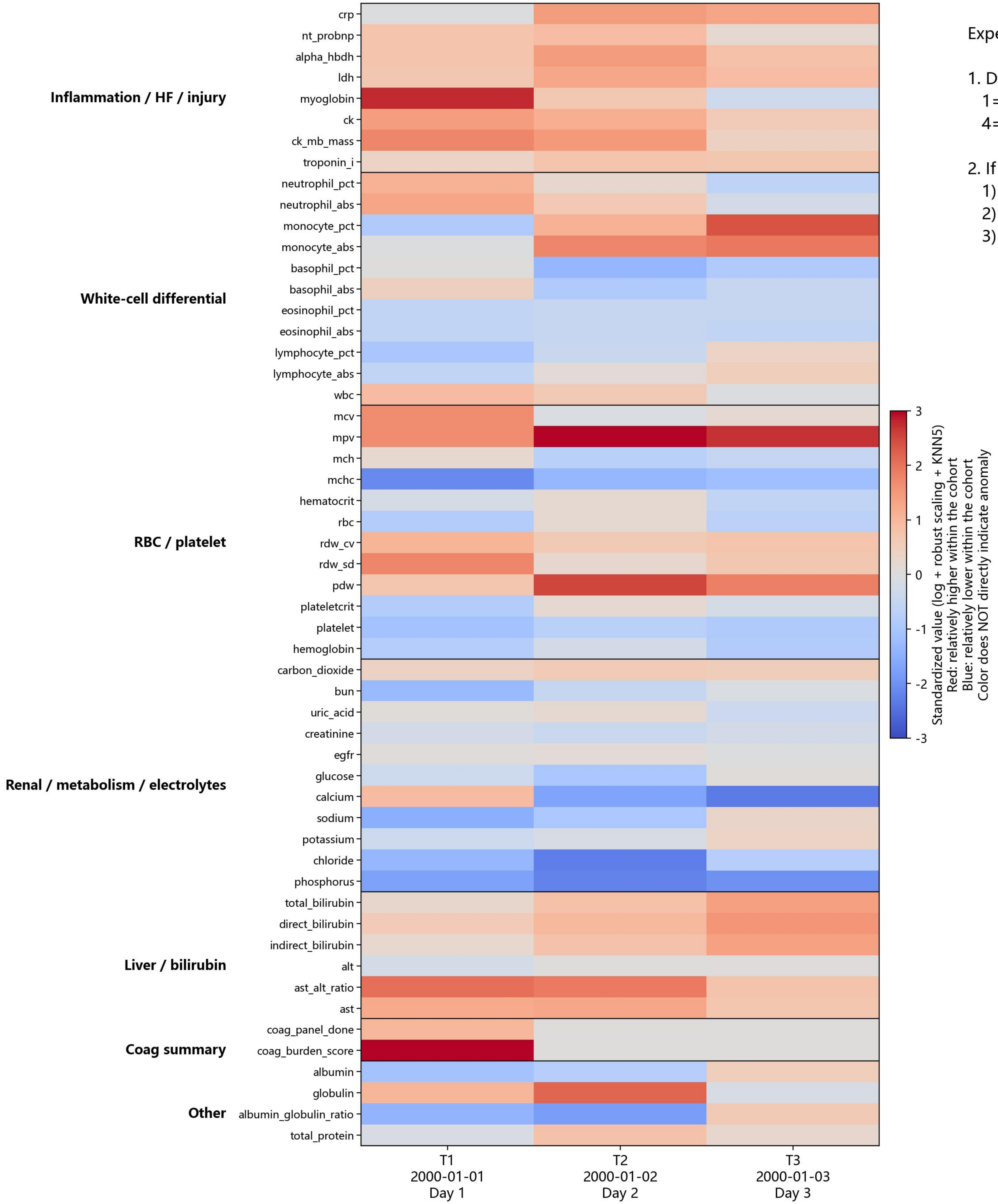

Expert review (blinded; no model score shown)

1. Degree of anomaly for this 3-point window (1-5):
   1=very typical; 2=relatively typical; 3=gray zone;
   4=relatively abnormal; 5=very abnormal

2. If scored 4-5, list the 3 most abnormal / noteworthy variables:
   1) ______________________
   2) ______________________
   3) ______________________

Patient-window heatmap card for blinded expert review
ID: P024   Window: W01

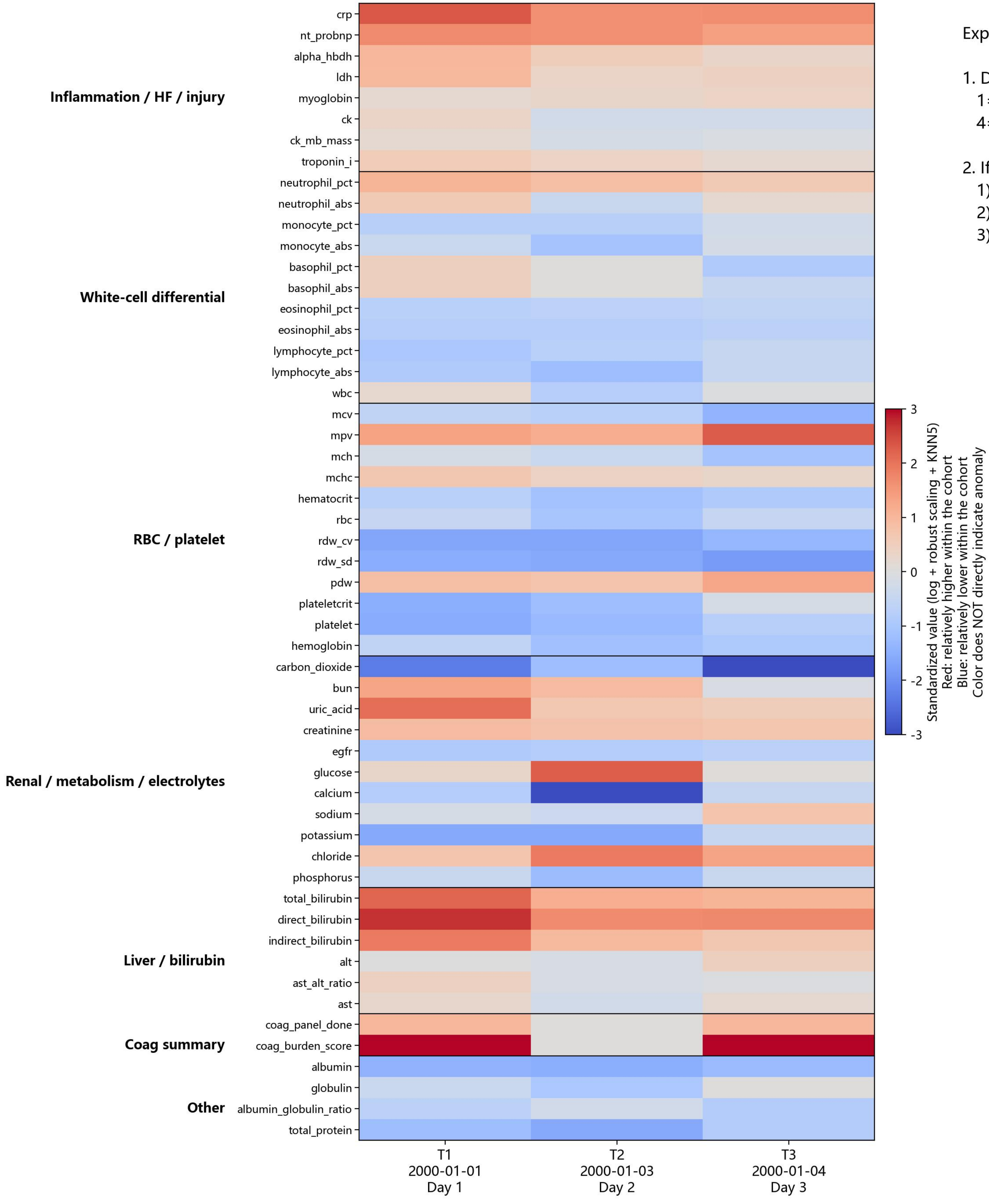

Expert review (blinded; no model score shown)

1. Degree of anomaly for this 3-point window (1-5):
   1=very typical; 2=relatively typical; 3=gray zone;
   4=relatively abnormal; 5=very abnormal

2. If scored 4-5, list the 3 most abnormal / noteworthy variables:
   1) ________________________
   2) ________________________
   3) ________________________

Patient-window heatmap card for blinded expert review
ID: P025   Window: W01

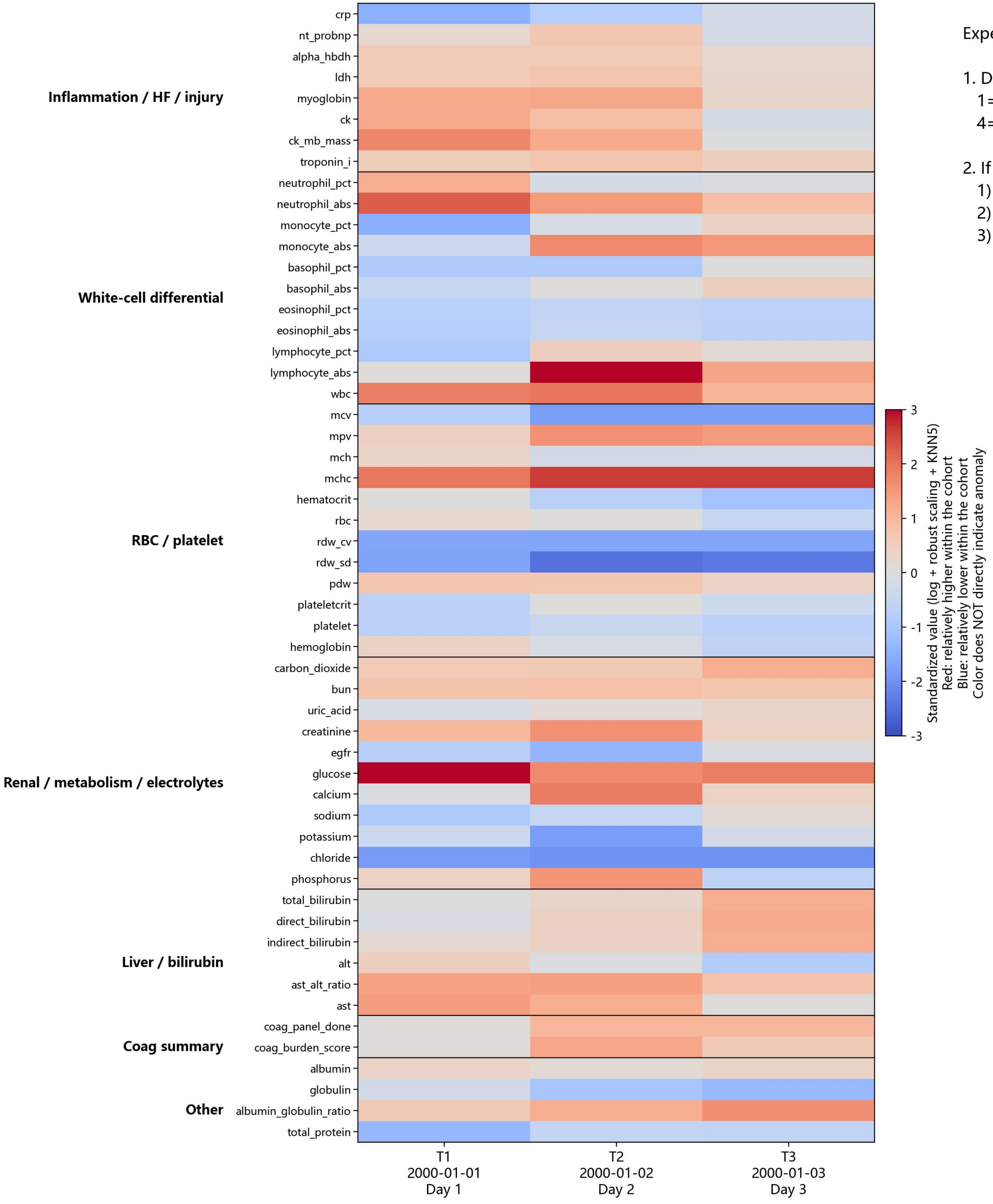

Expert review (blinded; no model score shown)

1. Degree of anomaly for this 3-point window (1-5):
   1=very typical; 2=relatively typical; 3=gray zone;
   4=relatively abnormal; 5=very abnormal

2. If scored 4-5, list the 3 most abnormal / noteworthy variables:
   1) ________________________
   2) ________________________
   3) ________________________

Patient-window heatmap card for blinded expert review
ID: P026   Window: W01

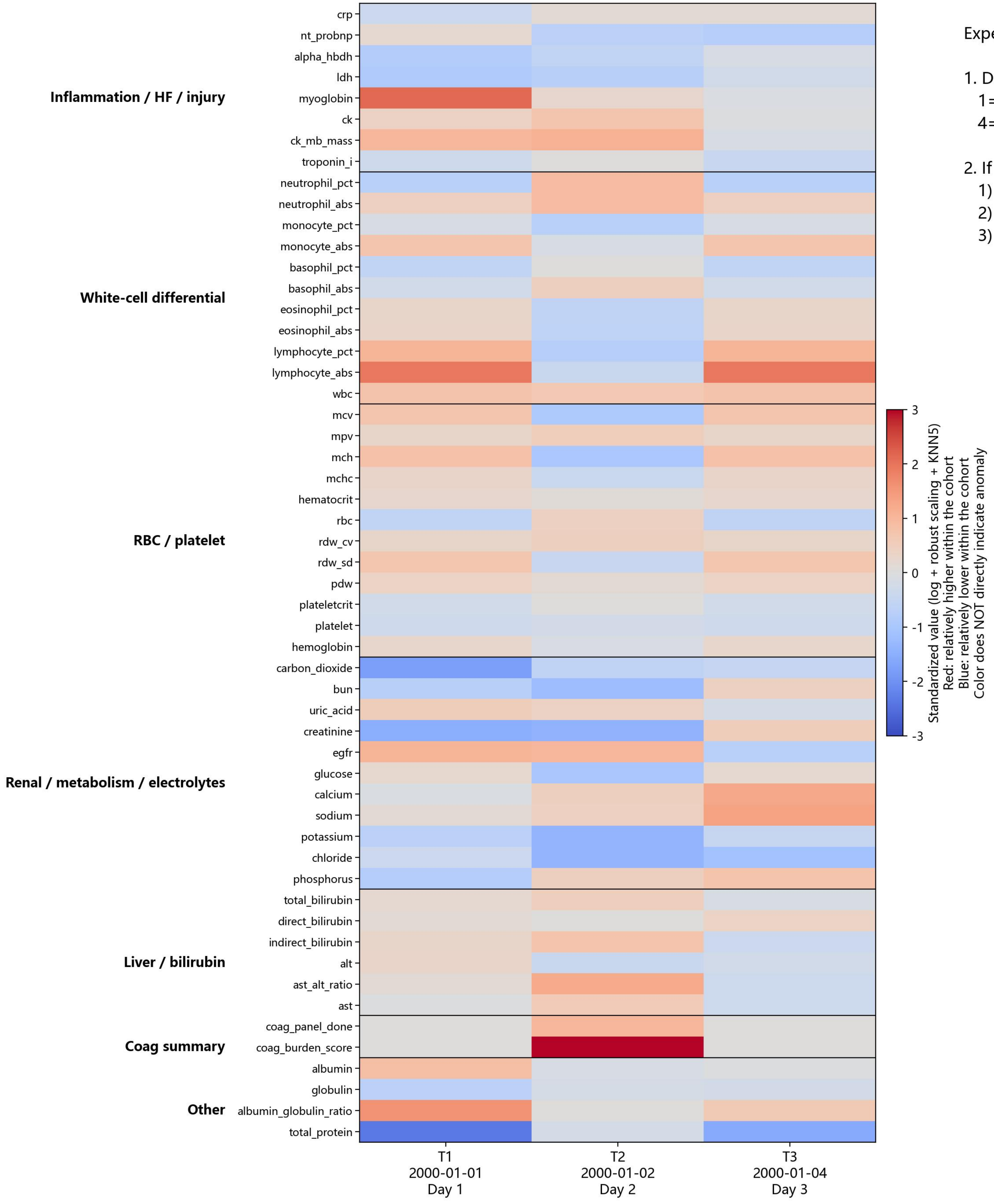

Expert review (blinded; no model score shown)

1. Degree of anomaly for this 3-point window (1-5):
   1=very typical; 2=relatively typical; 3=gray zone;
   4=relatively abnormal; 5=very abnormal

2. If scored 4-5, list the 3 most abnormal / noteworthy variables:
   1) ___________________________
   2) ___________________________
   3) ___________________________

Patient-window heatmap card for blinded expert review
ID: P027   Window: W01

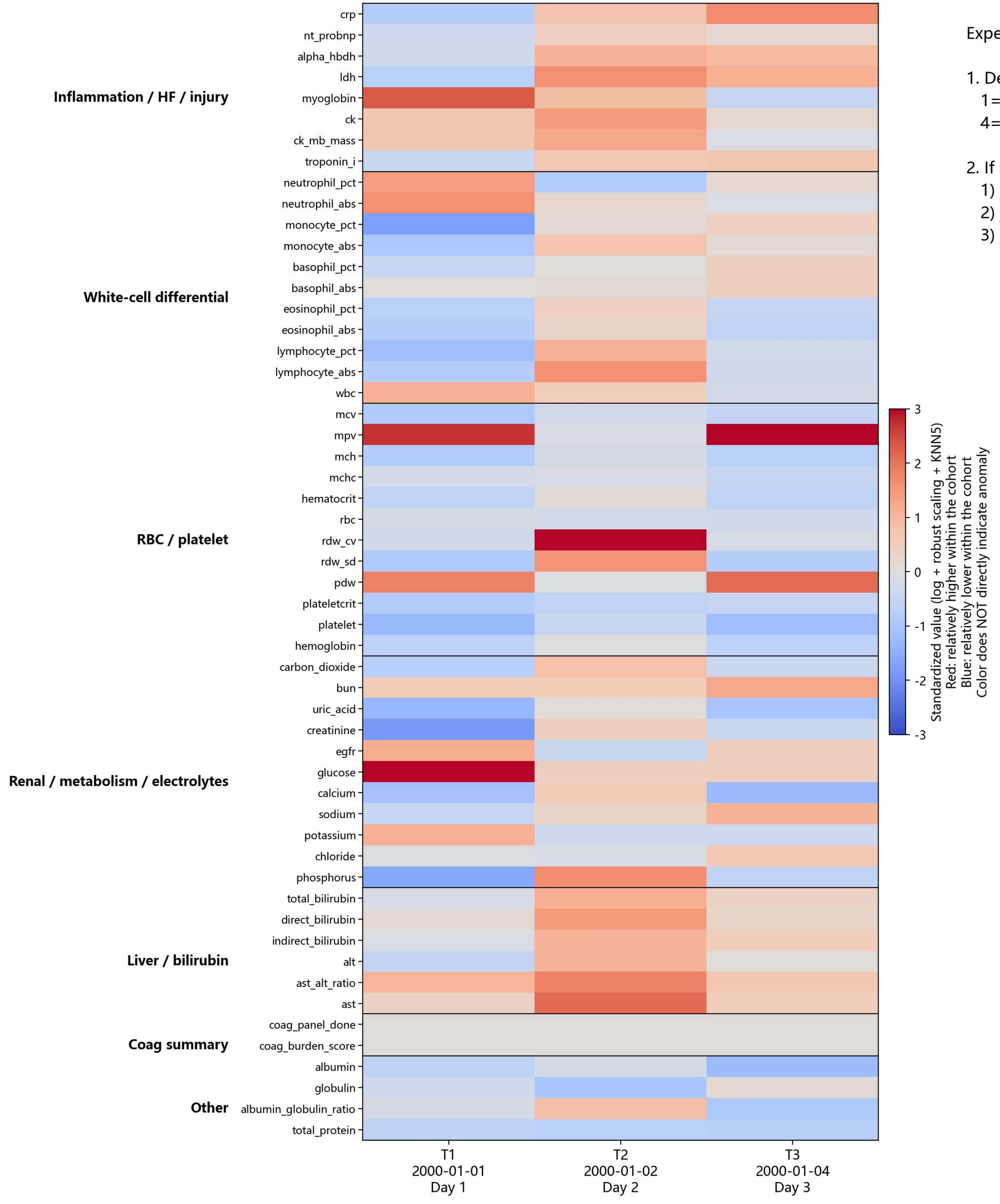

Expert review (blinded; no model score shown)

1. Degree of anomaly for this 3-point window (1-5):
   1=very typical; 2=relatively typical; 3=gray zone;
   4=relatively abnormal; 5=very abnormal

2. If scored 4-5, list the 3 most abnormal / noteworthy variables:
   1) ____________________
   2) ____________________
   3) ____________________

Patient-window heatmap card for blinded expert review
ID: P028   Window: W01

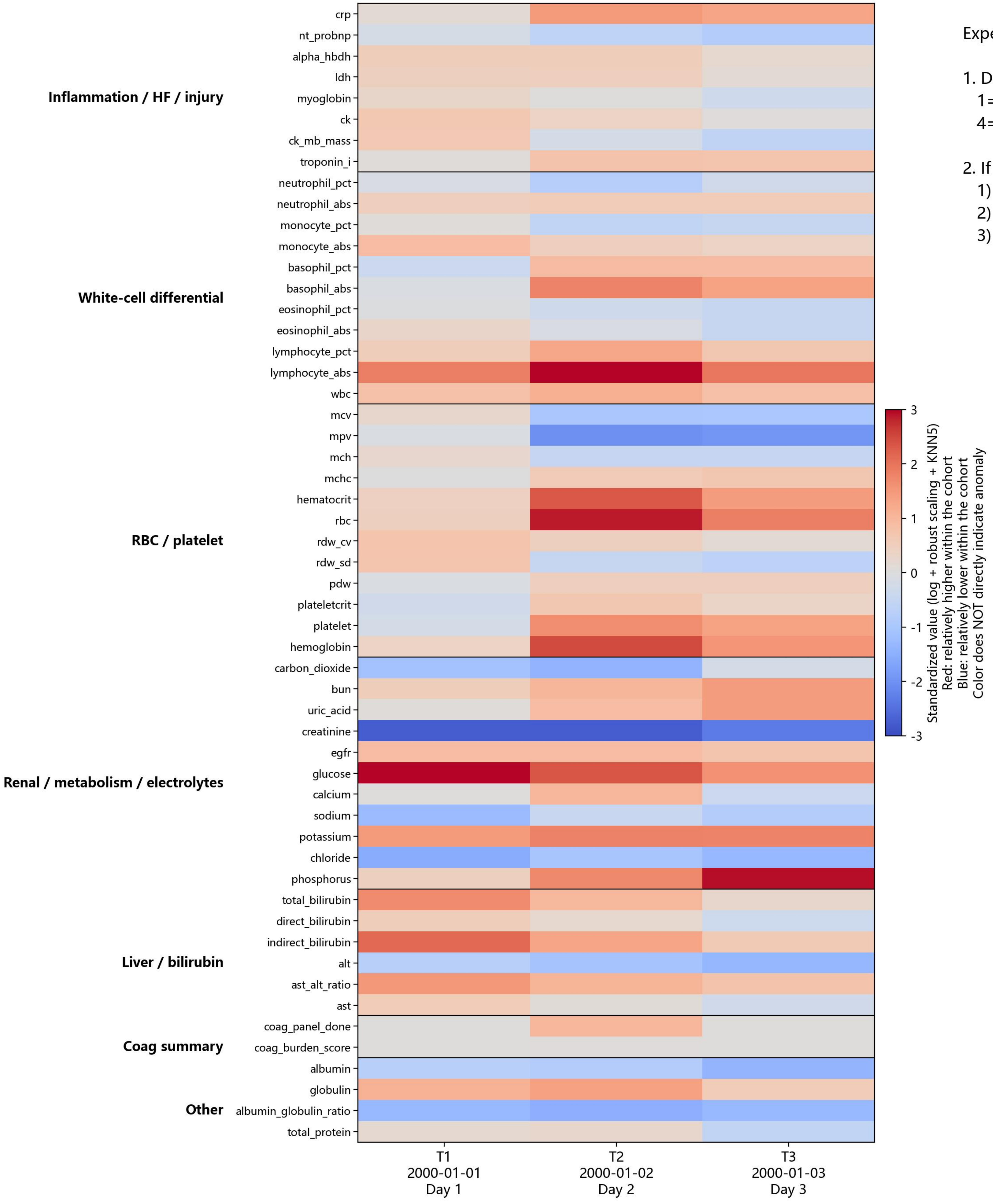

Expert review (blinded; no model score shown)

1. Degree of anomaly for this 3-point window (1-5):
   1=very typical; 2=relatively typical; 3=gray zone;
   4=relatively abnormal; 5=very abnormal

2. If scored 4-5, list the 3 most abnormal / noteworthy variables:
   1) ____________________
   2) ____________________
   3) ____________________

Patient-window heatmap card for blinded expert review
ID: P029   Window: W01

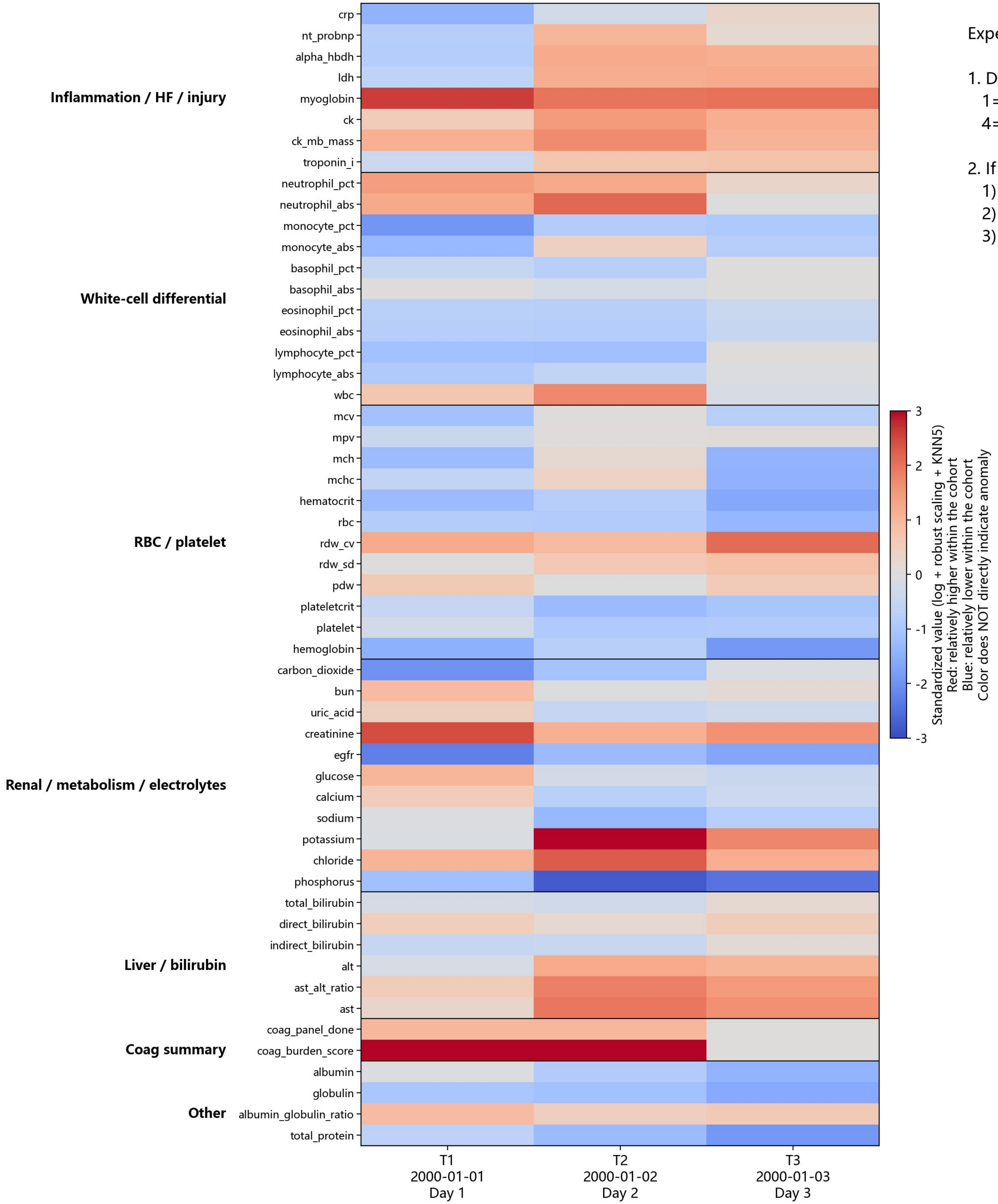

Expert review (blinded; no model score shown)

1. Degree of anomaly for this 3-point window (1-5):
   1=very typical; 2=relatively typical; 3=gray zone;
   4=relatively abnormal; 5=very abnormal

2. If scored 4-5, list the 3 most abnormal / noteworthy variables:
   1) ________________________
   2) ________________________
   3) ________________________

Patient-window heatmap card for blinded expert review
ID: P030   Window: W01

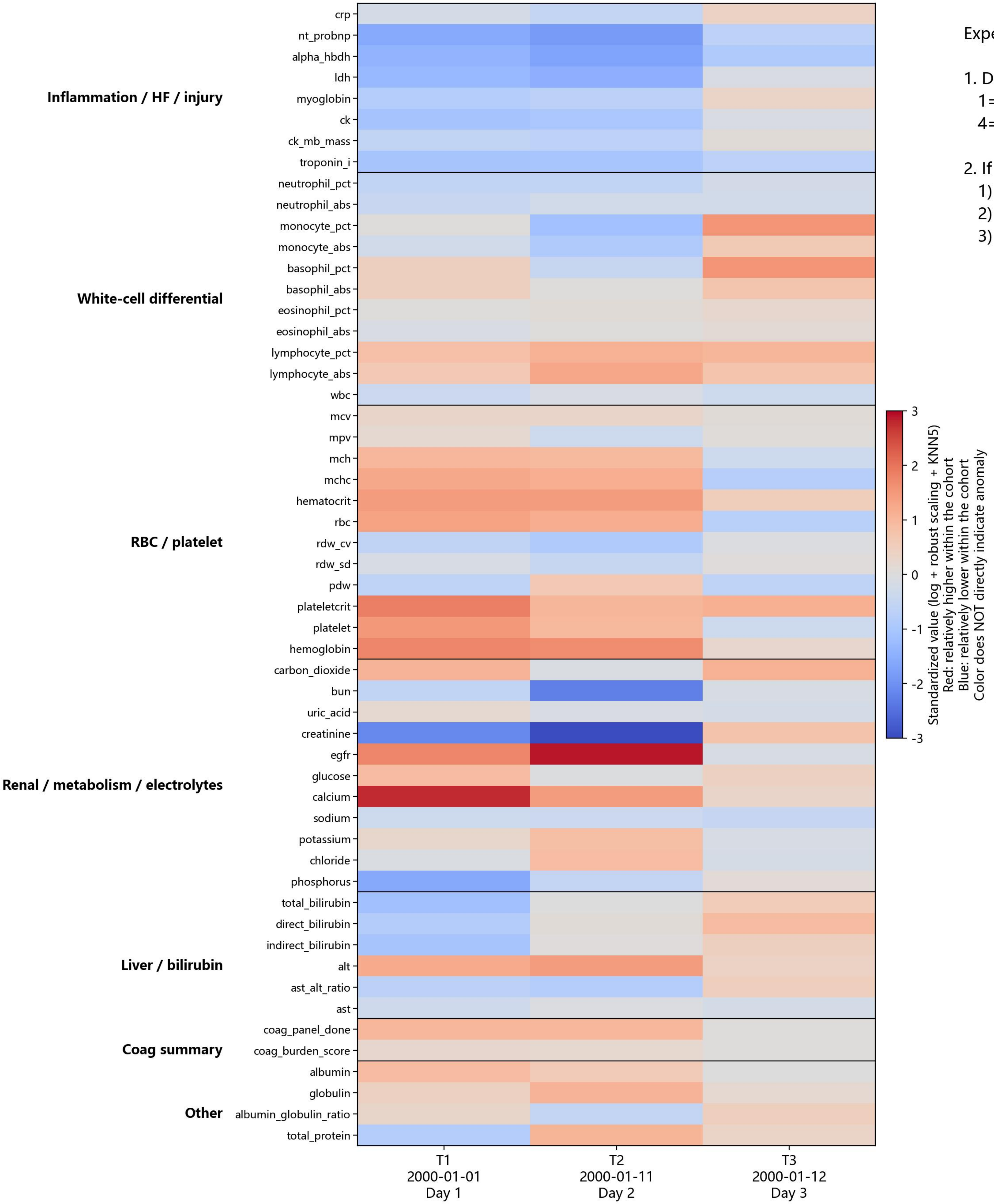

Expert review (blinded; no model score shown)

1. Degree of anomaly for this 3-point window (1-5):
   1=very typical; 2=relatively typical; 3=gray zone;
   4=relatively abnormal; 5=very abnormal

2. If scored 4-5, list the 3 most abnormal / noteworthy variables:
   1) ________________________
   2) ________________________
   3) ________________________

Patient-window heatmap card for blinded expert review
ID: P031   Window: W01

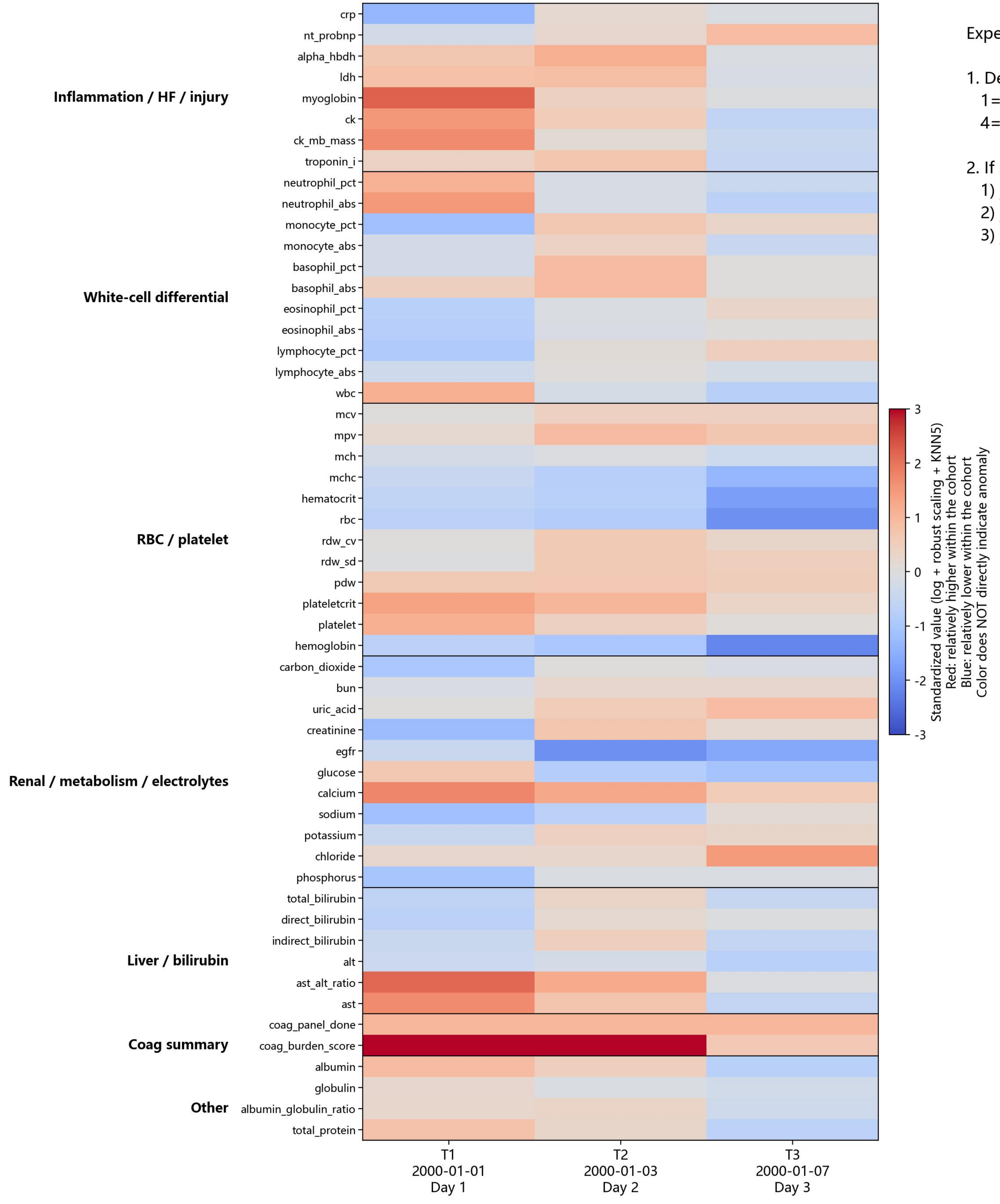

Expert review (blinded; no model score shown)

1. Degree of anomaly for this 3-point window (1-5):
1=very typical; 2=relatively typical; 3=gray zone;
4=relatively abnormal; 5=very abnormal

2. If scored 4-5, list the 3 most abnormal / noteworthy variables:
1) ________________________
2) ________________________
3) ________________________

Patient-window heatmap card for blinded expert review
ID: P032 Window: W01

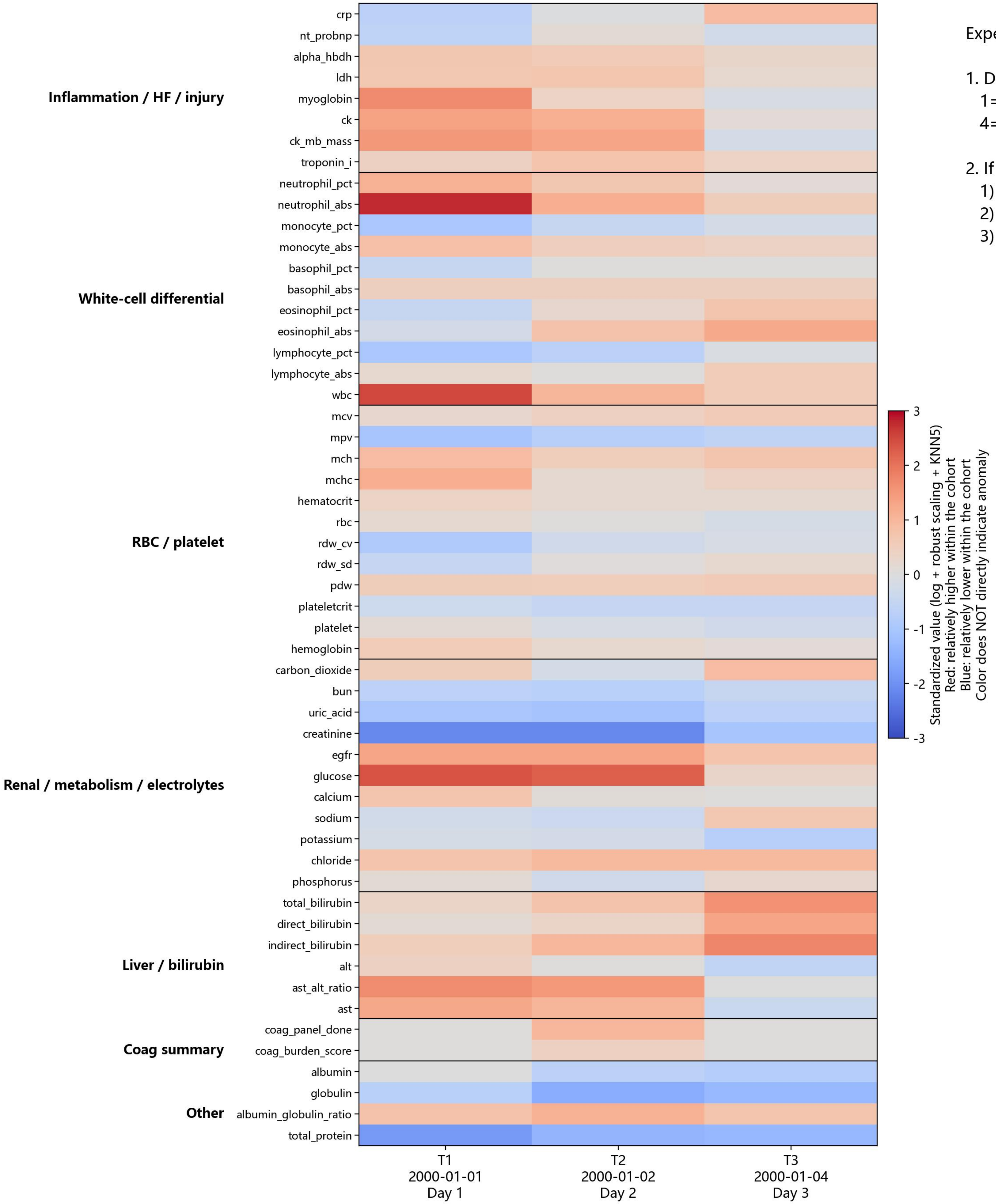

Expert review (blinded; no model score shown)

1. Degree of anomaly for this 3-point window (1-5):
   1=very typical; 2=relatively typical; 3=gray zone;
   4=relatively abnormal; 5=very abnormal

2. If scored 4-5, list the 3 most abnormal / noteworthy variables:
   1) ______________________
   2) ______________________
   3) ______________________

Patient-window heatmap card for blinded expert review
ID: P033 Window: W01

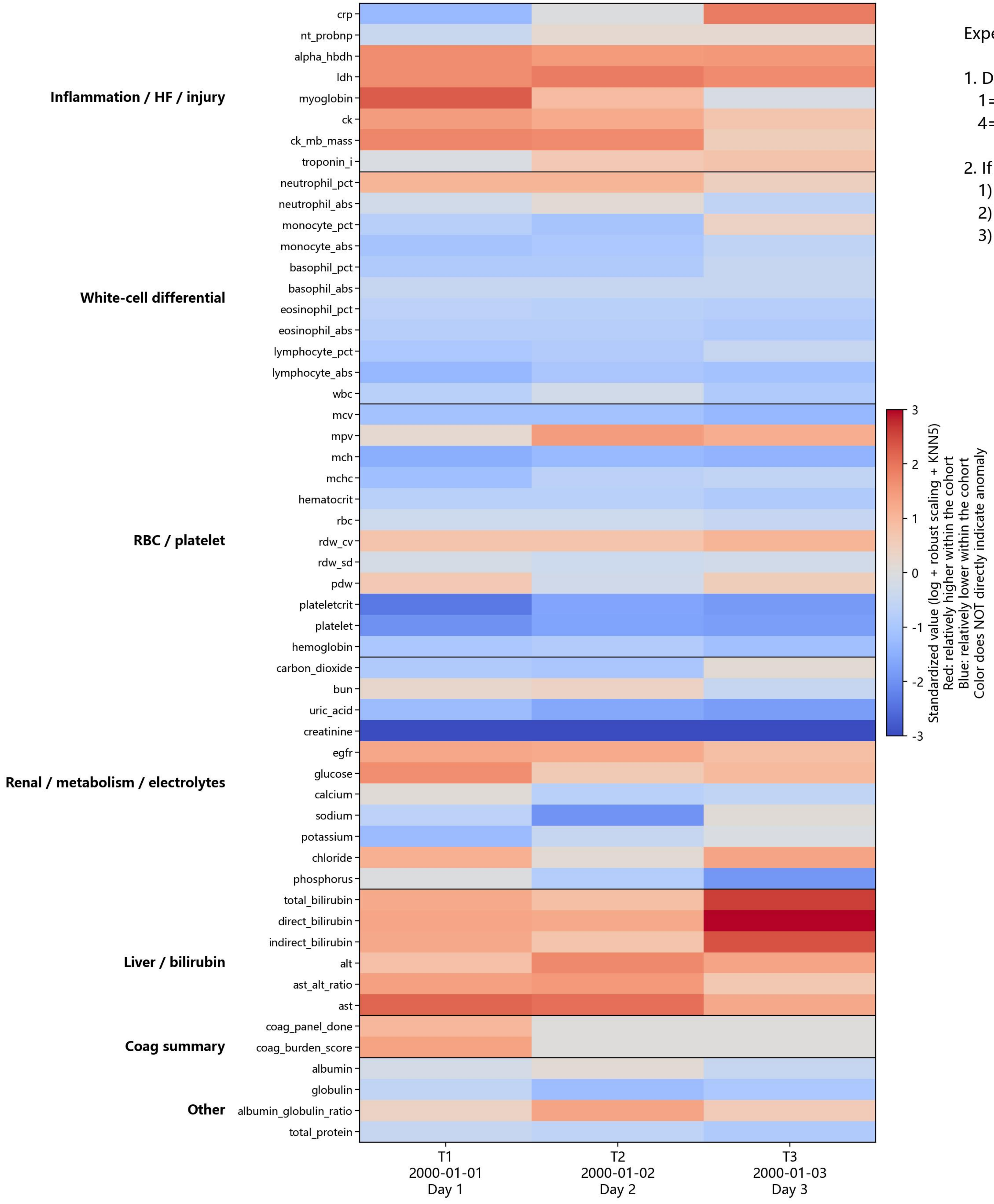

Expert review (blinded; no model score shown)

1. Degree of anomaly for this 3-point window (1-5):
   1=very typical; 2=relatively typical; 3=gray zone;
   4=relatively abnormal; 5=very abnormal

2. If scored 4-5, list the 3 most abnormal / noteworthy variables:
   1) ________________________
   2) ________________________
   3) ________________________

Patient-window heatmap card for blinded expert review
ID: P034 Window: W01

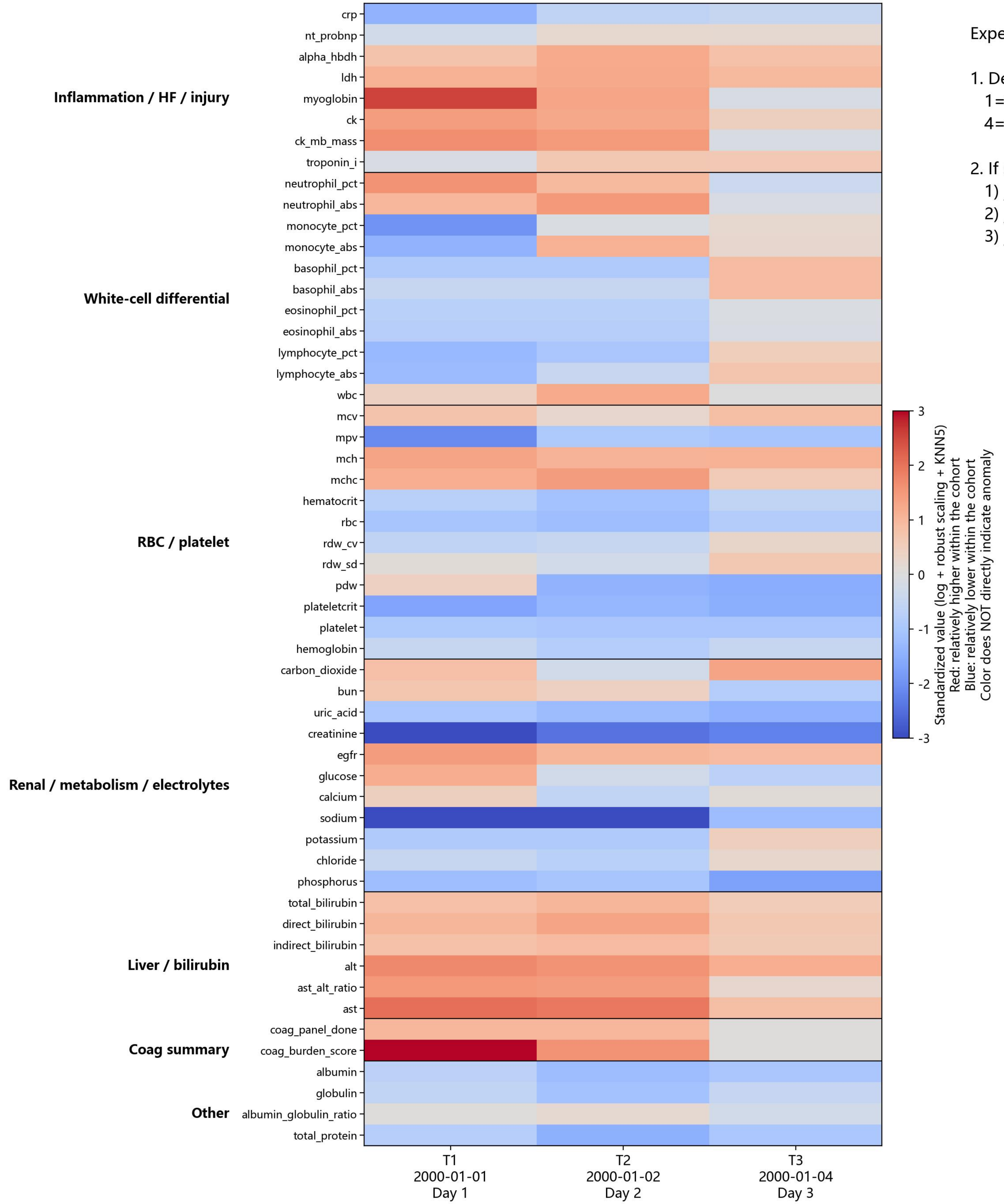

Expert review (blinded; no model score shown)

1. Degree of anomaly for this 3-point window (1-5):
   1=very typical; 2=relatively typical; 3=gray zone;
   4=relatively abnormal; 5=very abnormal

2. If scored 4-5, list the 3 most abnormal / noteworthy variables:
   1) ____________________________
   2) ____________________________
   3) ____________________________

Patient-window heatmap card for blinded expert review
ID: P035   Window: W01

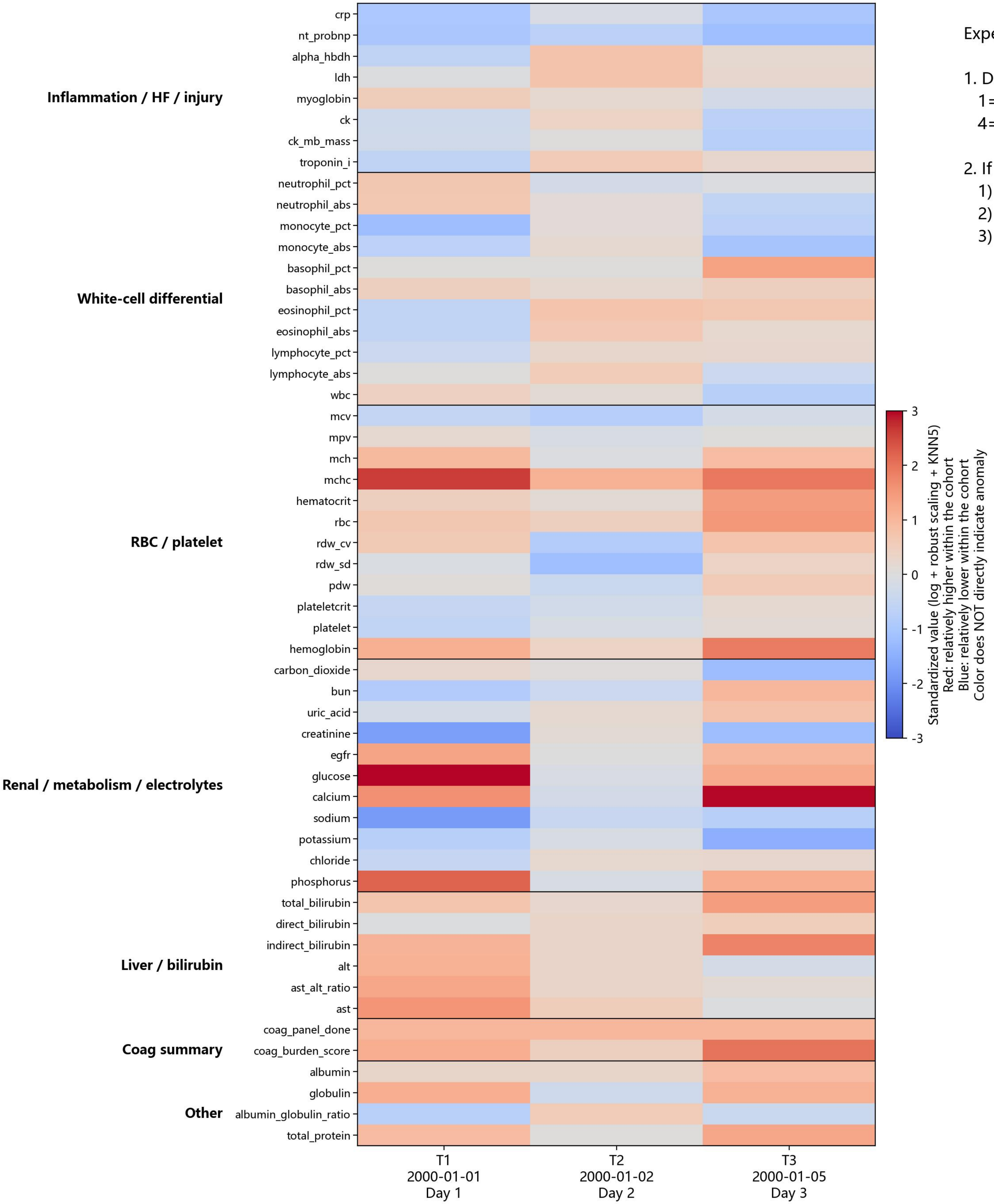

Expert review (blinded; no model score shown)

1. Degree of anomaly for this 3-point window (1-5):
   1=very typical; 2=relatively typical; 3=gray zone;
   4=relatively abnormal; 5=very abnormal

2. If scored 4-5, list the 3 most abnormal / noteworthy variables:
   1) __________________________
   2) __________________________
   3) __________________________

Patient-window heatmap card for blinded expert review
ID: P036   Window: W01

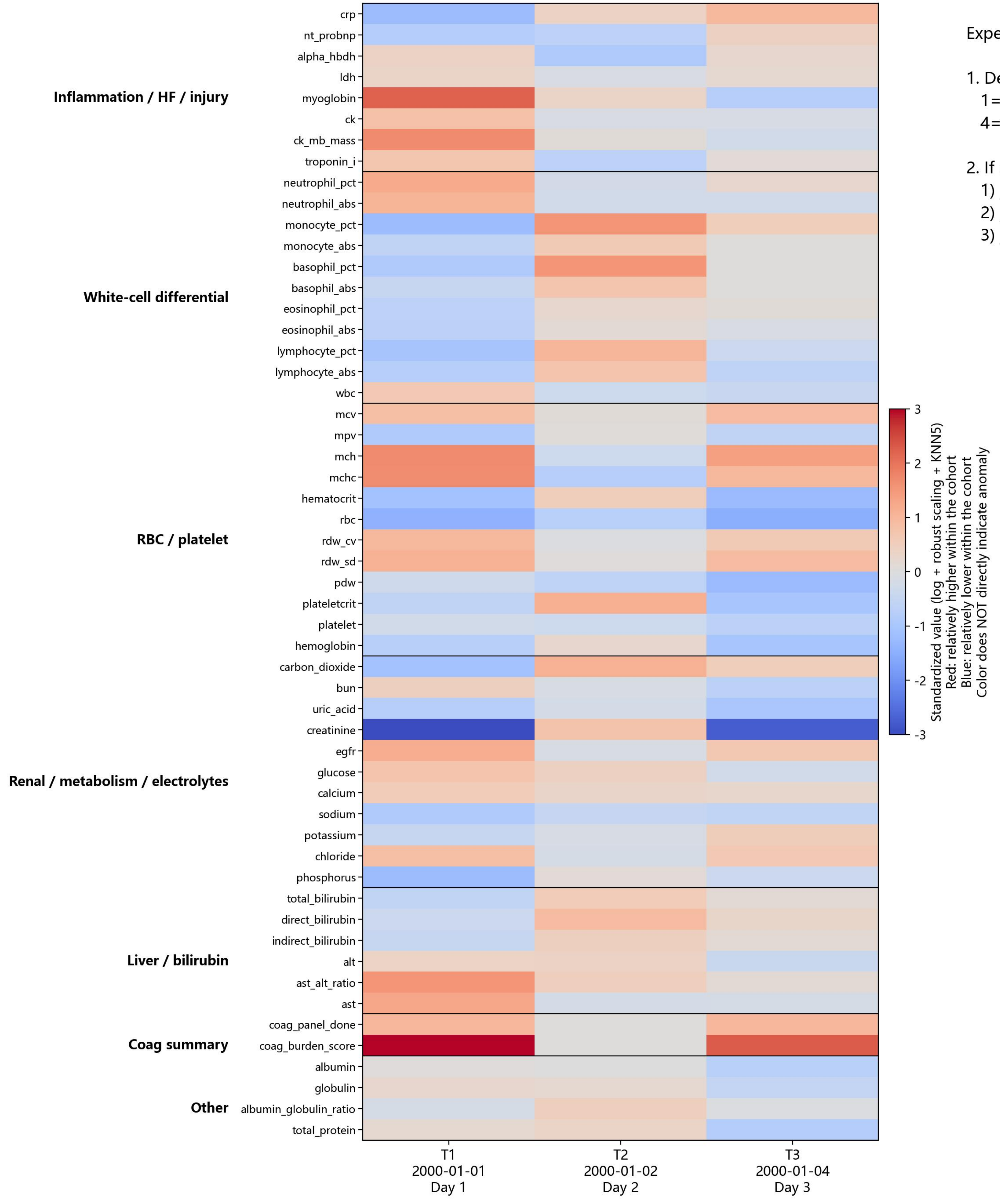

Expert review (blinded; no model score shown)

1. Degree of anomaly for this 3-point window (1-5):
   1=very typical; 2=relatively typical; 3=gray zone;
   4=relatively abnormal; 5=very abnormal

2. If scored 4-5, list the 3 most abnormal / noteworthy variables:
   1) ________________________
   2) ________________________
   3) ________________________

Patient-window heatmap card for blinded expert review
ID: P037   Window: W01

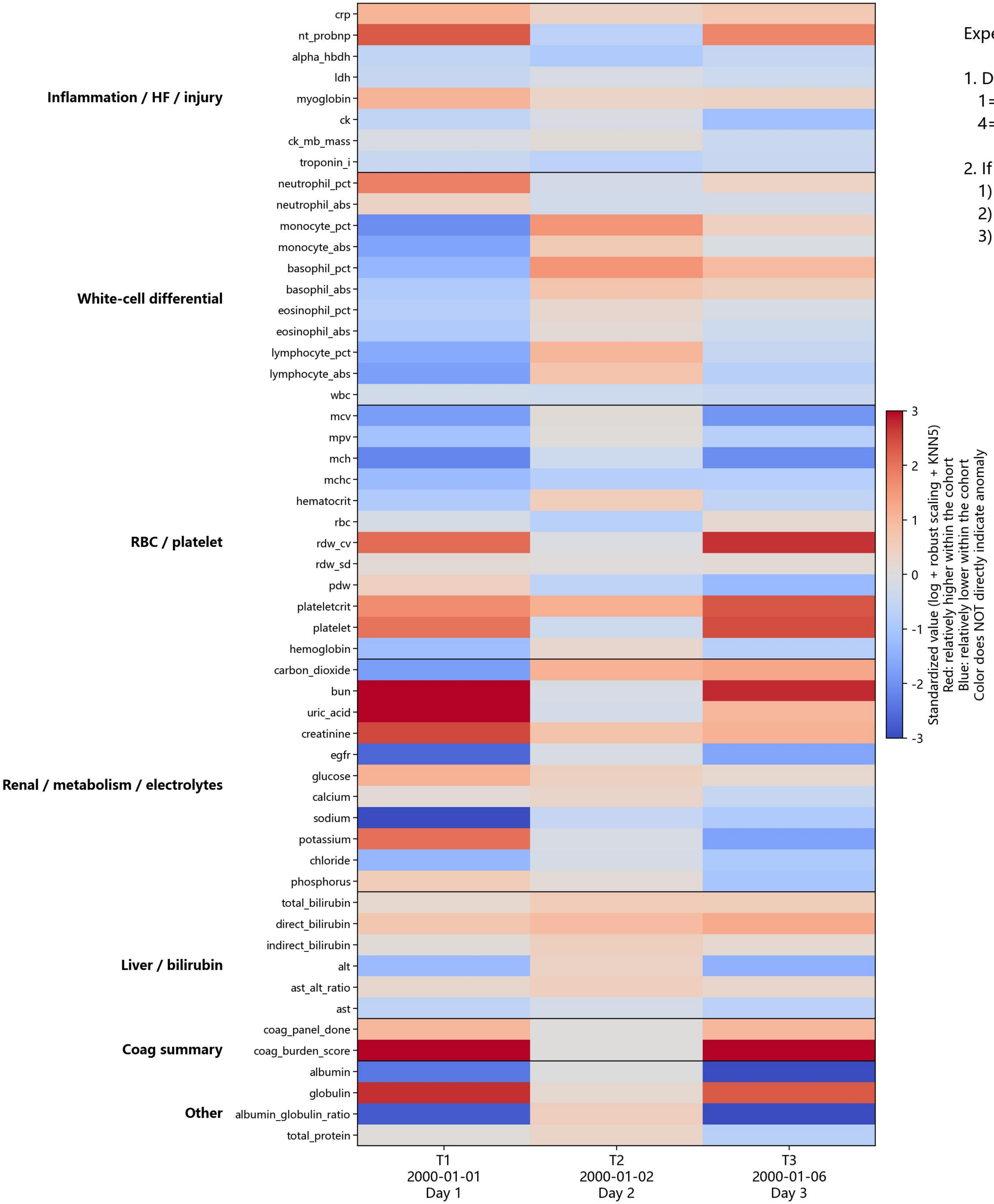

Expert review (blinded; no model score shown)

1. Degree of anomaly for this 3-point window (1-5):
   1=very typical; 2=relatively typical; 3=gray zone;
   4=relatively abnormal; 5=very abnormal

2. If scored 4-5, list the 3 most abnormal / noteworthy variables:
   1) ________________________
   2) ________________________
   3) ________________________

Patient-window heatmap card for blinded expert review
ID: P038   Window: W01

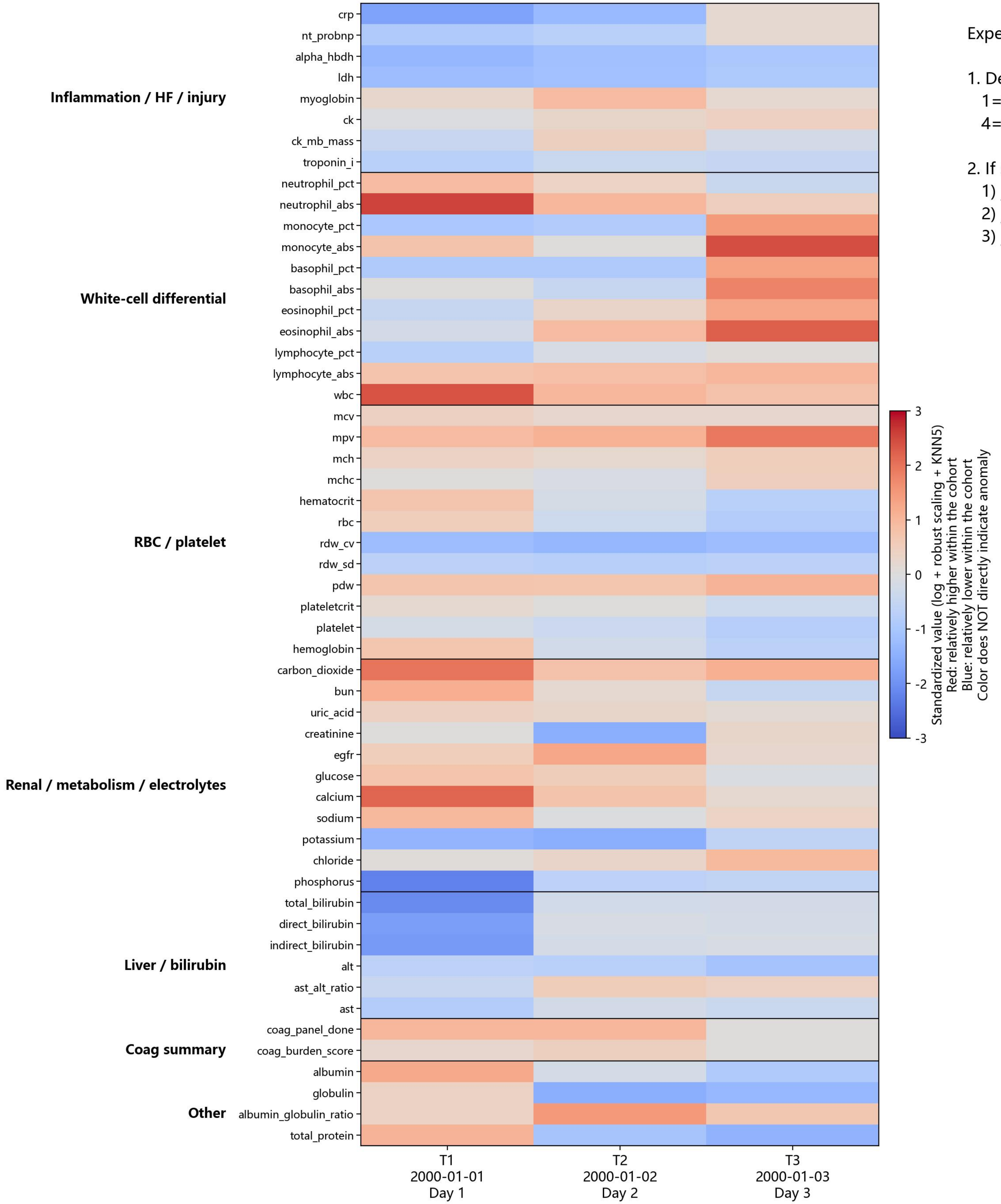

Expert review (blinded; no model score shown)

1. Degree of anomaly for this 3-point window (1-5):
   1=very typical; 2=relatively typical; 3=gray zone;
   4=relatively abnormal; 5=very abnormal

2. If scored 4-5, list the 3 most abnormal / noteworthy variables:
   1) ______________________
   2) ______________________
   3) ______________________

Patient-window heatmap card for blinded expert review
ID: P039   Window: W01

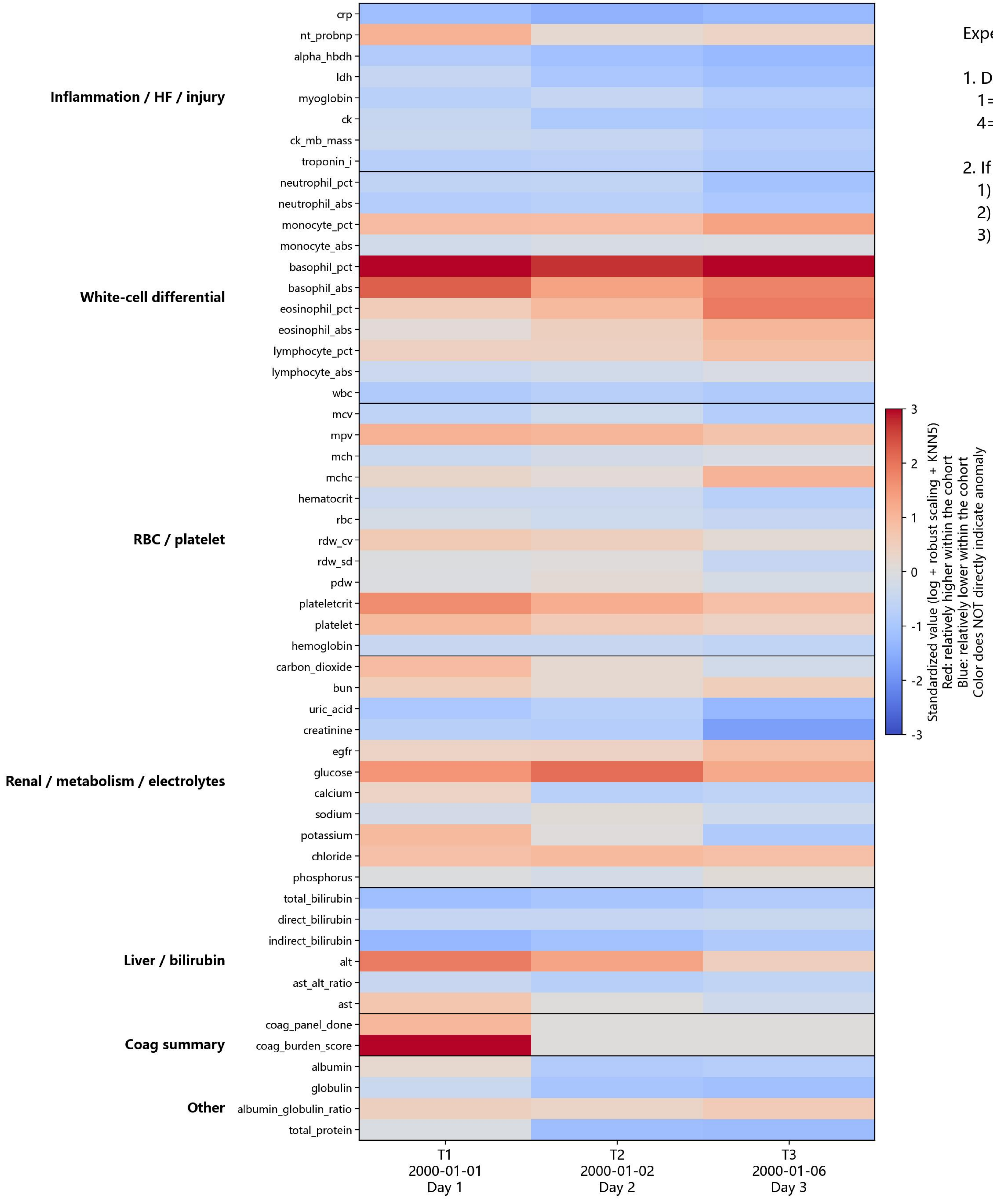

Expert review (blinded; no model score shown)

1. Degree of anomaly for this 3-point window (1-5):
   1=very typical; 2=relatively typical; 3=gray zone;
   4=relatively abnormal; 5=very abnormal

2. If scored 4-5, list the 3 most abnormal / noteworthy variables:
   1) ________________________
   2) ________________________
   3) ________________________

Patient-window heatmap card for blinded expert review
ID: P040   Window: W01

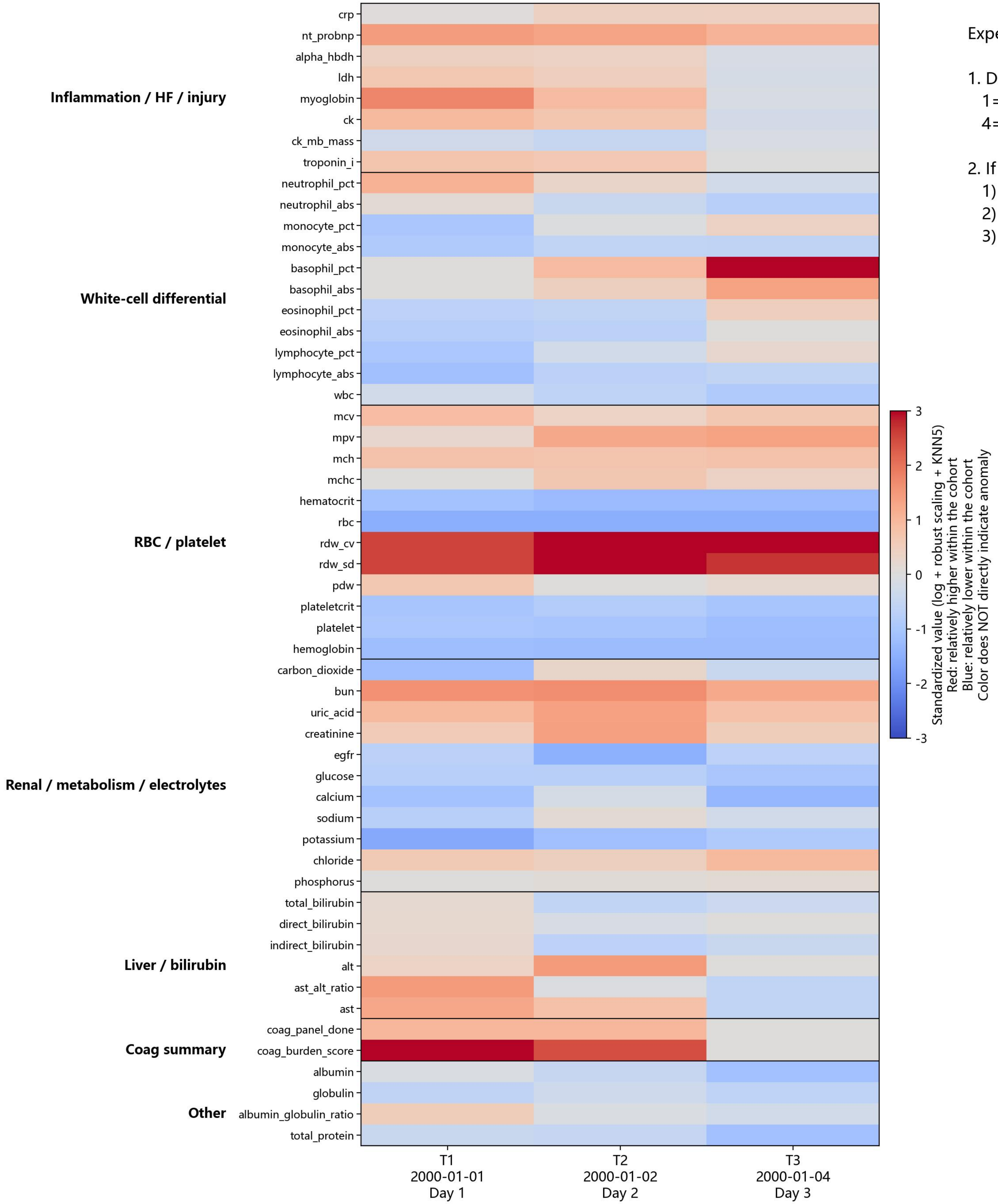

Expert review (blinded; no model score shown)

1. Degree of anomaly for this 3-point window (1-5):
   1=very typical; 2=relatively typical; 3=gray zone;
   4=relatively abnormal; 5=very abnormal

2. If scored 4-5, list the 3 most abnormal / noteworthy variables:
   1) ___________________________
   2) ___________________________
   3) ___________________________

Patient-window heatmap card for blinded expert review
ID: P041   Window: W01

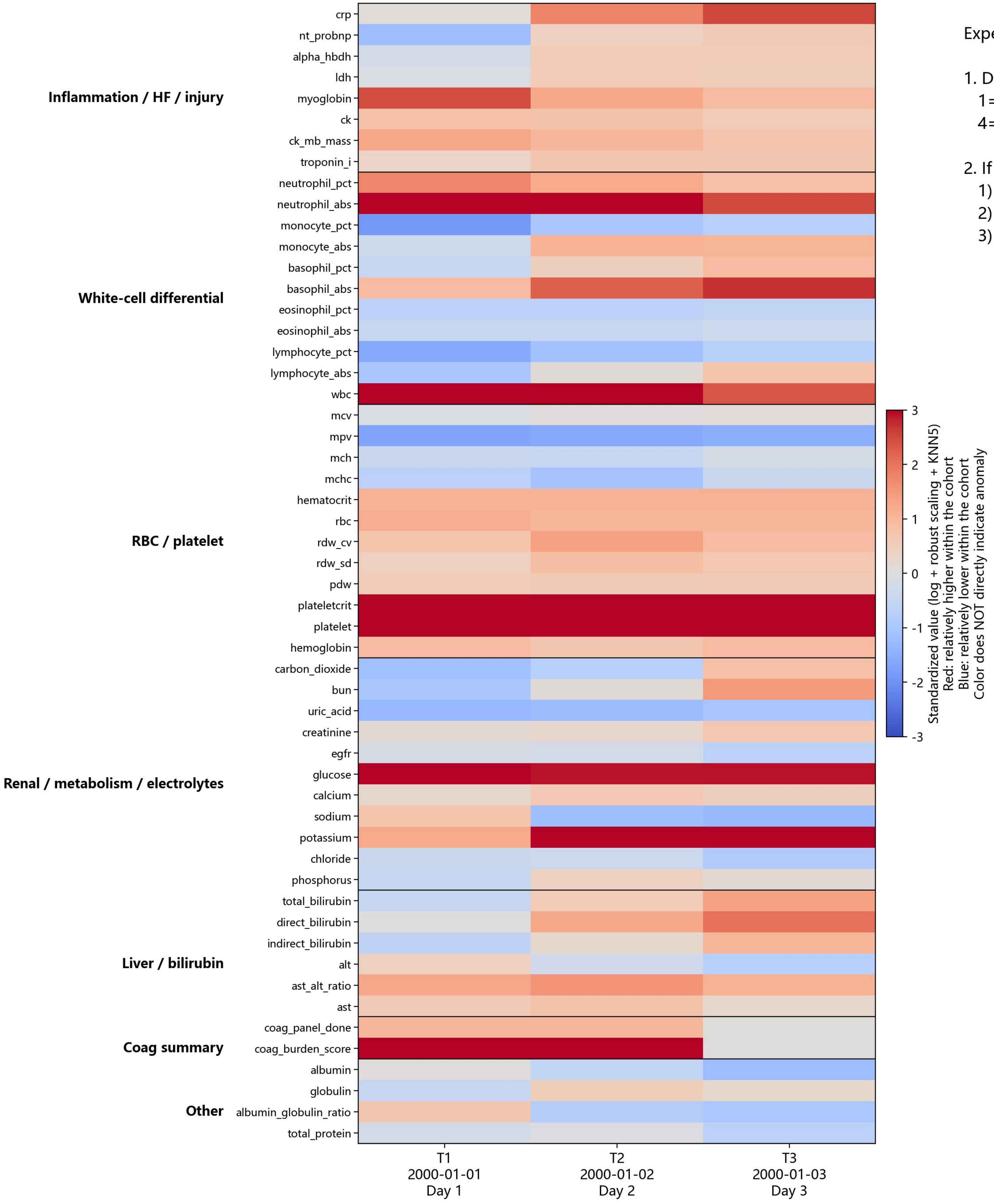

Expert review (blinded; no model score shown)

1. Degree of anomaly for this 3-point window (1-5):
   1=very typical; 2=relatively typical; 3=gray zone;
   4=relatively abnormal; 5=very abnormal

2. If scored 4-5, list the 3 most abnormal / noteworthy variables:
   1) ____________________________
   2) ____________________________
   3) ____________________________

Patient-window heatmap card for blinded expert review
ID: P042 Window: W01

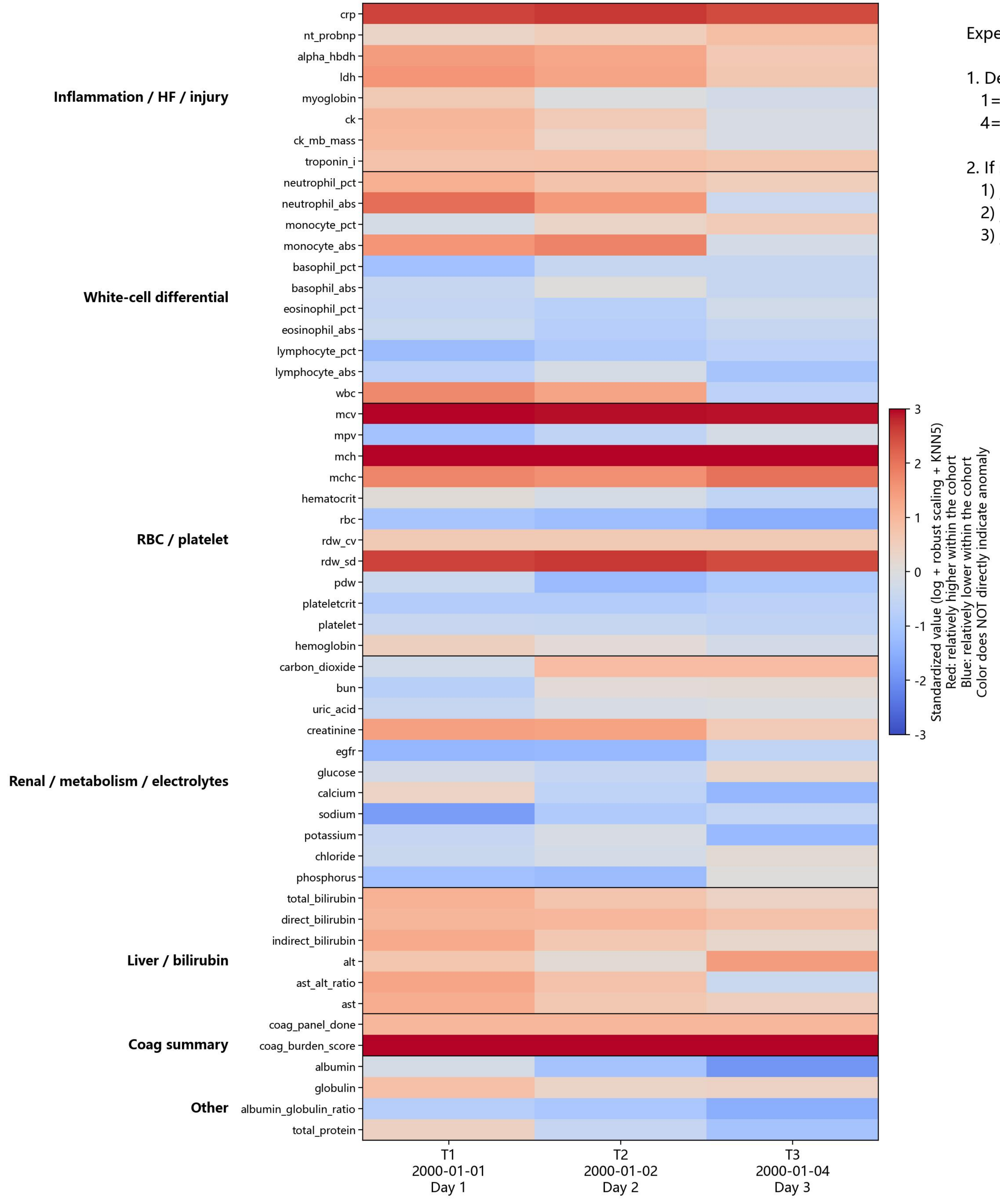

Expert review (blinded; no model score shown)

1. Degree of anomaly for this 3-point window (1-5):
1=very typical; 2=relatively typical; 3=gray zone;
4=relatively abnormal; 5=very abnormal

2. If scored 4-5, list the 3 most abnormal / noteworthy variables:
1) ________________________
2) ________________________
3) ________________________

Patient-window heatmap card for blinded expert review
ID: P043   Window: W01

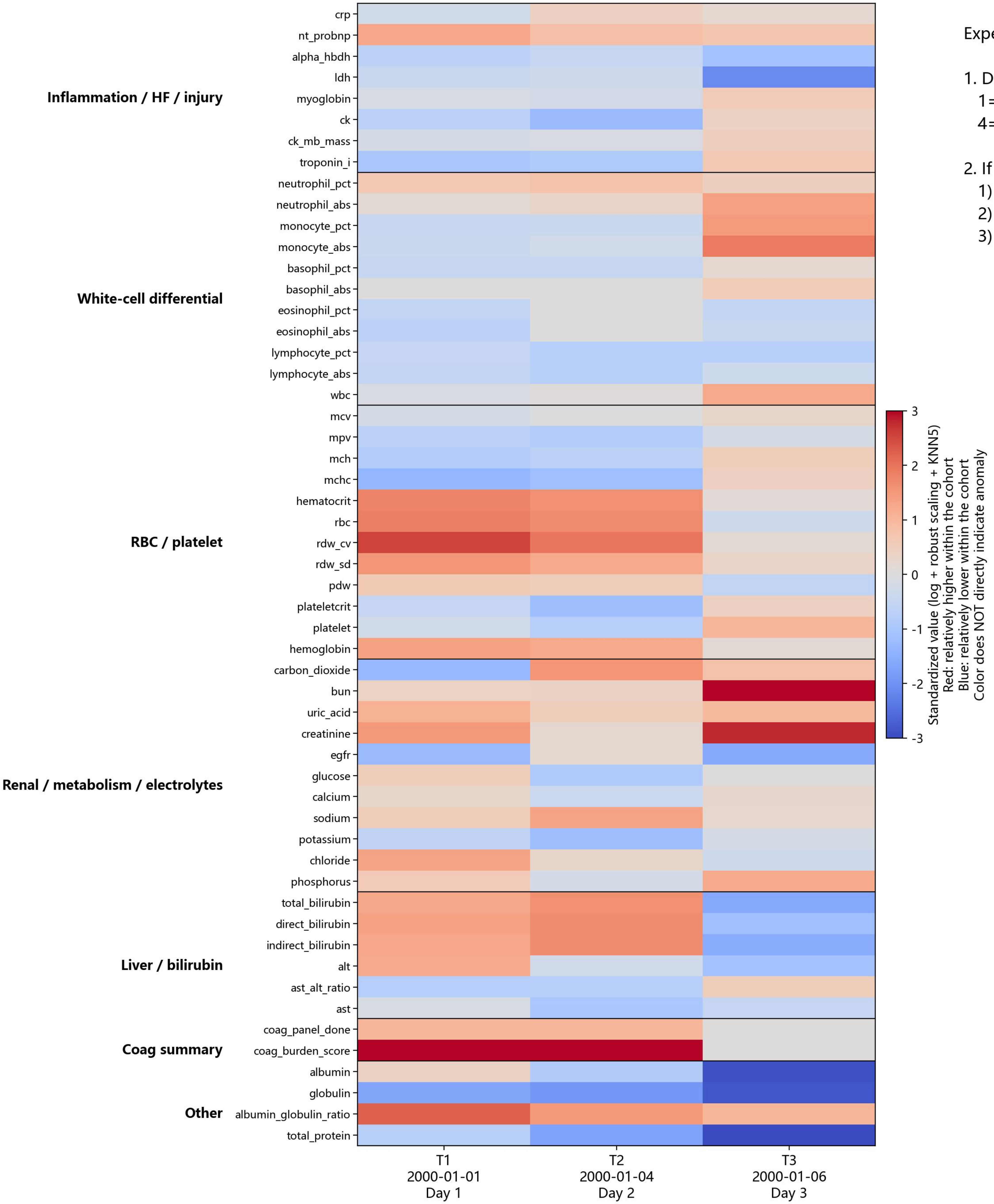

Expert review (blinded; no model score shown)

1. Degree of anomaly for this 3-point window (1-5):
   1=very typical; 2=relatively typical; 3=gray zone;
   4=relatively abnormal; 5=very abnormal

2. If scored 4-5, list the 3 most abnormal / noteworthy variables:
   1) ________________________
   2) ________________________
   3) ________________________

Patient-window heatmap card for blinded expert review
ID: P044 Window: W01

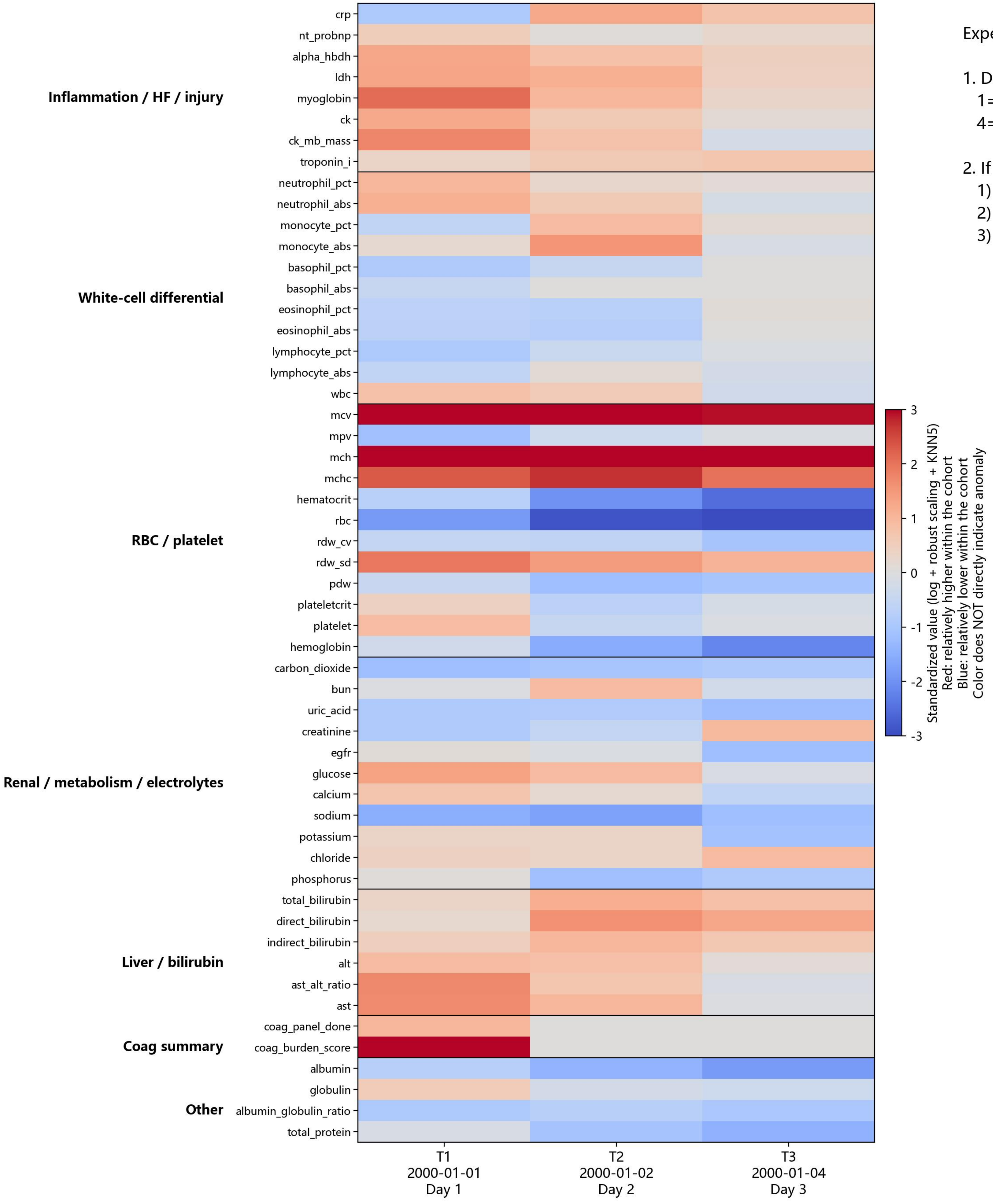

Expert review (blinded; no model score shown)

1. Degree of anomaly for this 3-point window (1-5):
   1=very typical; 2=relatively typical; 3=gray zone;
   4=relatively abnormal; 5=very abnormal

2. If scored 4-5, list the 3 most abnormal / noteworthy variables:
   1) ____________________________
   2) ____________________________
   3) ____________________________

Patient-window heatmap card for blinded expert review
ID: P045   Window: W01

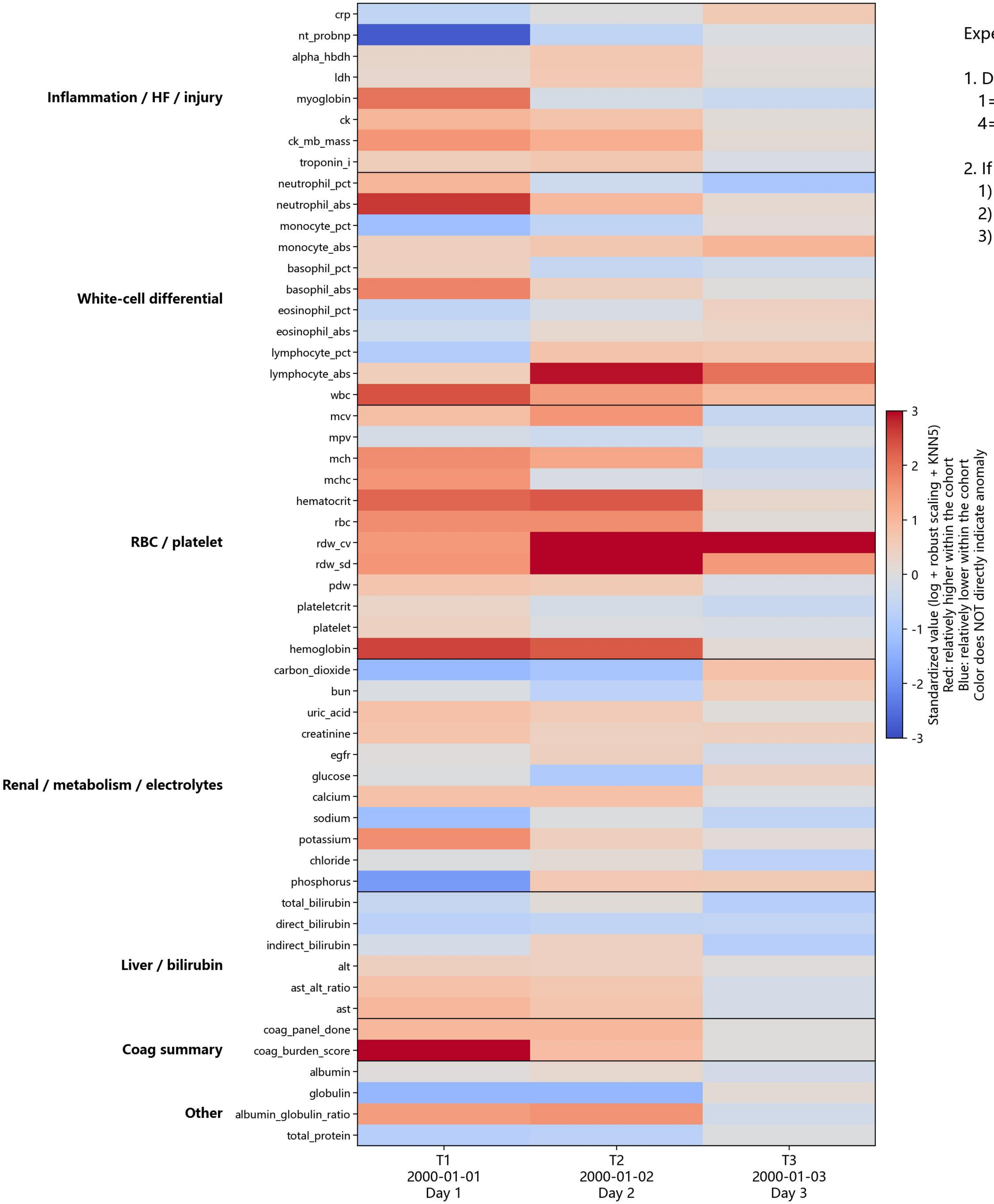

Expert review (blinded; no model score shown)

1. Degree of anomaly for this 3-point window (1-5):
   1=very typical; 2=relatively typical; 3=gray zone;
   4=relatively abnormal; 5=very abnormal

2. If scored 4-5, list the 3 most abnormal / noteworthy variables:
   1) ________________________
   2) ________________________
   3) ________________________

Patient-window heatmap card for blinded expert review
ID: P046   Window: W01

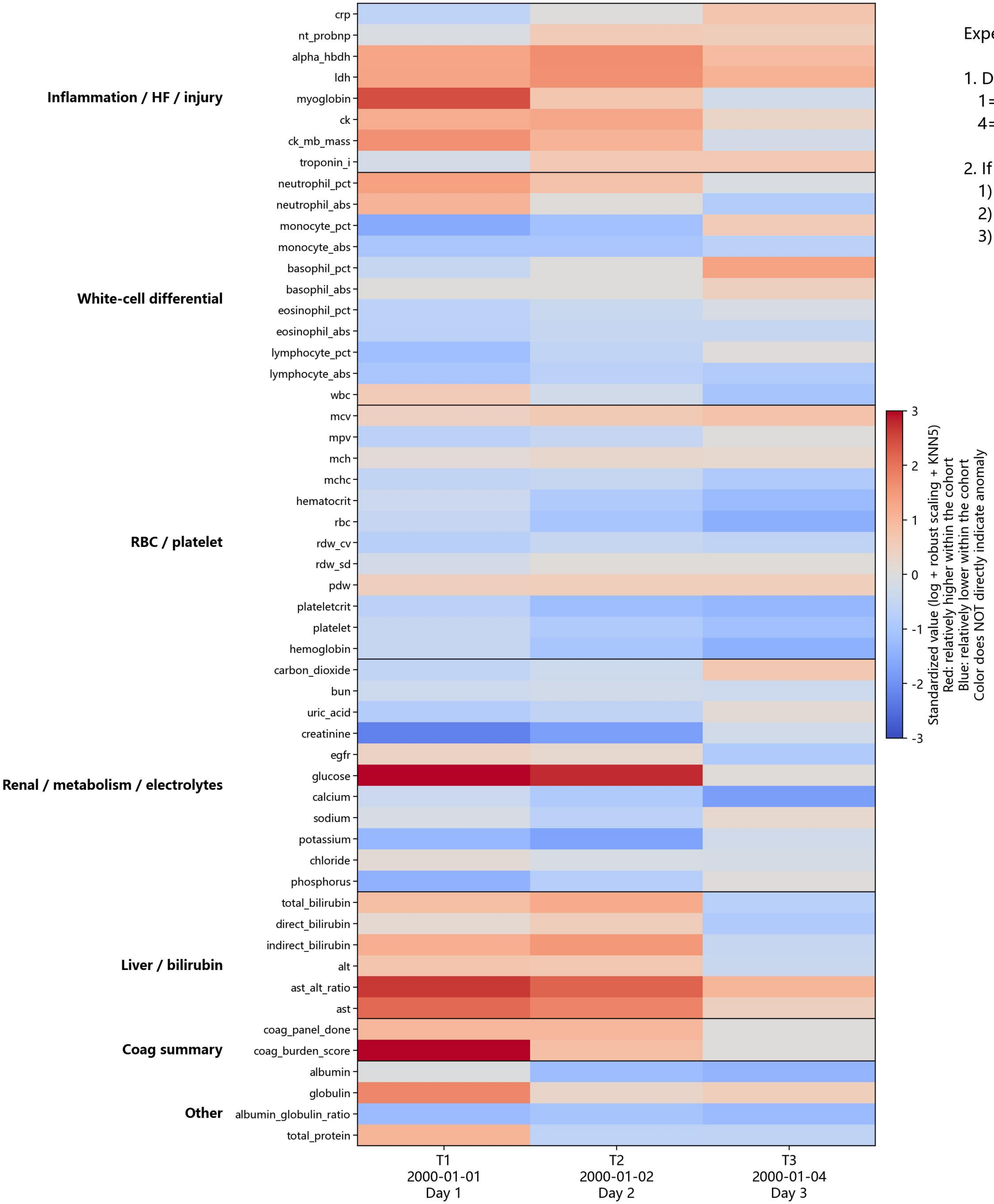

Expert review (blinded; no model score shown)

1. Degree of anomaly for this 3-point window (1-5):
   1=very typical; 2=relatively typical; 3=gray zone;
   4=relatively abnormal; 5=very abnormal

2. If scored 4-5, list the 3 most abnormal / noteworthy variables:
   1) ____________________________
   2) ____________________________
   3) ____________________________

Patient-window heatmap card for blinded expert review
ID: P047   Window: W01

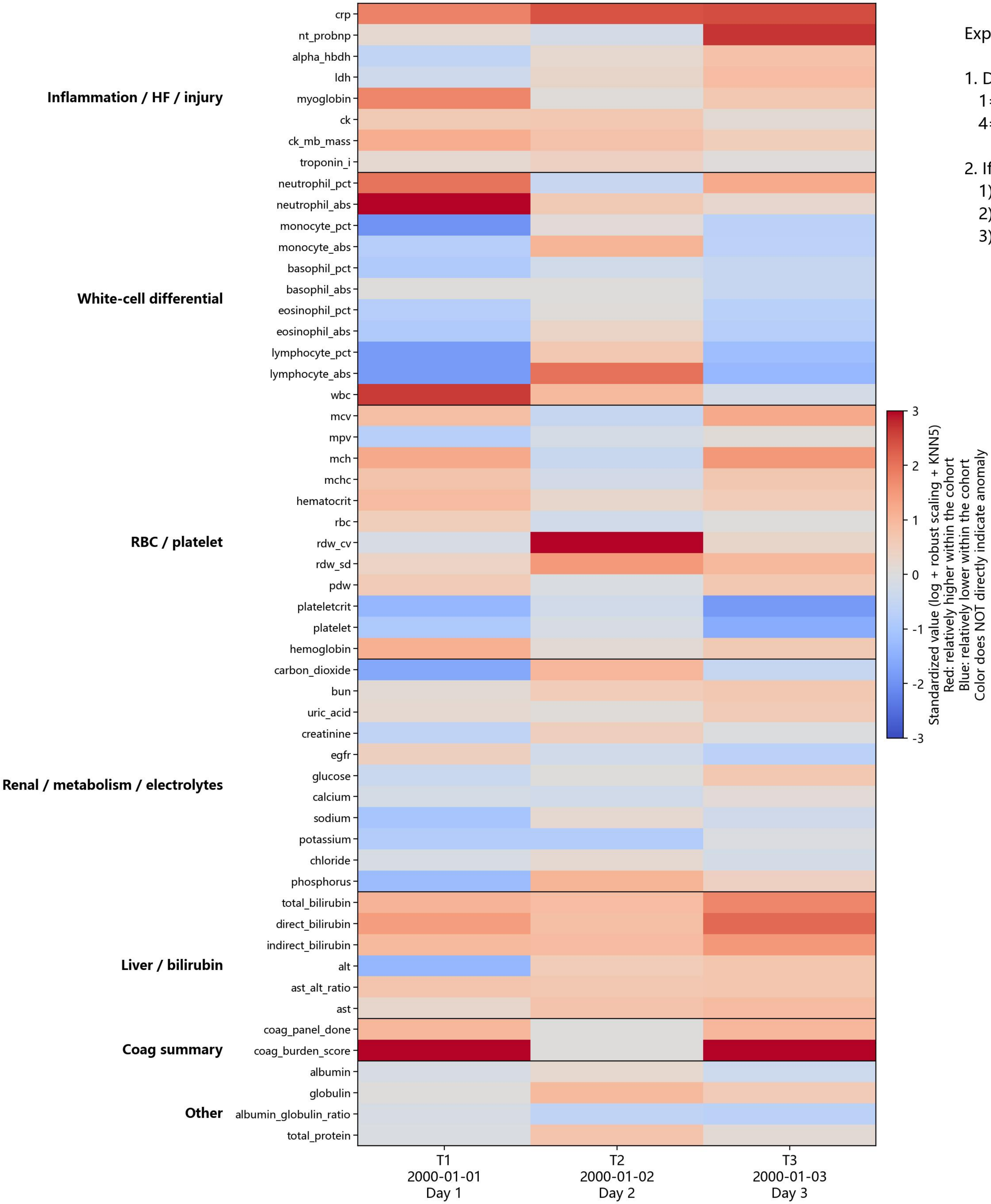

Expert review (blinded; no model score shown)

1. Degree of anomaly for this 3-point window (1-5):
   1=very typical; 2=relatively typical; 3=gray zone;
   4=relatively abnormal; 5=very abnormal

2. If scored 4-5, list the 3 most abnormal / noteworthy variables:
   1) ____________________________
   2) ____________________________
   3) ____________________________

Patient-window heatmap card for blinded expert review
ID: P048   Window: W01

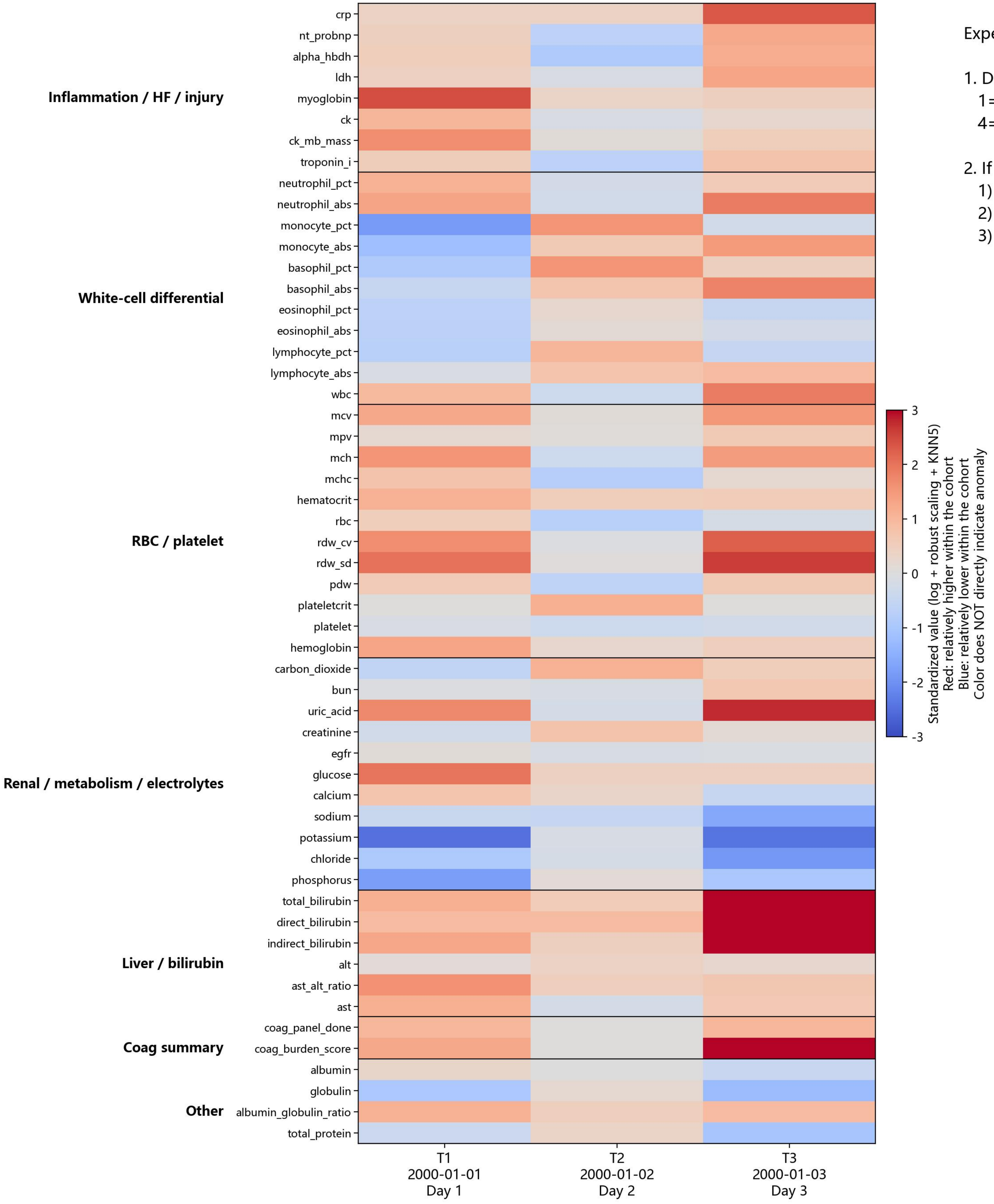

Expert review (blinded; no model score shown)

1. Degree of anomaly for this 3-point window (1-5):
   1=very typical; 2=relatively typical; 3=gray zone;
   4=relatively abnormal; 5=very abnormal

2. If scored 4-5, list the 3 most abnormal / noteworthy variables:
   1) ______________________
   2) ______________________
   3) ______________________

Patient-window heatmap card for blinded expert review
ID: P049 Window: W01

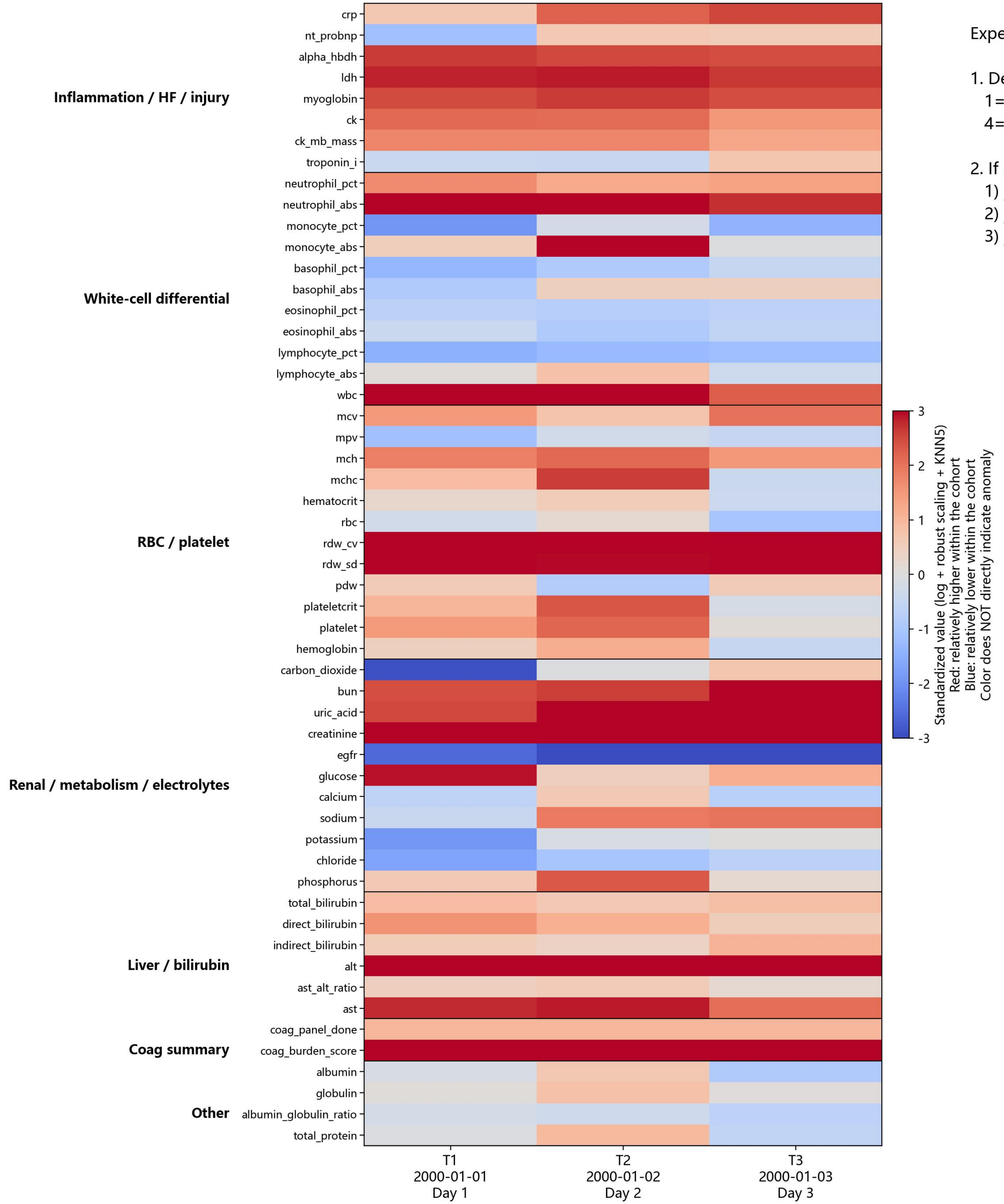

Expert review (blinded; no model score shown)

1. Degree of anomaly for this 3-point window (1-5):
   1=very typical; 2=relatively typical; 3=gray zone;
   4=relatively abnormal; 5=very abnormal

2. If scored 4-5, list the 3 most abnormal / noteworthy variables:
   1) ________________________
   2) ________________________
   3) ________________________

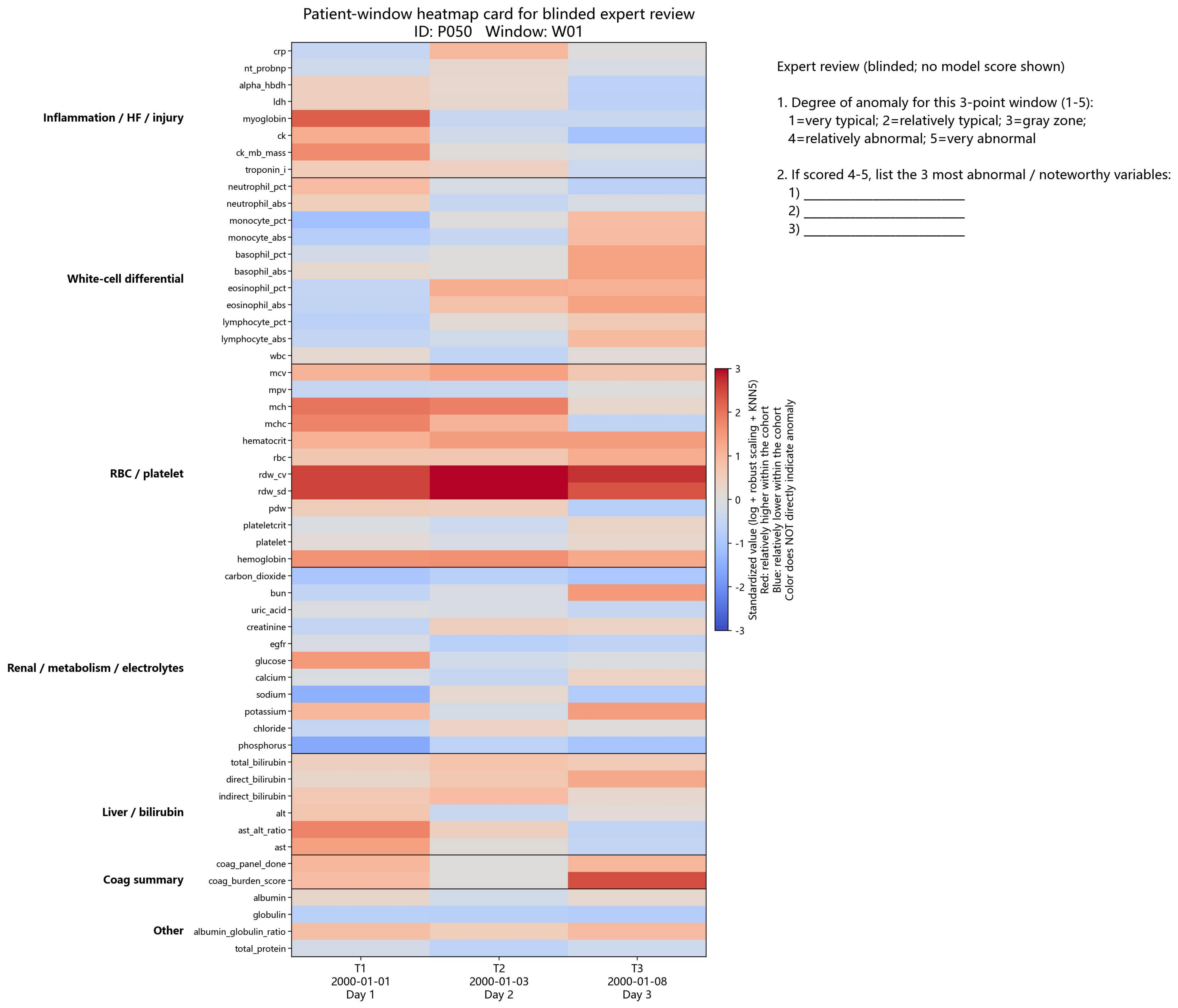

Patient-window heatmap card for blinded expert review
ID: P050 Window: W01
Inflammation / HF / injury
crp
nt_probnp
alpha_hbdh
ldh
myoglobin
ck
ck_mb_mass
troponin_i
White-cell differential
neutrophil_pct
neutrophil_abs
monocyte_pct
monocyte_abs
basophil_pct
basophil_abs
eosinophil_pct
eosinophil_abs
lymphocyte_pct
lymphocyte_abs
wbc
RBC / platelet
mcv
mpv
mch
mchc
hematocrit
rbc
rdw_cv
rdw_sd
pdw
plateletcrit
platelet
hemoglobin
Renal / metabolism / electrolytes
carbon_dioxide
bun
uric_acid
creatinine
egfr
glucose
calcium
sodium
potassium
chloride
phosphorus
Liver / bilirubin
total_bilirubin
direct_bilirubin
indirect_bilirubin
alt
ast_alt_ratio
ast
Coag summary
coag_panel_done
coag_burden_score
Other
albumin
globulin
albumin_globulin_ratio
total_protein
T1
2000-01-01
Day 1
T2
2000-01-03
Day 2
T3
2000-01-08
Day 3
3
2
1
0
-1
-2
-3
Standardized value (log + robust scaling + KNN5)
Red: relatively higher within the cohort
Blue: relatively lower within the cohort
Color does NOT directly indicate anomaly
Expert review (blinded; no model score shown)
1. Degree of anomaly for this 3-point window (1-5):
1=very typical; 2=relatively typical; 3=gray zone;
4=relatively abnormal; 5=very abnormal
2. If scored 4-5, list the 3 most abnormal / noteworthy variables:
1) ________________________
2) ________________________
3) ________________________

Patient-window heatmap card for blinded expert review
ID: P051   Window: W01

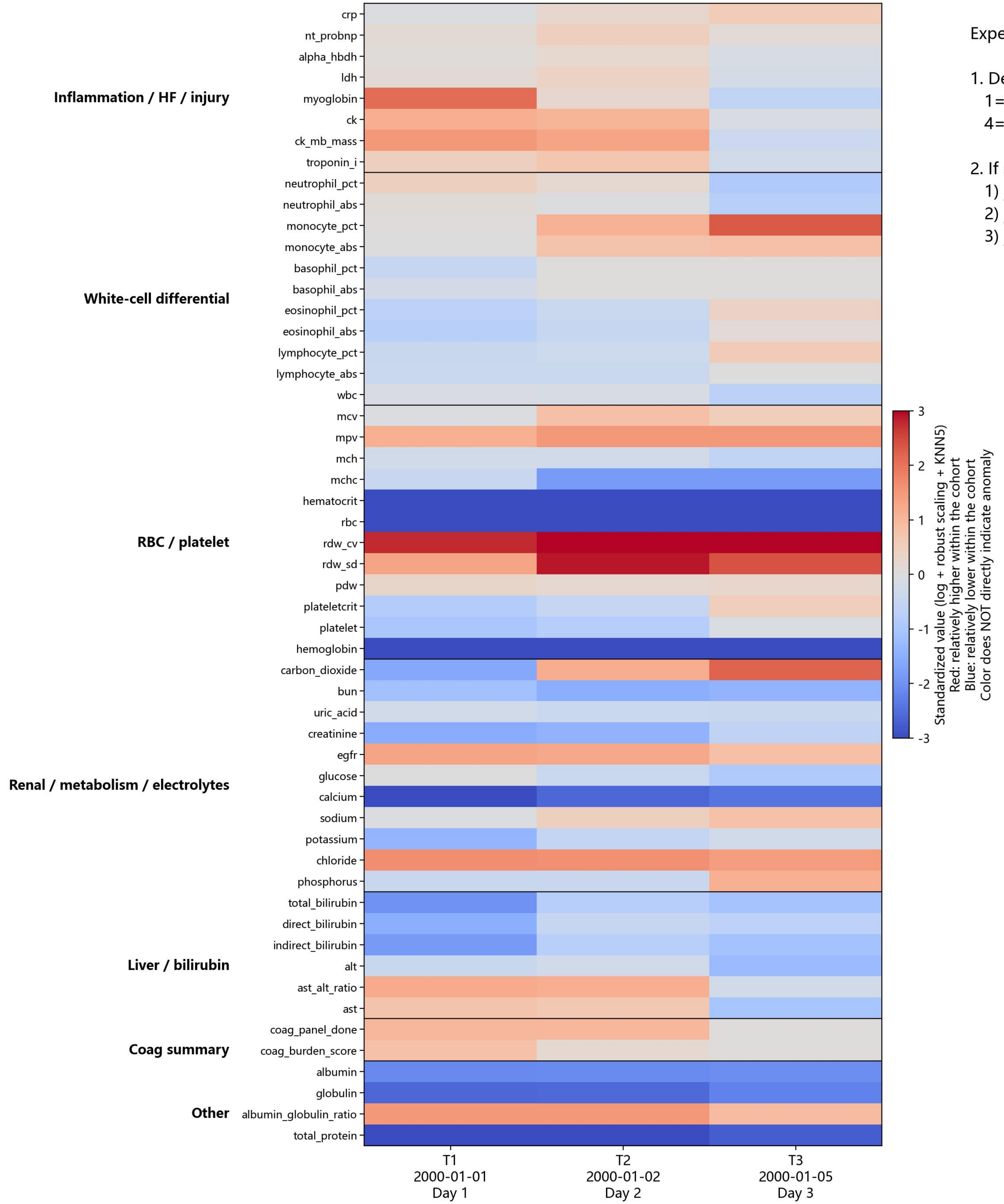

Expert review (blinded; no model score shown)

1. Degree of anomaly for this 3-point window (1-5):
   1=very typical; 2=relatively typical; 3=gray zone;
   4=relatively abnormal; 5=very abnormal

2. If scored 4-5, list the 3 most abnormal / noteworthy variables:
   1) ________________________
   2) ________________________
   3) ________________________

Patient-window heatmap card for blinded expert review
ID: P052 Window: W01

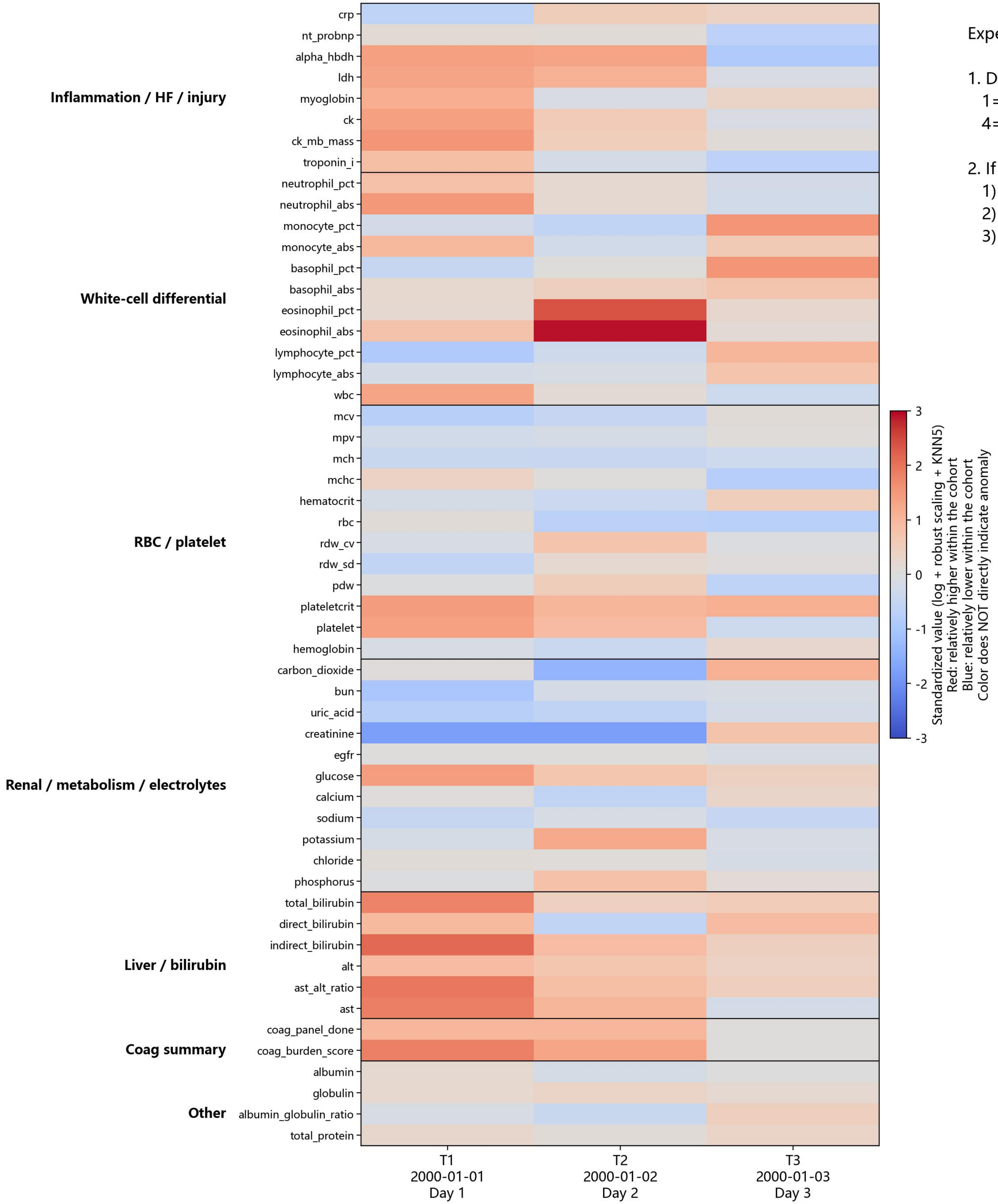

Expert review (blinded; no model score shown)

1. Degree of anomaly for this 3-point window (1-5):
   1=very typical; 2=relatively typical; 3=gray zone;
   4=relatively abnormal; 5=very abnormal

2. If scored 4-5, list the 3 most abnormal / noteworthy variables:
   1) ________________________
   2) ________________________
   3) ________________________

Patient-window heatmap card for blinded expert review
ID: P053   Window: W01

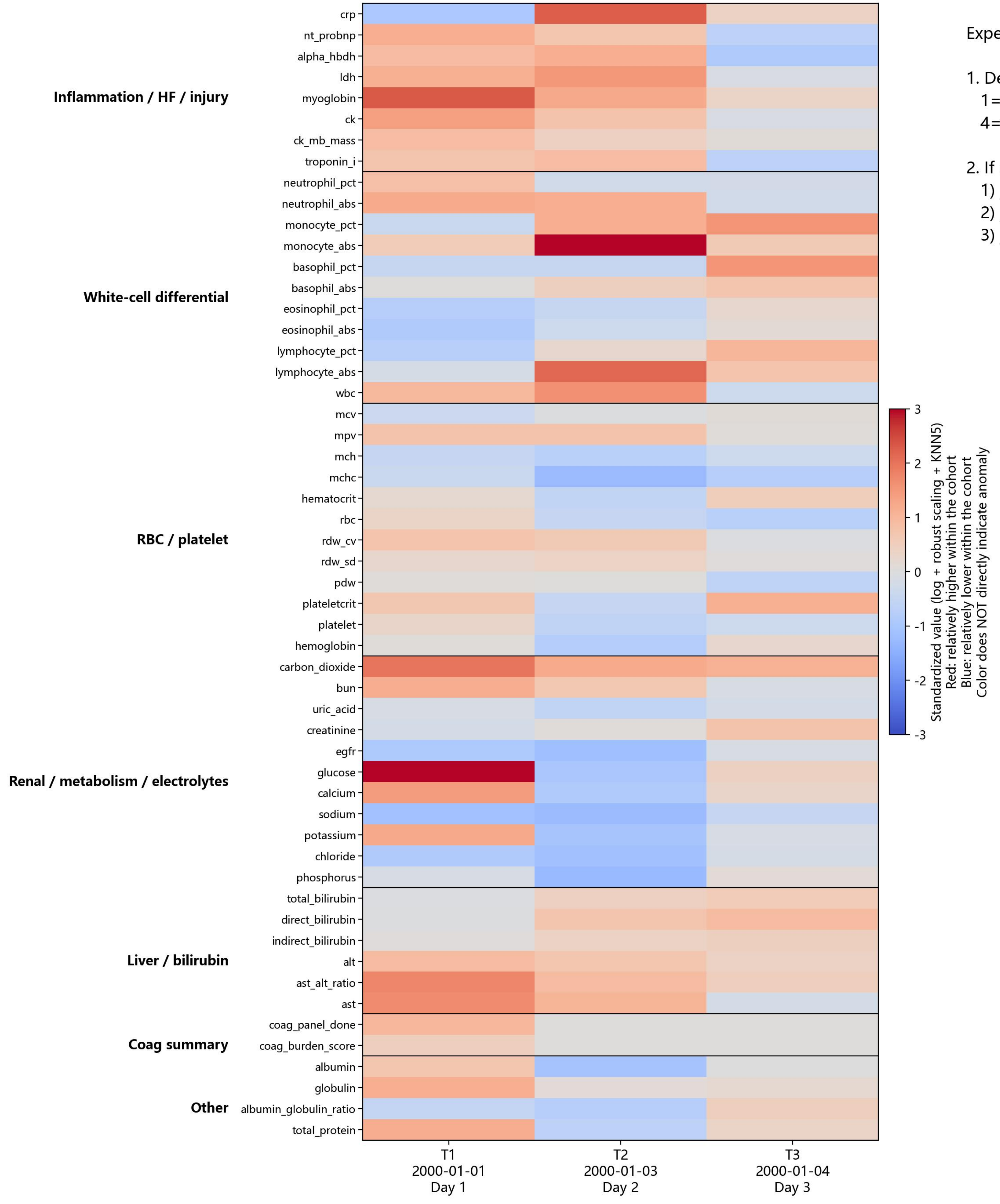

Expert review (blinded; no model score shown)

1. Degree of anomaly for this 3-point window (1-5):
   1=very typical; 2=relatively typical; 3=gray zone;
   4=relatively abnormal; 5=very abnormal

2. If scored 4-5, list the 3 most abnormal / noteworthy variables:
   1) ________________________
   2) ________________________
   3) ________________________

Patient-window heatmap card for blinded expert review
ID: P054   Window: W01

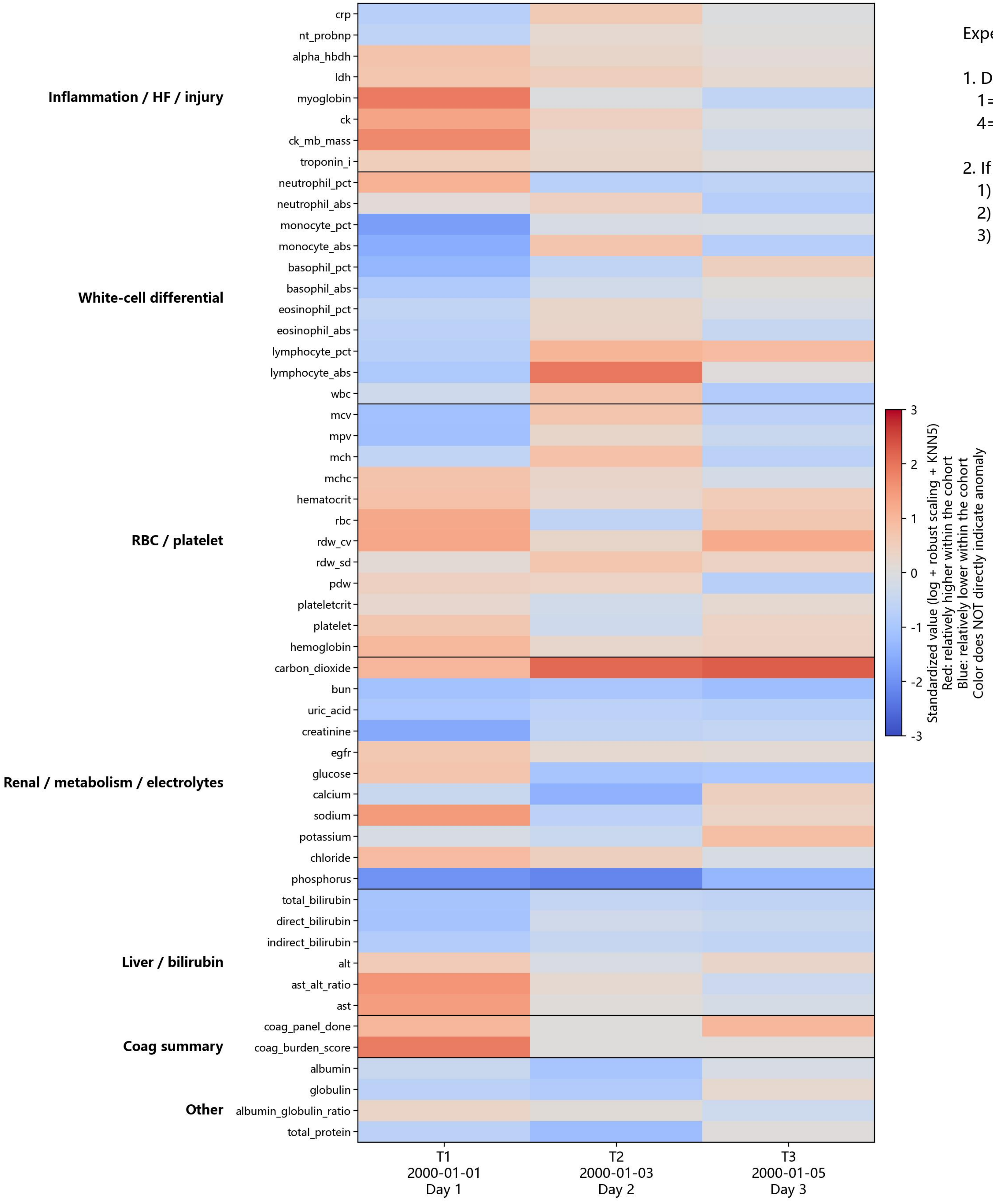

Expert review (blinded; no model score shown)

1. Degree of anomaly for this 3-point window (1-5):
   1=very typical; 2=relatively typical; 3=gray zone;
   4=relatively abnormal; 5=very abnormal

2. If scored 4-5, list the 3 most abnormal / noteworthy variables:
   1) ________________________
   2) ________________________
   3) ________________________

Patient-window heatmap card for blinded expert review
ID: P055   Window: W01

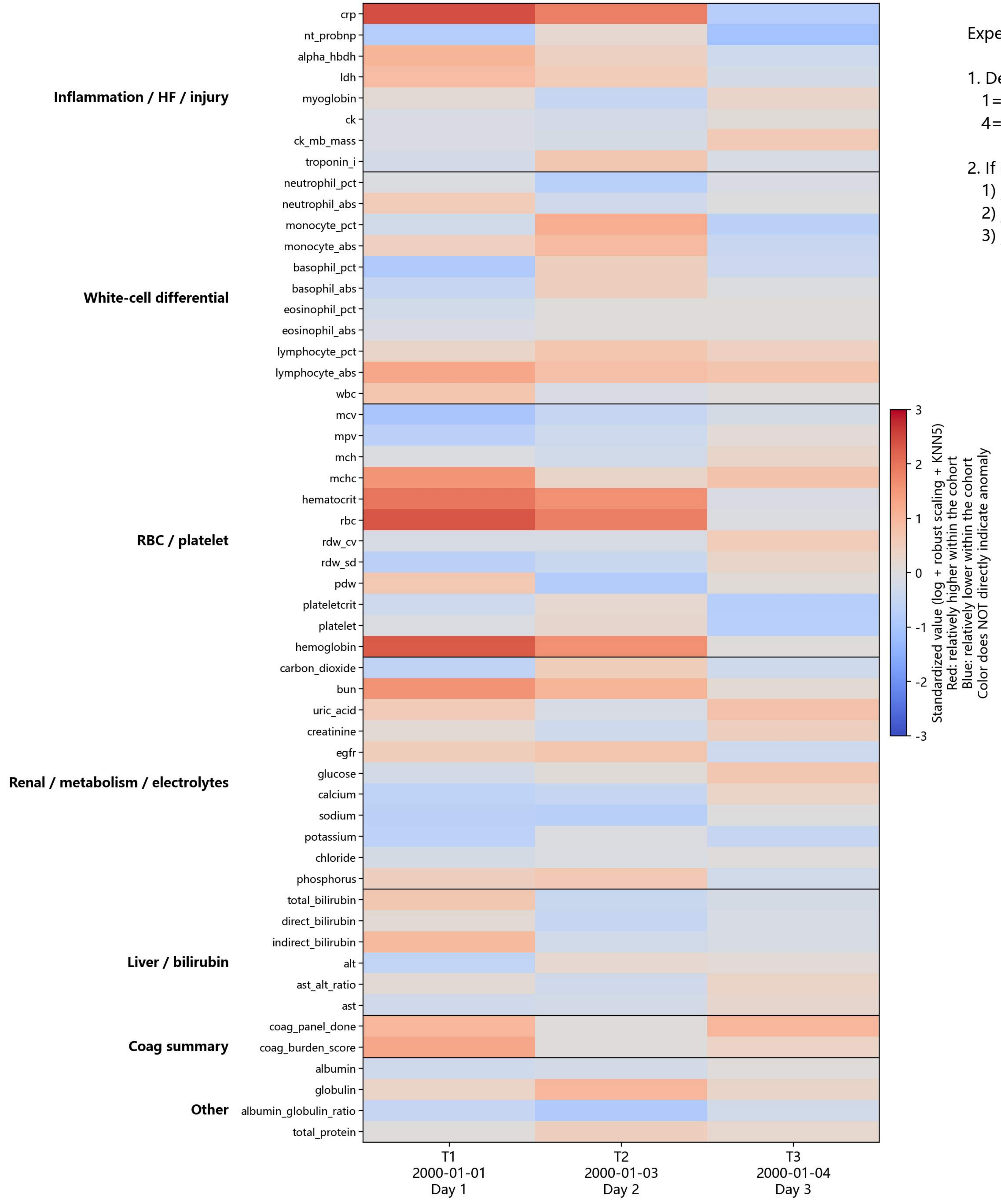

Expert review (blinded; no model score shown)

1. Degree of anomaly for this 3-point window (1-5):
   1=very typical; 2=relatively typical; 3=gray zone;
   4=relatively abnormal; 5=very abnormal

2. If scored 4-5, list the 3 most abnormal / noteworthy variables:
   1) ________________________
   2) ________________________
   3) ________________________

Patient-window heatmap card for blinded expert review
ID: P056   Window: W01

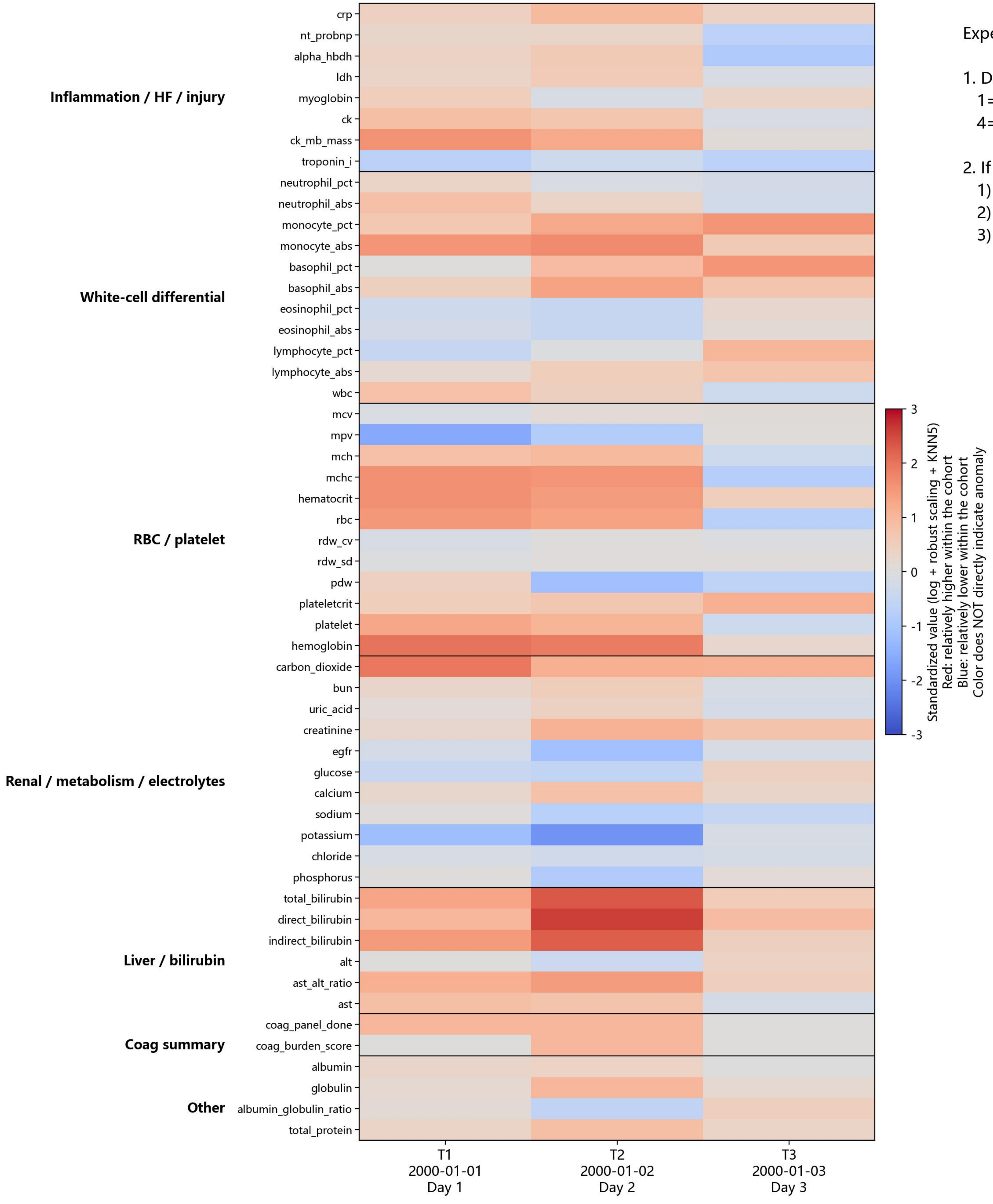

Expert review (blinded; no model score shown)

1. Degree of anomaly for this 3-point window (1-5):
   1=very typical; 2=relatively typical; 3=gray zone;
   4=relatively abnormal; 5=very abnormal

2. If scored 4-5, list the 3 most abnormal / noteworthy variables:
   1) ________________________
   2) ________________________
   3) ________________________

Patient-window heatmap card for blinded expert review
ID: P057   Window: W01

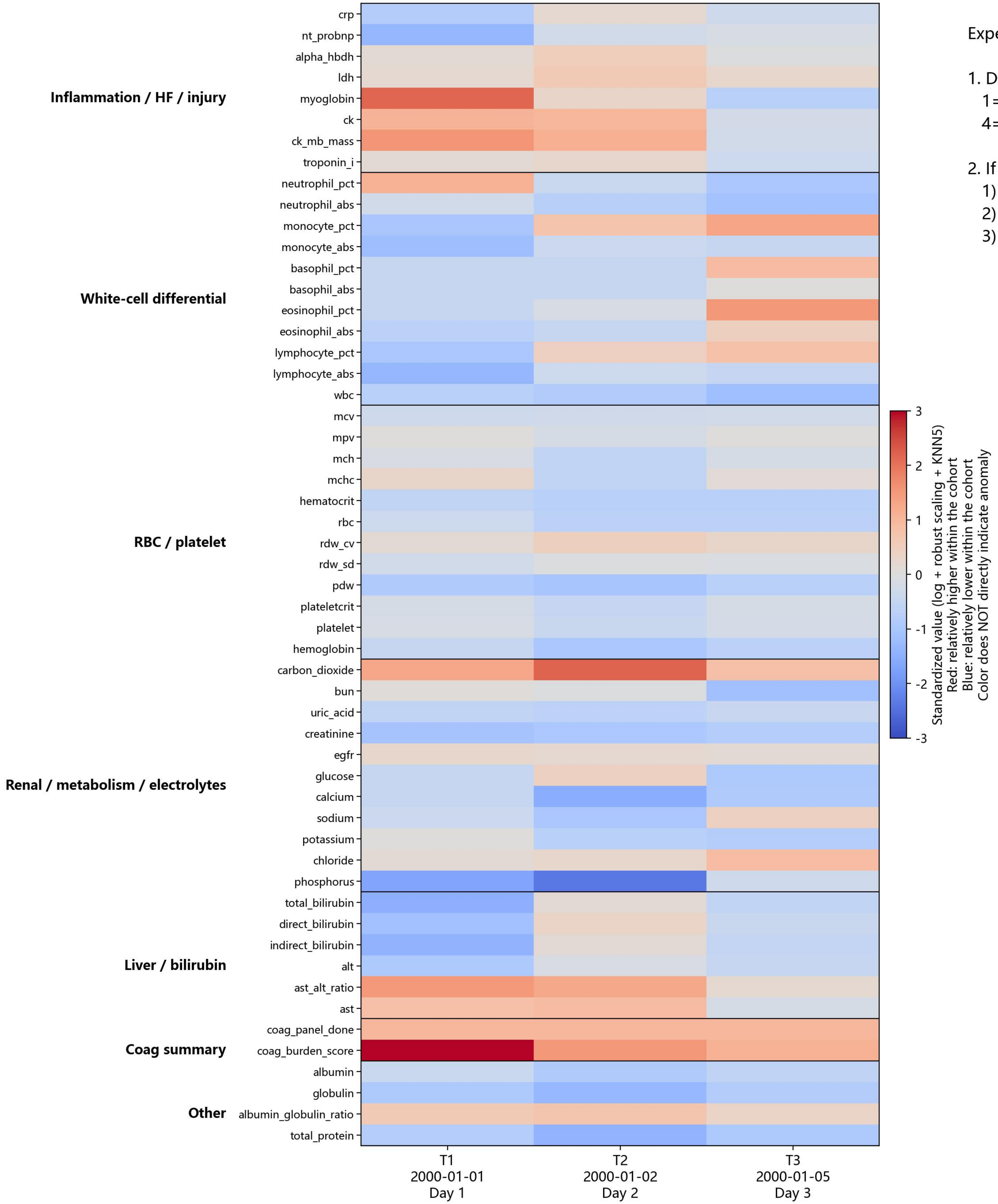

Expert review (blinded; no model score shown)

1. Degree of anomaly for this 3-point window (1-5):
   1=very typical; 2=relatively typical; 3=gray zone;
   4=relatively abnormal; 5=very abnormal

2. If scored 4-5, list the 3 most abnormal / noteworthy variables:
   1) ________________________
   2) ________________________
   3) ________________________

Patient-window heatmap card for blinded expert review
ID: P058 Window: W01

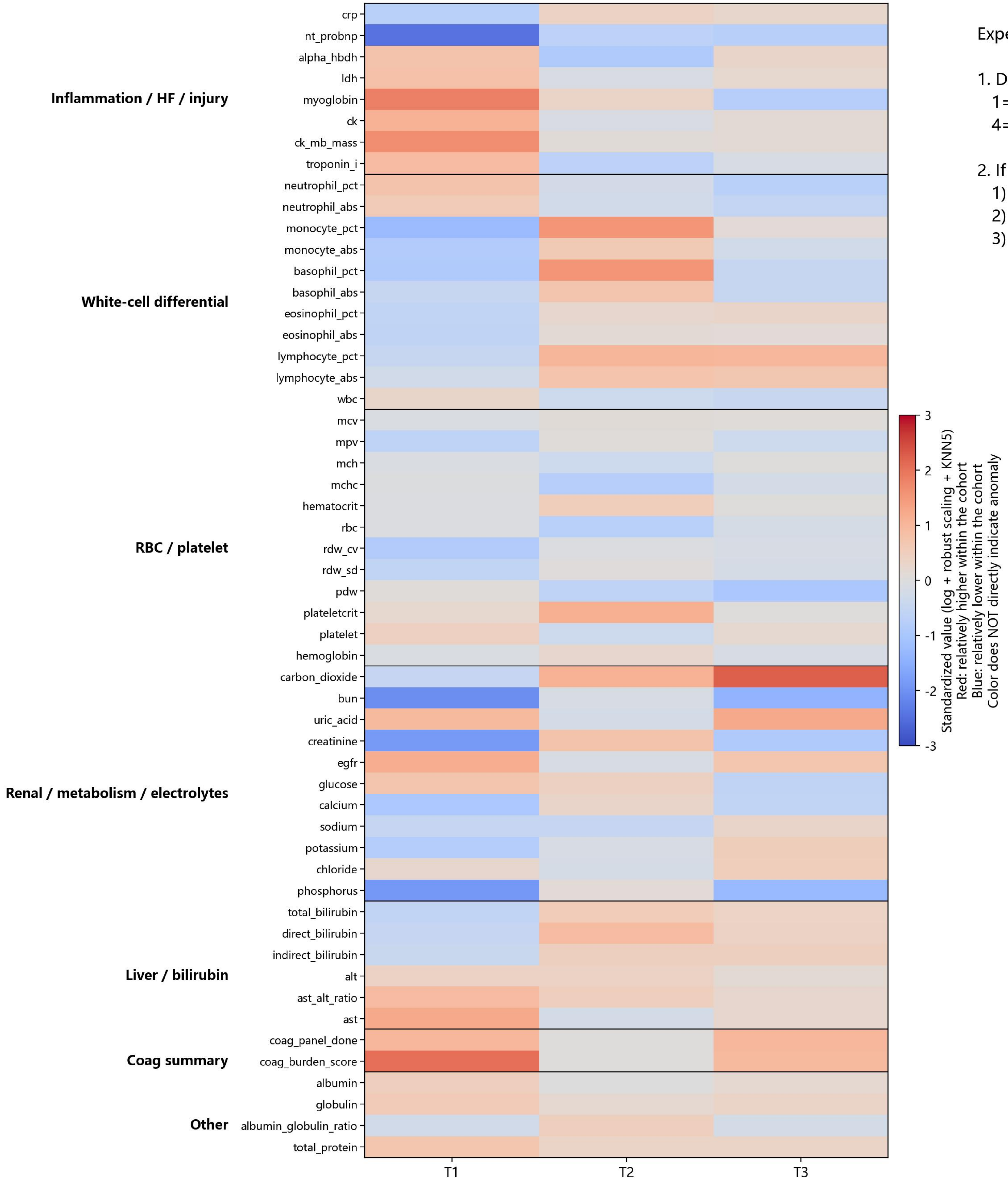

Expert review (blinded; no model score shown)

1. Degree of anomaly for this 3-point window (1-5):
   1=very typical; 2=relatively typical; 3=gray zone;
   4=relatively abnormal; 5=very abnormal

2. If scored 4-5, list the 3 most abnormal / noteworthy variables:
   1) ________________________
   2) ________________________
   3) ________________________

Patient-window heatmap card for blinded expert review
ID: P059   Window: W01

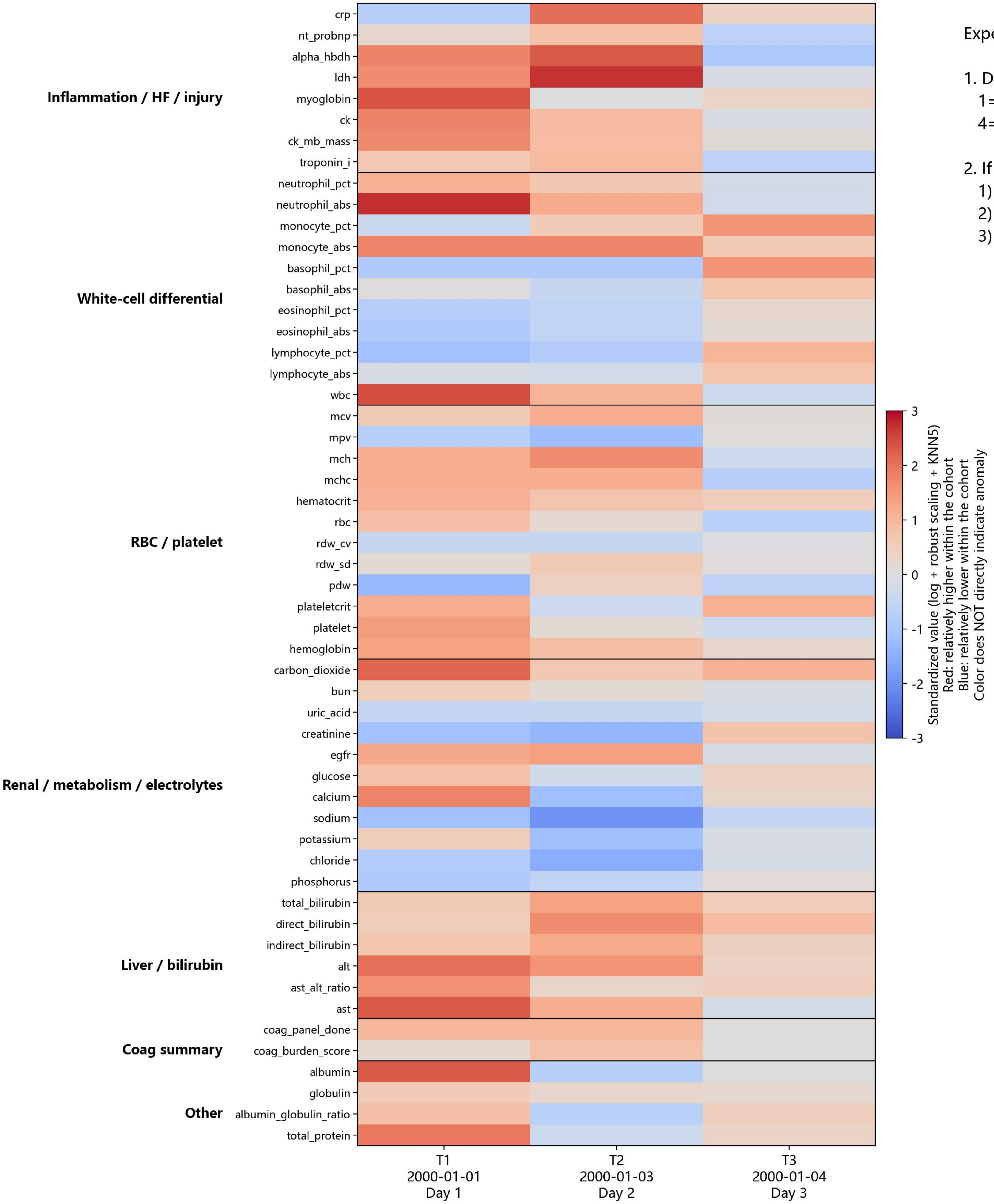

Expert review (blinded; no model score shown)

1. Degree of anomaly for this 3-point window (1-5):
   1=very typical; 2=relatively typical; 3=gray zone;
   4=relatively abnormal; 5=very abnormal

2. If scored 4-5, list the 3 most abnormal / noteworthy variables:
   1) ____________________
   2) ____________________
   3) ____________________

Patient-window heatmap card for blinded expert review
ID: P060   Window: W01

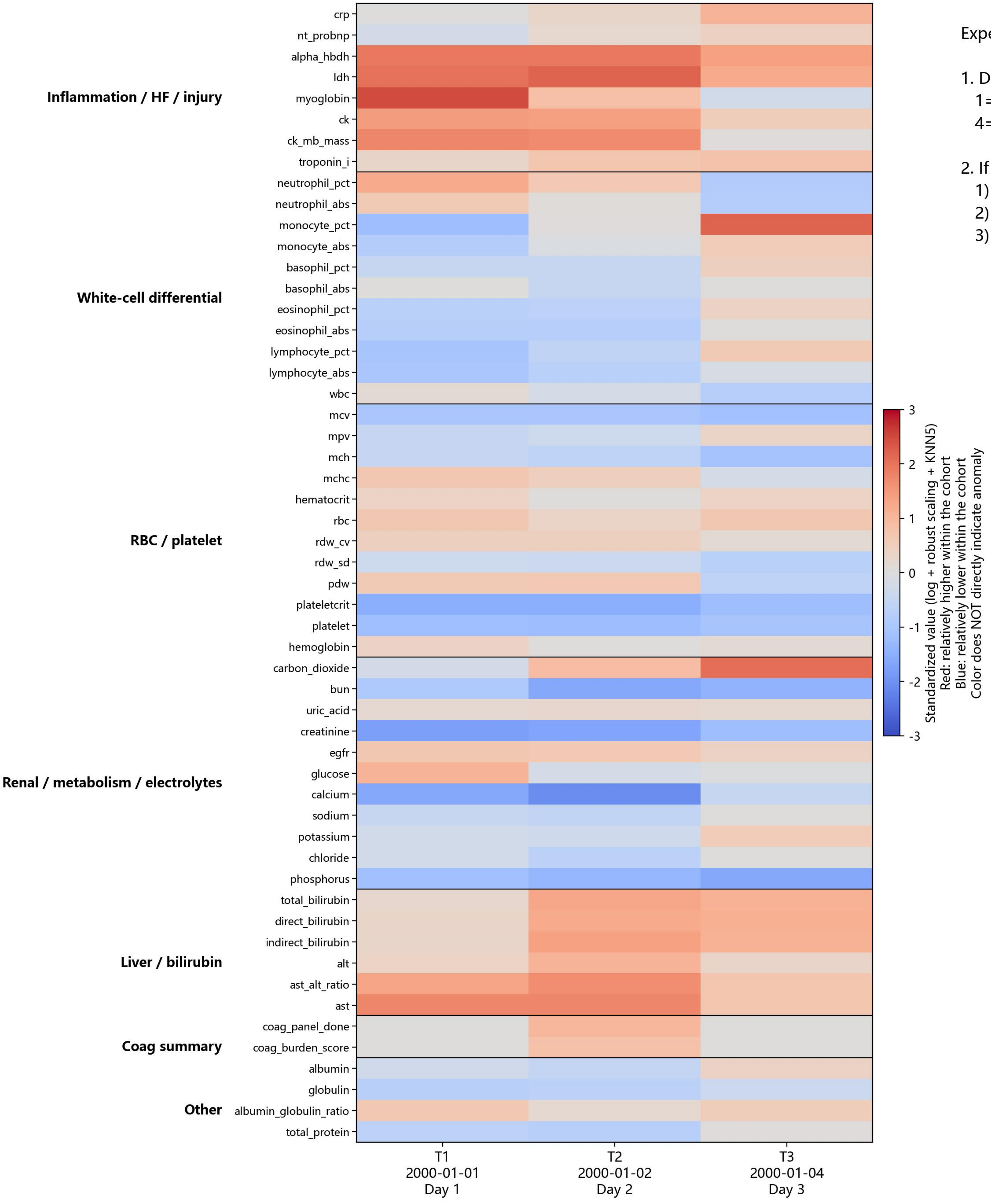

Expert review (blinded; no model score shown)

1. Degree of anomaly for this 3-point window (1-5):
1=very typical; 2=relatively typical; 3=gray zone;
4=relatively abnormal; 5=very abnormal

2. If scored 4-5, list the 3 most abnormal / noteworthy variables:
1) ___________________________
2) ___________________________
3) ___________________________

Patient-window heatmap card for blinded expert review
ID: P061   Window: W01

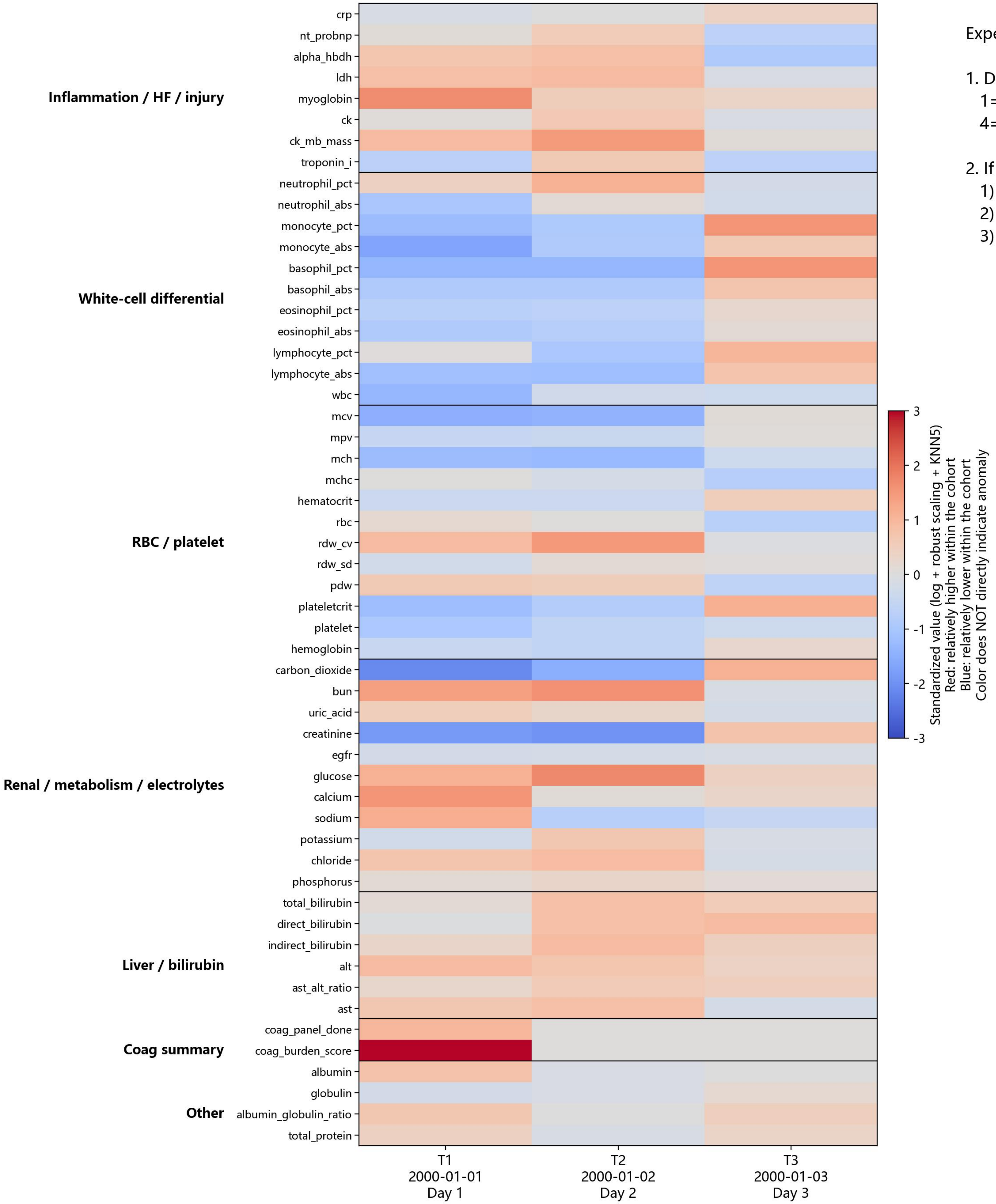

Expert review (blinded; no model score shown)

1. Degree of anomaly for this 3-point window (1-5):
   1=very typical; 2=relatively typical; 3=gray zone;
   4=relatively abnormal; 5=very abnormal

2. If scored 4-5, list the 3 most abnormal / noteworthy variables:
   1) ________________________
   2) ________________________
   3) ________________________

Patient-window heatmap card for blinded expert review
ID: P062   Window: W01

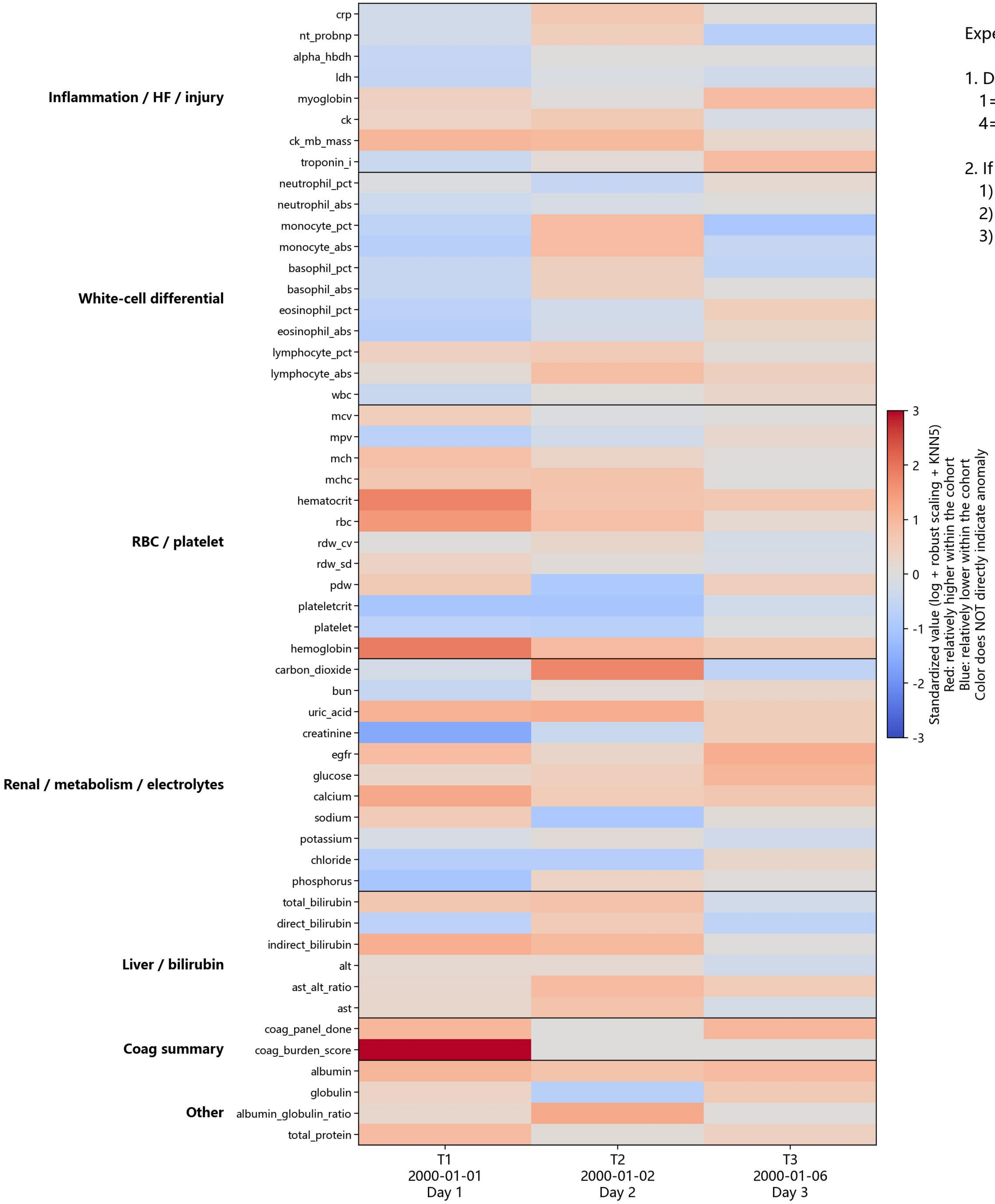

Expert review (blinded; no model score shown)

1. Degree of anomaly for this 3-point window (1-5):
   1=very typical; 2=relatively typical; 3=gray zone;
   4=relatively abnormal; 5=very abnormal

2. If scored 4-5, list the 3 most abnormal / noteworthy variables:
   1) ________________________
   2) ________________________
   3) ________________________

Patient-window heatmap card for blinded expert review
ID: P063   Window: W01

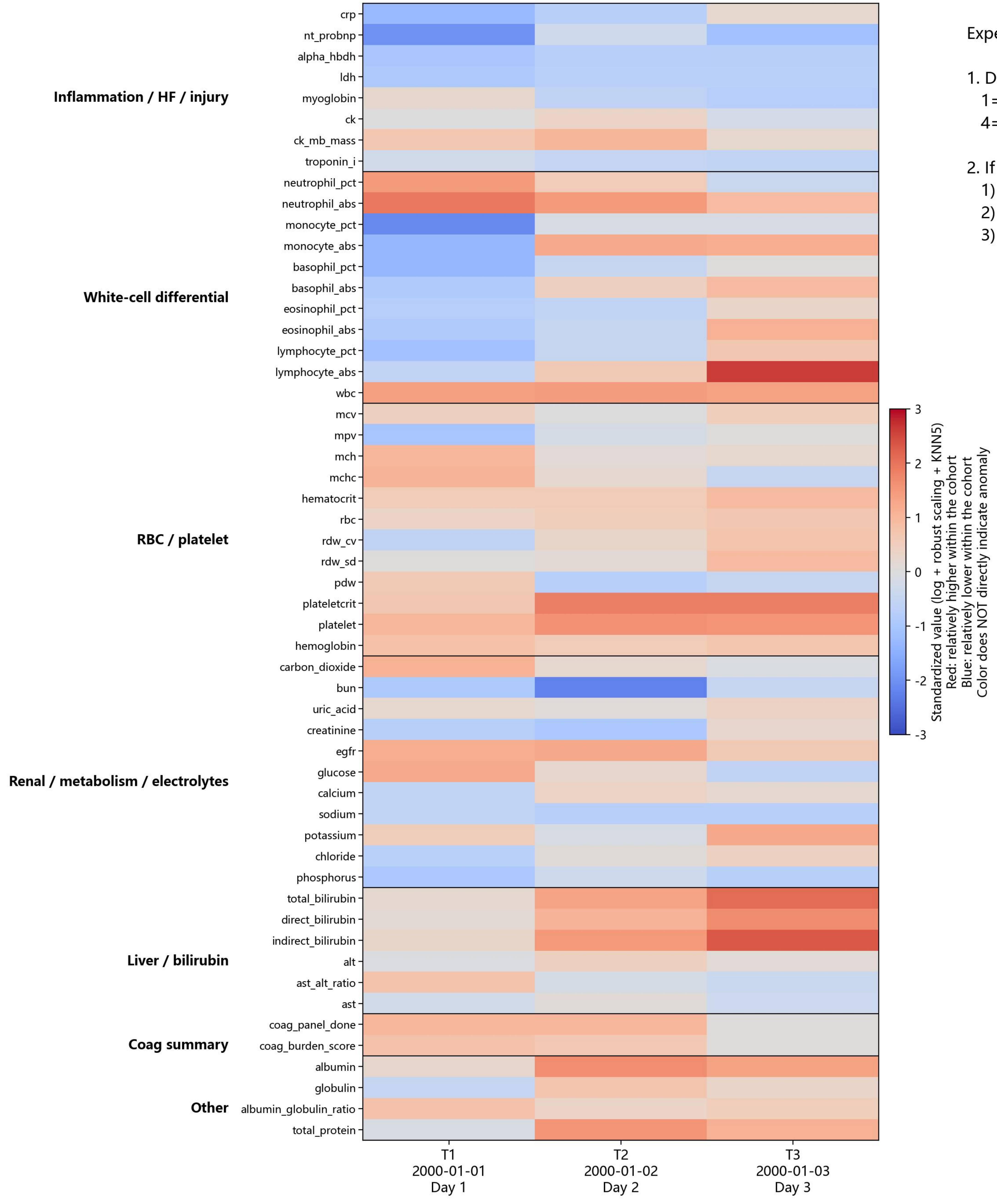

Expert review (blinded; no model score shown)

1. Degree of anomaly for this 3-point window (1-5):
   1=very typical; 2=relatively typical; 3=gray zone;
   4=relatively abnormal; 5=very abnormal

2. If scored 4-5, list the 3 most abnormal / noteworthy variables:
   1) ________________________
   2) ________________________
   3) ________________________

Patient-window heatmap card for blinded expert review
ID: P064   Window: W01

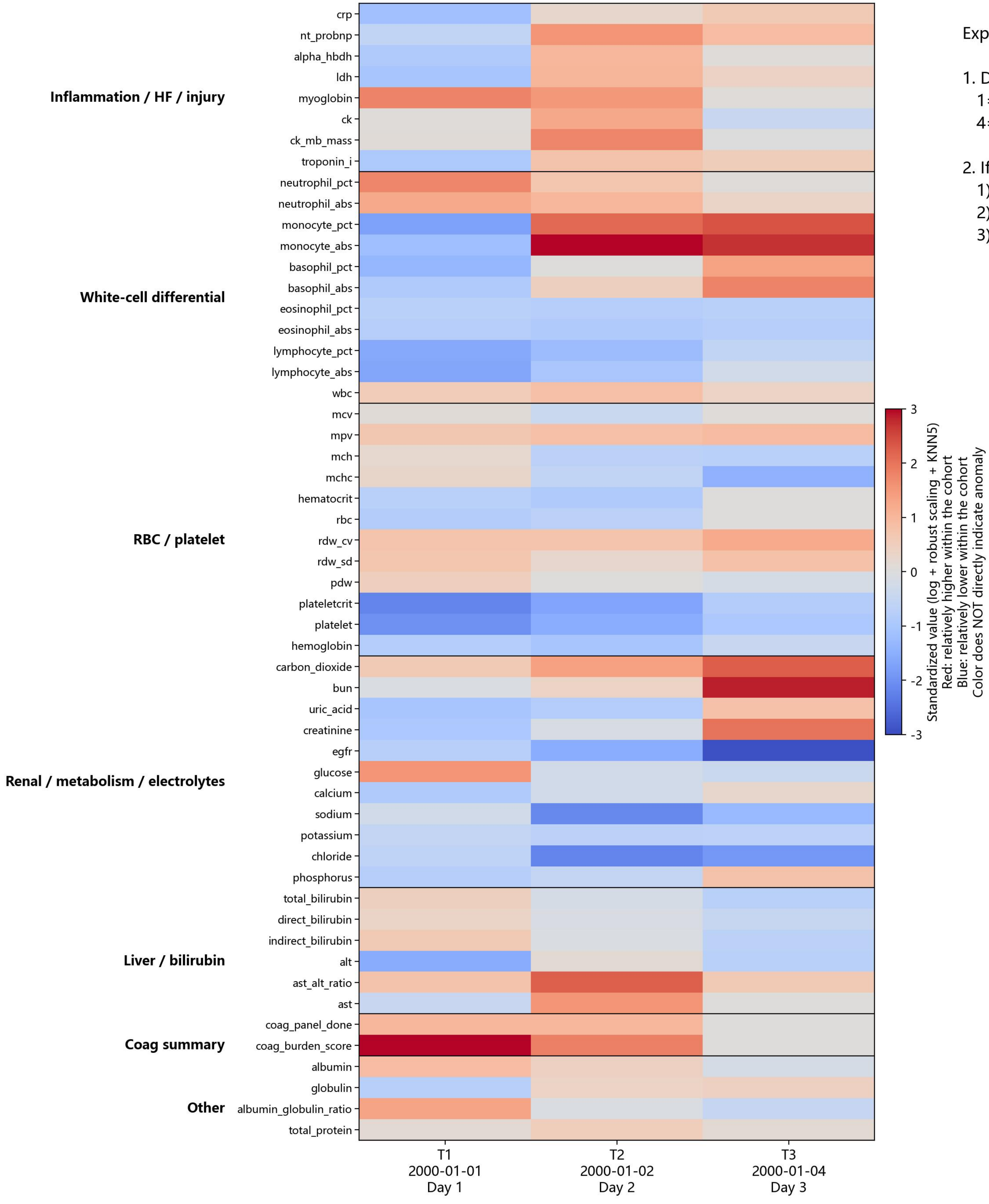

Expert review (blinded; no model score shown)

1. Degree of anomaly for this 3-point window (1-5):
   1=very typical; 2=relatively typical; 3=gray zone;
   4=relatively abnormal; 5=very abnormal

2. If scored 4-5, list the 3 most abnormal / noteworthy variables:
   1) ________________________
   2) ________________________
   3) ________________________

Patient-window heatmap card for blinded expert review
ID: P065   Window: W01

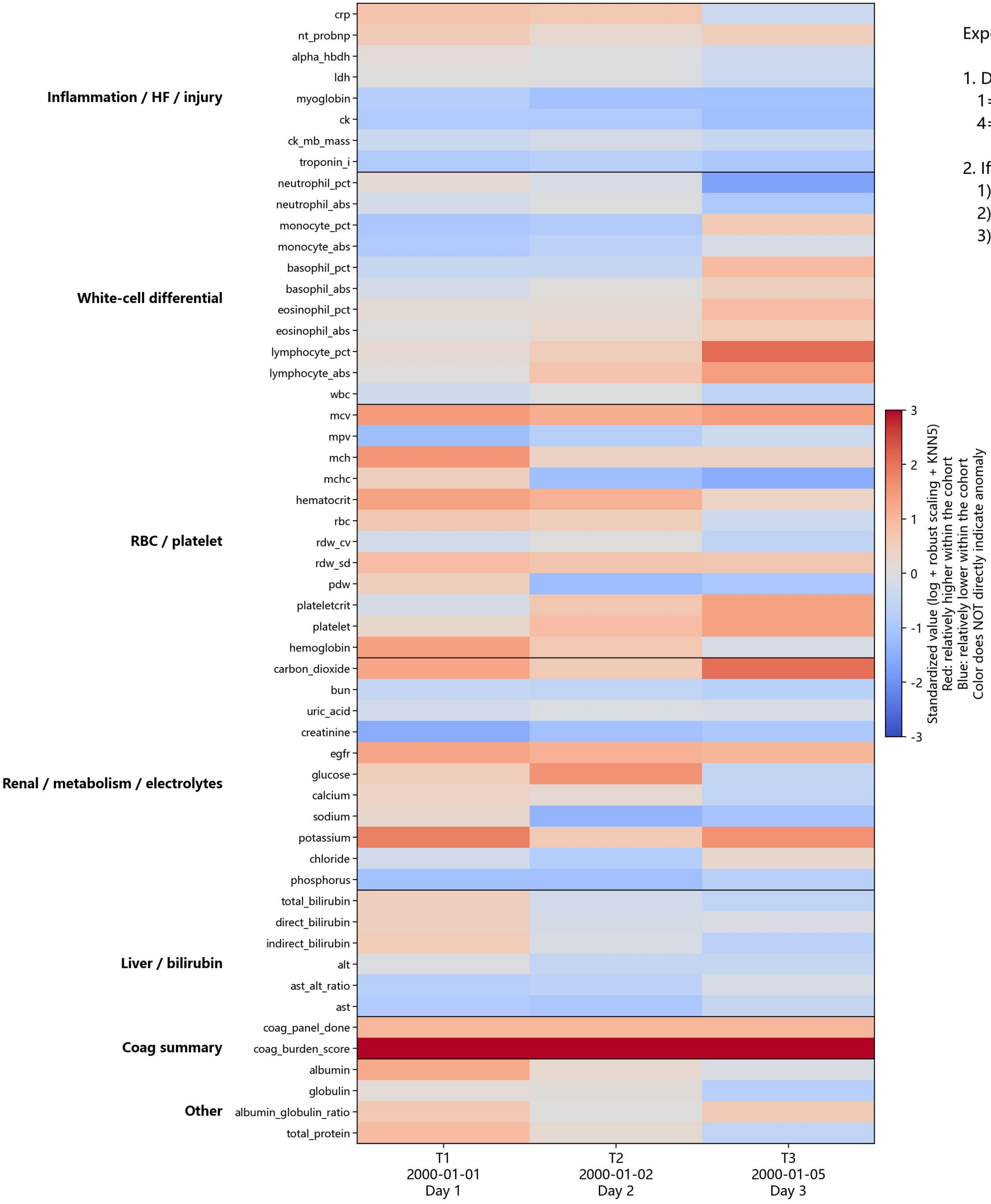

Expert review (blinded; no model score shown)

1. Degree of anomaly for this 3-point window (1-5):
   1=very typical; 2=relatively typical; 3=gray zone;
   4=relatively abnormal; 5=very abnormal

2. If scored 4-5, list the 3 most abnormal / noteworthy variables:
   1) ________________________
   2) ________________________
   3) ________________________

Patient-window heatmap card for blinded expert review
ID: P066 Window: W01

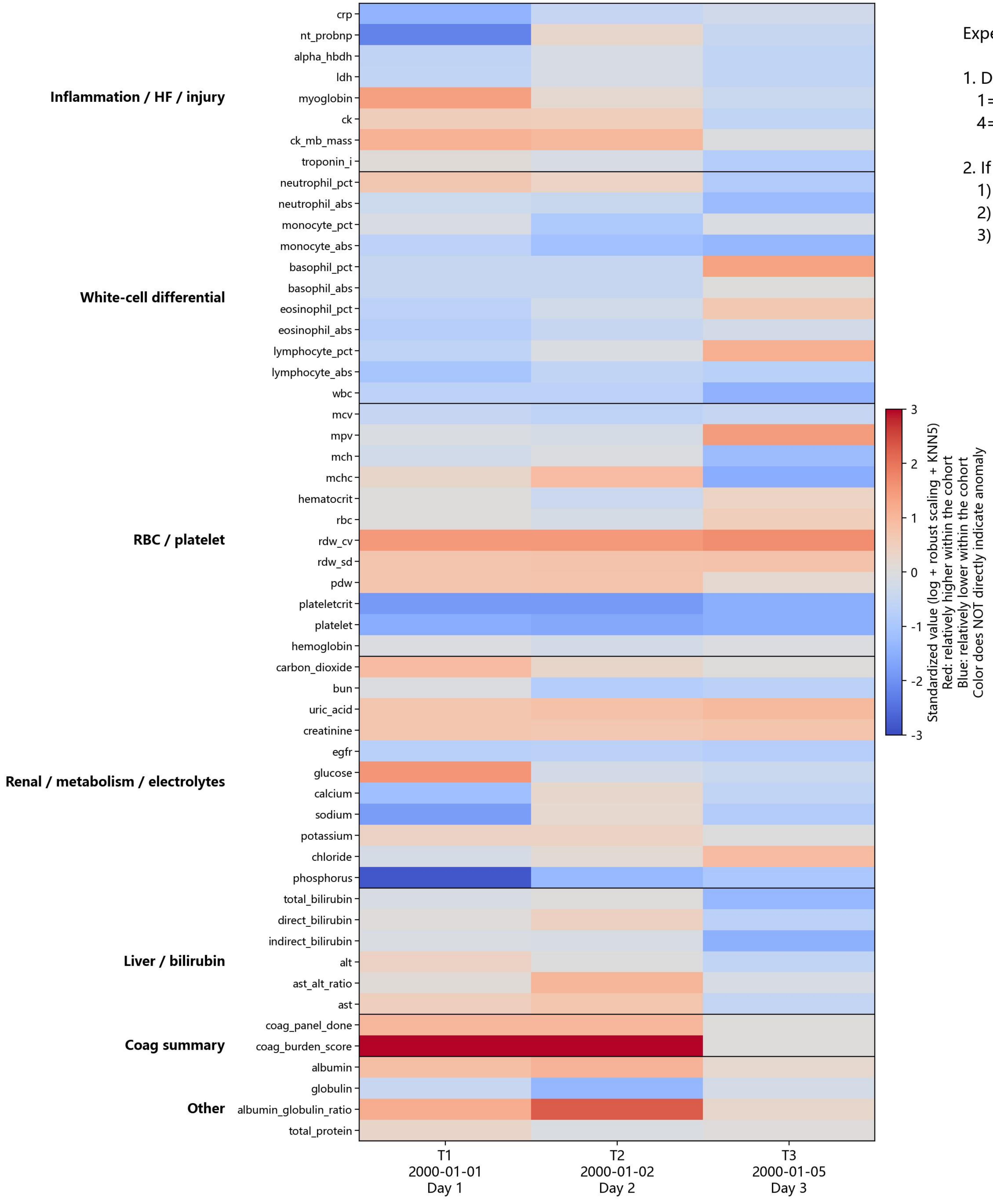

Expert review (blinded; no model score shown)

1. Degree of anomaly for this 3-point window (1-5):
   1=very typical; 2=relatively typical; 3=gray zone;
   4=relatively abnormal; 5=very abnormal

2. If scored 4-5, list the 3 most abnormal / noteworthy variables:
   1) ________________________
   2) ________________________
   3) ________________________

Patient-window heatmap card for blinded expert review
ID: P067   Window: W01

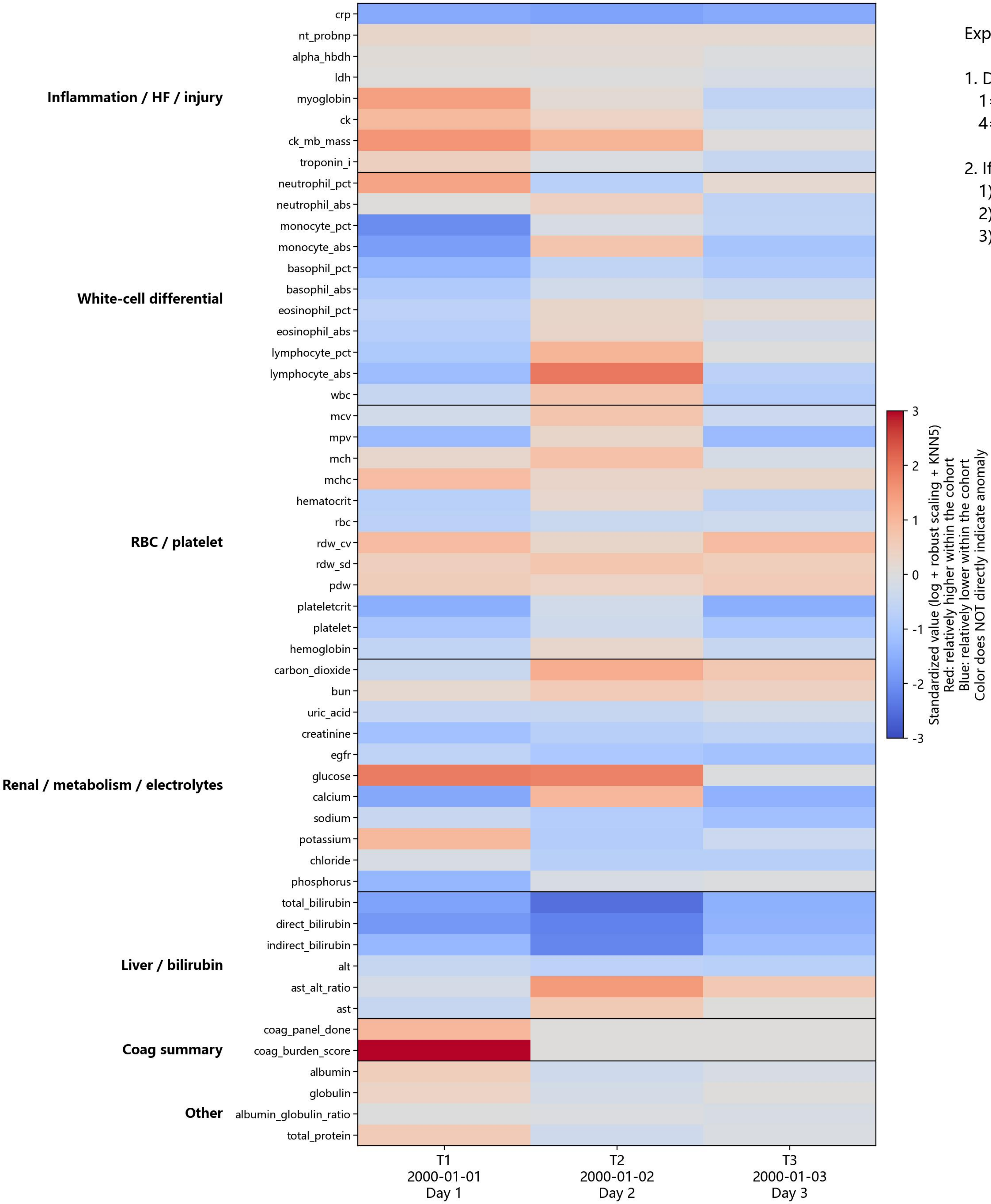

Expert review (blinded; no model score shown)

1. Degree of anomaly for this 3-point window (1-5):
   1=very typical; 2=relatively typical; 3=gray zone;
   4=relatively abnormal; 5=very abnormal

2. If scored 4-5, list the 3 most abnormal / noteworthy variables:
   1) ________________________
   2) ________________________
   3) ________________________

Patient-window heatmap card for blinded expert review
ID: P068   Window: W01

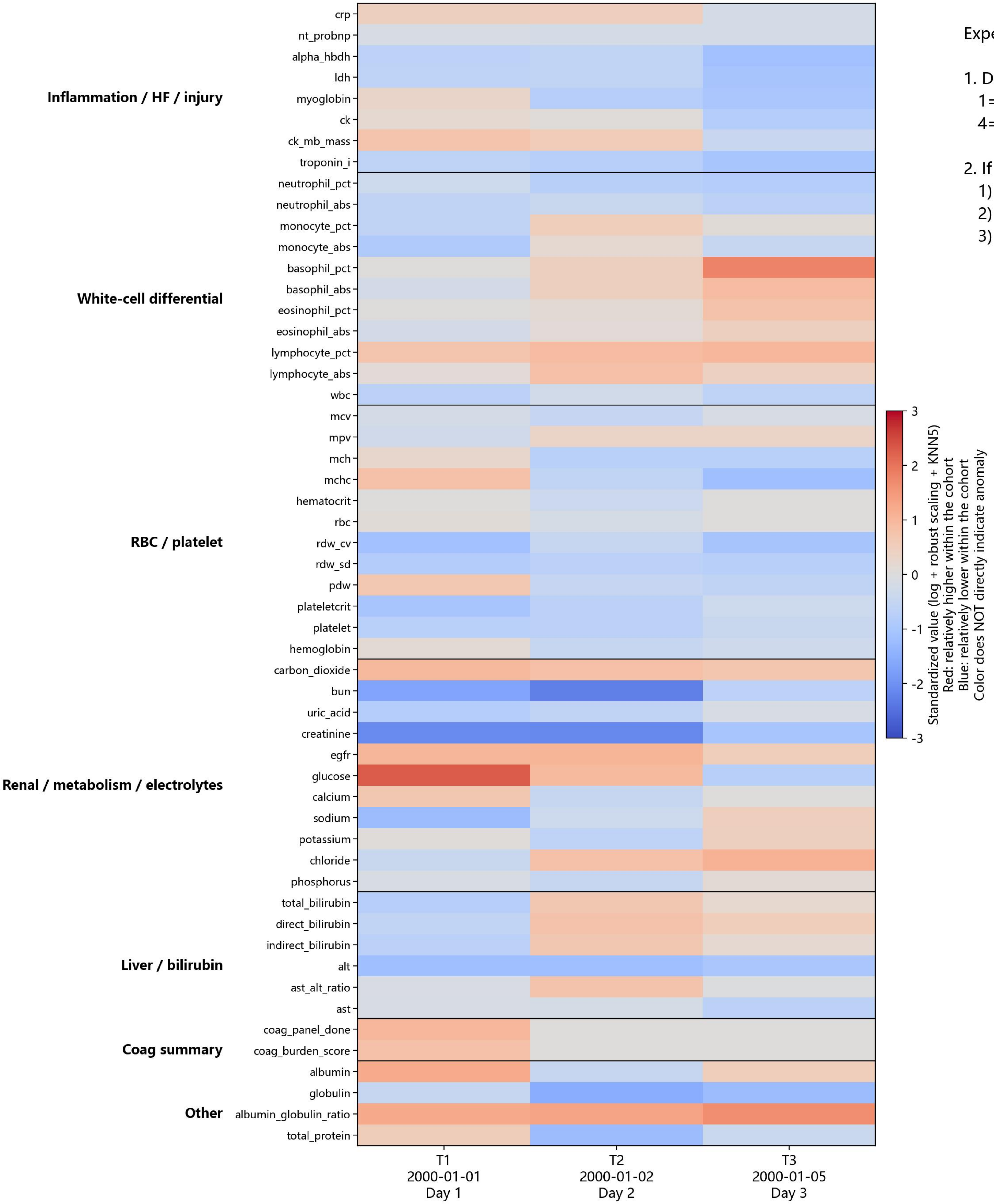

Expert review (blinded; no model score shown)

1. Degree of anomaly for this 3-point window (1-5):
   1=very typical; 2=relatively typical; 3=gray zone;
   4=relatively abnormal; 5=very abnormal

2. If scored 4-5, list the 3 most abnormal / noteworthy variables:
   1) ________________________
   2) ________________________
   3) ________________________

Patient-window heatmap card for blinded expert review
ID: P069 Window: W01

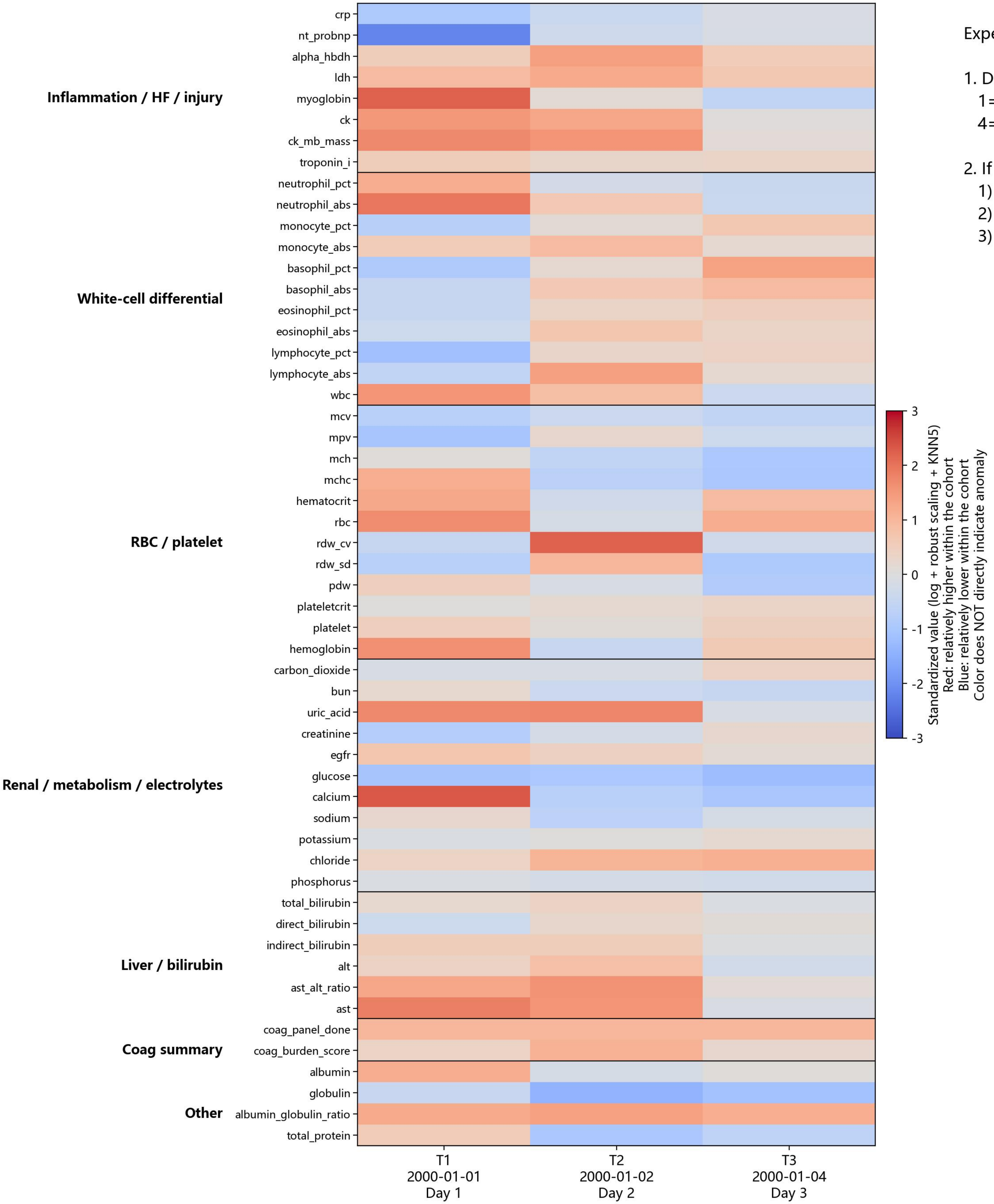

Expert review (blinded; no model score shown)

1. Degree of anomaly for this 3-point window (1-5):
   1=very typical; 2=relatively typical; 3=gray zone;
   4=relatively abnormal; 5=very abnormal

2. If scored 4-5, list the 3 most abnormal / noteworthy variables:
   1) ____________________________
   2) ____________________________
   3) ____________________________

Patient-window heatmap card for blinded expert review
ID: P070   Window: W01

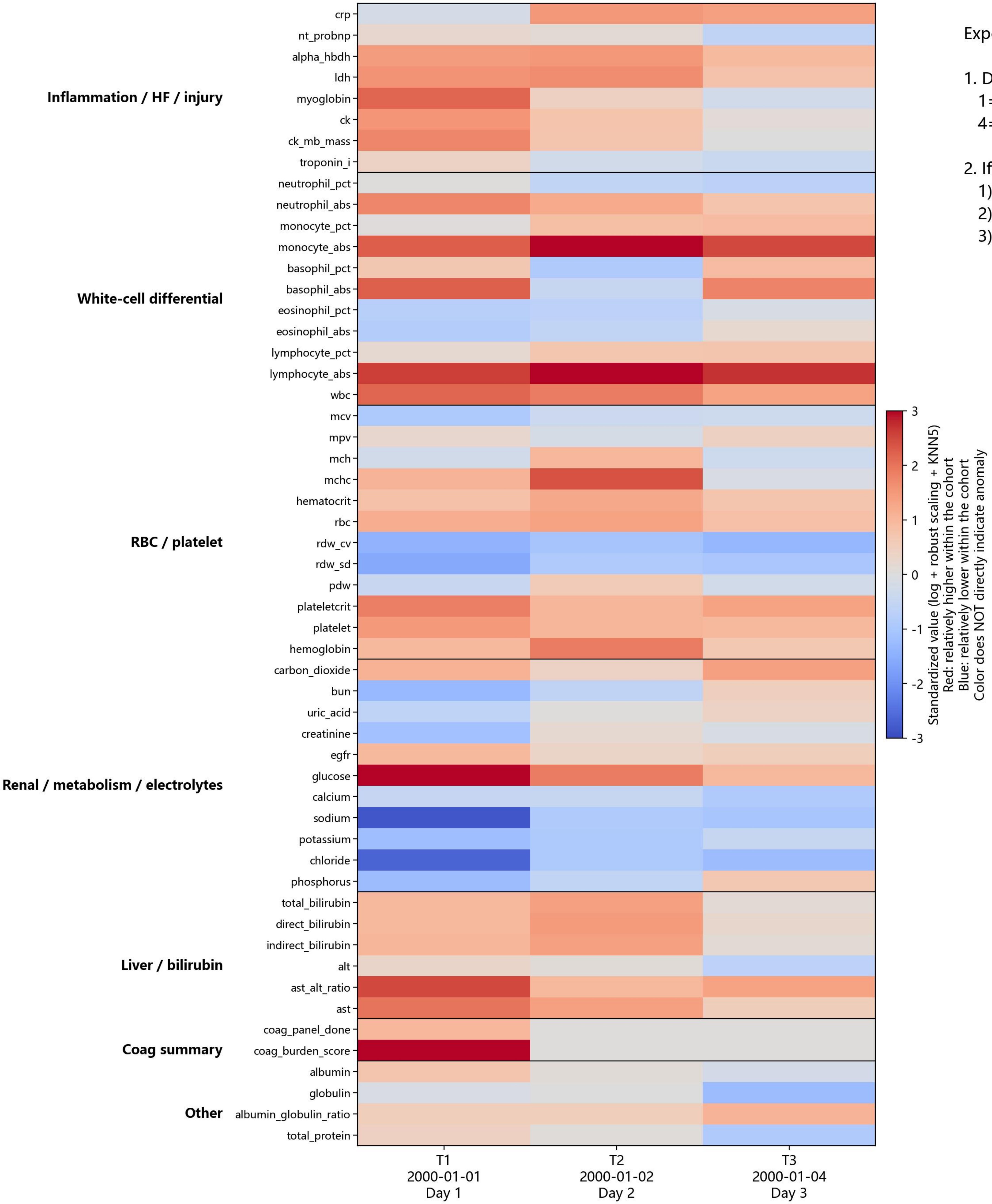

Expert review (blinded; no model score shown)

1. Degree of anomaly for this 3-point window (1-5):
   1=very typical; 2=relatively typical; 3=gray zone;
   4=relatively abnormal; 5=very abnormal

2. If scored 4-5, list the 3 most abnormal / noteworthy variables:
   1) ________________________
   2) ________________________
   3) ________________________

Patient-window heatmap card for blinded expert review
ID: P071   Window: W01

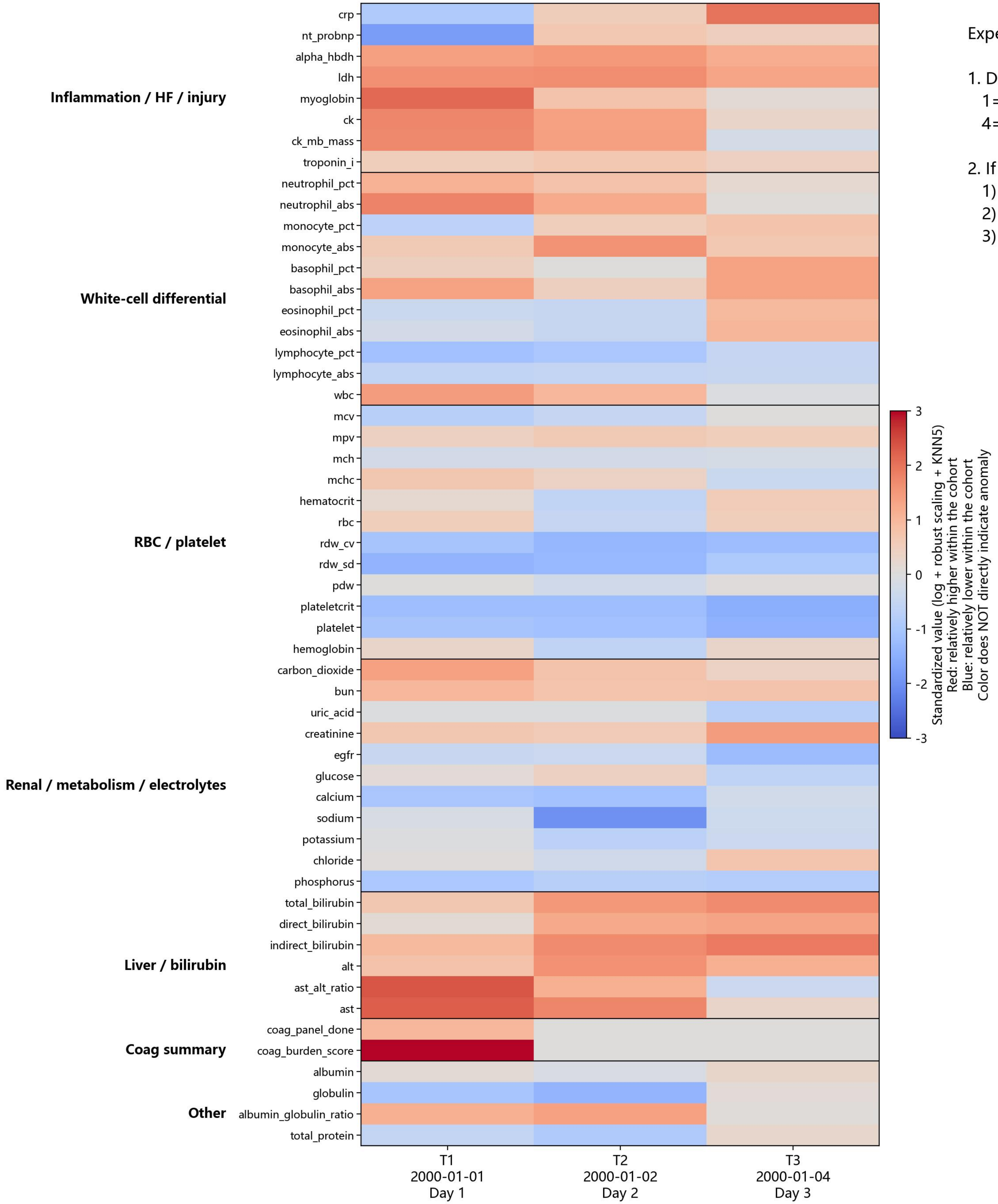

Expert review (blinded; no model score shown)

1. Degree of anomaly for this 3-point window (1-5):
   1=very typical; 2=relatively typical; 3=gray zone;
   4=relatively abnormal; 5=very abnormal

2. If scored 4-5, list the 3 most abnormal / noteworthy variables:
   1) ________________________
   2) ________________________
   3) ________________________

Patient-window heatmap card for blinded expert review
ID: P072 Window: W01

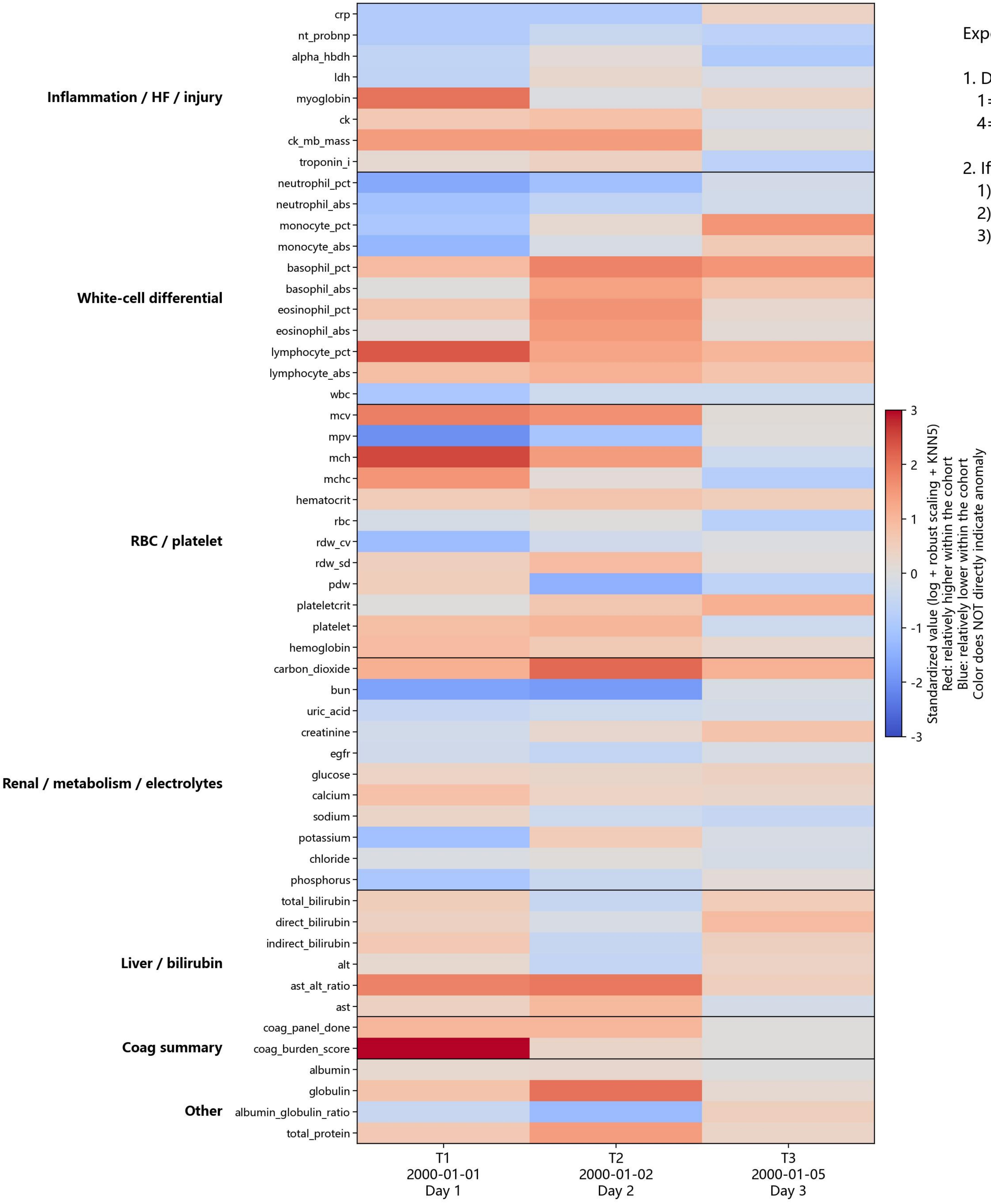

Expert review (blinded; no model score shown)

1. Degree of anomaly for this 3-point window (1-5):
   1=very typical; 2=relatively typical; 3=gray zone;
   4=relatively abnormal; 5=very abnormal

2. If scored 4-5, list the 3 most abnormal / noteworthy variables:
   1) ________________________
   2) ________________________
   3) ________________________

Patient-window heatmap card for blinded expert review
ID: P073   Window: W01

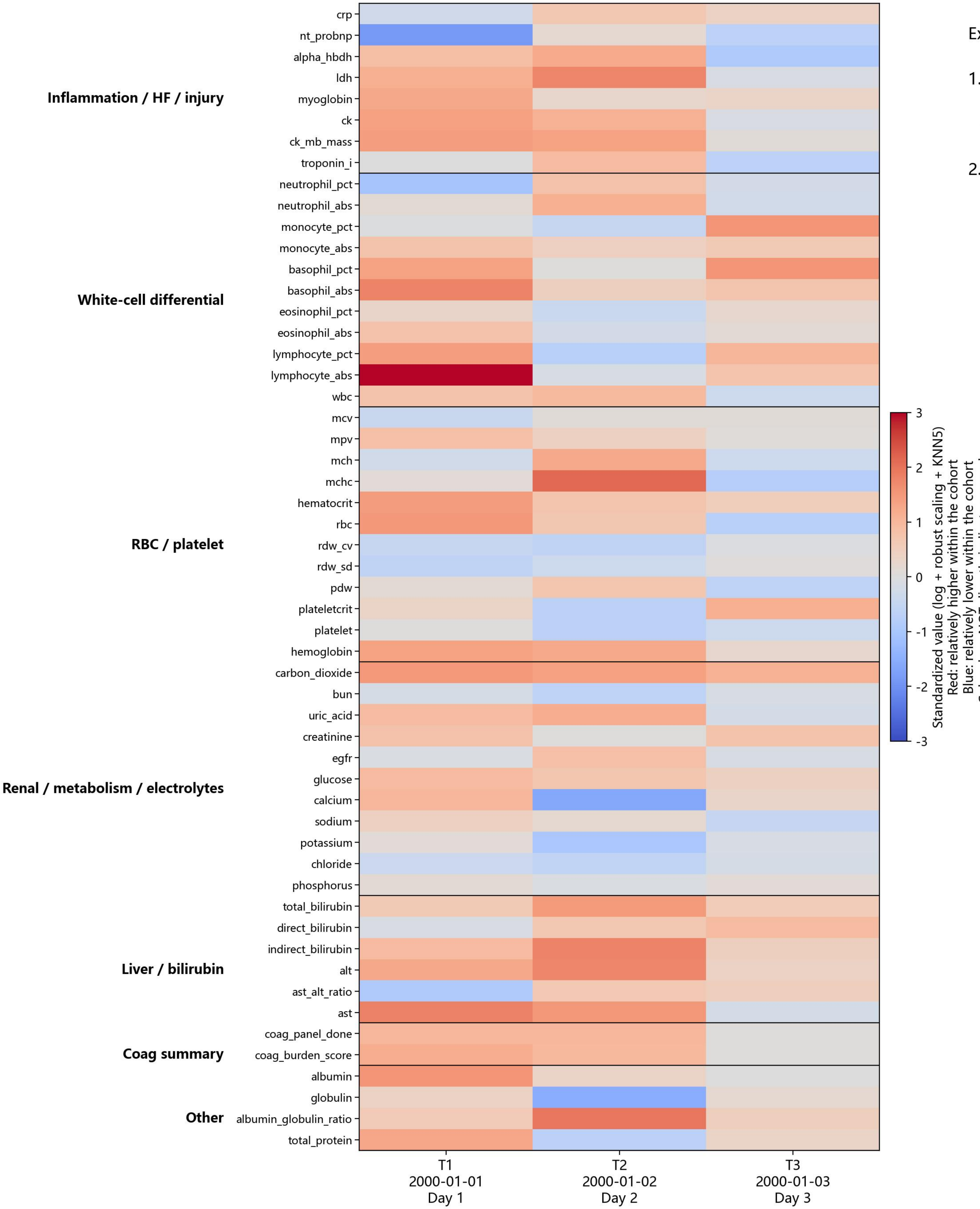

Expert review (blinded; no model score shown)

1. Degree of anomaly for this 3-point window (1-5):
   1=very typical; 2=relatively typical; 3=gray zone;
   4=relatively abnormal; 5=very abnormal

2. If scored 4-5, list the 3 most abnormal / noteworthy variables:
   1) ________________________
   2) ________________________
   3) ________________________

Patient-window heatmap card for blinded expert review
ID: P074   Window: W01

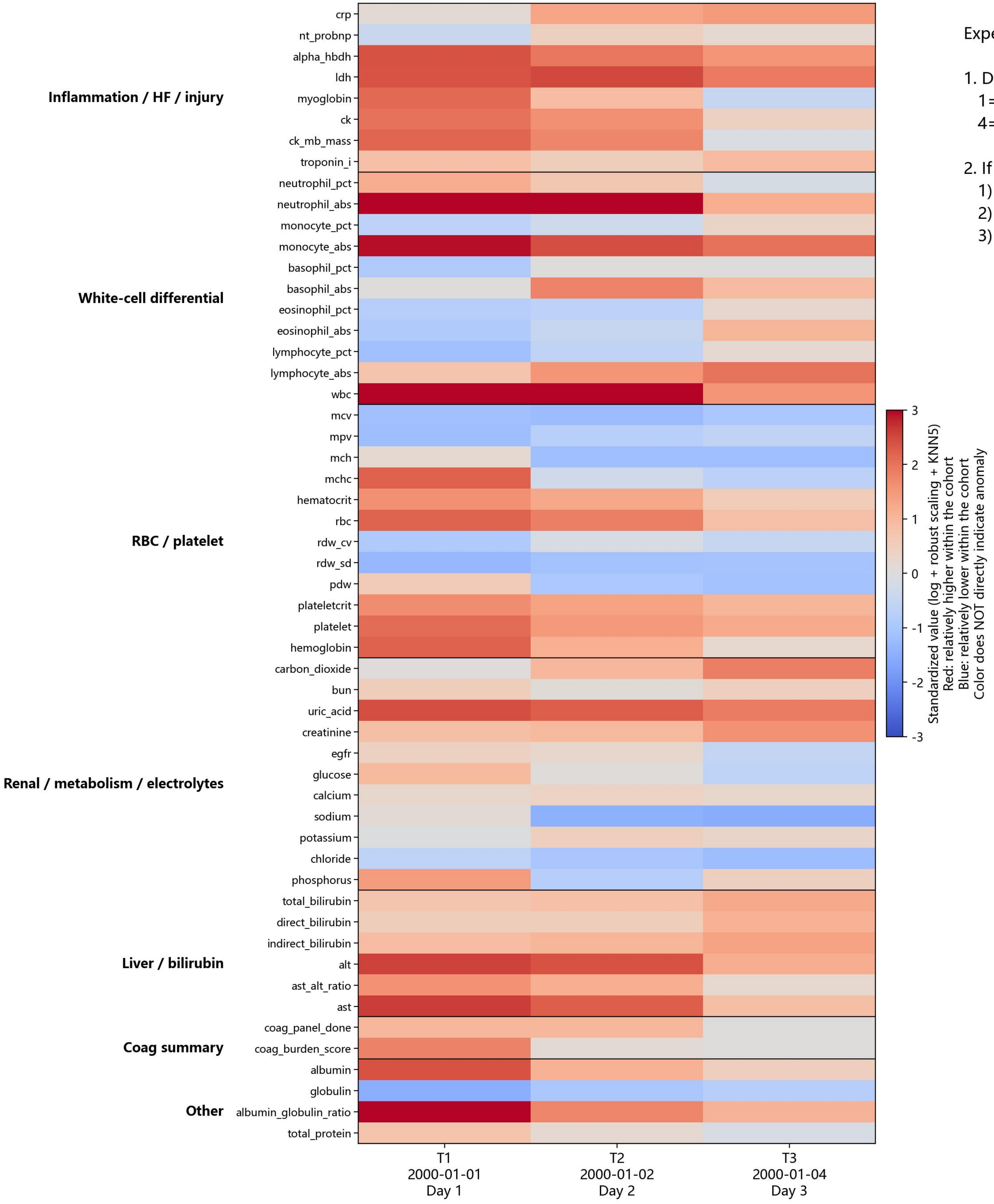

Expert review (blinded; no model score shown)

1. Degree of anomaly for this 3-point window (1-5):
   1=very typical; 2=relatively typical; 3=gray zone;
   4=relatively abnormal; 5=very abnormal

2. If scored 4-5, list the 3 most abnormal / noteworthy variables:
   1) ________________________
   2) ________________________
   3) ________________________

Patient-window heatmap card for blinded expert review
ID: P075   Window: W01

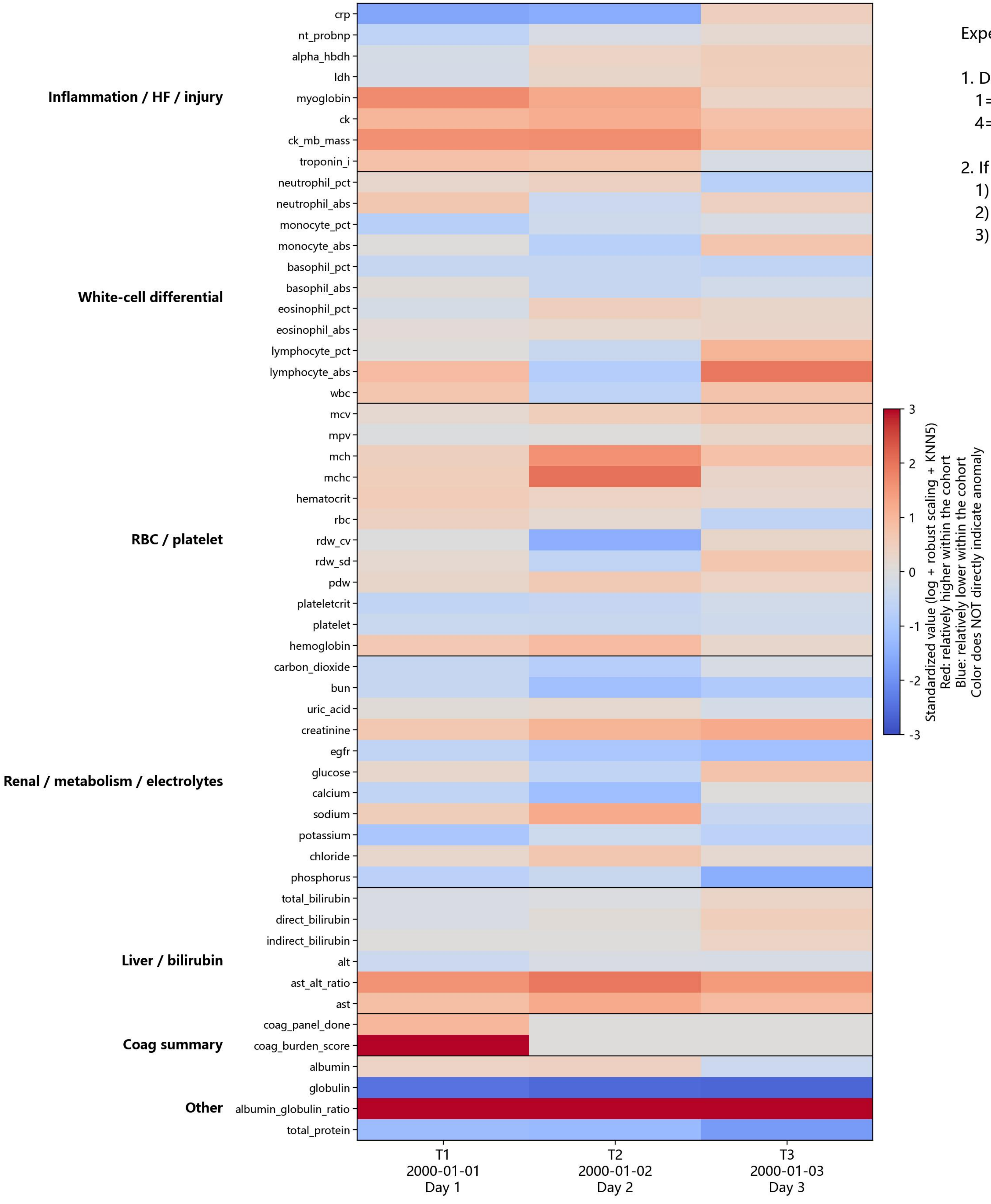

Expert review (blinded; no model score shown)

1. Degree of anomaly for this 3-point window (1-5):
   1=very typical; 2=relatively typical; 3=gray zone;
   4=relatively abnormal; 5=very abnormal

2. If scored 4-5, list the 3 most abnormal / noteworthy variables:
   1) ____________________
   2) ____________________
   3) ____________________

Patient-window heatmap card for blinded expert review
ID: P076   Window: W01

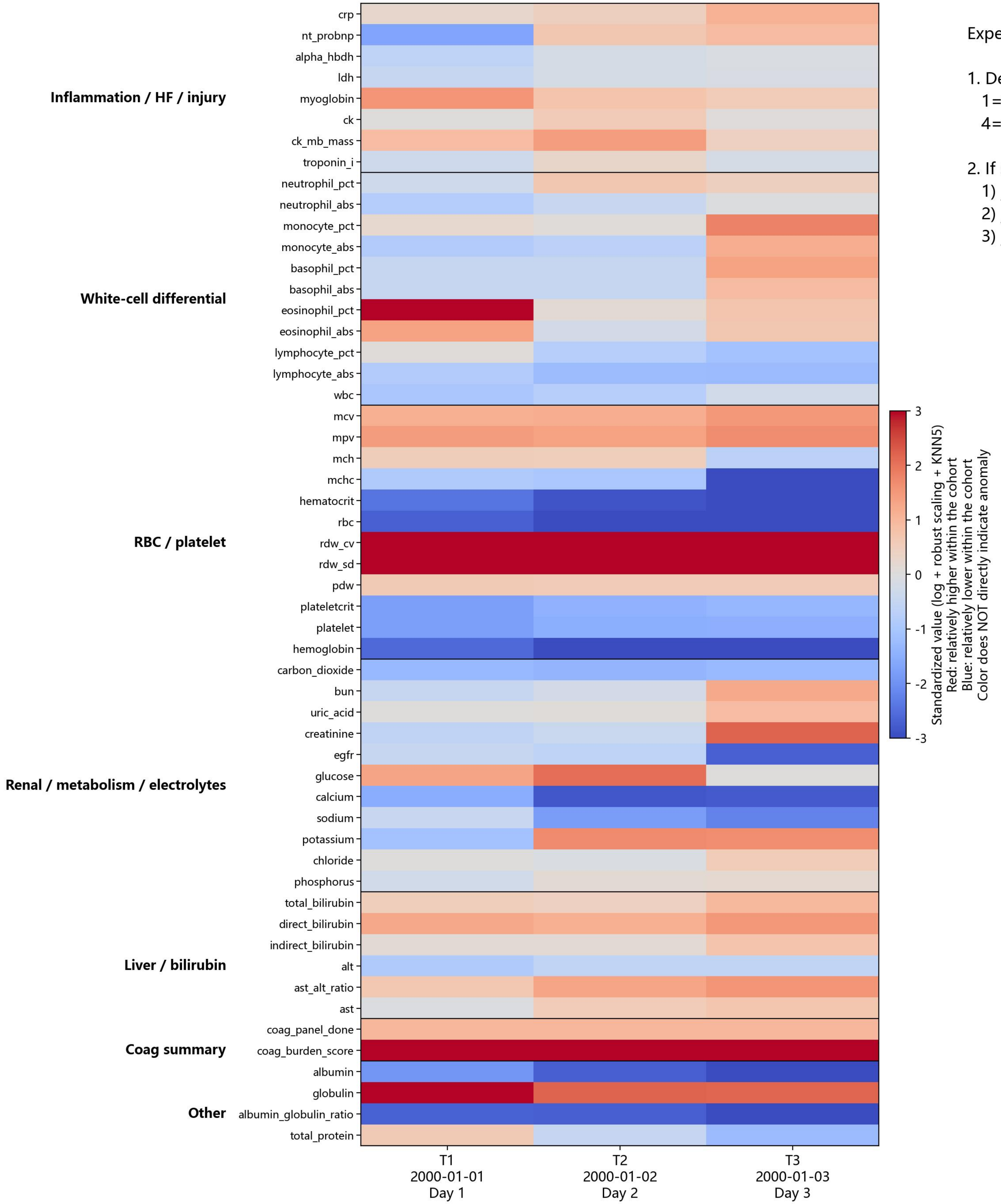

Expert review (blinded; no model score shown)

1. Degree of anomaly for this 3-point window (1-5):
   1=very typical; 2=relatively typical; 3=gray zone;
   4=relatively abnormal; 5=very abnormal

2. If scored 4-5, list the 3 most abnormal / noteworthy variables:
   1) ________________________
   2) ________________________
   3) ________________________

Patient-window heatmap card for blinded expert review
ID: P077   Window: W01

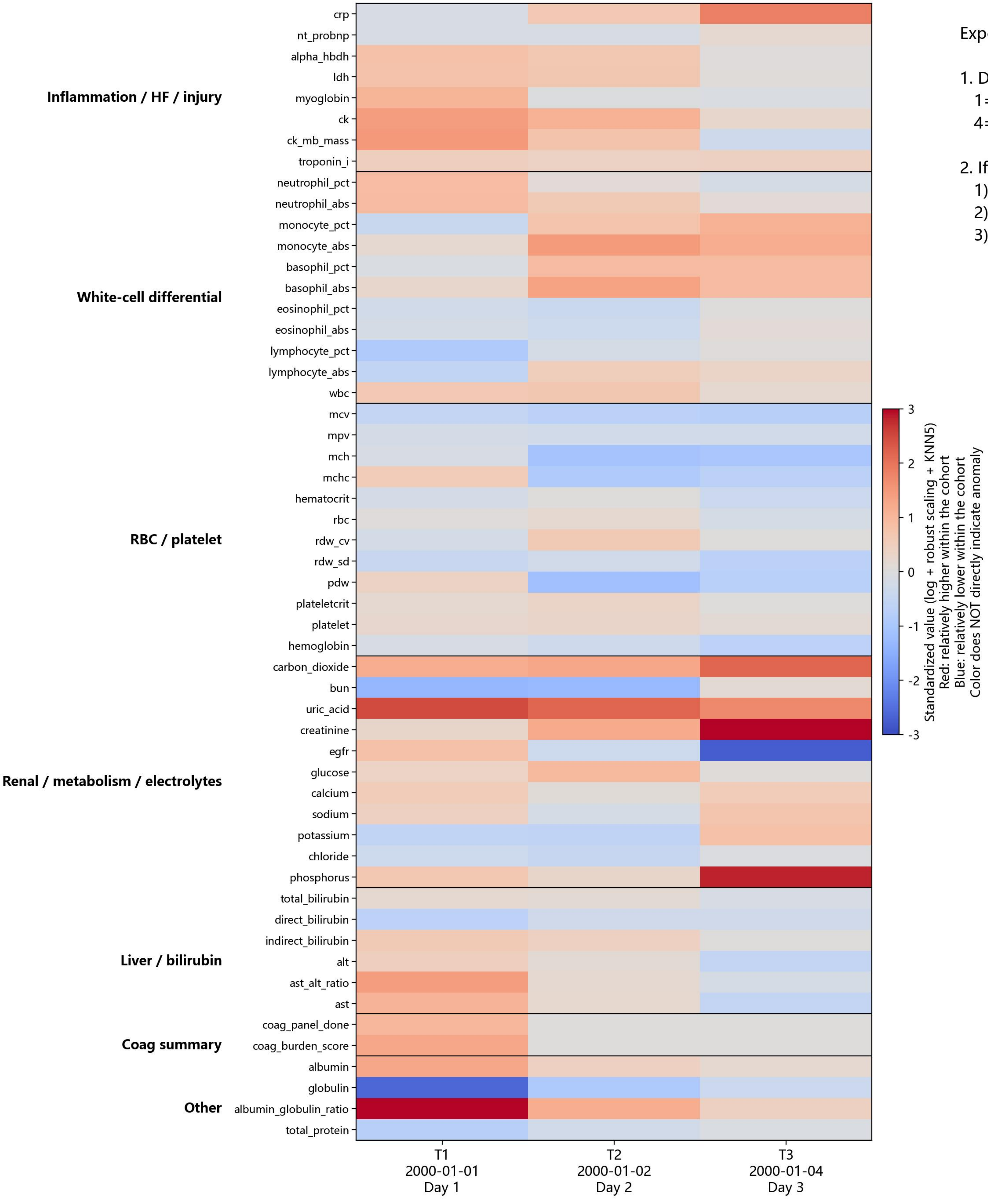

Expert review (blinded; no model score shown)

1. Degree of anomaly for this 3-point window (1-5):
   1=very typical; 2=relatively typical; 3=gray zone;
   4=relatively abnormal; 5=very abnormal

2. If scored 4-5, list the 3 most abnormal / noteworthy variables:
   1) ________________________
   2) ________________________
   3) ________________________

Patient-window heatmap card for blinded expert review
ID: P078   Window: W01

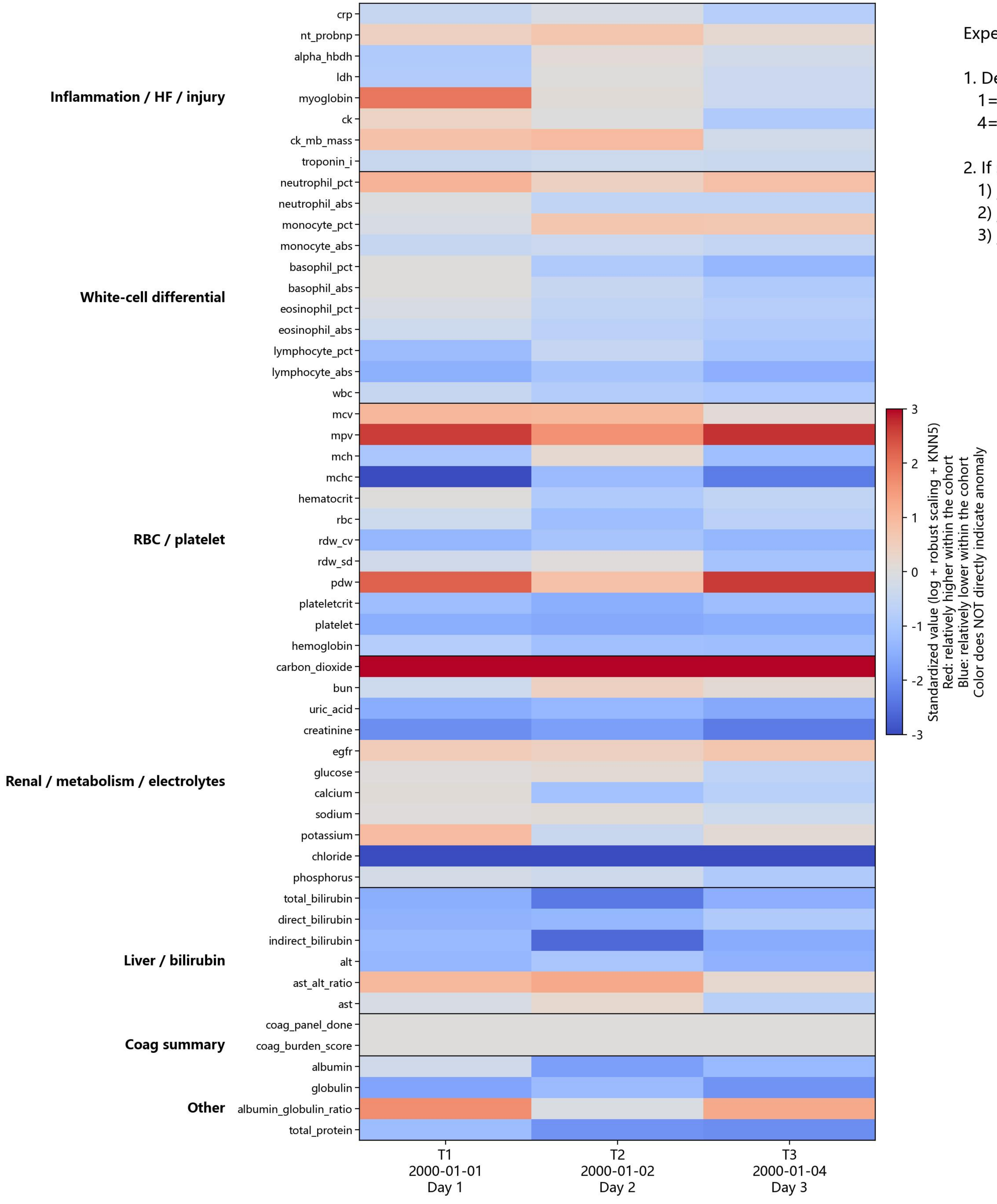

Expert review (blinded; no model score shown)

1. Degree of anomaly for this 3-point window (1-5):
   1=very typical; 2=relatively typical; 3=gray zone;
   4=relatively abnormal; 5=very abnormal

2. If scored 4-5, list the 3 most abnormal / noteworthy variables:
   1) ________________________
   2) ________________________
   3) ________________________

Patient-window heatmap card for blinded expert review
ID: P079 Window: W01

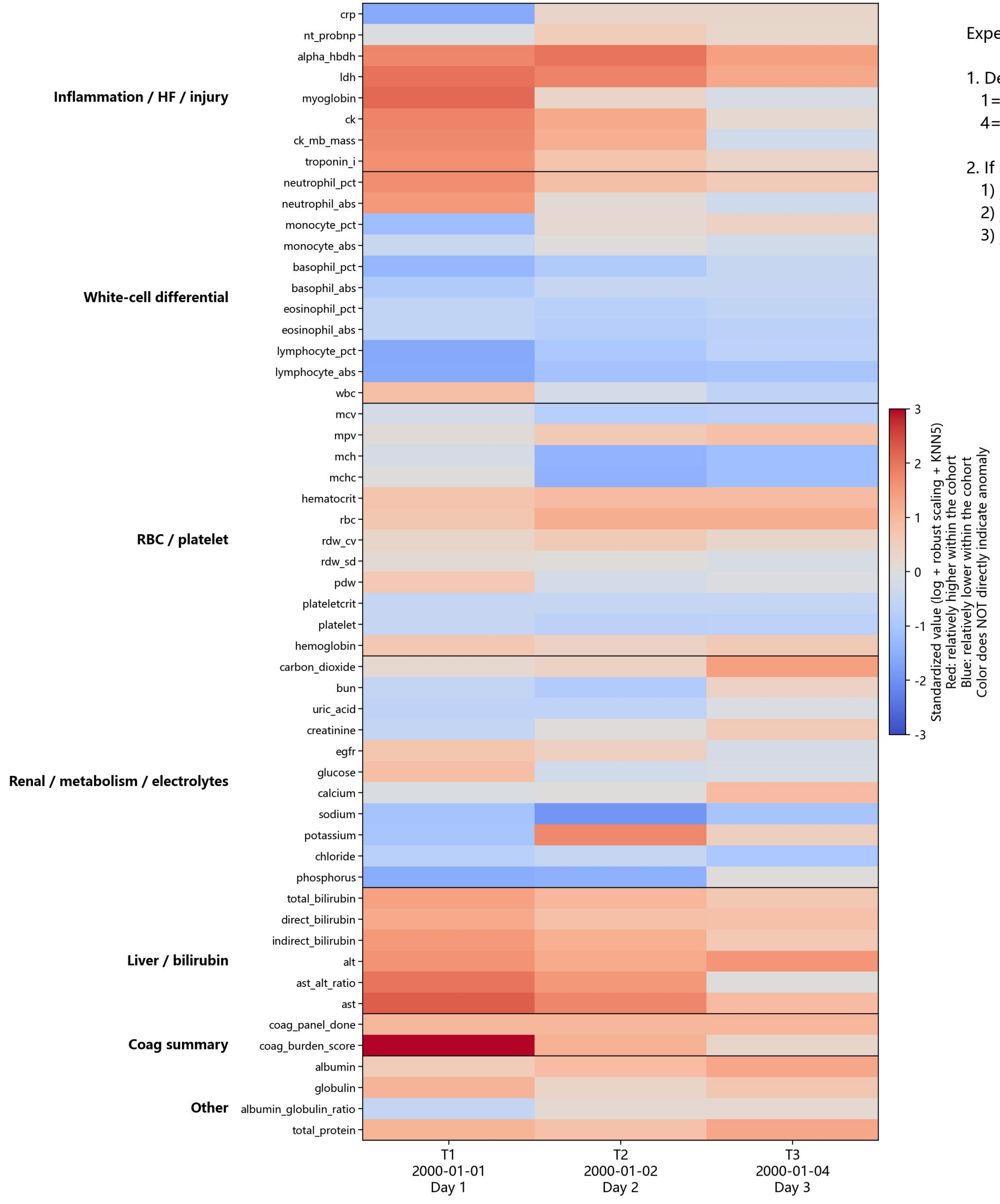

Expert review (blinded; no model score shown)

1. Degree of anomaly for this 3-point window (1-5):
1=very typical; 2=relatively typical; 3=gray zone;
4=relatively abnormal; 5=very abnormal

2. If scored 4-5, list the 3 most abnormal / noteworthy variables:
1) ________________________
2) ________________________
3) ________________________

Patient-window heatmap card for blinded expert review
ID: P080   Window: W01

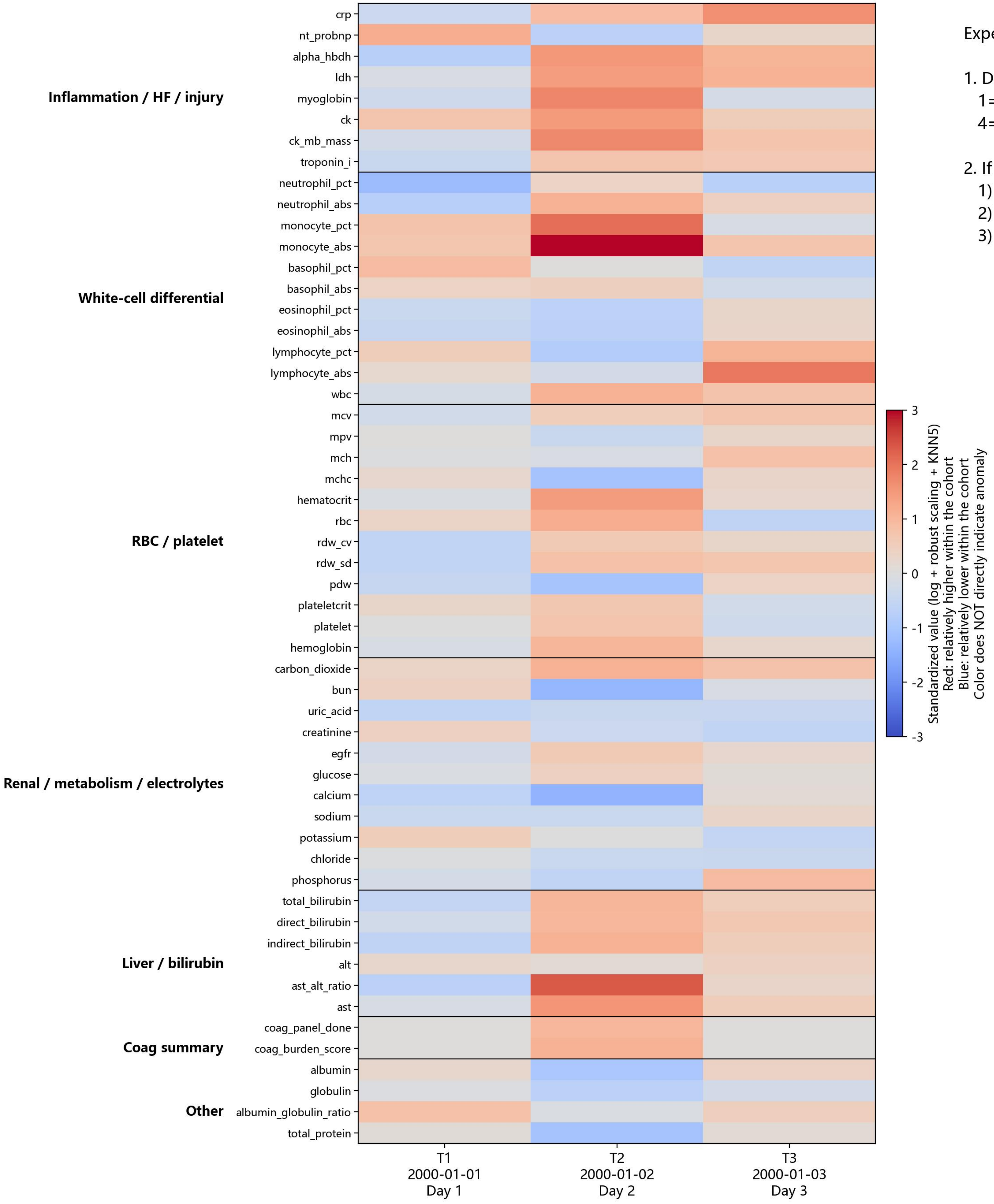

Expert review (blinded; no model score shown)

1. Degree of anomaly for this 3-point window (1-5):
   1=very typical; 2=relatively typical; 3=gray zone;
   4=relatively abnormal; 5=very abnormal

2. If scored 4-5, list the 3 most abnormal / noteworthy variables:
   1) ________________________
   2) ________________________
   3) ________________________

Patient-window heatmap card for blinded expert review
ID: P081   Window: W01

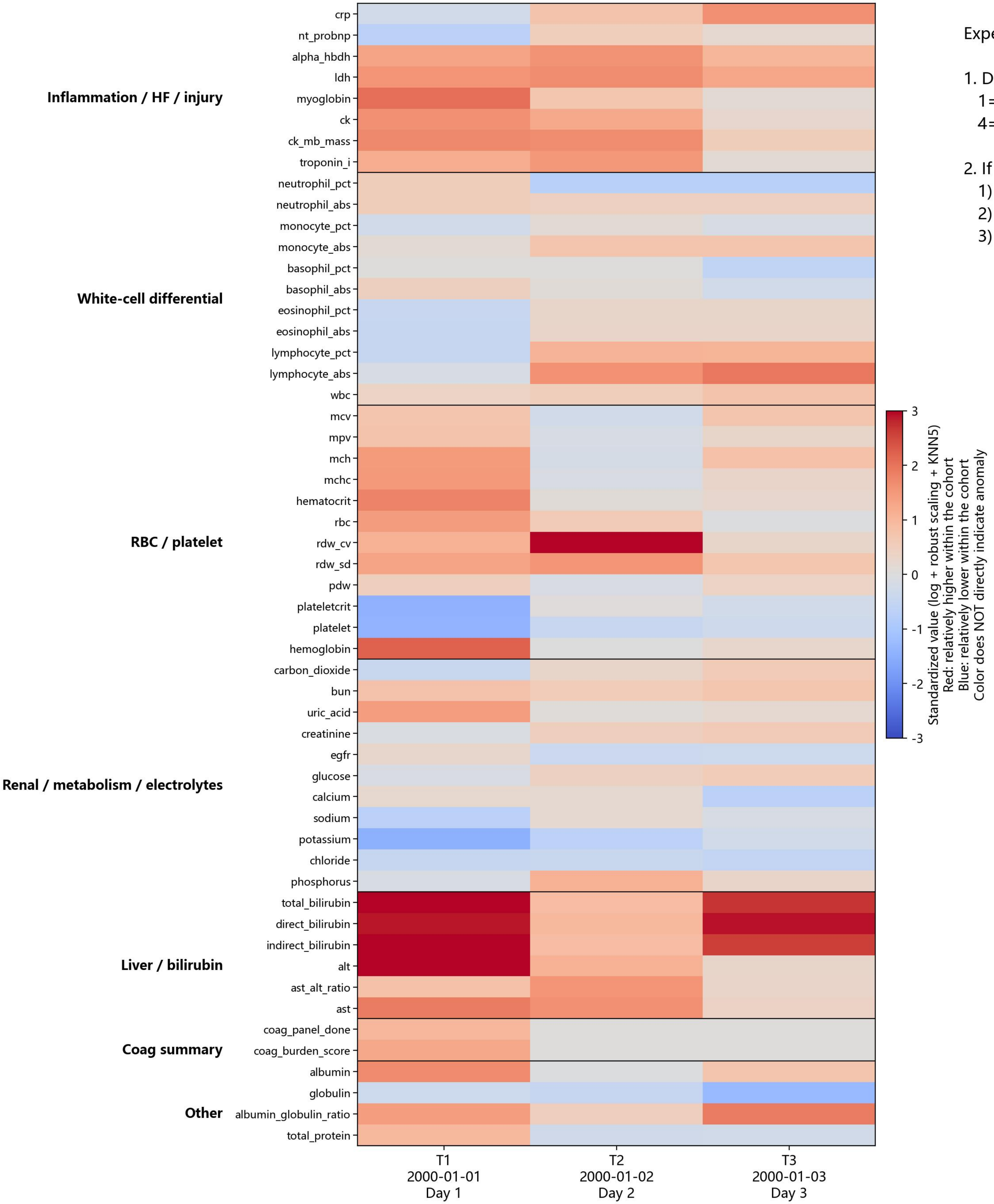

Expert review (blinded; no model score shown)

1. Degree of anomaly for this 3-point window (1-5):
   1=very typical; 2=relatively typical; 3=gray zone;
   4=relatively abnormal; 5=very abnormal

2. If scored 4-5, list the 3 most abnormal / noteworthy variables:
   1) ________________________
   2) ________________________
   3) ________________________

Patient-window heatmap card for blinded expert review
ID: P082   Window: W01

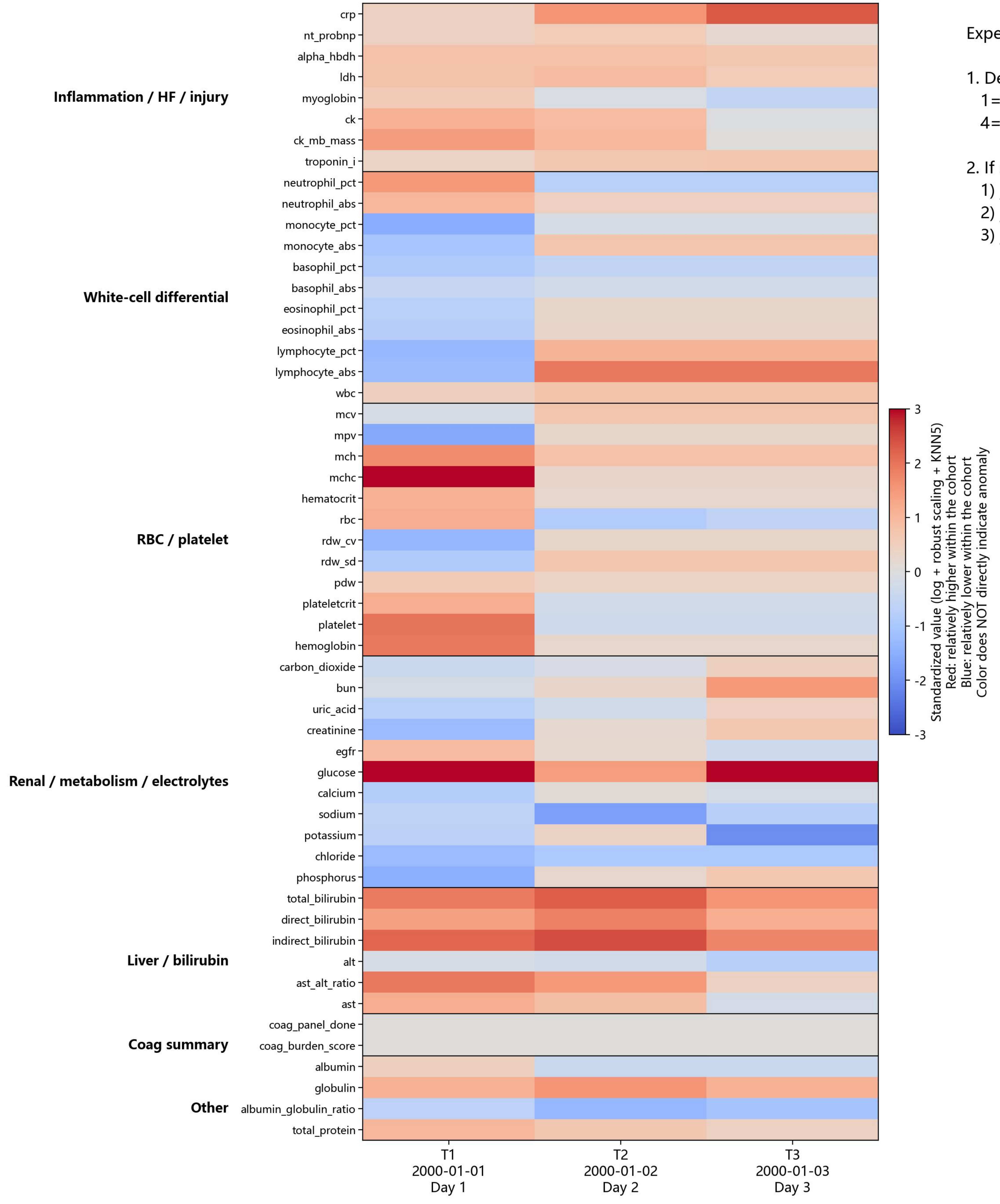

Expert review (blinded; no model score shown)

1. Degree of anomaly for this 3-point window (1-5):
   1=very typical; 2=relatively typical; 3=gray zone;
   4=relatively abnormal; 5=very abnormal

2. If scored 4-5, list the 3 most abnormal / noteworthy variables:
   1) ____________________________
   2) ____________________________
   3) ____________________________

Patient-window heatmap card for blinded expert review
ID: P083 Window: W01

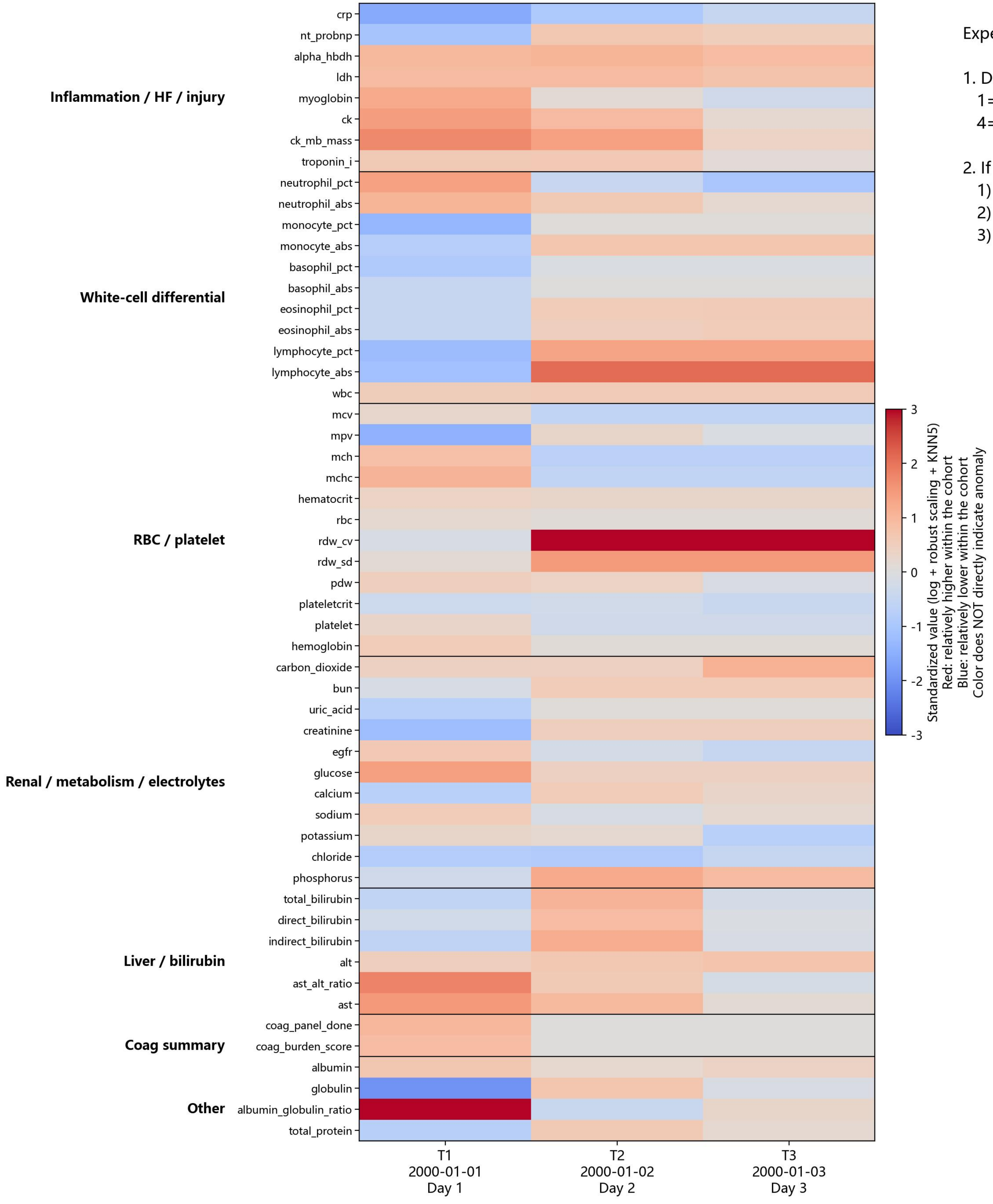

Expert review (blinded; no model score shown)

1. Degree of anomaly for this 3-point window (1-5):
1=very typical; 2=relatively typical; 3=gray zone;
4=relatively abnormal; 5=very abnormal

2. If scored 4-5, list the 3 most abnormal / noteworthy variables:
1) ____________________
2) ____________________
3) ____________________

Patient-window heatmap card for blinded expert review
ID: P084   Window: W01

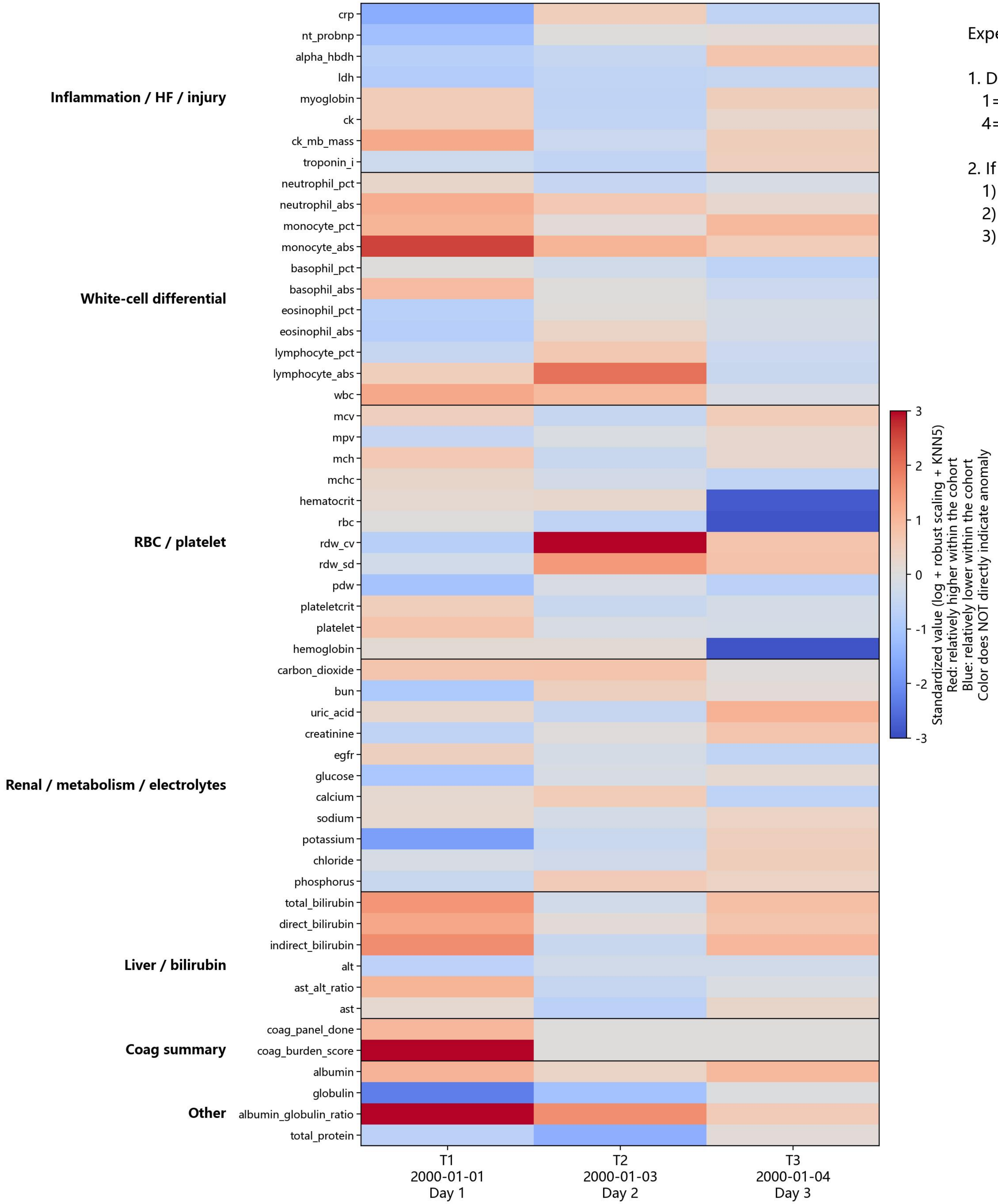

Expert review (blinded; no model score shown)

1. Degree of anomaly for this 3-point window (1-5):
1=very typical; 2=relatively typical; 3=gray zone;
4=relatively abnormal; 5=very abnormal

2. If scored 4-5, list the 3 most abnormal / noteworthy variables:
1) ________________________
2) ________________________
3) ________________________

Patient-window heatmap card for blinded expert review
ID: P085   Window: W01

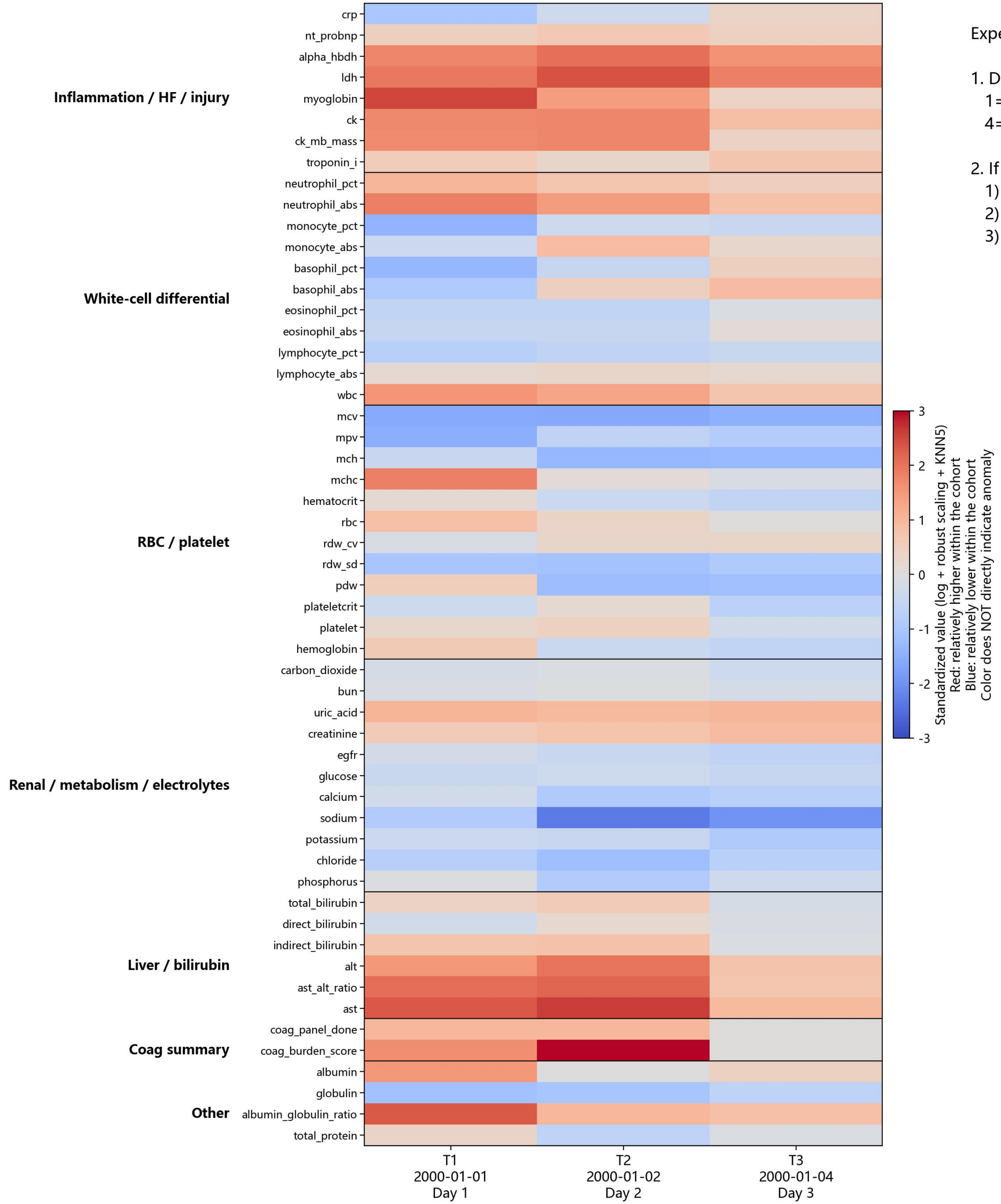

Expert review (blinded; no model score shown)

1. Degree of anomaly for this 3-point window (1-5):
   1=very typical; 2=relatively typical; 3=gray zone;
   4=relatively abnormal; 5=very abnormal

2. If scored 4-5, list the 3 most abnormal / noteworthy variables:
   1) ______________________
   2) ______________________
   3) ______________________

Patient-window heatmap card for blinded expert review
ID: P086   Window: W01

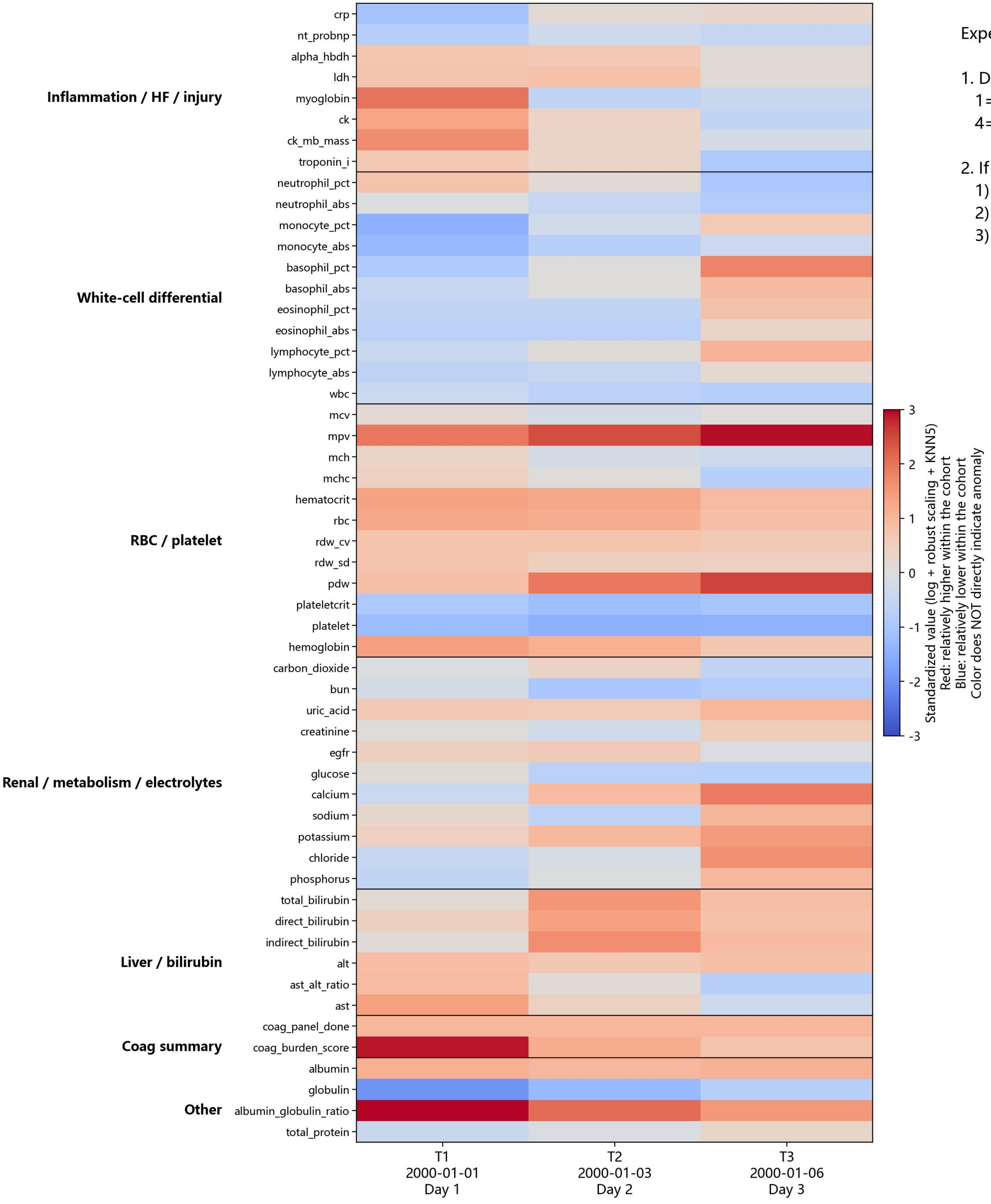

Expert review (blinded; no model score shown)

1. Degree of anomaly for this 3-point window (1-5):
   1=very typical; 2=relatively typical; 3=gray zone;
   4=relatively abnormal; 5=very abnormal

2. If scored 4-5, list the 3 most abnormal / noteworthy variables:
   1) ________________________
   2) ________________________
   3) ________________________

Patient-window heatmap card for blinded expert review
ID: P087   Window: W01

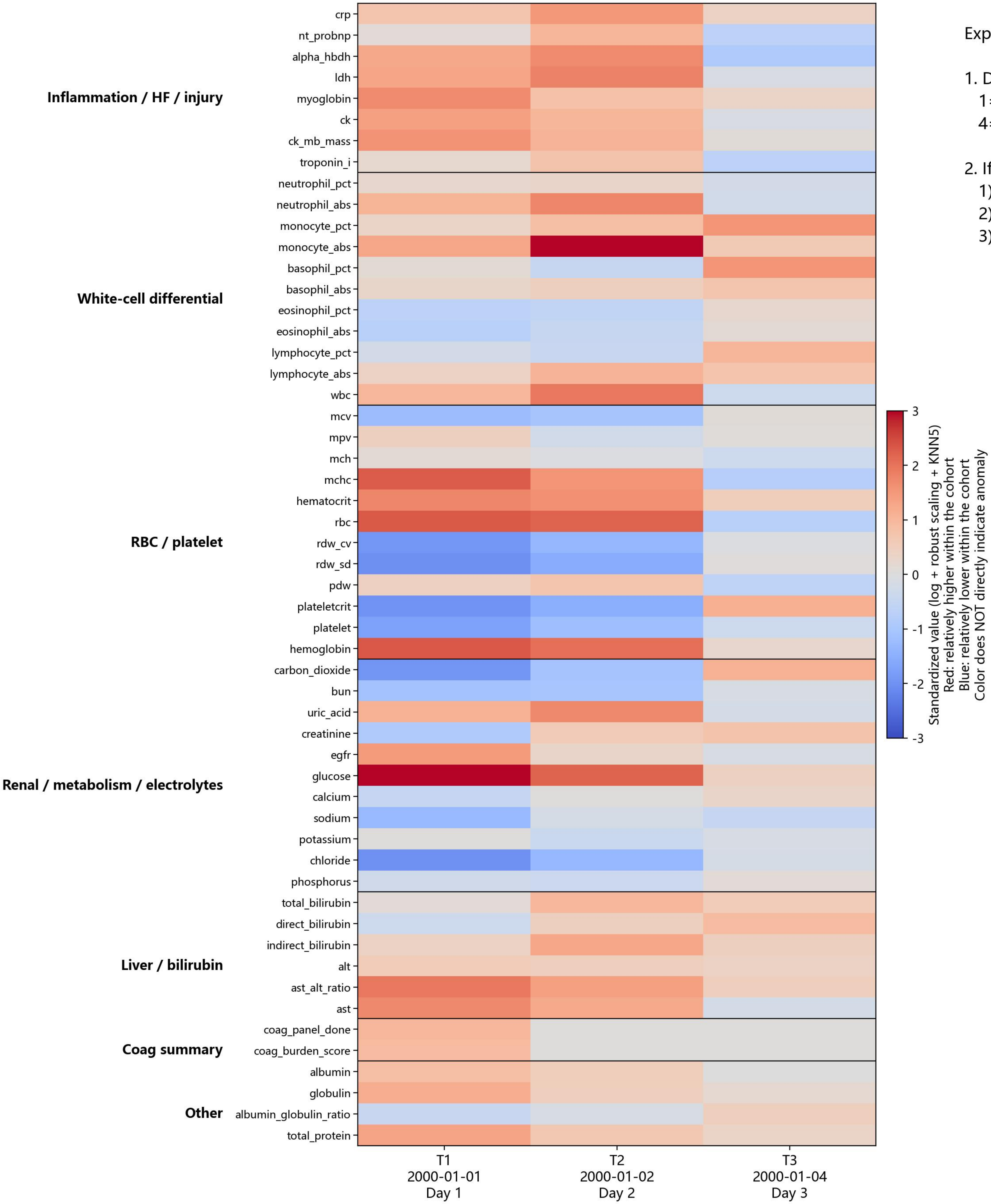

Expert review (blinded; no model score shown)

1. Degree of anomaly for this 3-point window (1-5):
   1=very typical; 2=relatively typical; 3=gray zone;
   4=relatively abnormal; 5=very abnormal

2. If scored 4-5, list the 3 most abnormal / noteworthy variables:
   1) ___________________________
   2) ___________________________
   3) ___________________________

Patient-window heatmap card for blinded expert review
ID: P088   Window: W01

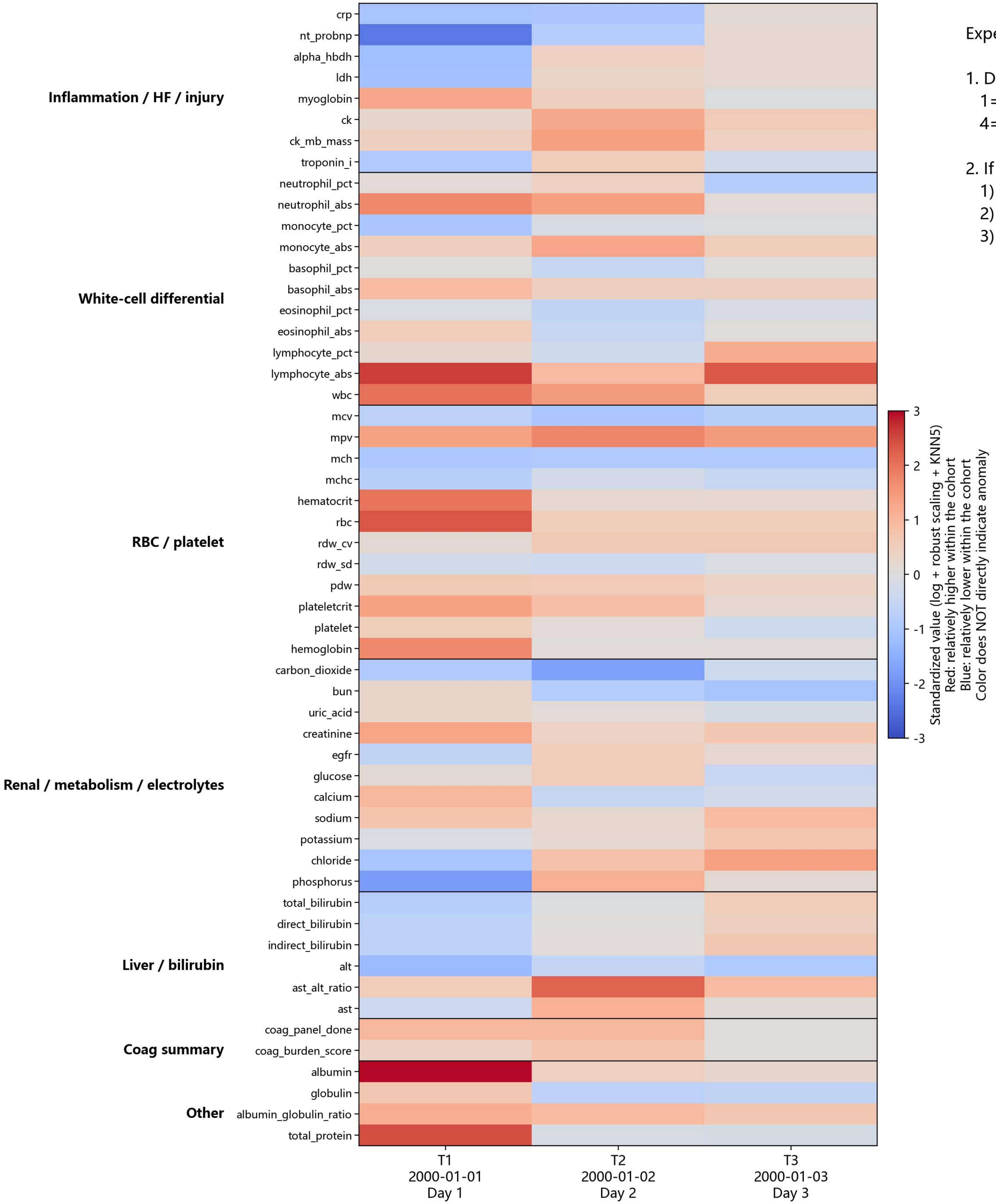

Expert review (blinded; no model score shown)

1. Degree of anomaly for this 3-point window (1-5):
   1=very typical; 2=relatively typical; 3=gray zone;
   4=relatively abnormal; 5=very abnormal

2. If scored 4-5, list the 3 most abnormal / noteworthy variables:
   1) ________________________
   2) ________________________
   3) ________________________

Patient-window heatmap card for blinded expert review
ID: P089   Window: W01

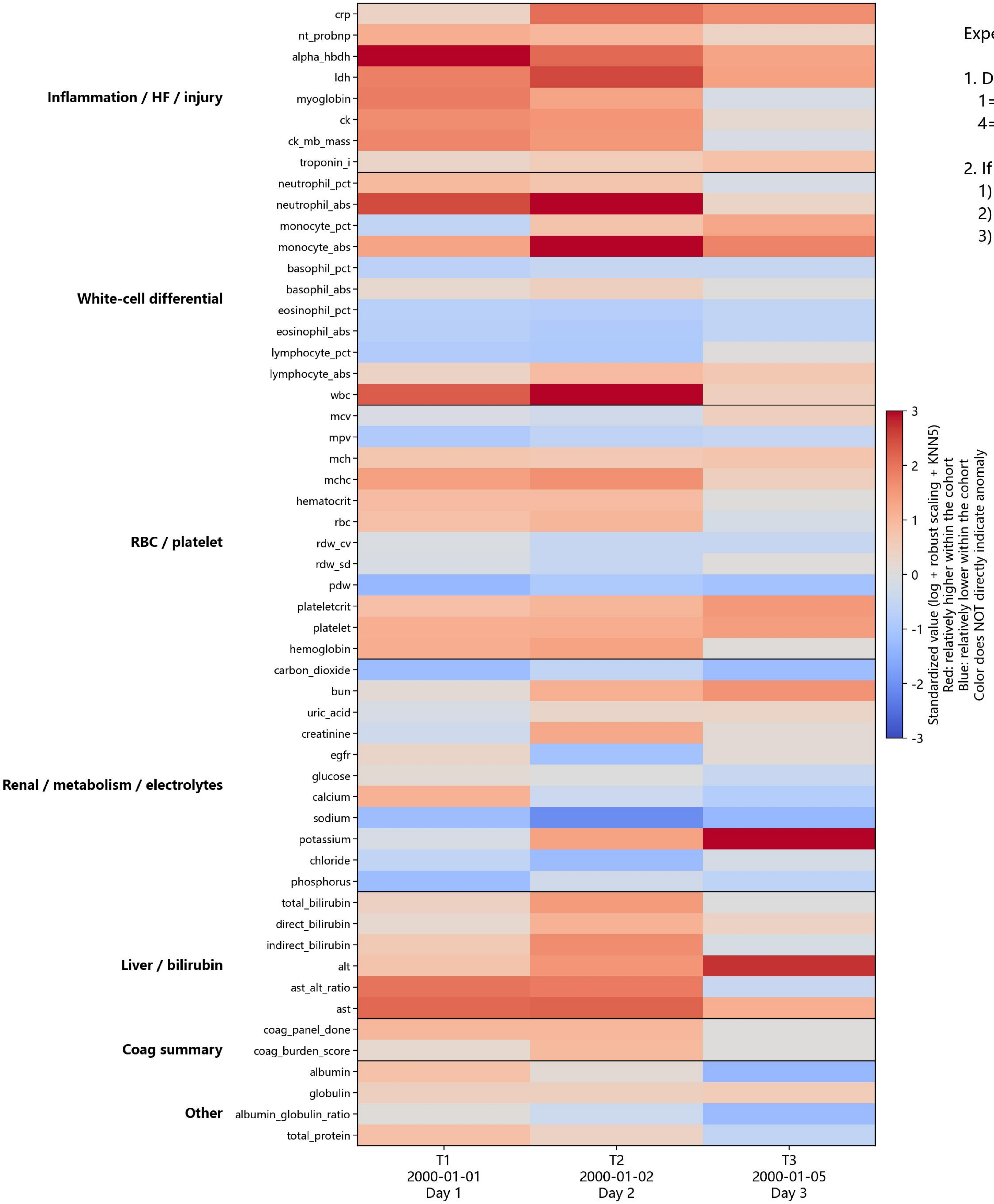

Expert review (blinded; no model score shown)

1. Degree of anomaly for this 3-point window (1-5):
   1=very typical; 2=relatively typical; 3=gray zone;
   4=relatively abnormal; 5=very abnormal

2. If scored 4-5, list the 3 most abnormal / noteworthy variables:
   1) ________________________
   2) ________________________
   3) ________________________

Patient-window heatmap card for blinded expert review
ID: P090   Window: W01

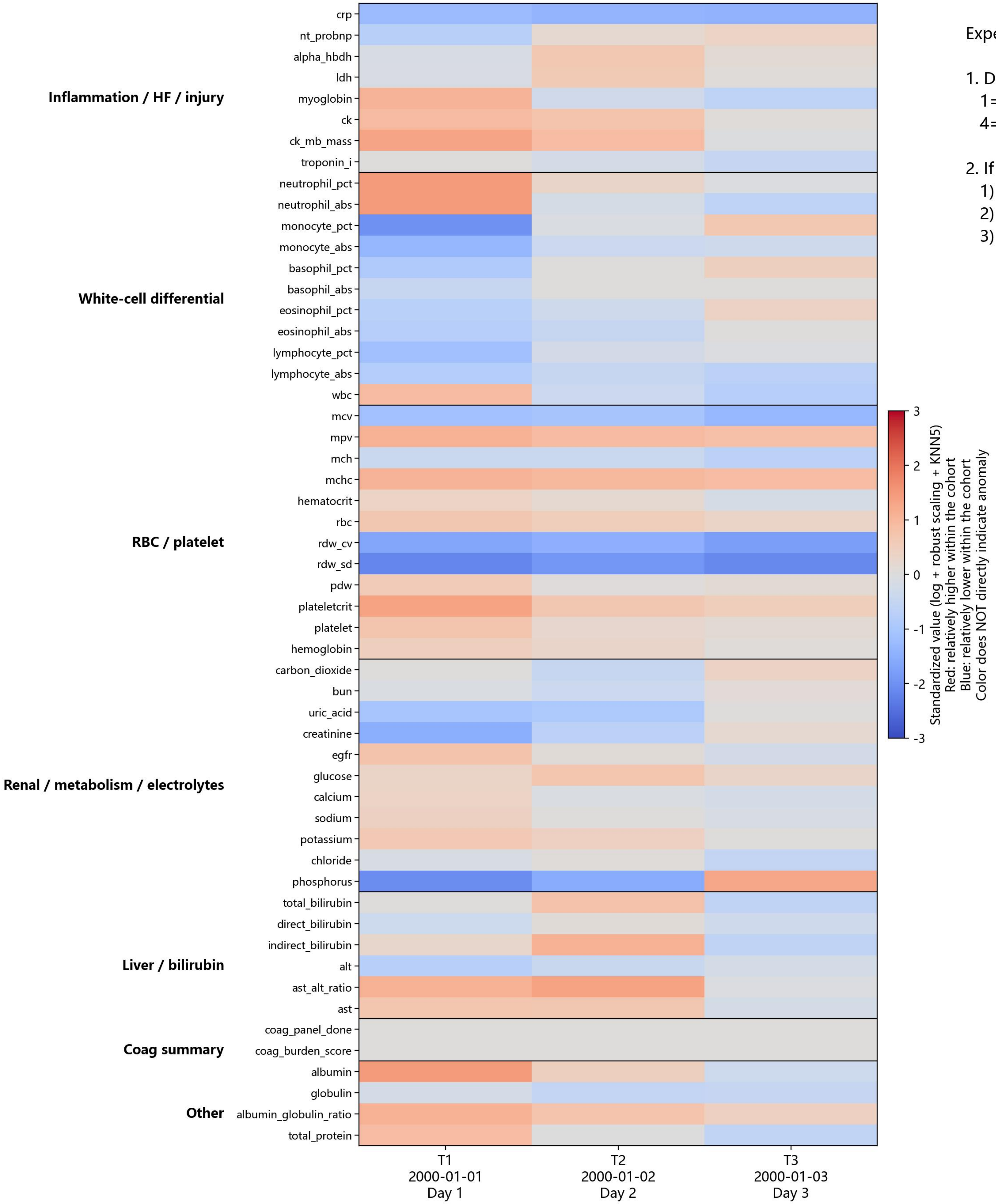

Expert review (blinded; no model score shown)

1. Degree of anomaly for this 3-point window (1-5):
   1=very typical; 2=relatively typical; 3=gray zone;
   4=relatively abnormal; 5=very abnormal

2. If scored 4-5, list the 3 most abnormal / noteworthy variables:
   1) ________________________
   2) ________________________
   3) ________________________

Patient-window heatmap card for blinded expert review
ID: P091 Window: W01

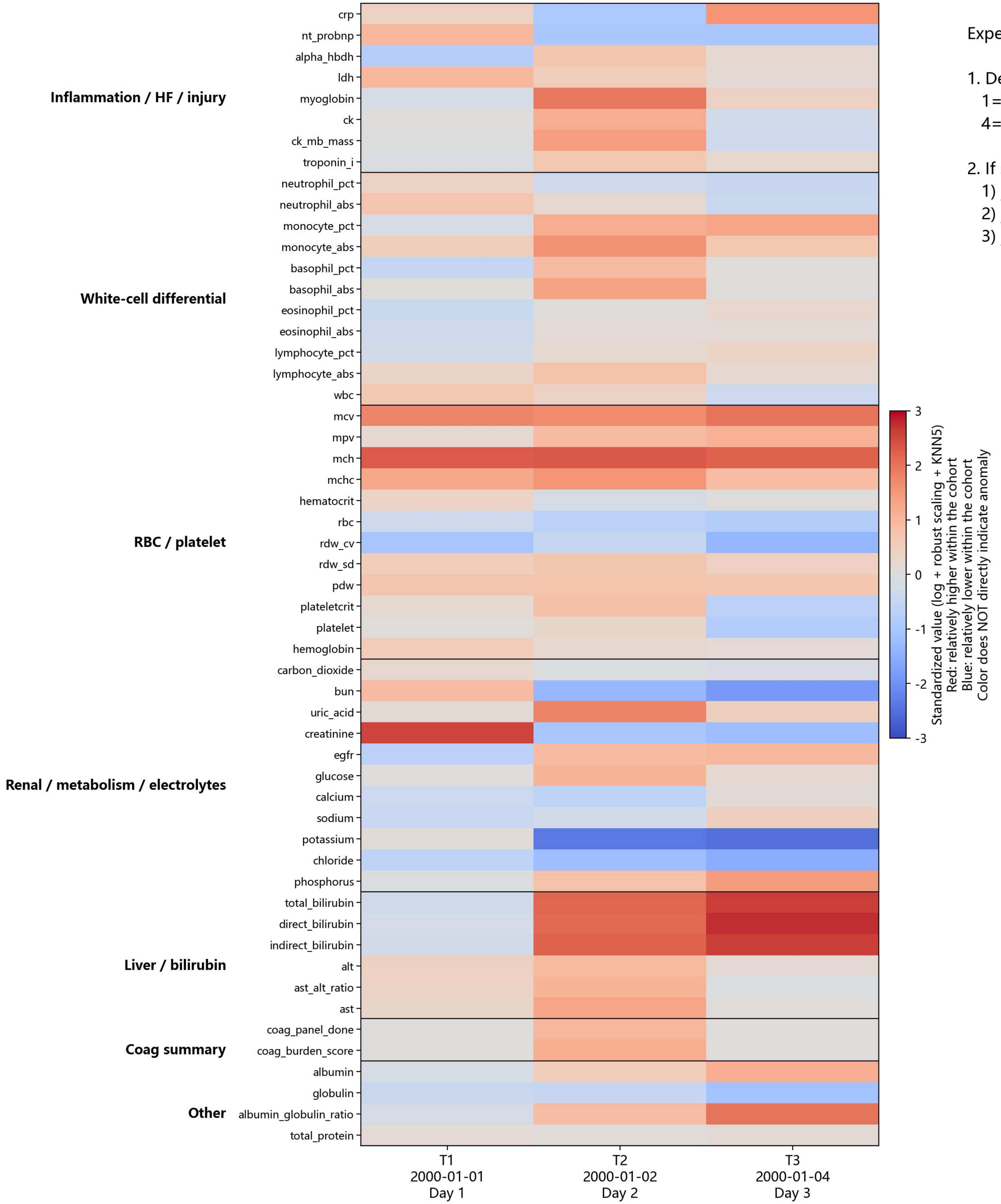

Expert review (blinded; no model score shown)

1. Degree of anomaly for this 3-point window (1-5):
   1=very typical; 2=relatively typical; 3=gray zone;
   4=relatively abnormal; 5=very abnormal

2. If scored 4-5, list the 3 most abnormal / noteworthy variables:
   1) ________________________
   2) ________________________
   3) ________________________

Patient-window heatmap card for blinded expert review
ID: P092   Window: W01

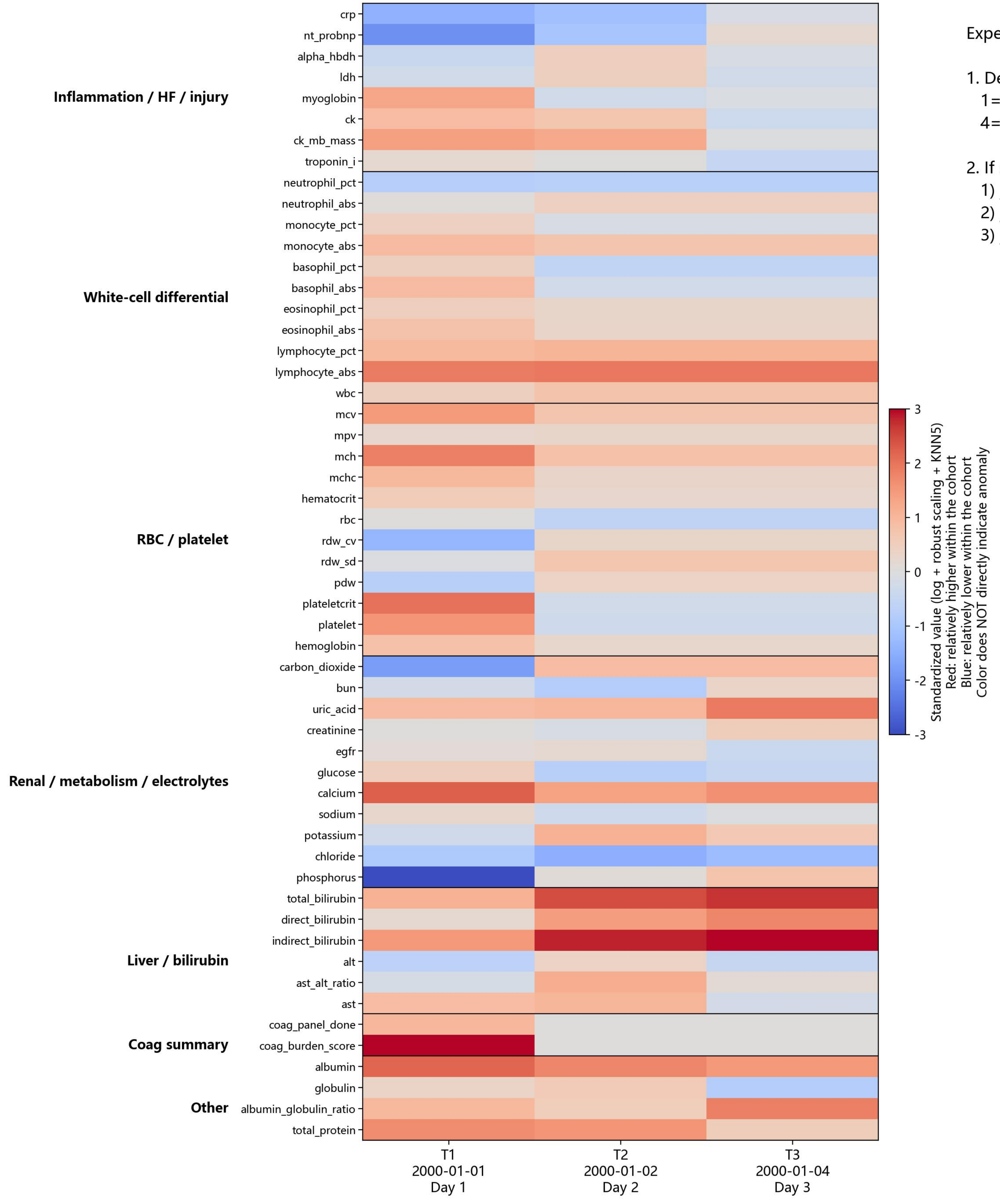

Expert review (blinded; no model score shown)

1. Degree of anomaly for this 3-point window (1-5):
   1=very typical; 2=relatively typical; 3=gray zone;
   4=relatively abnormal; 5=very abnormal

2. If scored 4-5, list the 3 most abnormal / noteworthy variables:
   1) ________________________
   2) ________________________
   3) ________________________

Patient-window heatmap card for blinded expert review
ID: P093   Window: W01

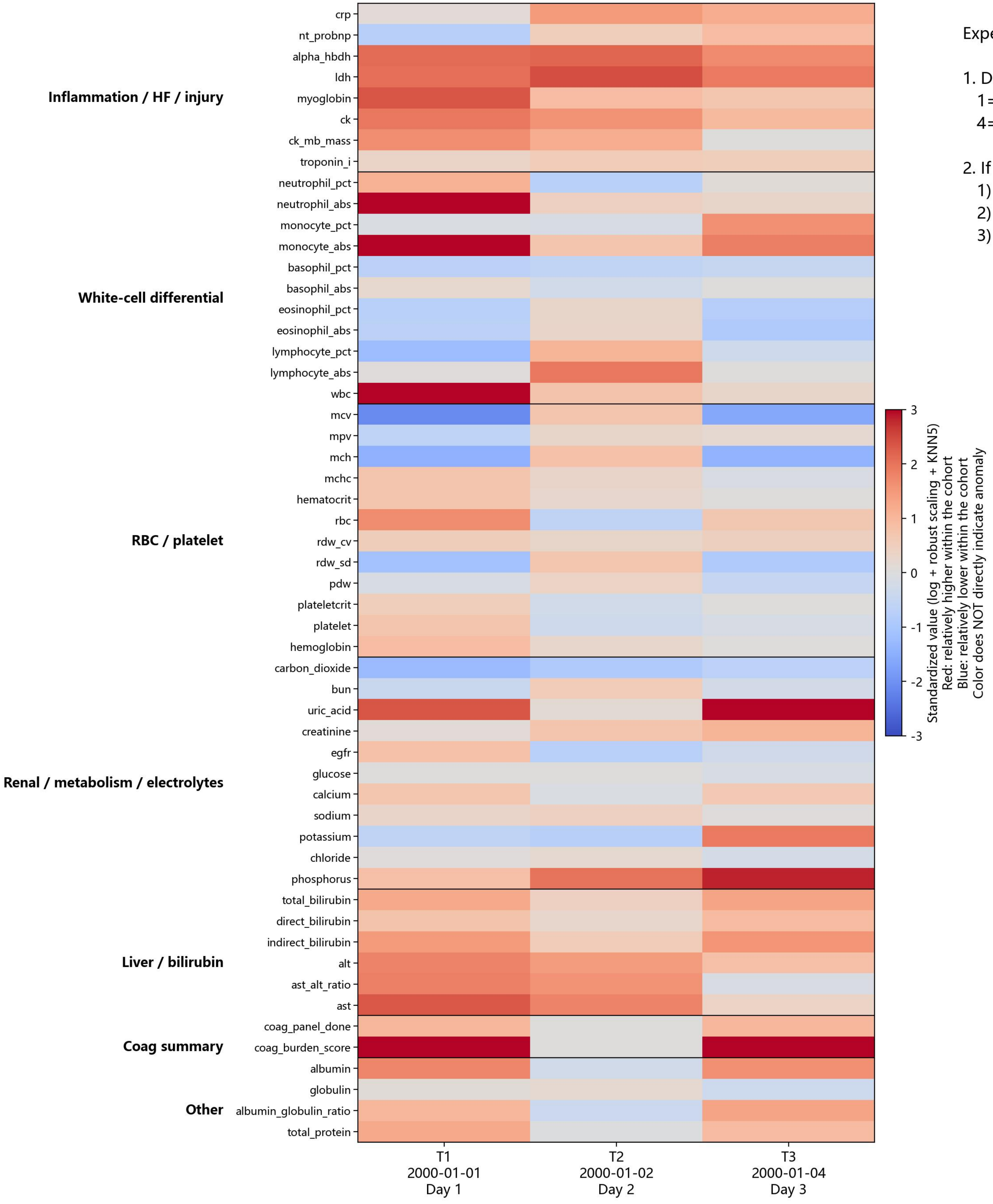

Expert review (blinded; no model score shown)

1. Degree of anomaly for this 3-point window (1-5):
   1=very typical; 2=relatively typical; 3=gray zone;
   4=relatively abnormal; 5=very abnormal

2. If scored 4-5, list the 3 most abnormal / noteworthy variables:
   1) ________________________
   2) ________________________
   3) ________________________

Patient-window heatmap card for blinded expert review
ID: P094   Window: W01

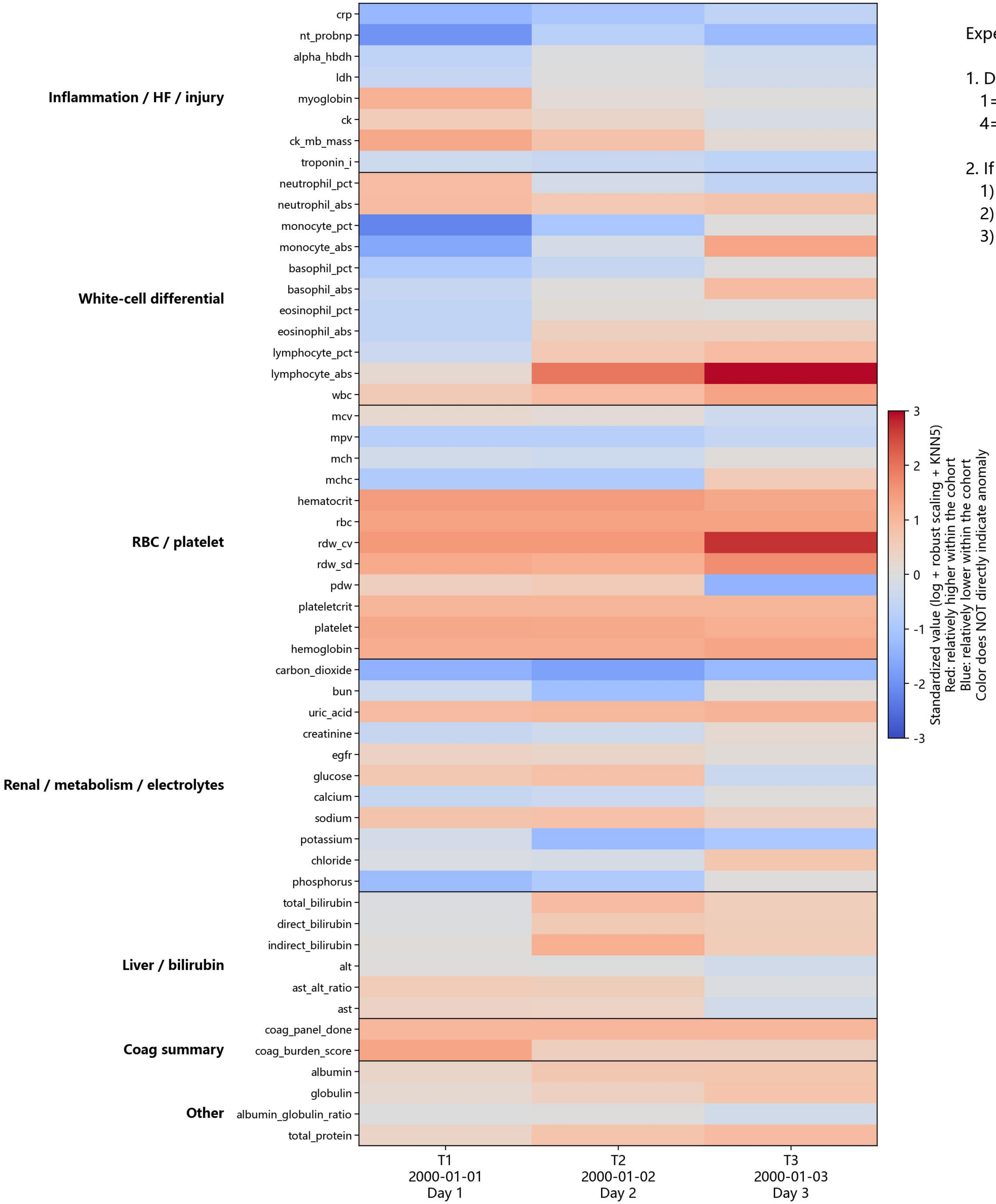

Expert review (blinded; no model score shown)

1. Degree of anomaly for this 3-point window (1-5):
   1=very typical; 2=relatively typical; 3=gray zone;
   4=relatively abnormal; 5=very abnormal

2. If scored 4-5, list the 3 most abnormal / noteworthy variables:
   1) ________________________
   2) ________________________
   3) ________________________

Patient-window heatmap card for blinded expert review
ID: P095   Window: W01

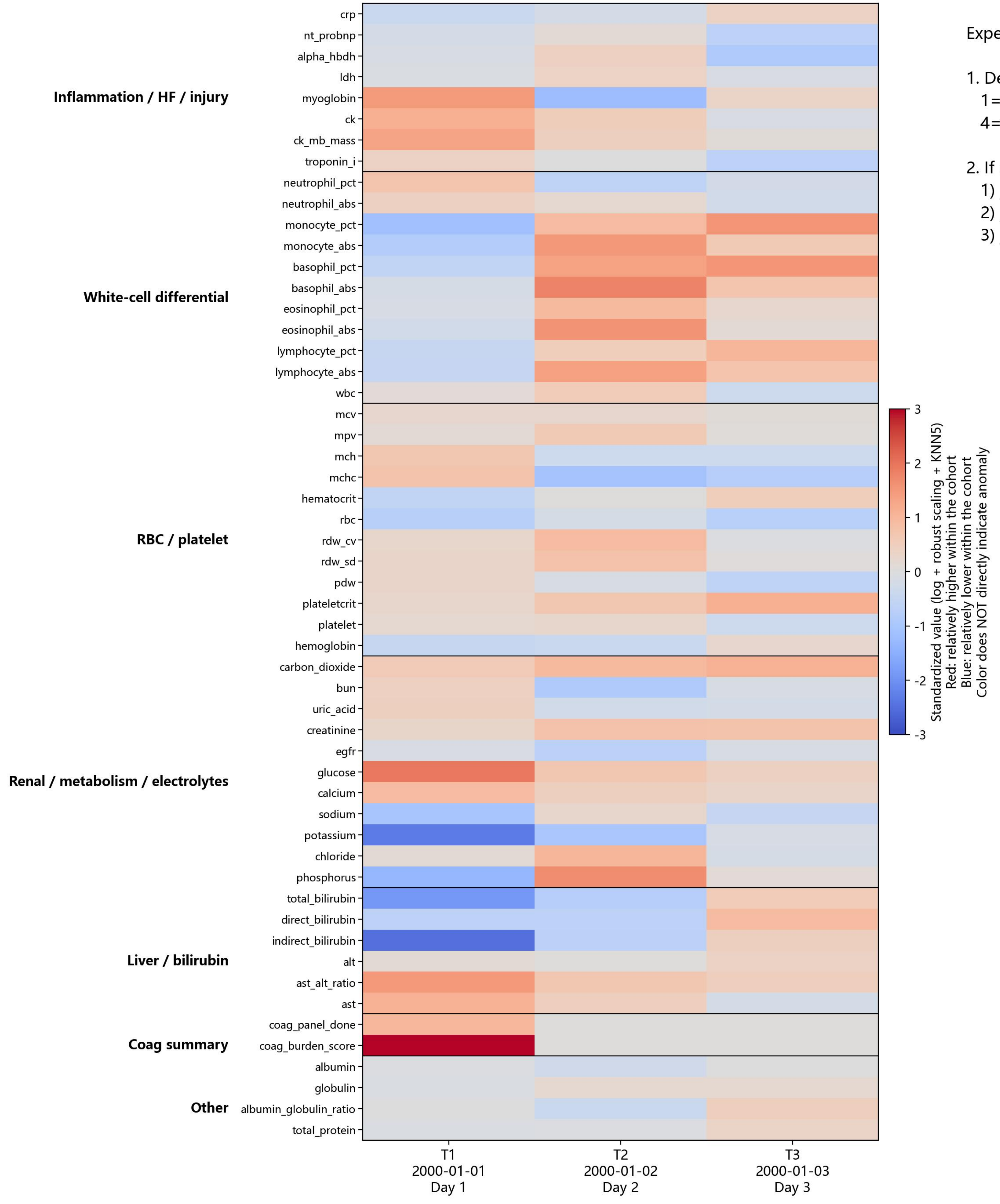

Expert review (blinded; no model score shown)

1. Degree of anomaly for this 3-point window (1-5):
1=very typical; 2=relatively typical; 3=gray zone;
4=relatively abnormal; 5=very abnormal

2. If scored 4-5, list the 3 most abnormal / noteworthy variables:
1) ______________________
2) ______________________
3) ______________________

Patient-window heatmap card for blinded expert review
ID: P096   Window: W01

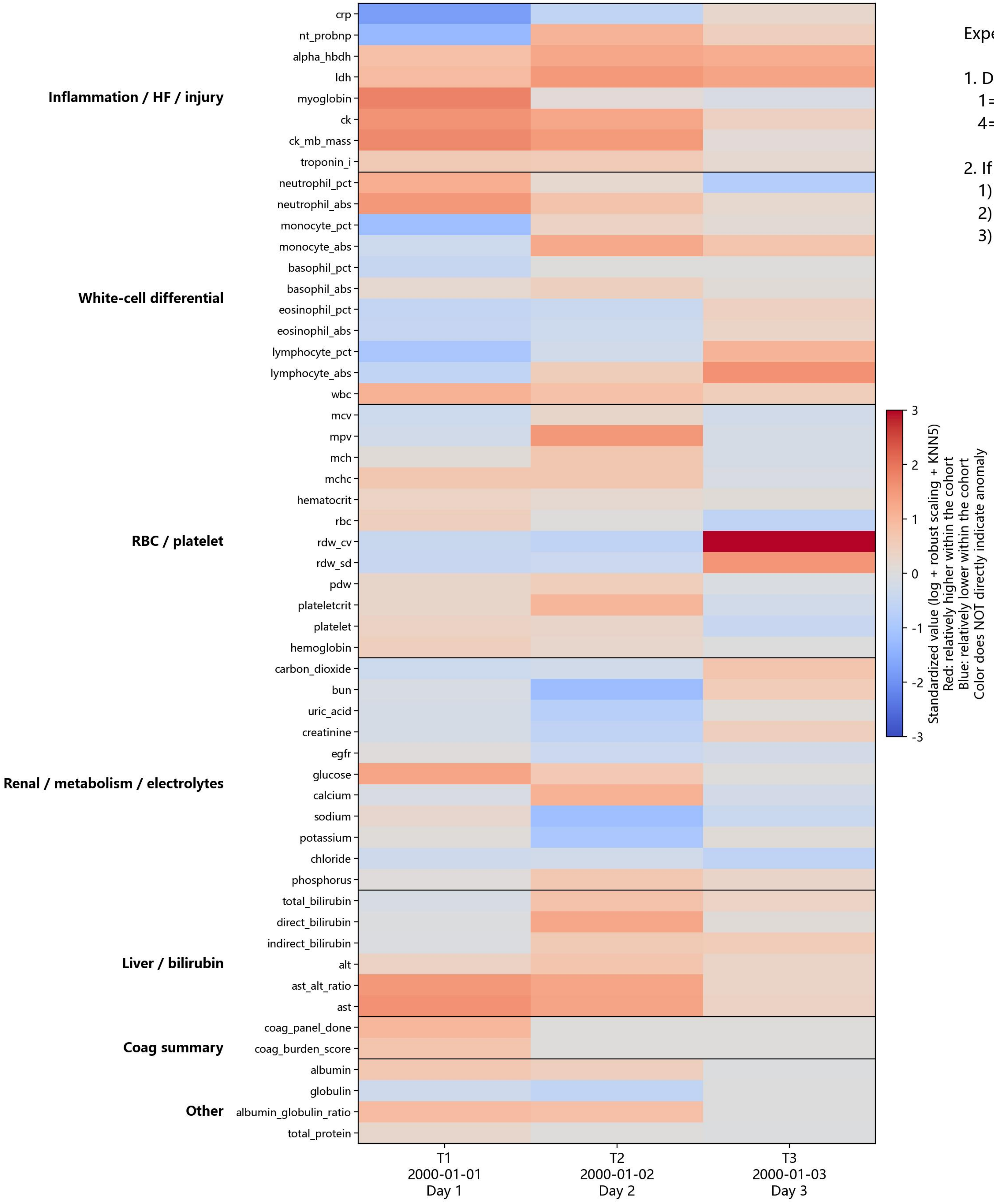

Expert review (blinded; no model score shown)

1. Degree of anomaly for this 3-point window (1-5):
   1=very typical; 2=relatively typical; 3=gray zone;
   4=relatively abnormal; 5=very abnormal

2. If scored 4-5, list the 3 most abnormal / noteworthy variables:
   1) ________________________
   2) ________________________
   3) ________________________

Patient-window heatmap card for blinded expert review
ID: P097   Window: W01

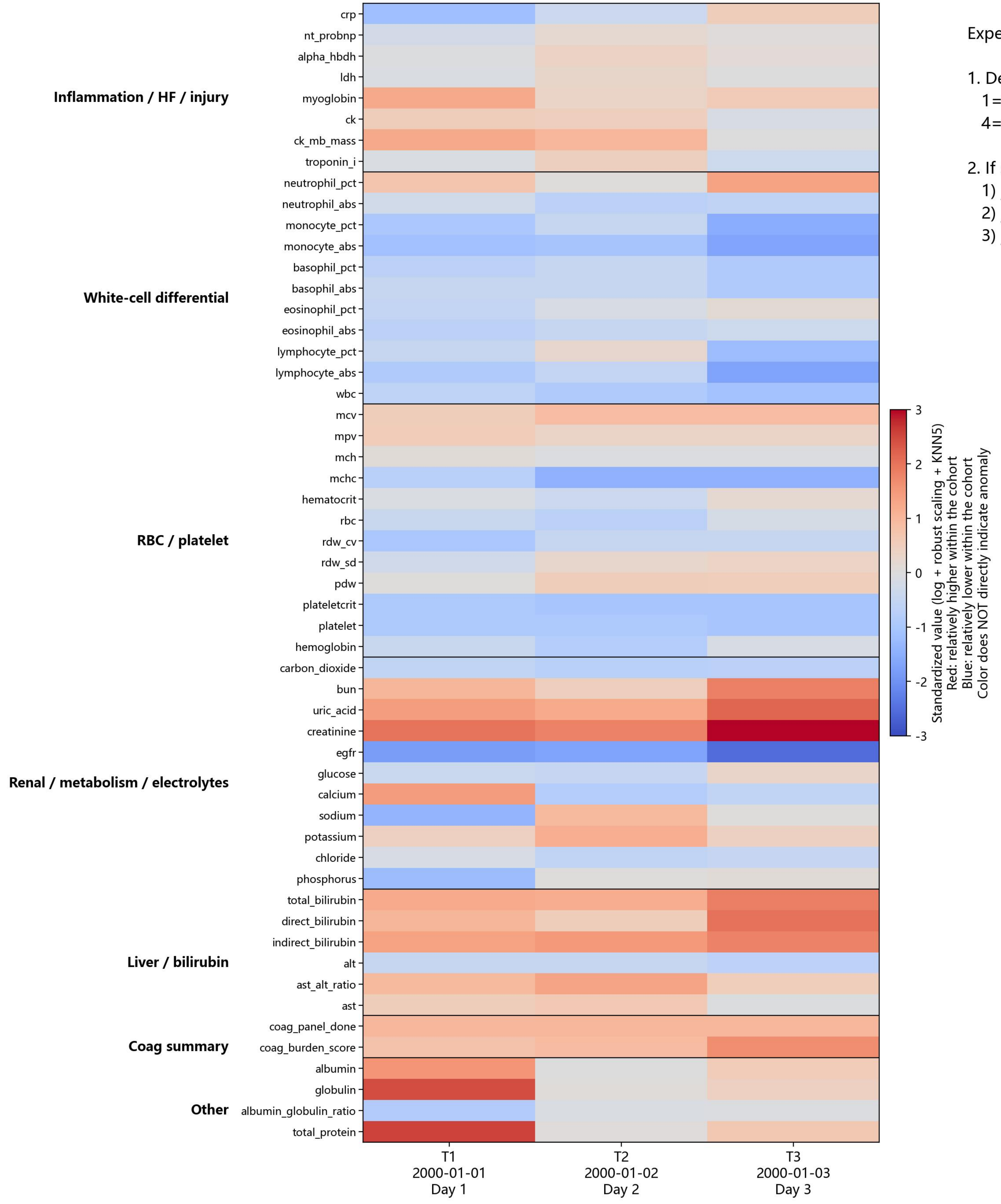

Expert review (blinded; no model score shown)

1. Degree of anomaly for this 3-point window (1-5):
   1=very typical; 2=relatively typical; 3=gray zone;
   4=relatively abnormal; 5=very abnormal

2. If scored 4-5, list the 3 most abnormal / noteworthy variables:
   1) ________________________
   2) ________________________
   3) ________________________

Patient-window heatmap card for blinded expert review
ID: P098   Window: W01

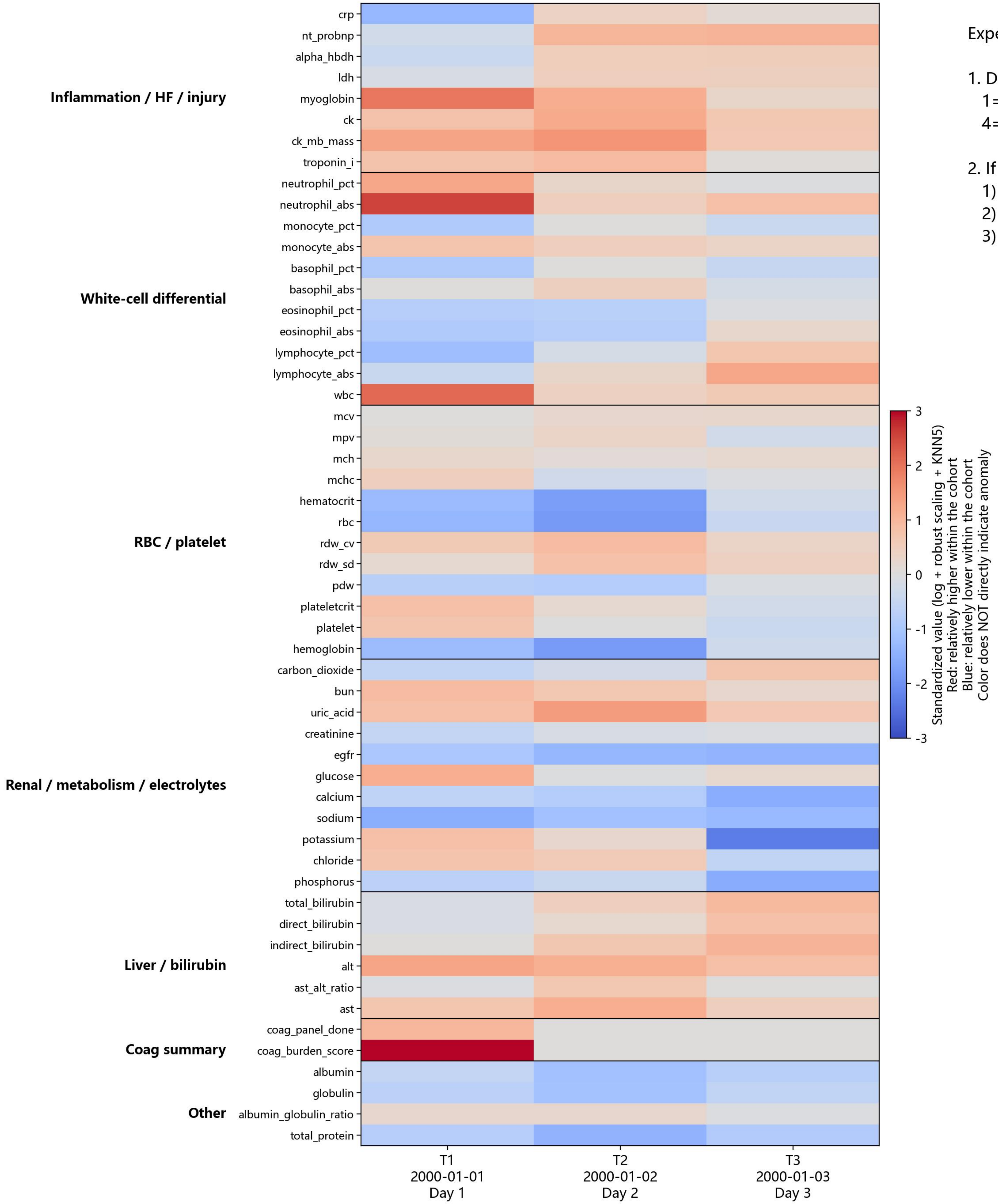

Expert review (blinded; no model score shown)

1. Degree of anomaly for this 3-point window (1-5):
   1=very typical; 2=relatively typical; 3=gray zone;
   4=relatively abnormal; 5=very abnormal

2. If scored 4-5, list the 3 most abnormal / noteworthy variables:
   1) ________________________
   2) ________________________
   3) ________________________

Patient-window heatmap card for blinded expert review
ID: P099   Window: W01

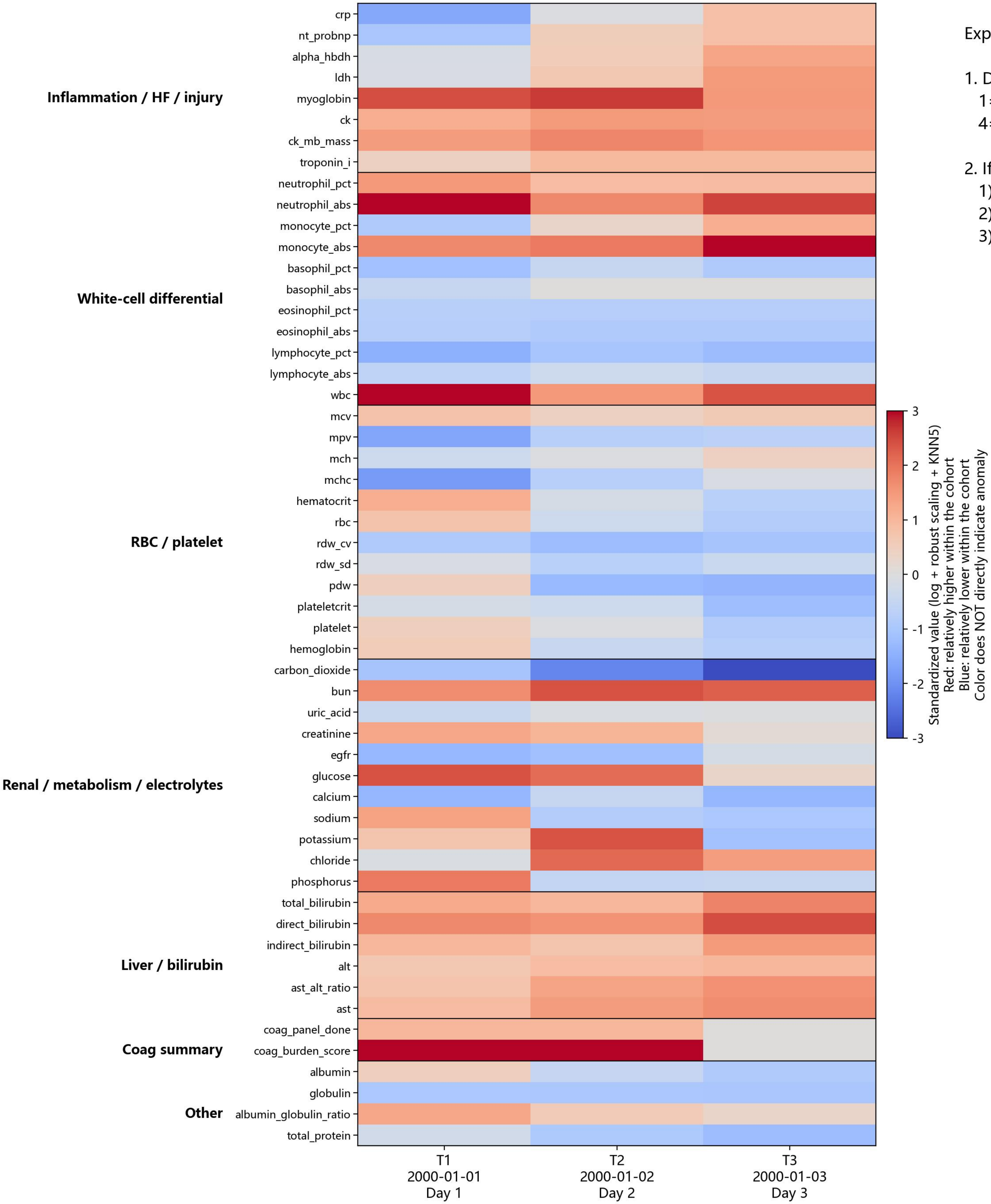

Expert review (blinded; no model score shown)

1. Degree of anomaly for this 3-point window (1-5):
   1=very typical; 2=relatively typical; 3=gray zone;
   4=relatively abnormal; 5=very abnormal

2. If scored 4-5, list the 3 most abnormal / noteworthy variables:
   1) ________________________
   2) ________________________
   3) ________________________

Patient-window heatmap card for blinded expert review
ID: P100 Window: W01

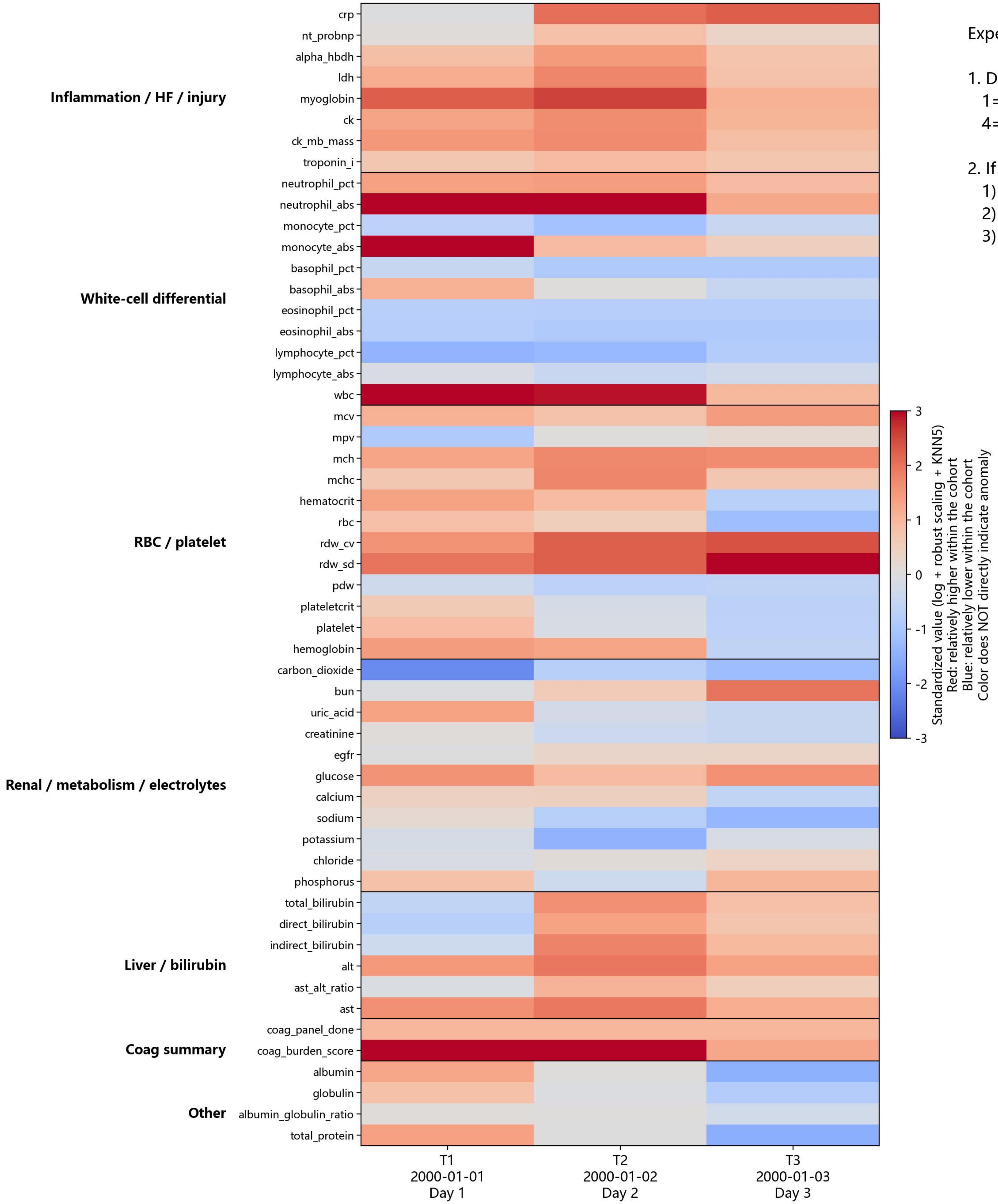

Expert review (blinded; no model score shown)

1. Degree of anomaly for this 3-point window (1-5):
   1=very typical; 2=relatively typical; 3=gray zone;
   4=relatively abnormal; 5=very abnormal

2. If scored 4-5, list the 3 most abnormal / noteworthy variables:
   1) ________________________
   2) ________________________
   3) ________________________

Patient-window heatmap card for blinded expert review
ID: P101 Window: W01

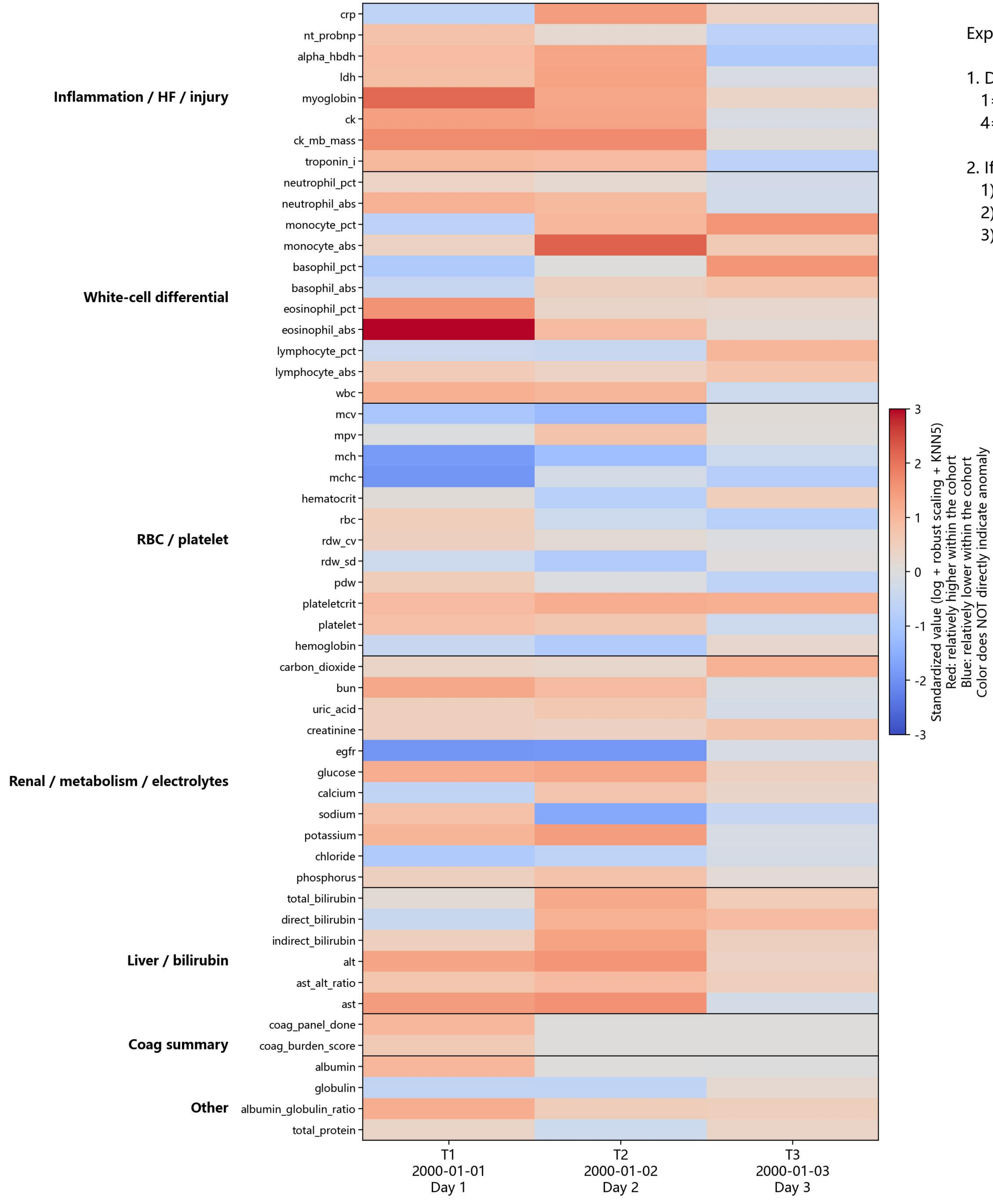

Expert review (blinded; no model score shown)

1. Degree of anomaly for this 3-point window (1-5):
 1=very typical; 2=relatively typical; 3=gray zone;
 4=relatively abnormal; 5=very abnormal

2. If scored 4-5, list the 3 most abnormal / noteworthy variables:
 1) ________________________
 2) ________________________
 3) ________________________

Patient-window heatmap card for blinded expert review
ID: P102 Window: W01

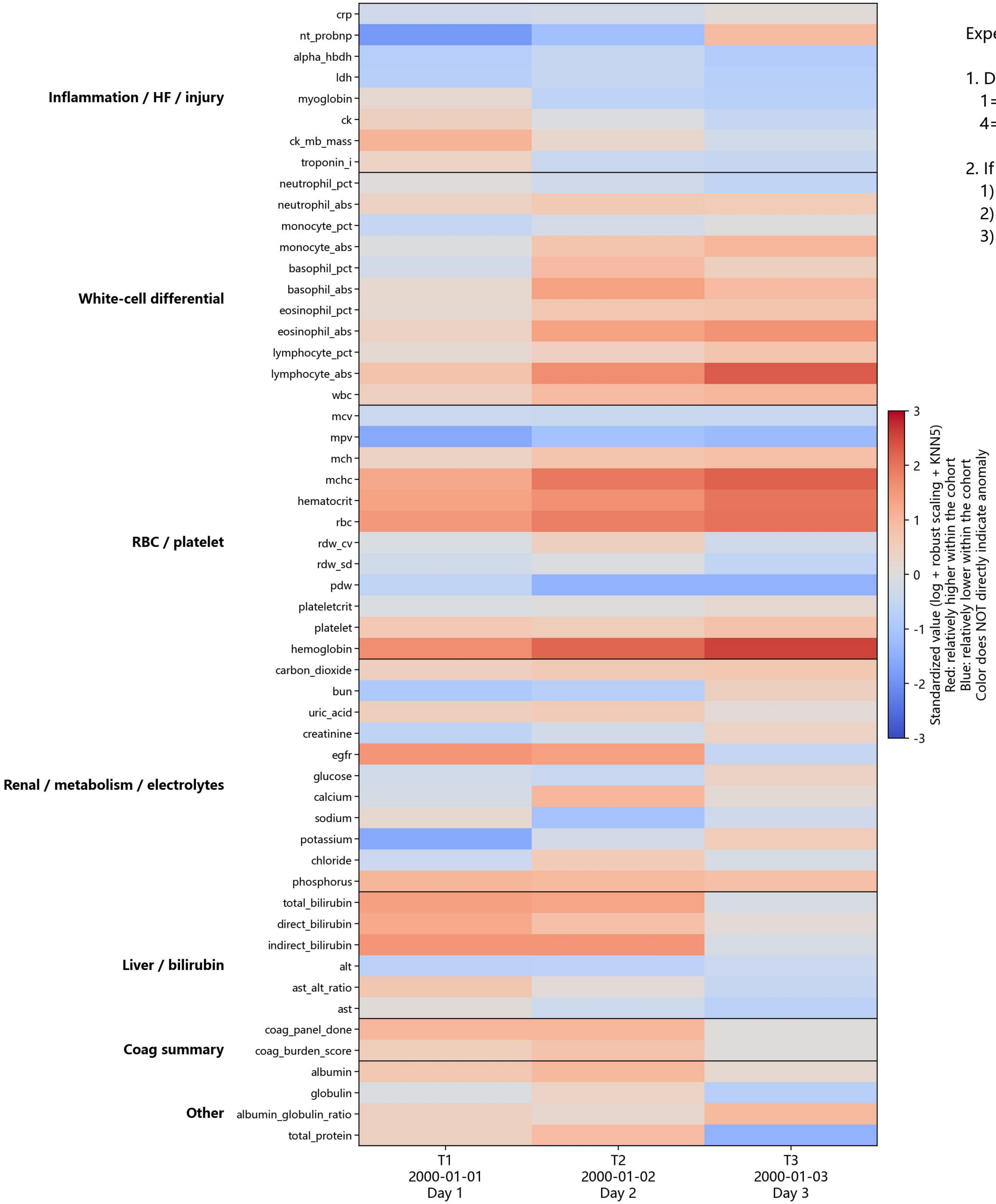

Expert review (blinded; no model score shown)

1. Degree of anomaly for this 3-point window (1-5):
   1=very typical; 2=relatively typical; 3=gray zone;
   4=relatively abnormal; 5=very abnormal

2. If scored 4-5, list the 3 most abnormal / noteworthy variables:
   1) ______________________
   2) ______________________
   3) ______________________

Patient-window heatmap card for blinded expert review
ID: P103   Window: W01

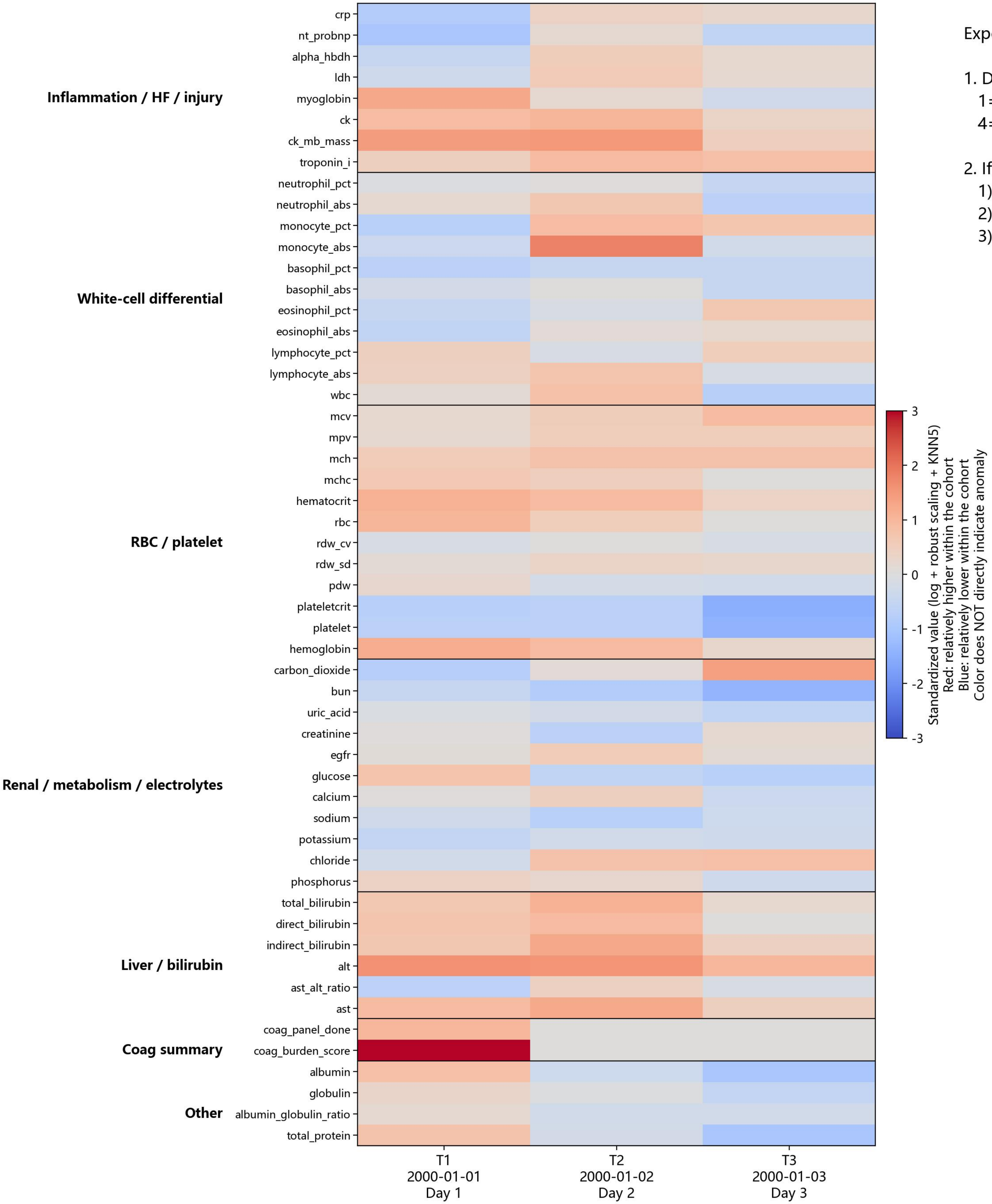

Expert review (blinded; no model score shown)

1. Degree of anomaly for this 3-point window (1-5):
   1=very typical; 2=relatively typical; 3=gray zone;
   4=relatively abnormal; 5=very abnormal

2. If scored 4-5, list the 3 most abnormal / noteworthy variables:
   1) ________________________
   2) ________________________
   3) ________________________

Patient-window heatmap card for blinded expert review
ID: P104   Window: W01

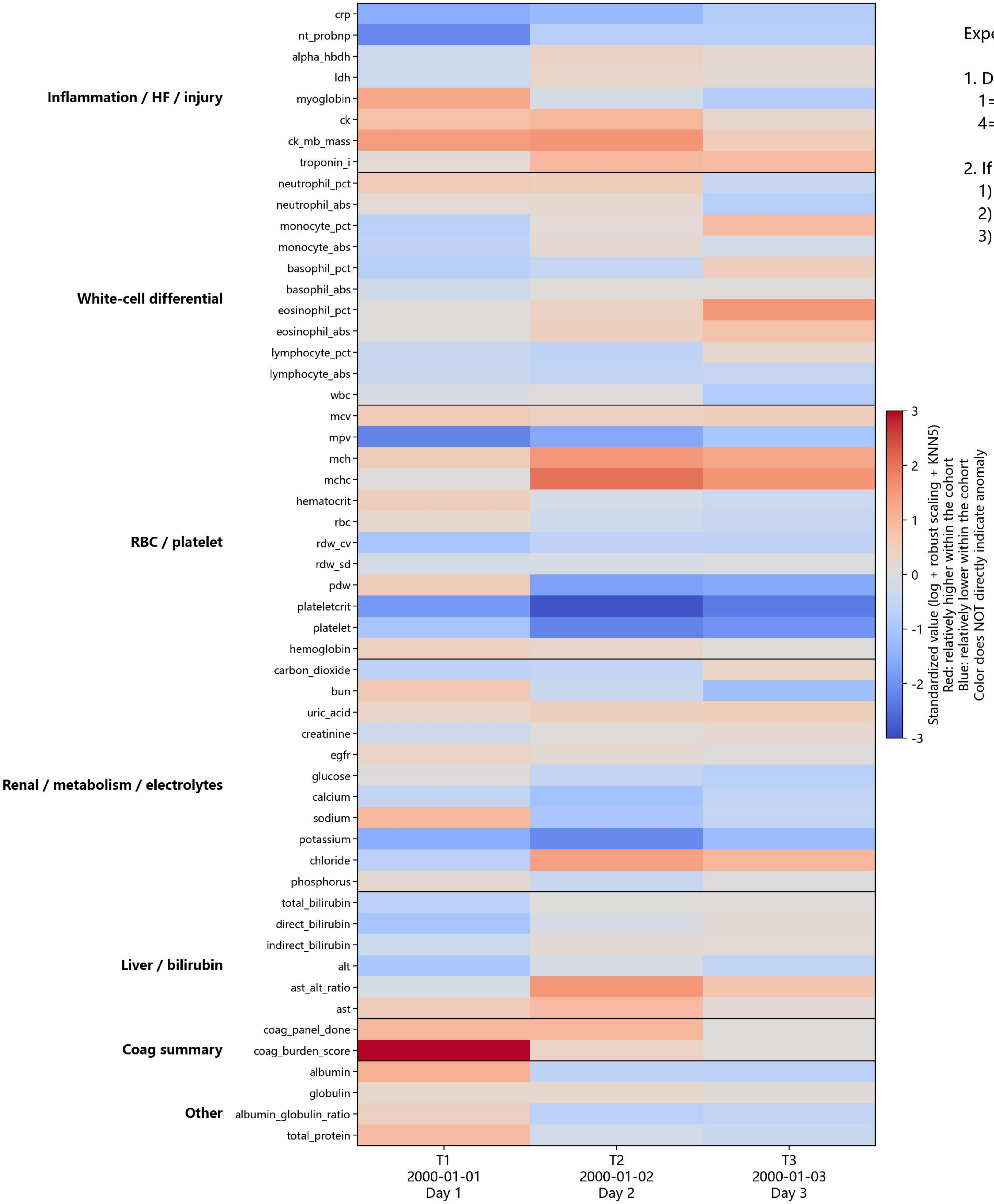

Expert review (blinded; no model score shown)

1. Degree of anomaly for this 3-point window (1-5):
   1=very typical; 2=relatively typical; 3=gray zone;
   4=relatively abnormal; 5=very abnormal

2. If scored 4-5, list the 3 most abnormal / noteworthy variables:
   1) ___________________________
   2) ___________________________
   3) ___________________________

Patient-window heatmap card for blinded expert review
ID: P105   Window: W01

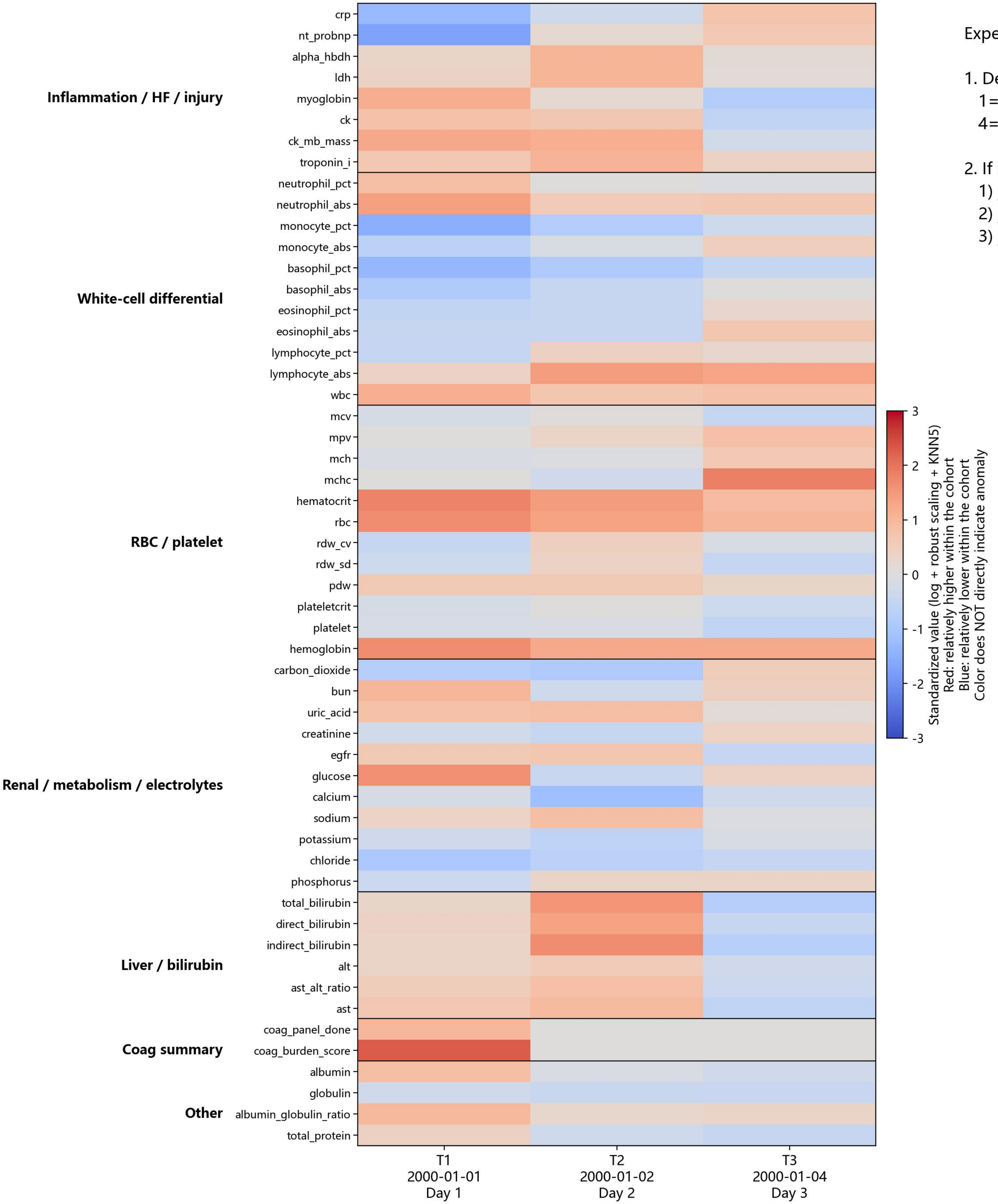

Expert review (blinded; no model score shown)

1. Degree of anomaly for this 3-point window (1-5):
   1=very typical; 2=relatively typical; 3=gray zone;
   4=relatively abnormal; 5=very abnormal

2. If scored 4-5, list the 3 most abnormal / noteworthy variables:
   1) ____________________________
   2) ____________________________
   3) ____________________________

Patient-window heatmap card for blinded expert review
ID: P106   Window: W01

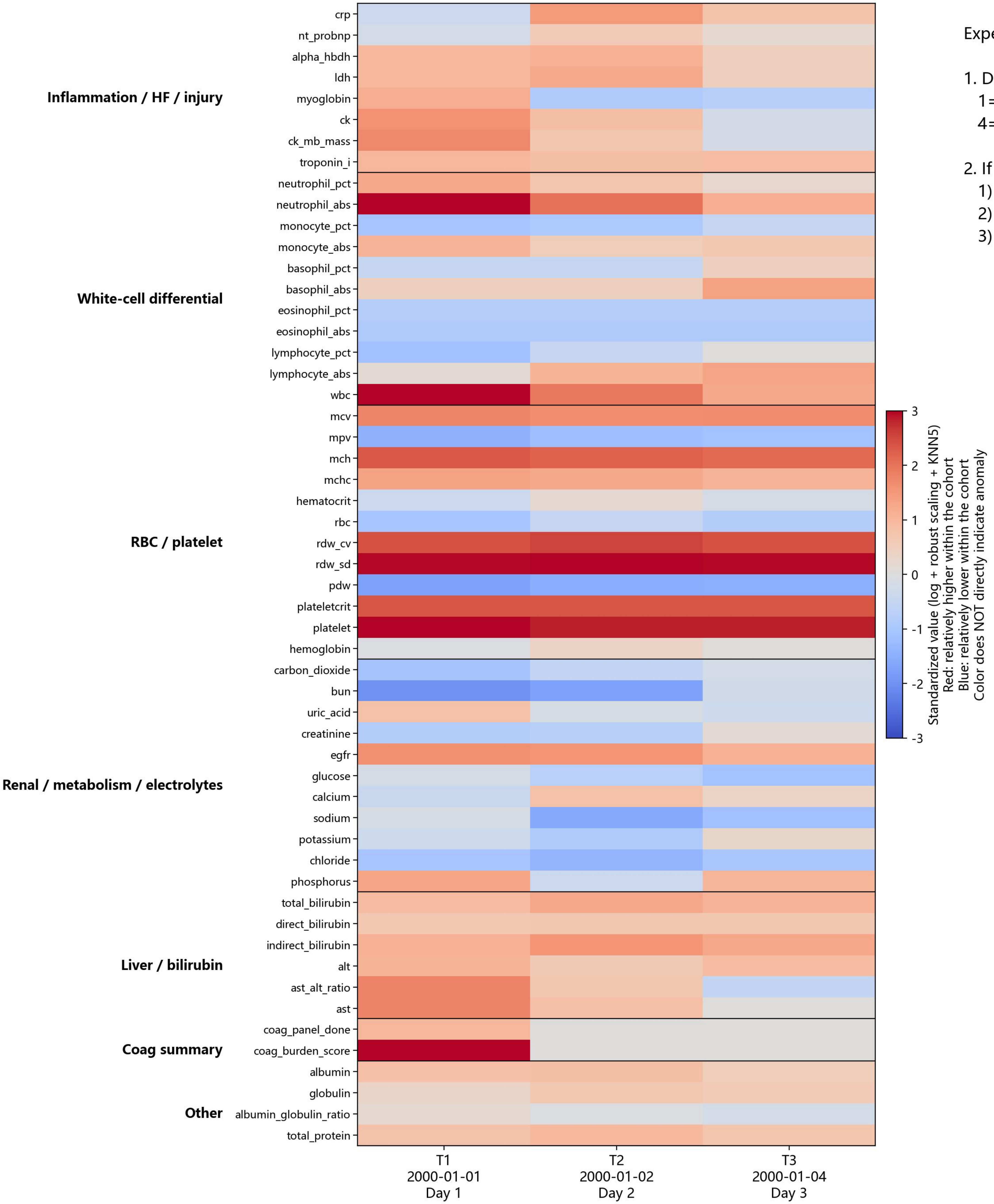

Expert review (blinded; no model score shown)

1. Degree of anomaly for this 3-point window (1-5):
1=very typical; 2=relatively typical; 3=gray zone;
4=relatively abnormal; 5=very abnormal

2. If scored 4-5, list the 3 most abnormal / noteworthy variables:
1) ________________________
2) ________________________
3) ________________________

Patient-window heatmap card for blinded expert review
ID: P107   Window: W01

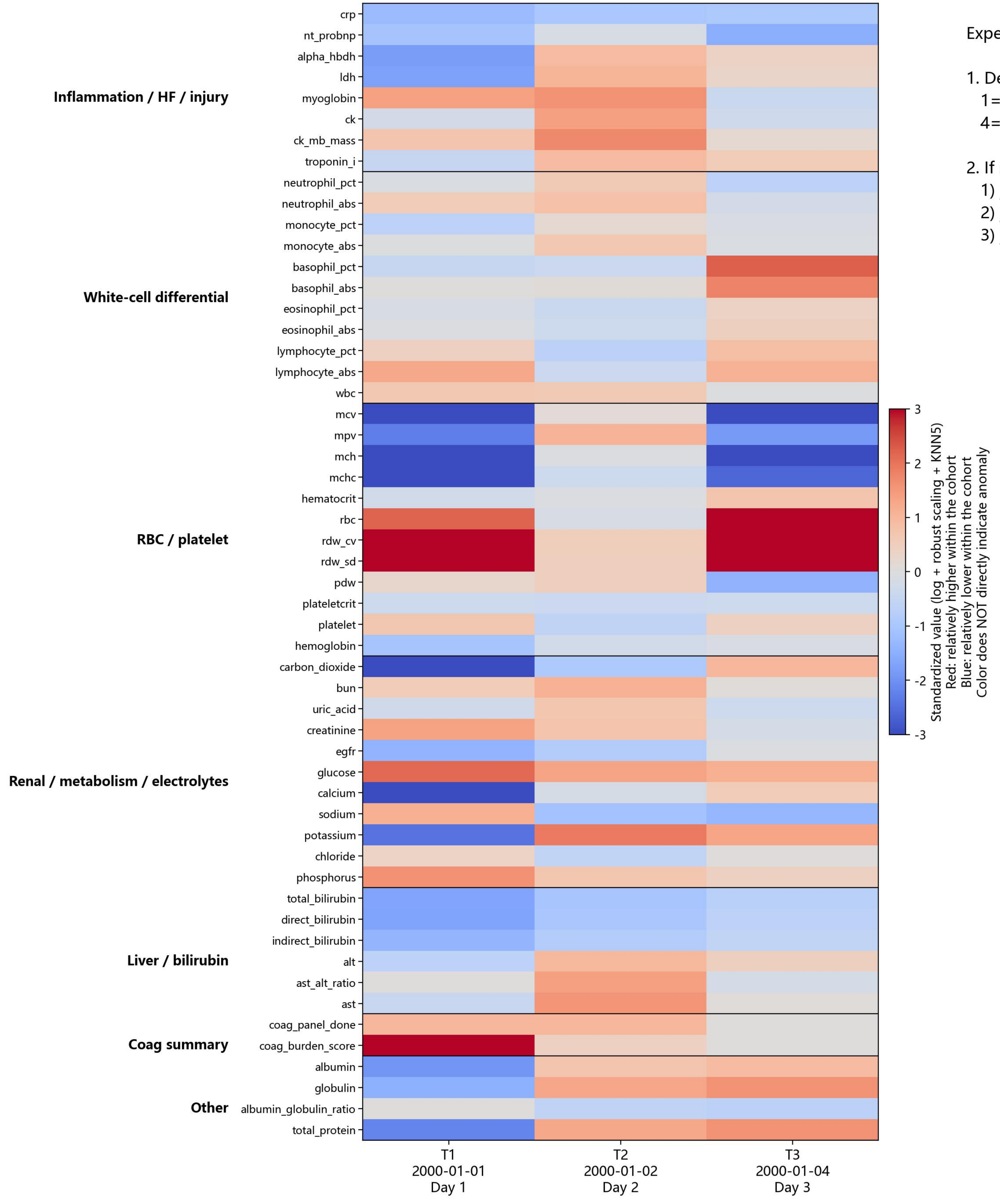

Expert review (blinded; no model score shown)

1. Degree of anomaly for this 3-point window (1-5):
   1=very typical; 2=relatively typical; 3=gray zone;
   4=relatively abnormal; 5=very abnormal

2. If scored 4-5, list the 3 most abnormal / noteworthy variables:
   1) ____________________________
   2) ____________________________
   3) ____________________________

Patient-window heatmap card for blinded expert review
ID: P108   Window: W01

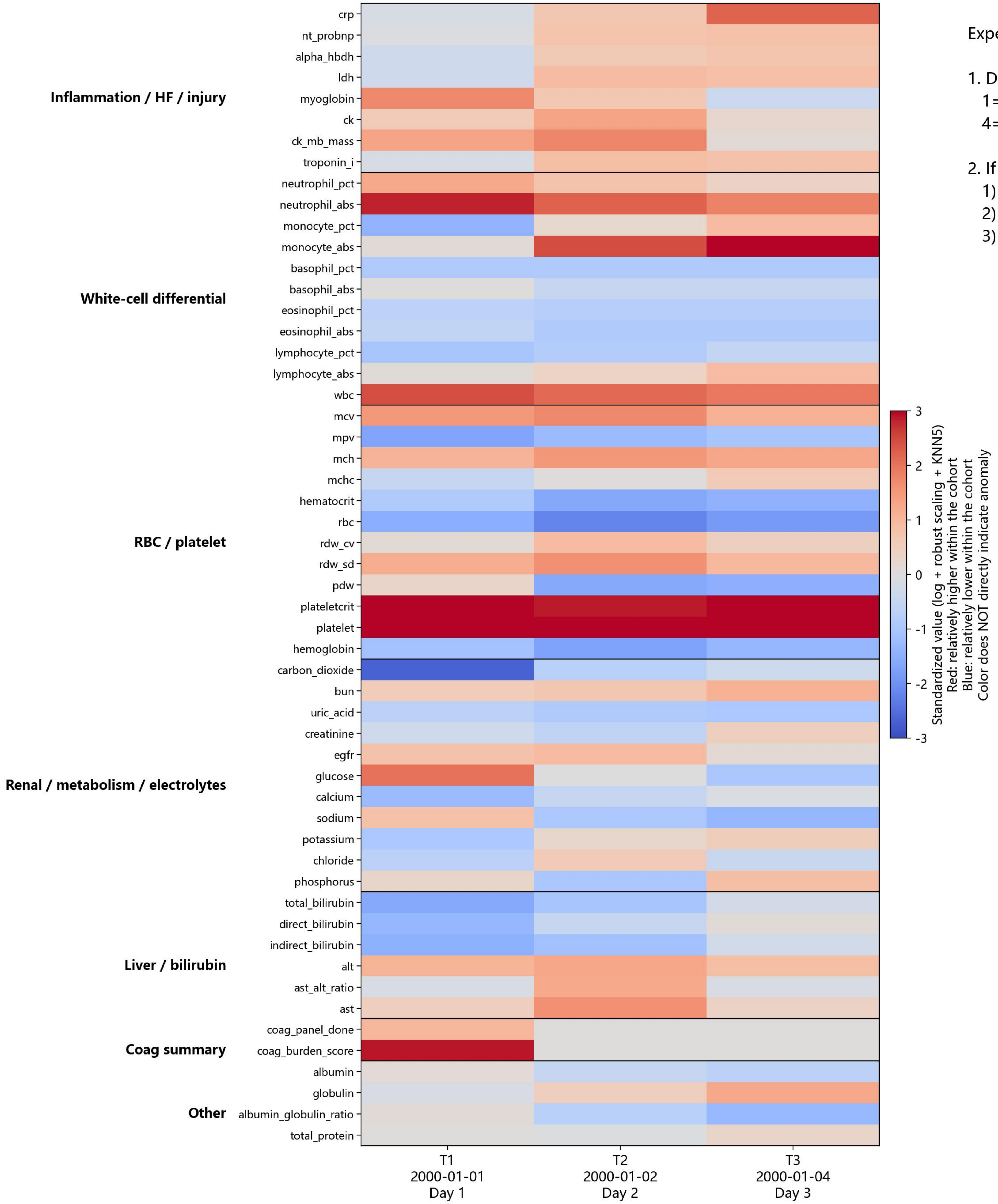

Expert review (blinded; no model score shown)

1. Degree of anomaly for this 3-point window (1-5):
   1=very typical; 2=relatively typical; 3=gray zone;
   4=relatively abnormal; 5=very abnormal

2. If scored 4-5, list the 3 most abnormal / noteworthy variables:
   1) ____________________
   2) ____________________
   3) ____________________

Patient-window heatmap card for blinded expert review
ID: P109   Window: W01

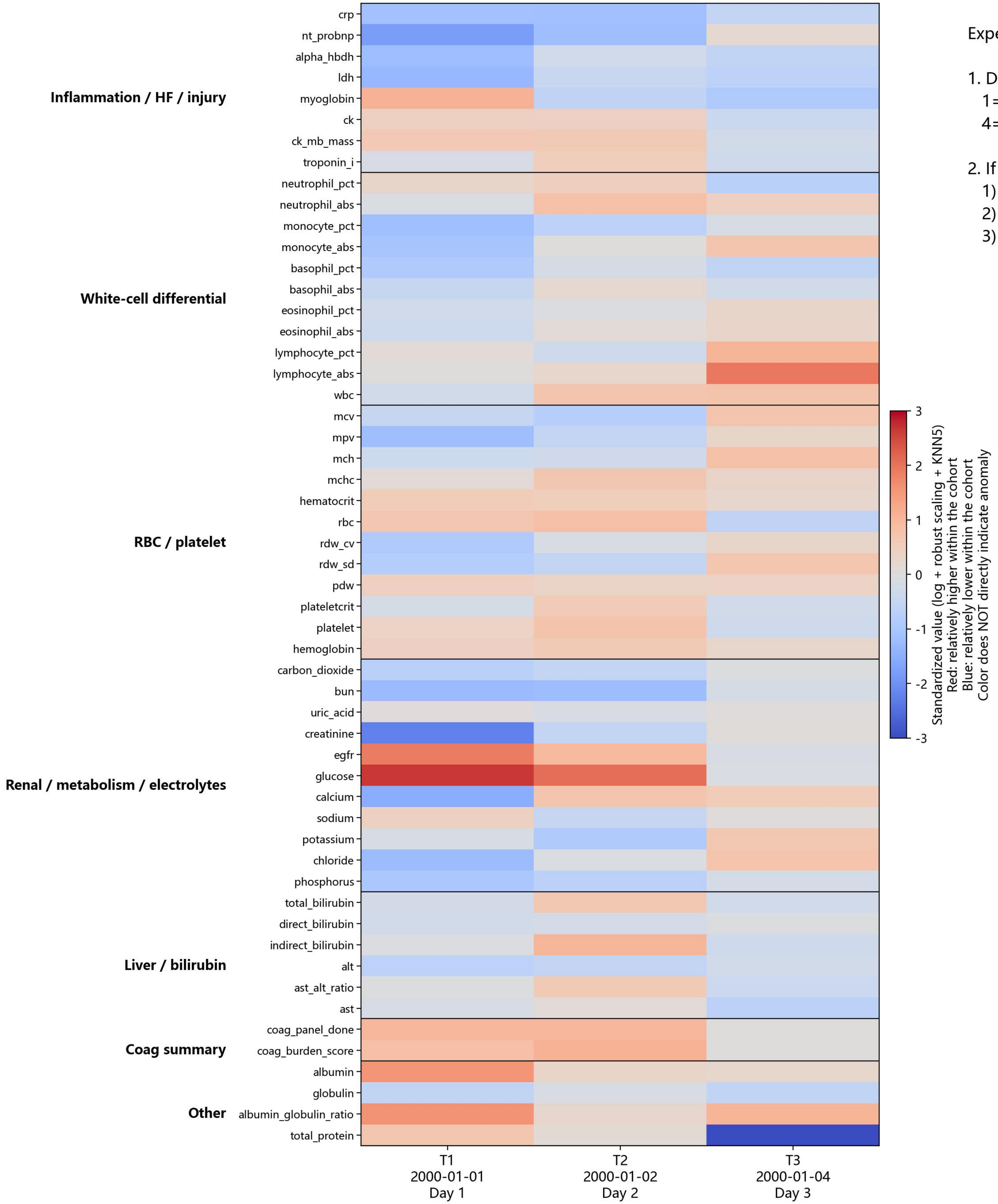

Expert review (blinded; no model score shown)

1. Degree of anomaly for this 3-point window (1-5):
   1=very typical; 2=relatively typical; 3=gray zone;
   4=relatively abnormal; 5=very abnormal

2. If scored 4-5, list the 3 most abnormal / noteworthy variables:
   1) ______________________
   2) ______________________
   3) ______________________

Patient-window heatmap card for blinded expert review
ID: P110   Window: W01

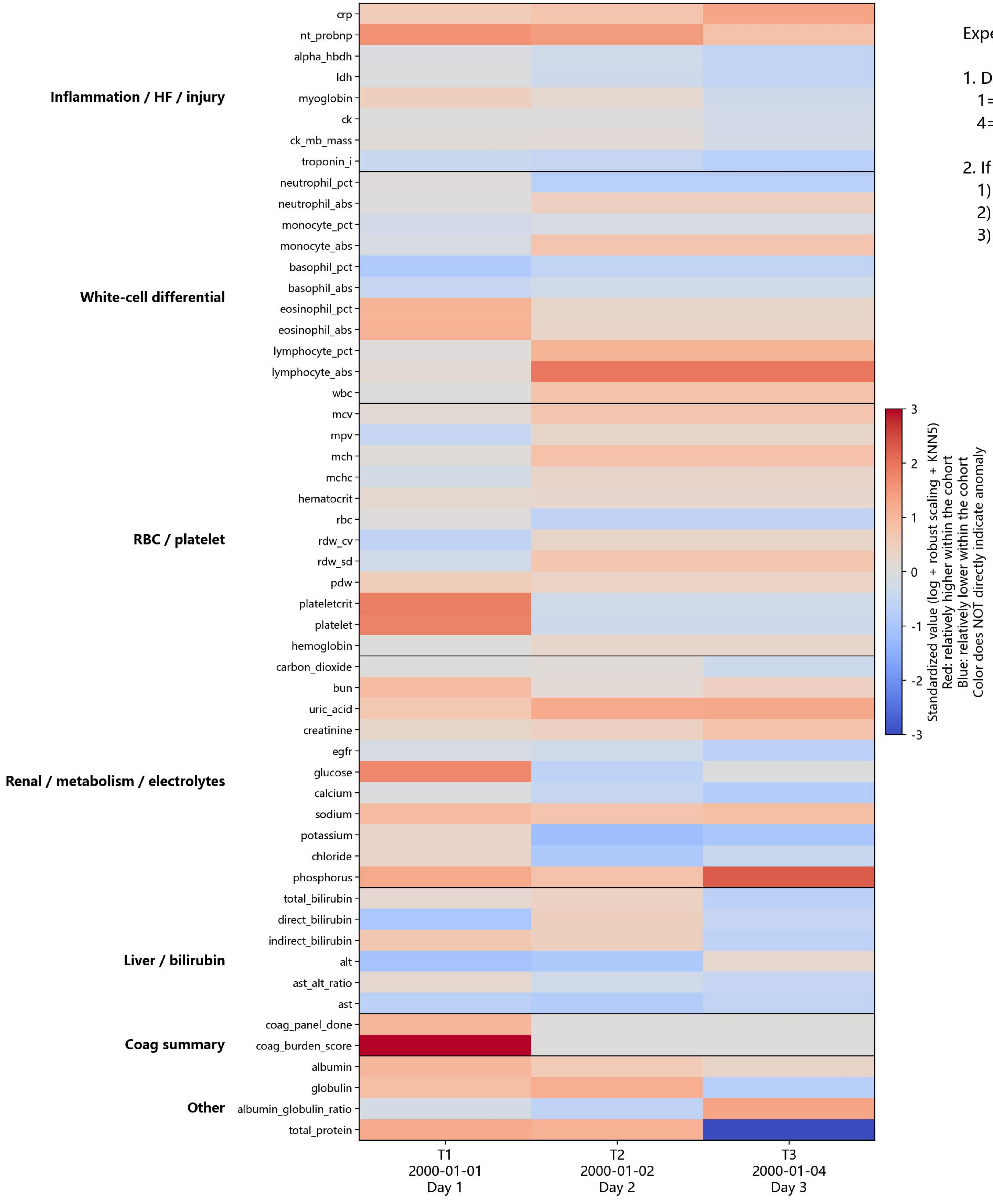

Expert review (blinded; no model score shown)

1. Degree of anomaly for this 3-point window (1-5):
   1=very typical; 2=relatively typical; 3=gray zone;
   4=relatively abnormal; 5=very abnormal

2. If scored 4-5, list the 3 most abnormal / noteworthy variables:
   1) ________________________
   2) ________________________
   3) ________________________

Patient-window heatmap card for blinded expert review
ID: P111 Window: W01

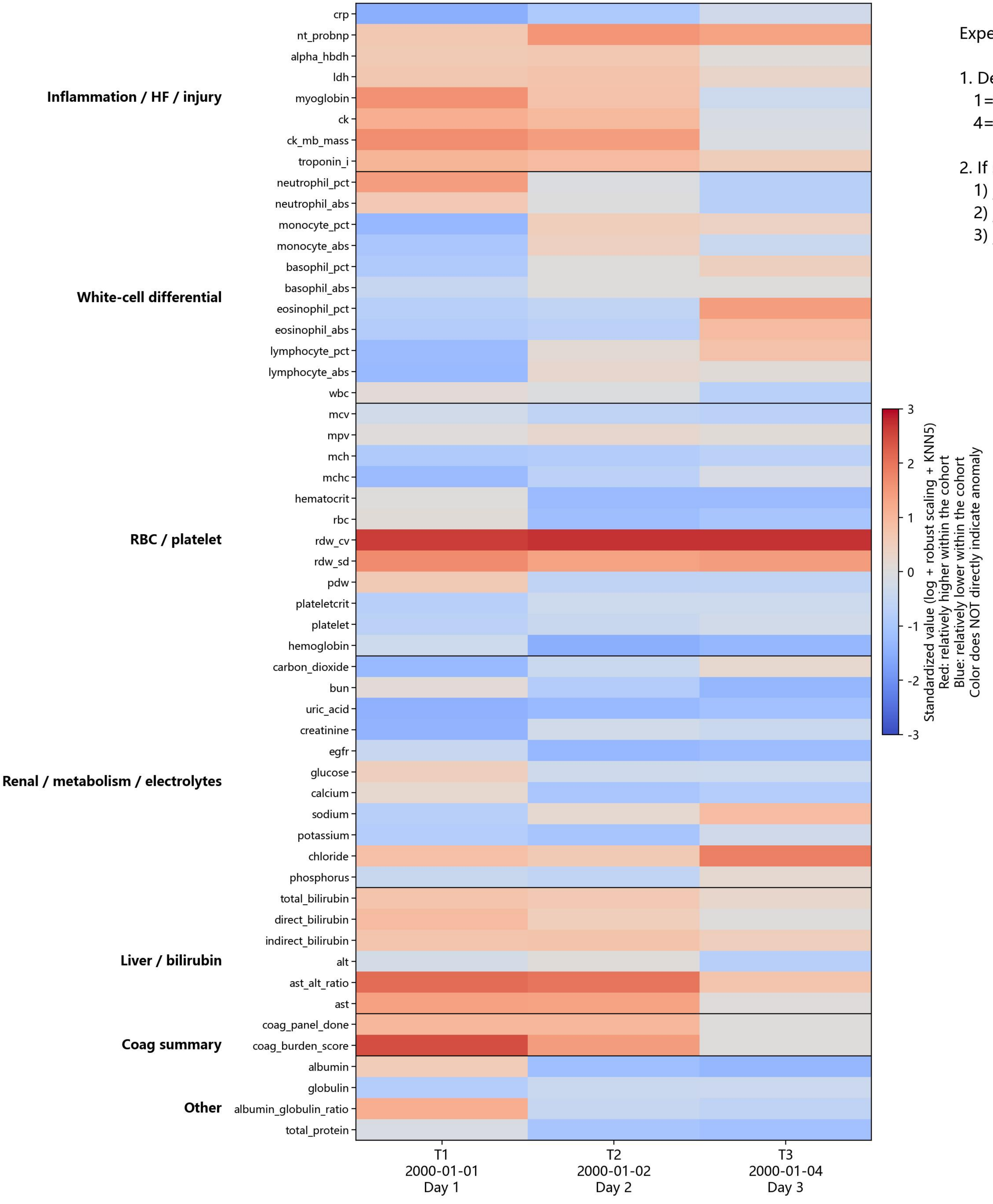

Expert review (blinded; no model score shown)

1. Degree of anomaly for this 3-point window (1-5):
   1=very typical; 2=relatively typical; 3=gray zone;
   4=relatively abnormal; 5=very abnormal

2. If scored 4-5, list the 3 most abnormal / noteworthy variables:
   1) ________________________
   2) ________________________
   3) ________________________

Patient-window heatmap card for blinded expert review
ID: P112   Window: W01

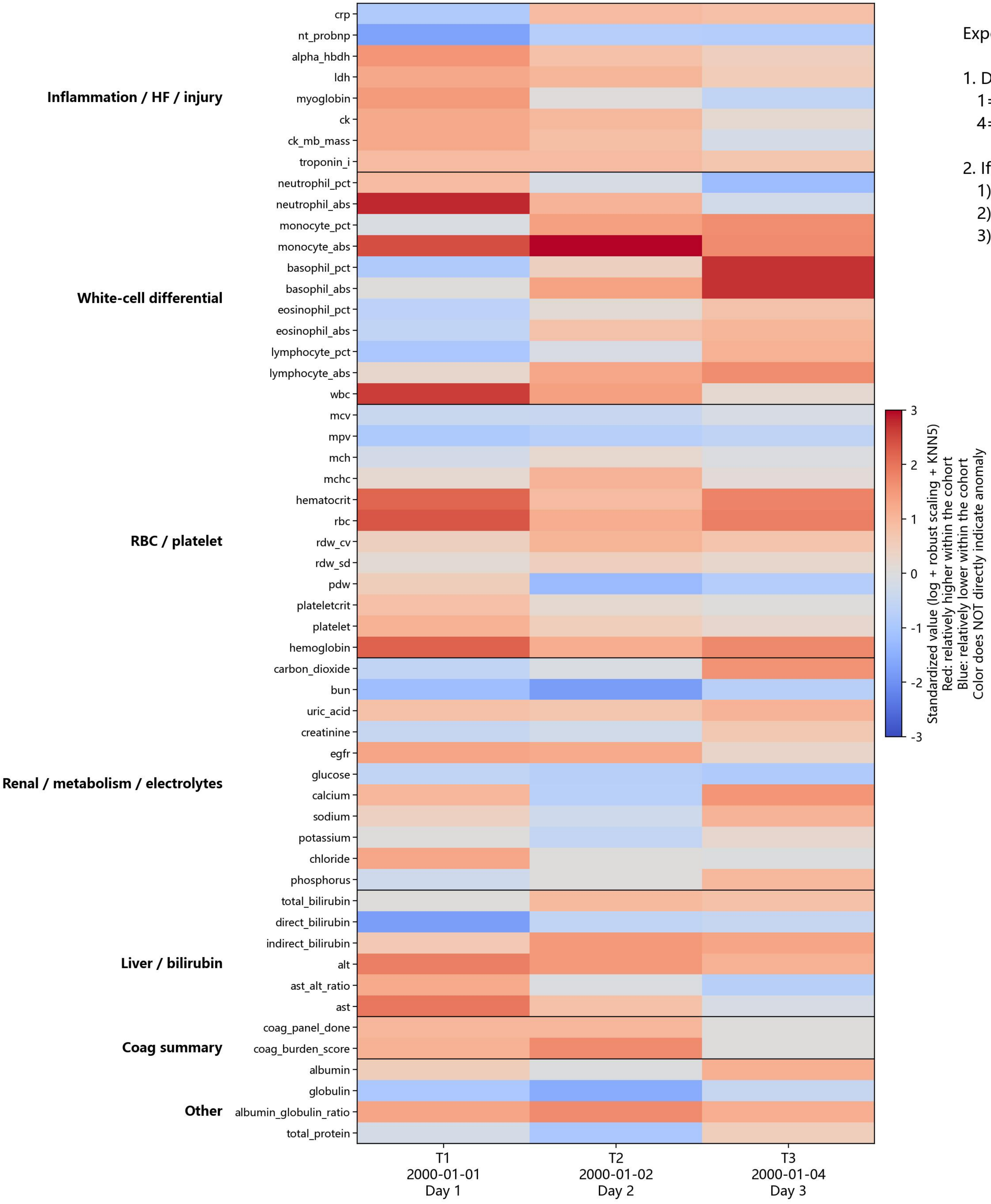

Expert review (blinded; no model score shown)

1. Degree of anomaly for this 3-point window (1-5):
   1=very typical; 2=relatively typical; 3=gray zone;
   4=relatively abnormal; 5=very abnormal

2. If scored 4-5, list the 3 most abnormal / noteworthy variables:
   1) ________________________
   2) ________________________
   3) ________________________

Patient-window heatmap card for blinded expert review
ID: P113   Window: W01

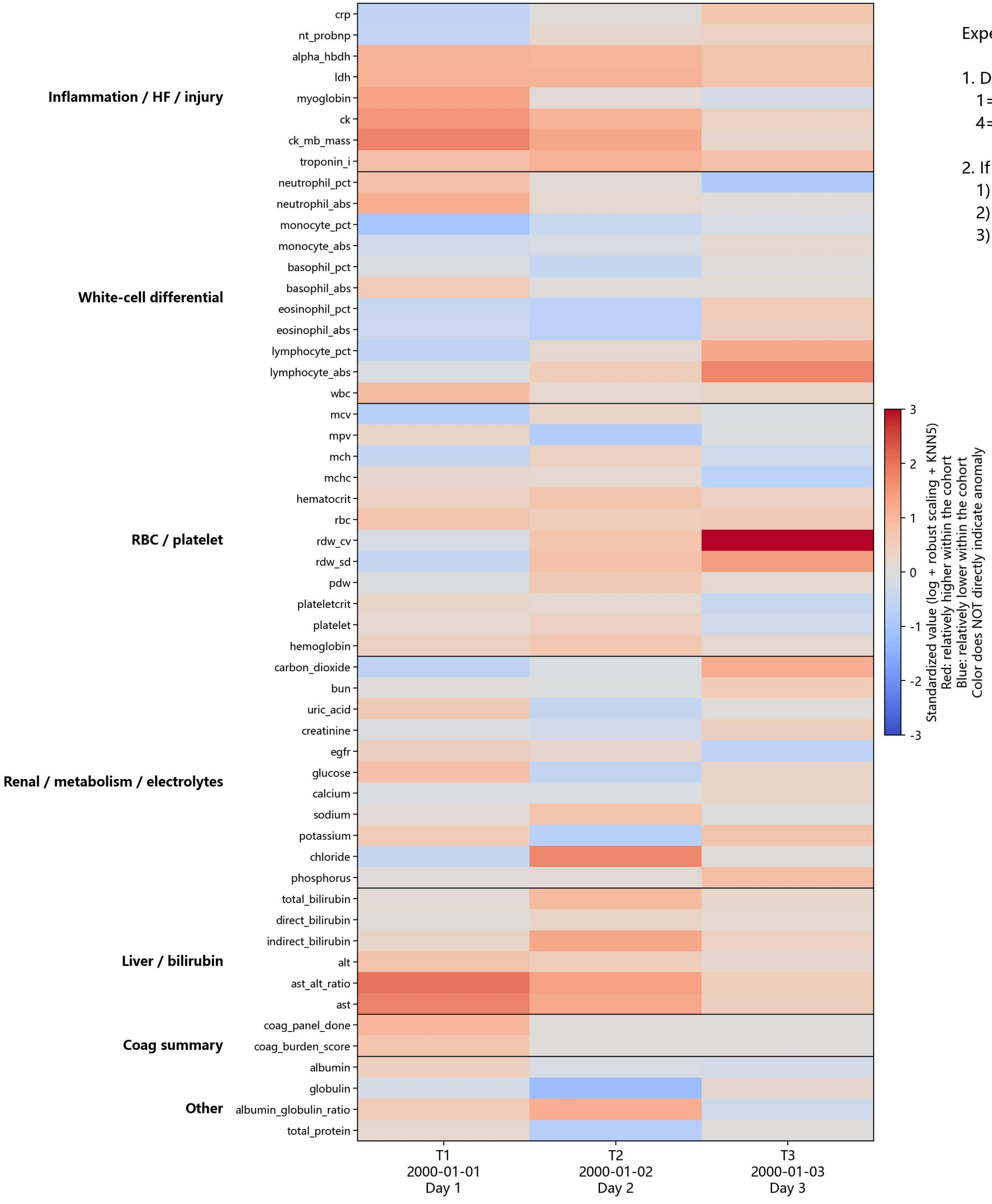

Expert review (blinded; no model score shown)

1. Degree of anomaly for this 3-point window (1-5):
   1=very typical; 2=relatively typical; 3=gray zone;
   4=relatively abnormal; 5=very abnormal

2. If scored 4-5, list the 3 most abnormal / noteworthy variables:
   1) ____________________________
   2) ____________________________
   3) ____________________________

Patient-window heatmap card for blinded expert review
ID: P114   Window: W01

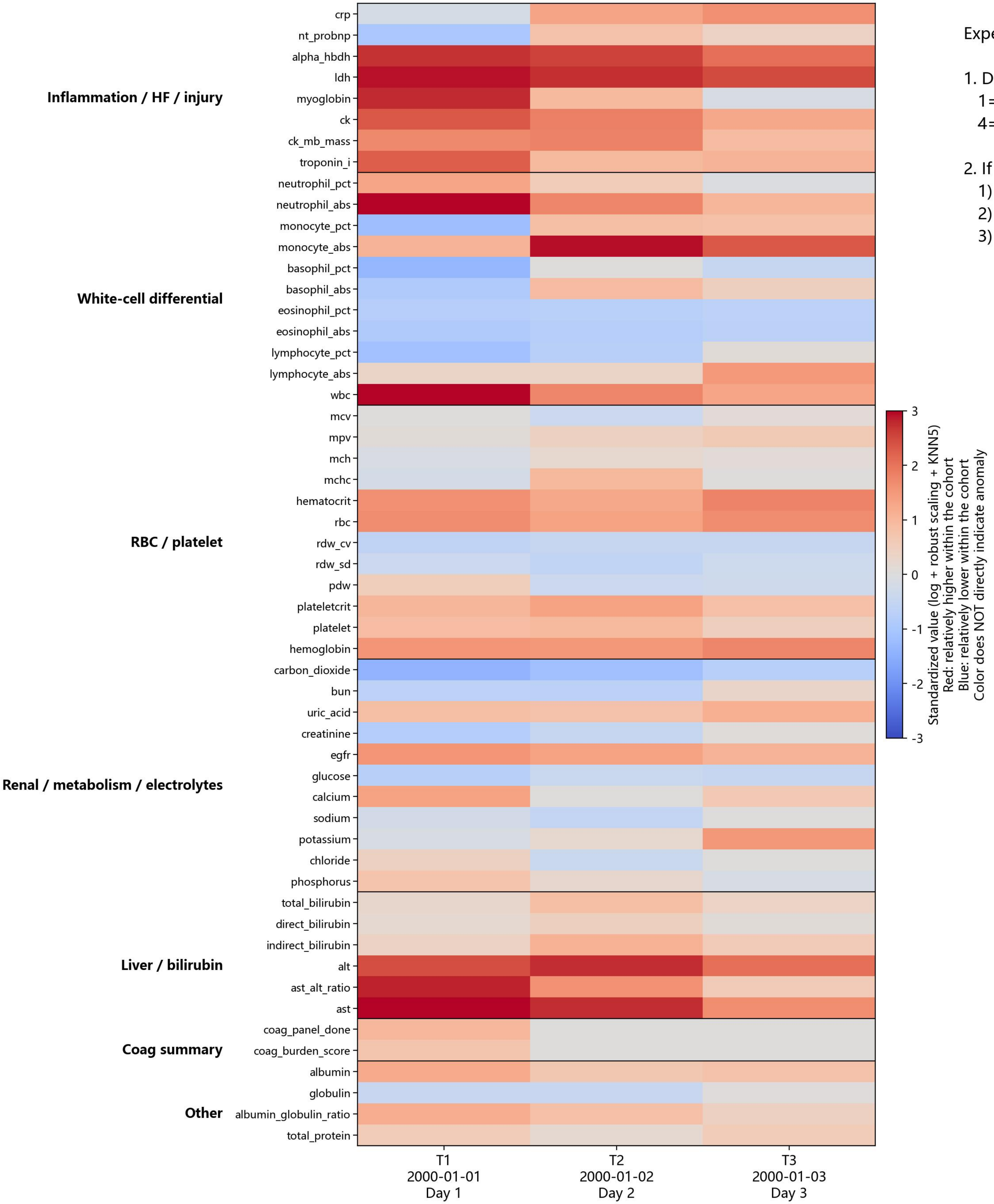

Expert review (blinded; no model score shown)

1. Degree of anomaly for this 3-point window (1-5):
   1=very typical; 2=relatively typical; 3=gray zone;
   4=relatively abnormal; 5=very abnormal

2. If scored 4-5, list the 3 most abnormal / noteworthy variables:
   1) ________________________
   2) ________________________
   3) ________________________

Patient-window heatmap card for blinded expert review
ID: P115   Window: W01

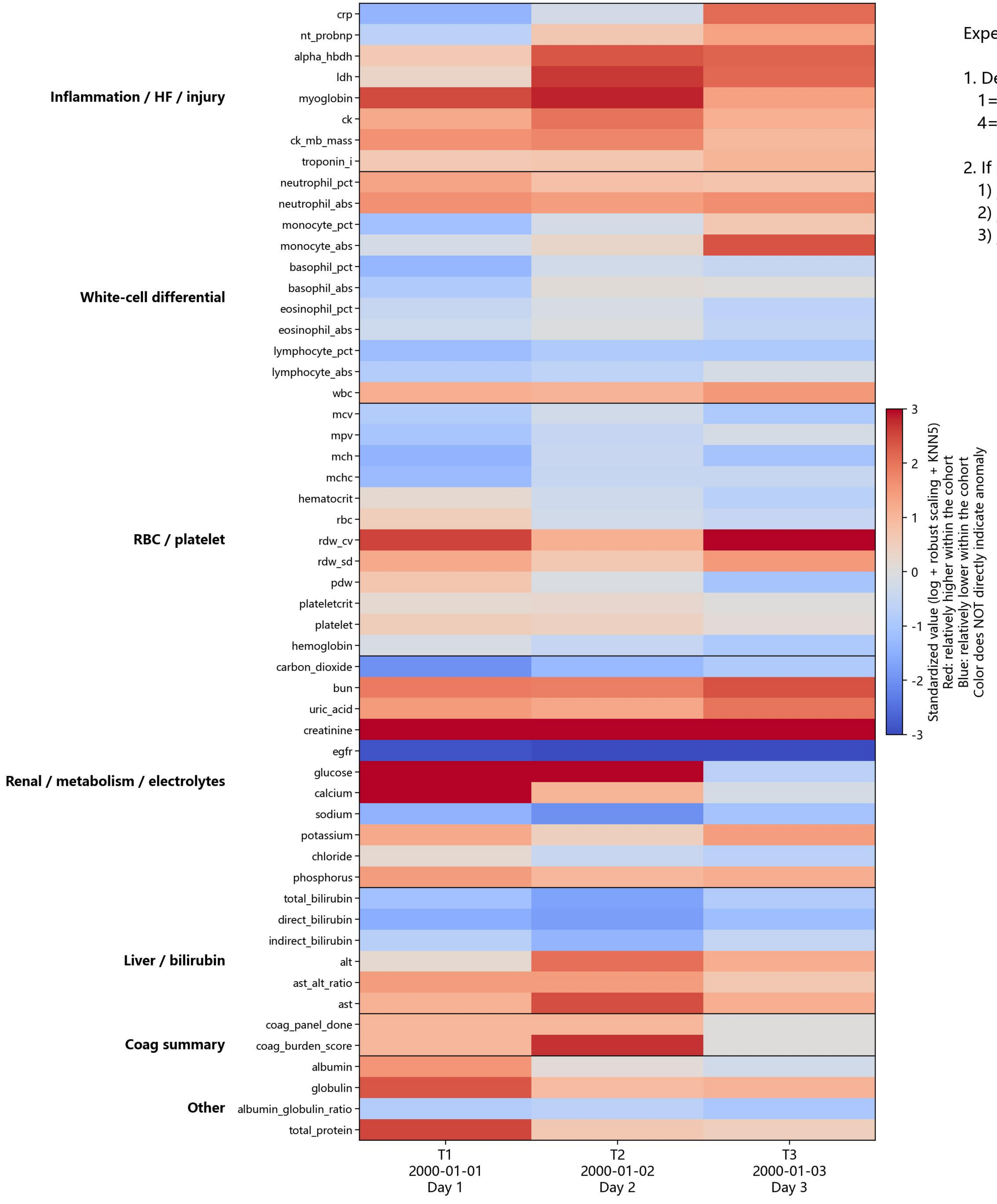

Expert review (blinded; no model score shown)

1. Degree of anomaly for this 3-point window (1-5):
   1=very typical; 2=relatively typical; 3=gray zone;
   4=relatively abnormal; 5=very abnormal

2. If scored 4-5, list the 3 most abnormal / noteworthy variables:
   1) ________________________
   2) ________________________
   3) ________________________

Patient-window heatmap card for blinded expert review
ID: P116 Window: W01

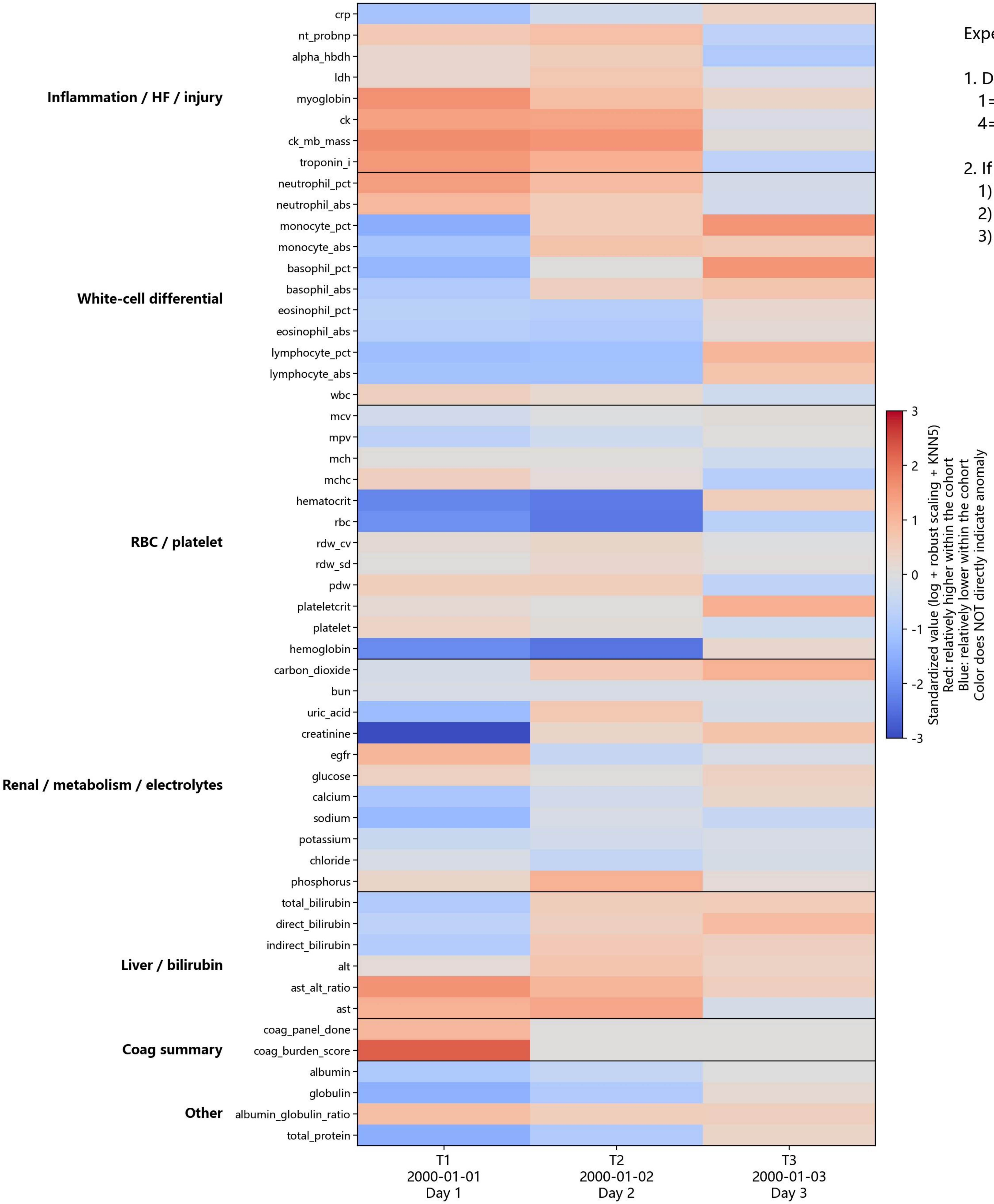

Expert review (blinded; no model score shown)

1. Degree of anomaly for this 3-point window (1-5):
1=very typical; 2=relatively typical; 3=gray zone;
4=relatively abnormal; 5=very abnormal

2. If scored 4-5, list the 3 most abnormal / noteworthy variables:
1) ________________________
2) ________________________
3) ________________________

Patient-window heatmap card for blinded expert review
ID: P117 Window: W01

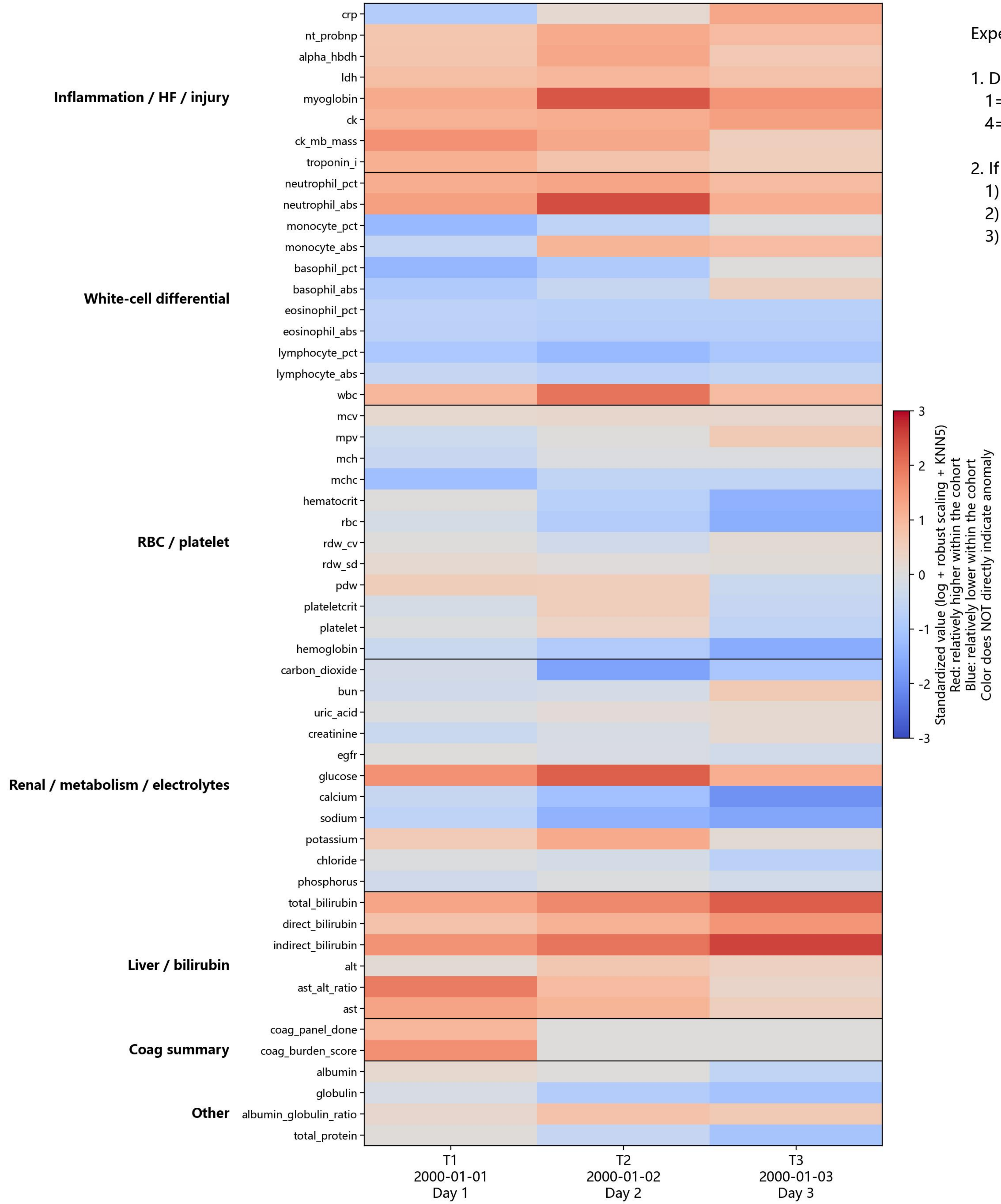

Expert review (blinded; no model score shown)

1. Degree of anomaly for this 3-point window (1-5):
   1=very typical; 2=relatively typical; 3=gray zone;
   4=relatively abnormal; 5=very abnormal

2. If scored 4-5, list the 3 most abnormal / noteworthy variables:
   1) ____________________
   2) ____________________
   3) ____________________

Patient-window heatmap card for blinded expert review
ID: P118 Window: W01

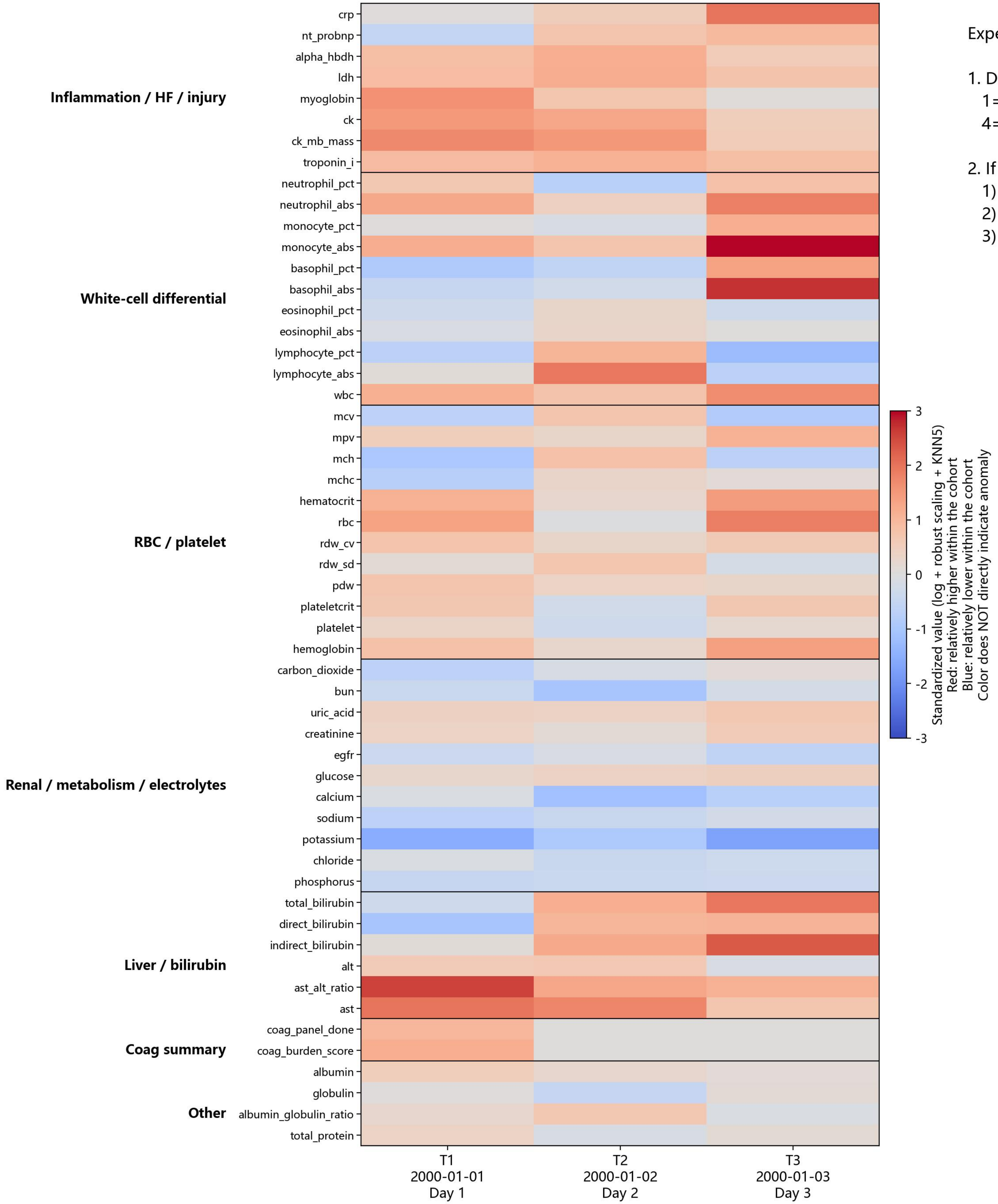

Expert review (blinded; no model score shown)

1. Degree of anomaly for this 3-point window (1-5):
   1=very typical; 2=relatively typical; 3=gray zone;
   4=relatively abnormal; 5=very abnormal

2. If scored 4-5, list the 3 most abnormal / noteworthy variables:
   1) ________________________
   2) ________________________
   3) ________________________

Patient-window heatmap card for blinded expert review
ID: P119   Window: W01

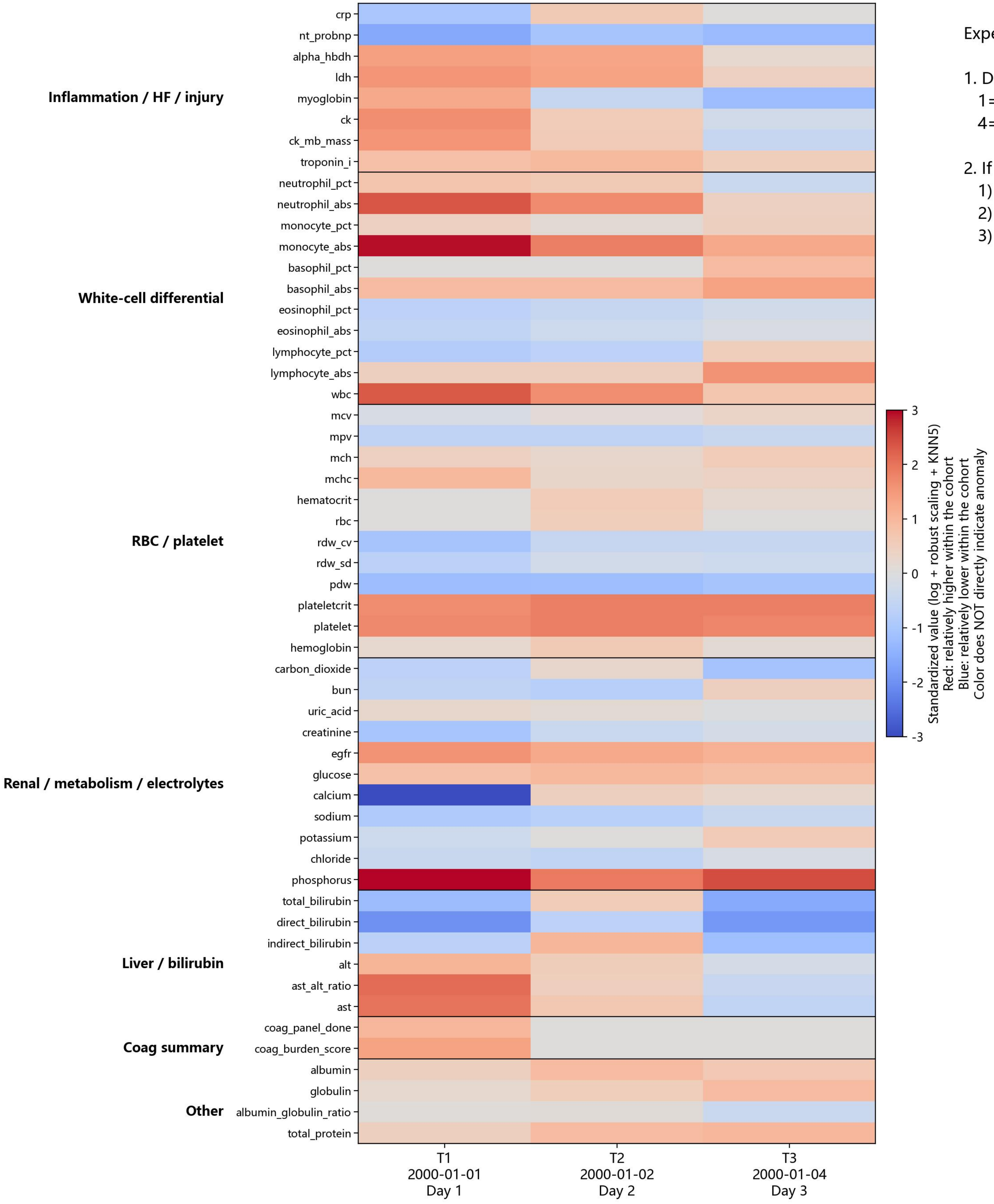

Expert review (blinded; no model score shown)

1. Degree of anomaly for this 3-point window (1-5):
   1=very typical; 2=relatively typical; 3=gray zone;
   4=relatively abnormal; 5=very abnormal

2. If scored 4-5, list the 3 most abnormal / noteworthy variables:
   1) ____________________________
   2) ____________________________
   3) ____________________________

Patient-window heatmap card for blinded expert review
ID: P120   Window: W01

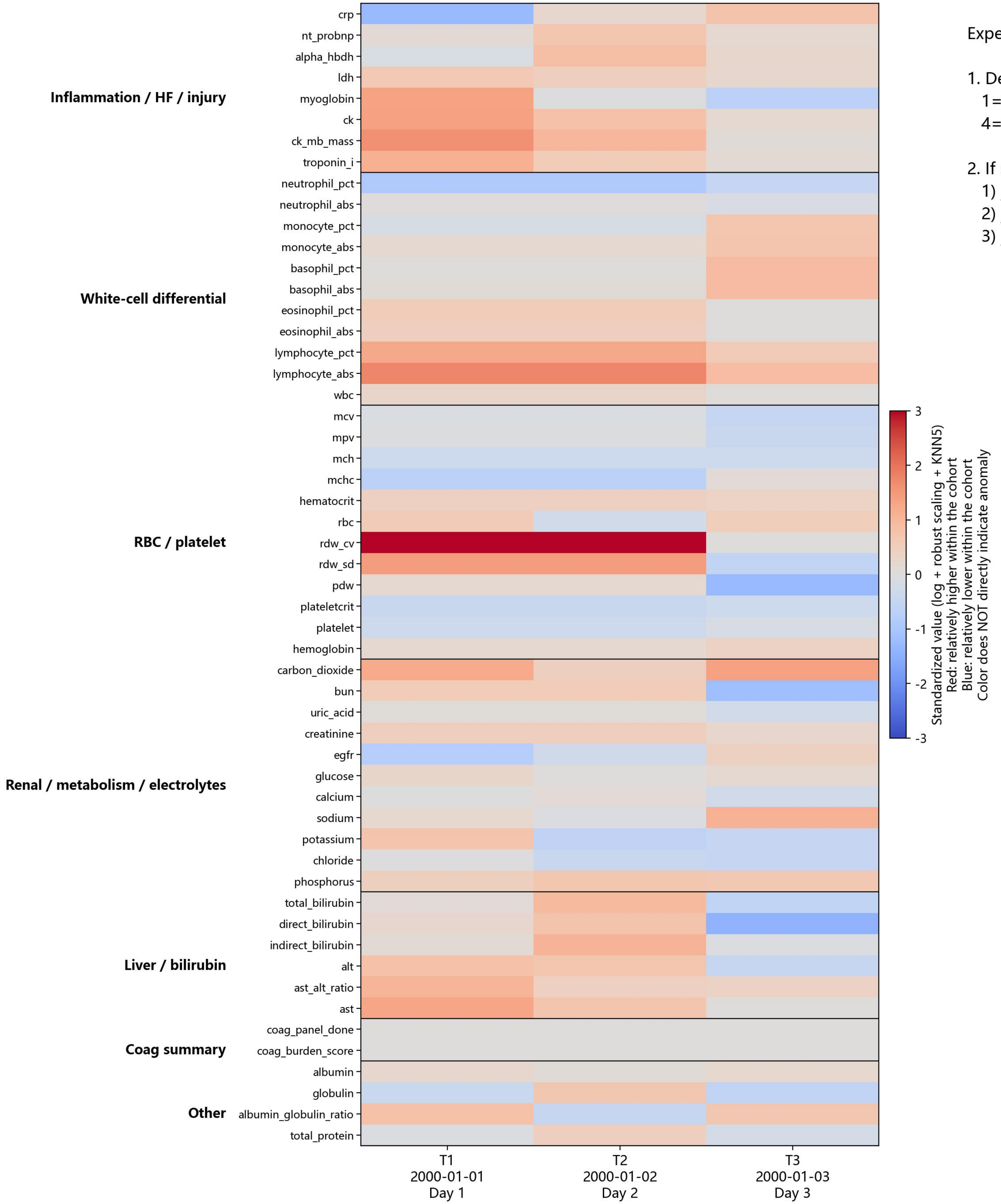

Expert review (blinded; no model score shown)

1. Degree of anomaly for this 3-point window (1-5):
   1=very typical; 2=relatively typical; 3=gray zone;
   4=relatively abnormal; 5=very abnormal

2. If scored 4-5, list the 3 most abnormal / noteworthy variables:
   1) ________________________
   2) ________________________
   3) ________________________

Patient-window heatmap card for blinded expert review
ID: P121   Window: W01

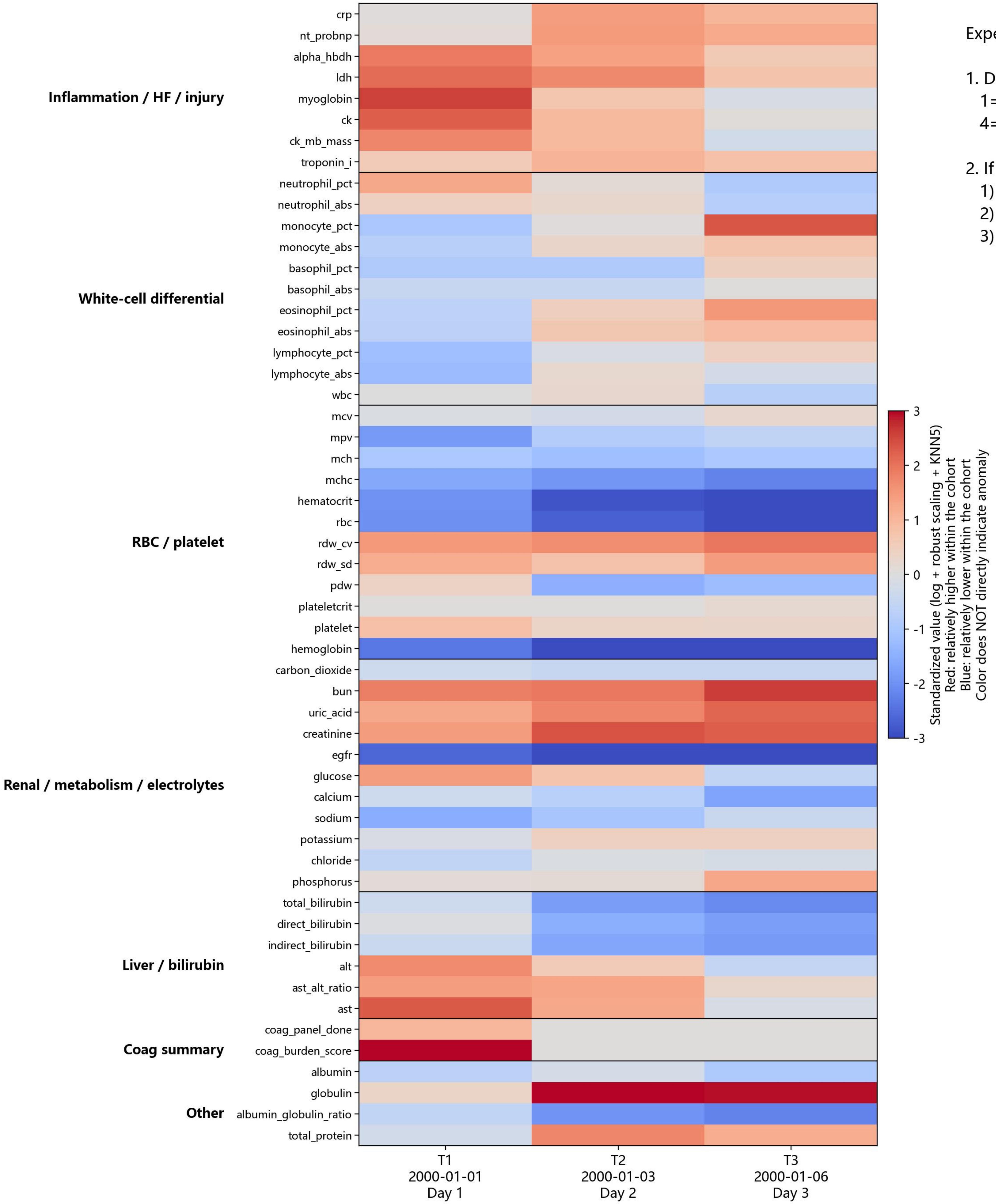

Expert review (blinded; no model score shown)

1. Degree of anomaly for this 3-point window (1-5):
   1=very typical; 2=relatively typical; 3=gray zone;
   4=relatively abnormal; 5=very abnormal

2. If scored 4-5, list the 3 most abnormal / noteworthy variables:
   1) ________________________
   2) ________________________
   3) ________________________

Patient-window heatmap card for blinded expert review
ID: P122 Window: W01

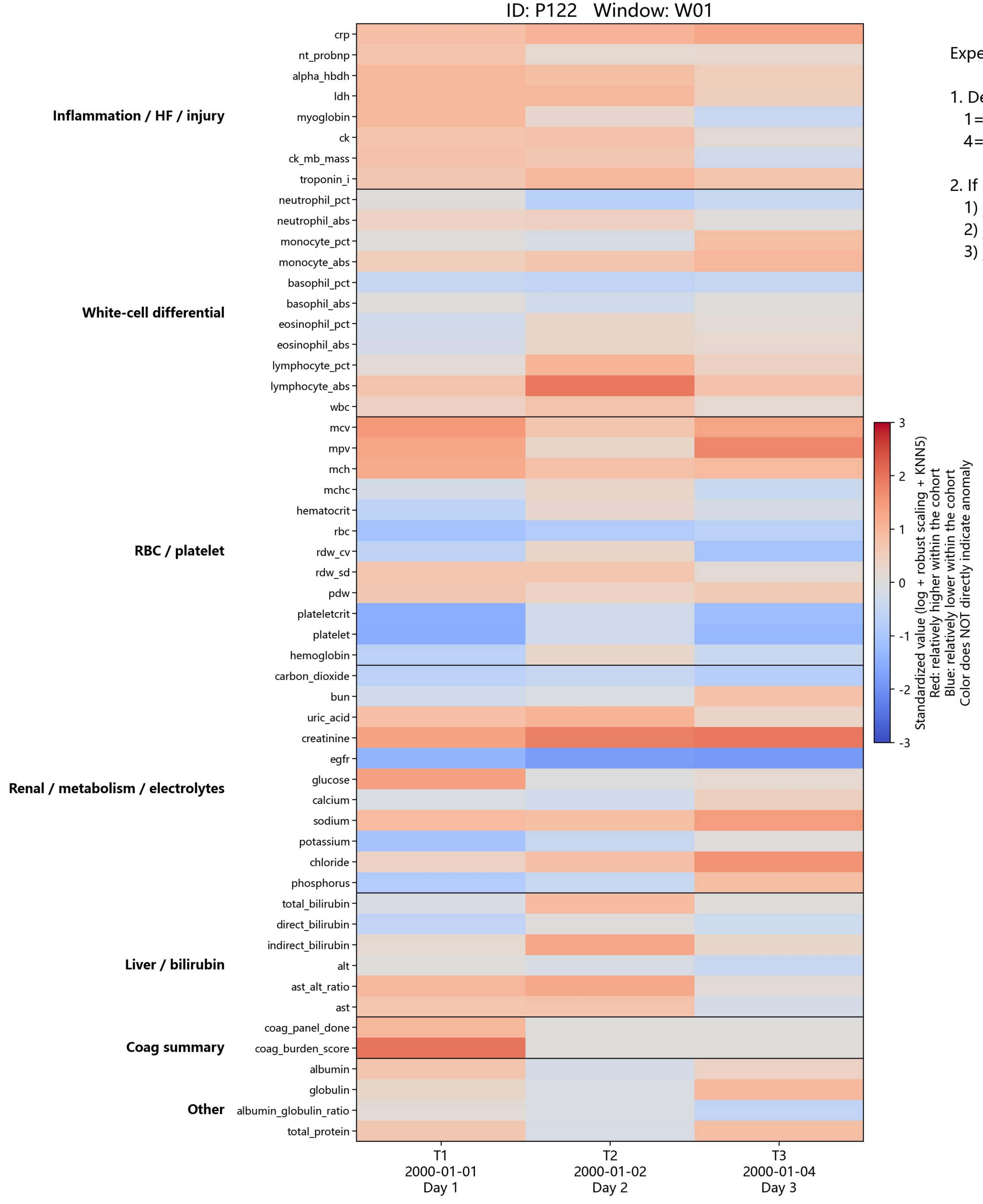

Expert review (blinded; no model score shown)

1. Degree of anomaly for this 3-point window (1-5):
   1=very typical; 2=relatively typical; 3=gray zone;
   4=relatively abnormal; 5=very abnormal

2. If scored 4-5, list the 3 most abnormal / noteworthy variables:
   1) ________________________
   2) ________________________
   3) ________________________

Patient-window heatmap card for blinded expert review
ID: P123   Window: W01

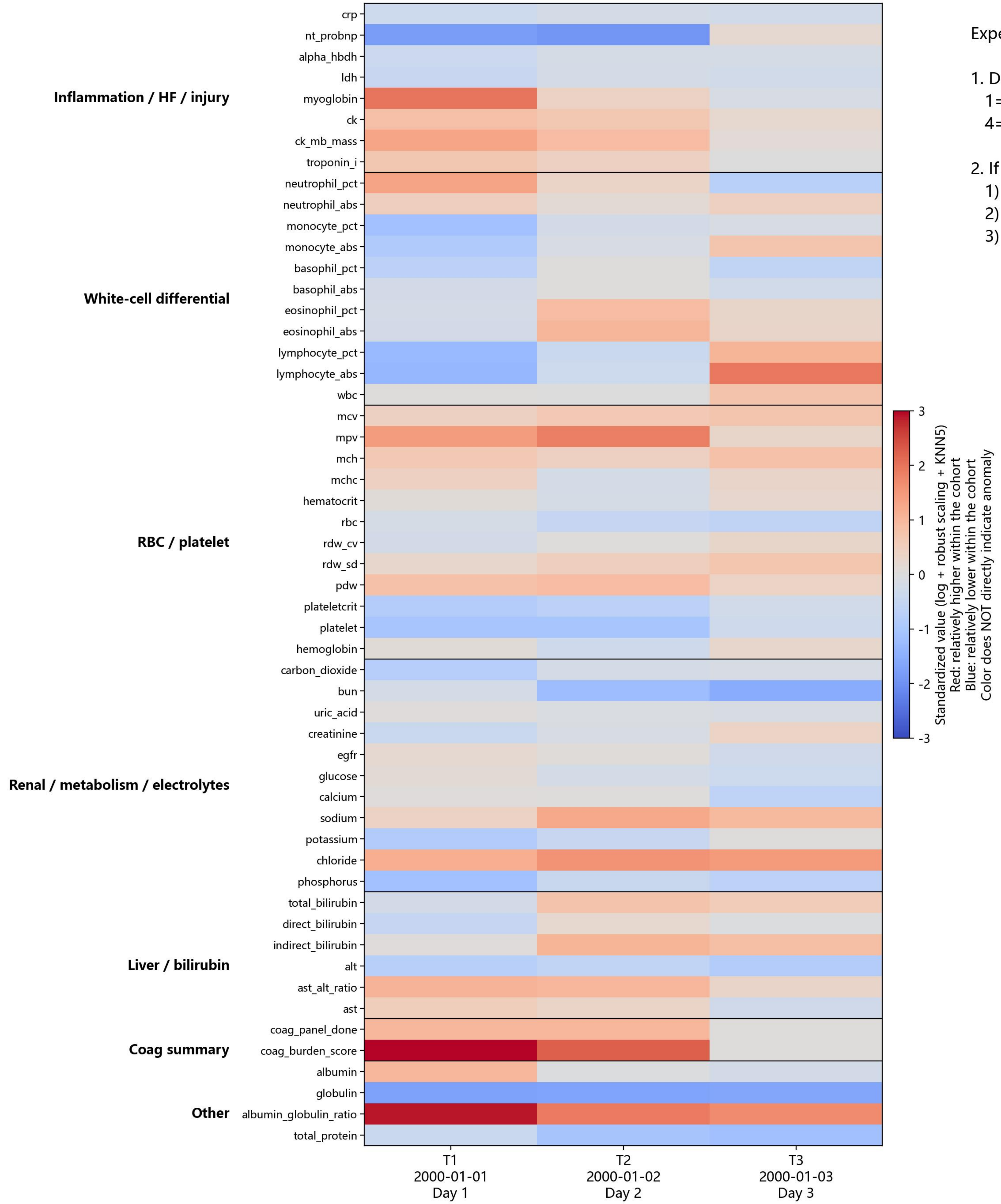

Expert review (blinded; no model score shown)

1. Degree of anomaly for this 3-point window (1-5):
   1=very typical; 2=relatively typical; 3=gray zone;
   4=relatively abnormal; 5=very abnormal

2. If scored 4-5, list the 3 most abnormal / noteworthy variables:
   1) ________________________
   2) ________________________
   3) ________________________

Patient-window heatmap card for blinded expert review
ID: P124   Window: W01

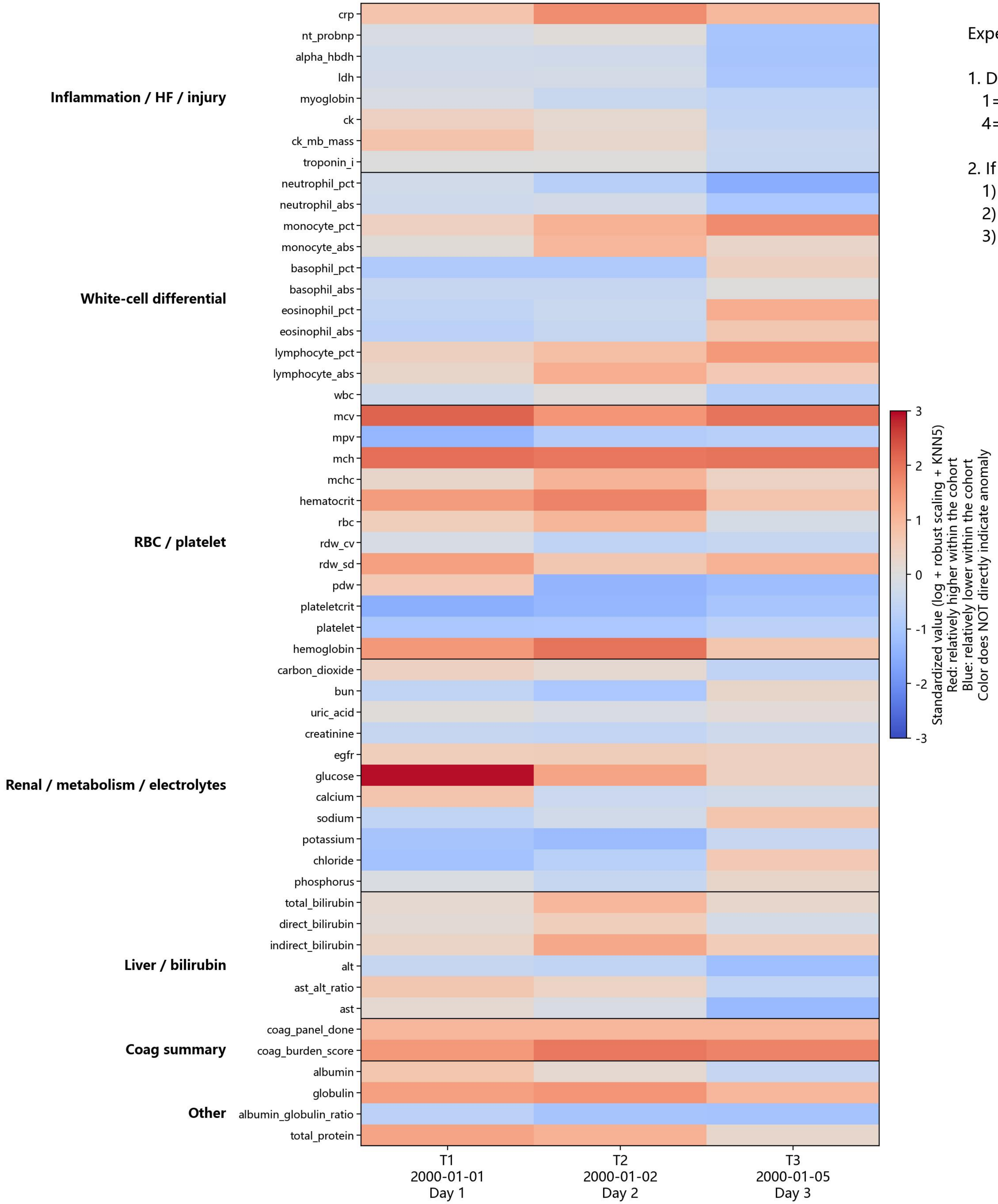

Expert review (blinded; no model score shown)

1. Degree of anomaly for this 3-point window (1-5):
   1=very typical; 2=relatively typical; 3=gray zone;
   4=relatively abnormal; 5=very abnormal

2. If scored 4-5, list the 3 most abnormal / noteworthy variables:
   1) ________________________
   2) ________________________
   3) ________________________

Patient-window heatmap card for blinded expert review
ID: P125 Window: W01

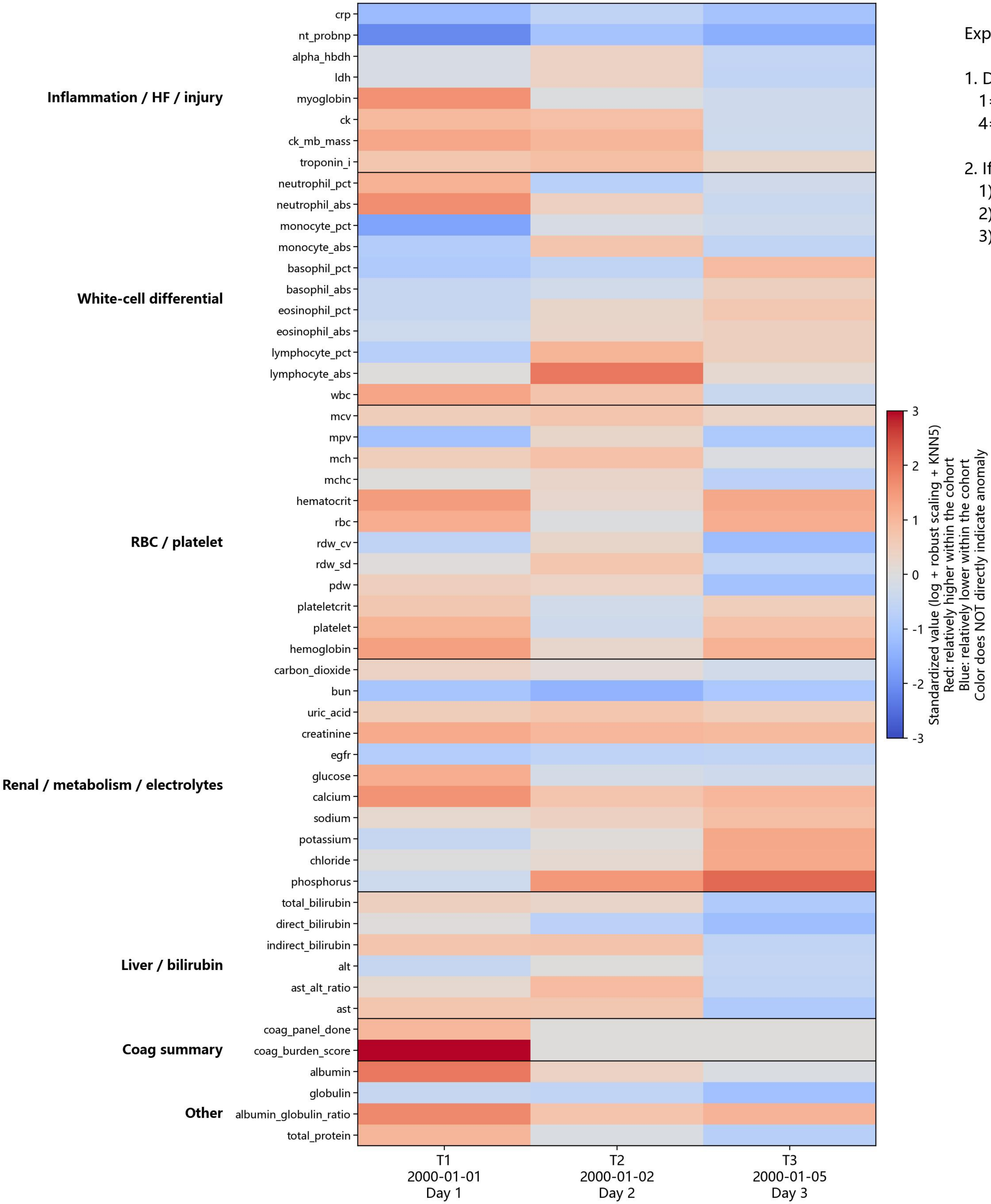

Expert review (blinded; no model score shown)

1. Degree of anomaly for this 3-point window (1-5):
   1=very typical; 2=relatively typical; 3=gray zone;
   4=relatively abnormal; 5=very abnormal

2. If scored 4-5, list the 3 most abnormal / noteworthy variables:
   1) ________________________
   2) ________________________
   3) ________________________

Patient-window heatmap card for blinded expert review
ID: P126   Window: W01

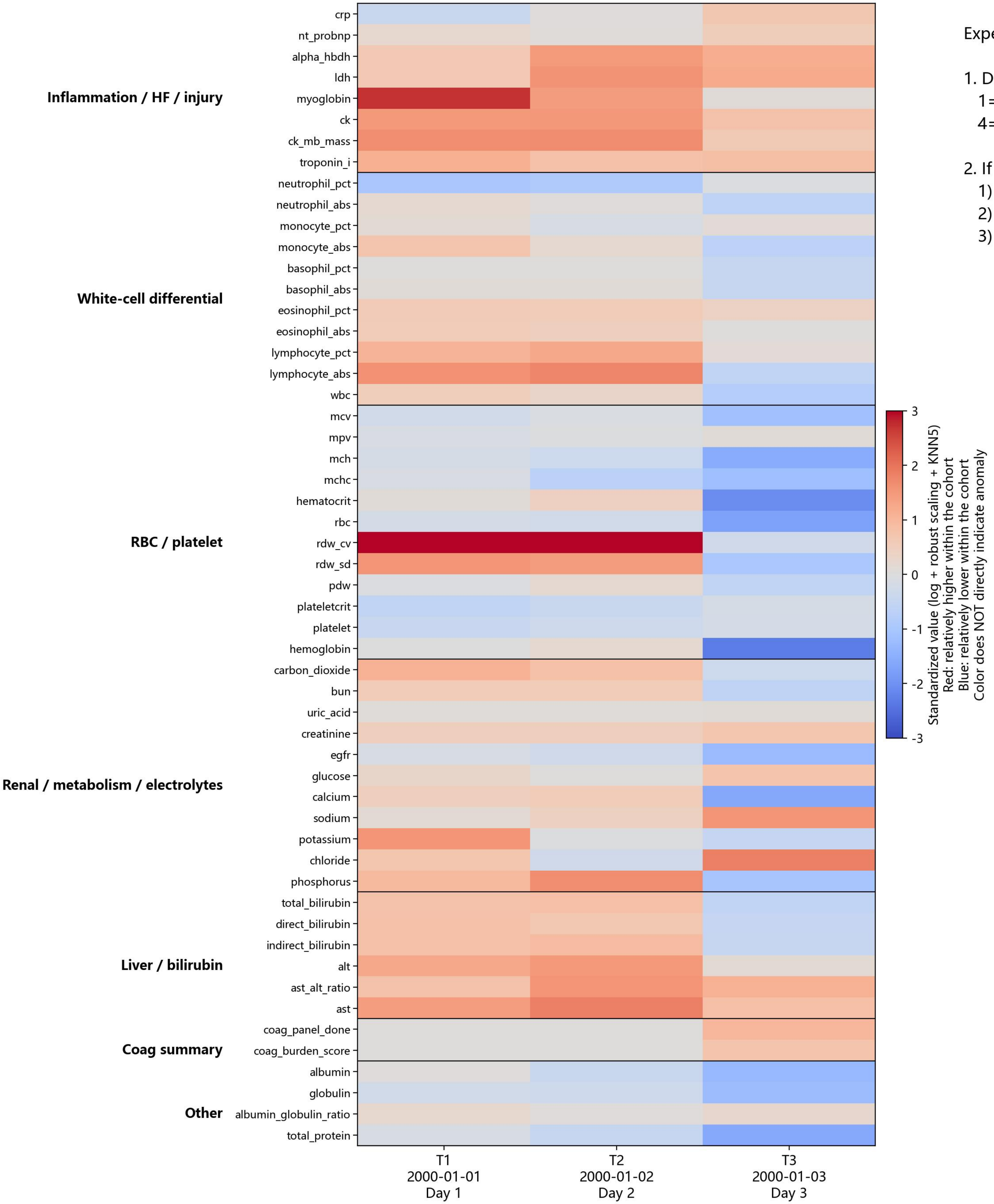

Expert review (blinded; no model score shown)

1. Degree of anomaly for this 3-point window (1-5):
   1=very typical; 2=relatively typical; 3=gray zone;
   4=relatively abnormal; 5=very abnormal

2. If scored 4-5, list the 3 most abnormal / noteworthy variables:
   1) ______________________
   2) ______________________
   3) ______________________

Patient-window heatmap card for blinded expert review
ID: P127   Window: W01

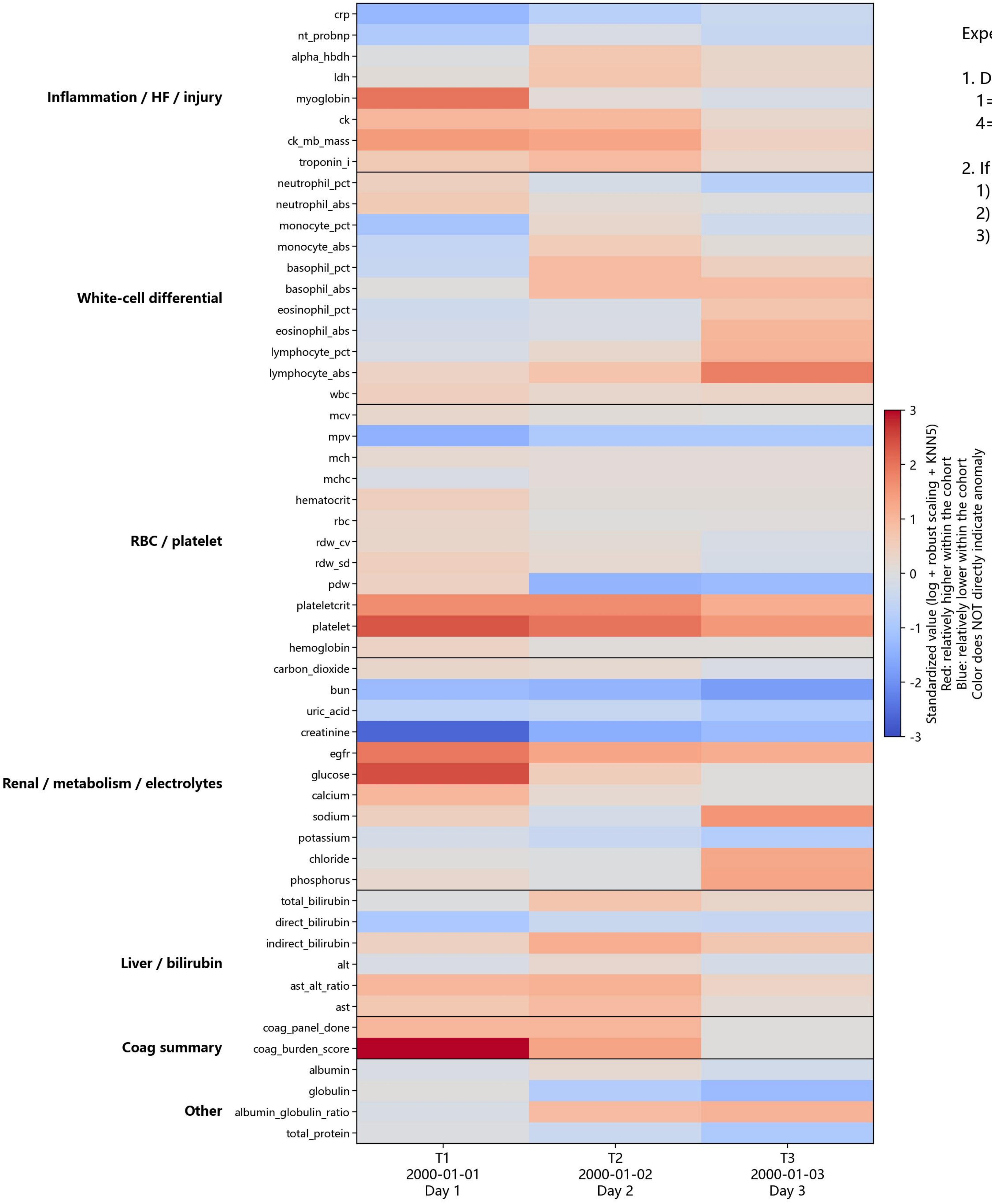

Expert review (blinded; no model score shown)

1. Degree of anomaly for this 3-point window (1-5):
   1=very typical; 2=relatively typical; 3=gray zone;
   4=relatively abnormal; 5=very abnormal

2. If scored 4-5, list the 3 most abnormal / noteworthy variables:
   1) ___________________________
   2) ___________________________
   3) ___________________________

Patient-window heatmap card for blinded expert review
ID: P128   Window: W01

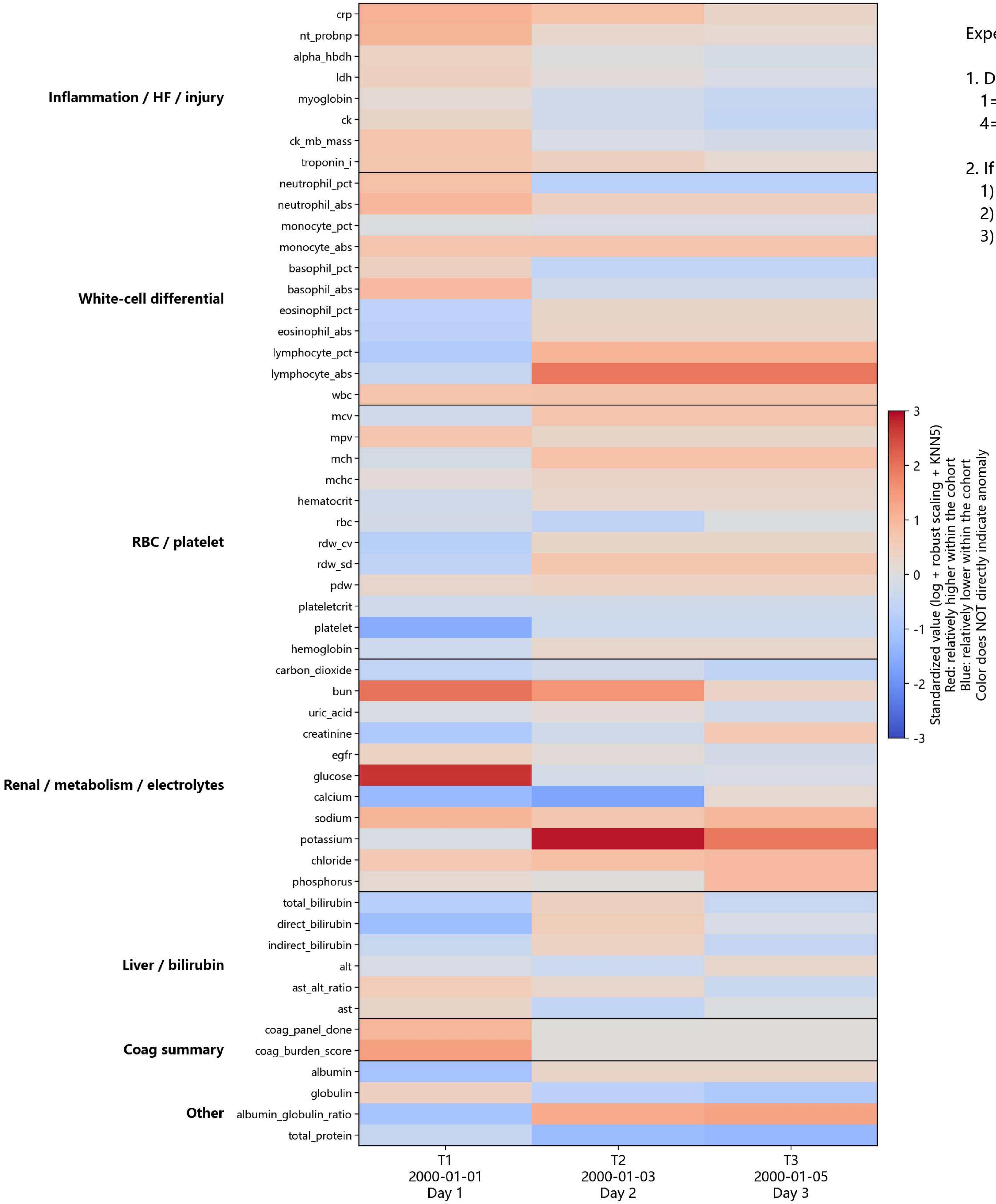

Expert review (blinded; no model score shown)

1. Degree of anomaly for this 3-point window (1-5):
   1=very typical; 2=relatively typical; 3=gray zone;
   4=relatively abnormal; 5=very abnormal

2. If scored 4-5, list the 3 most abnormal / noteworthy variables:
   1) ____________________
   2) ____________________
   3) ____________________

Patient-window heatmap card for blinded expert review
ID: P129   Window: W01

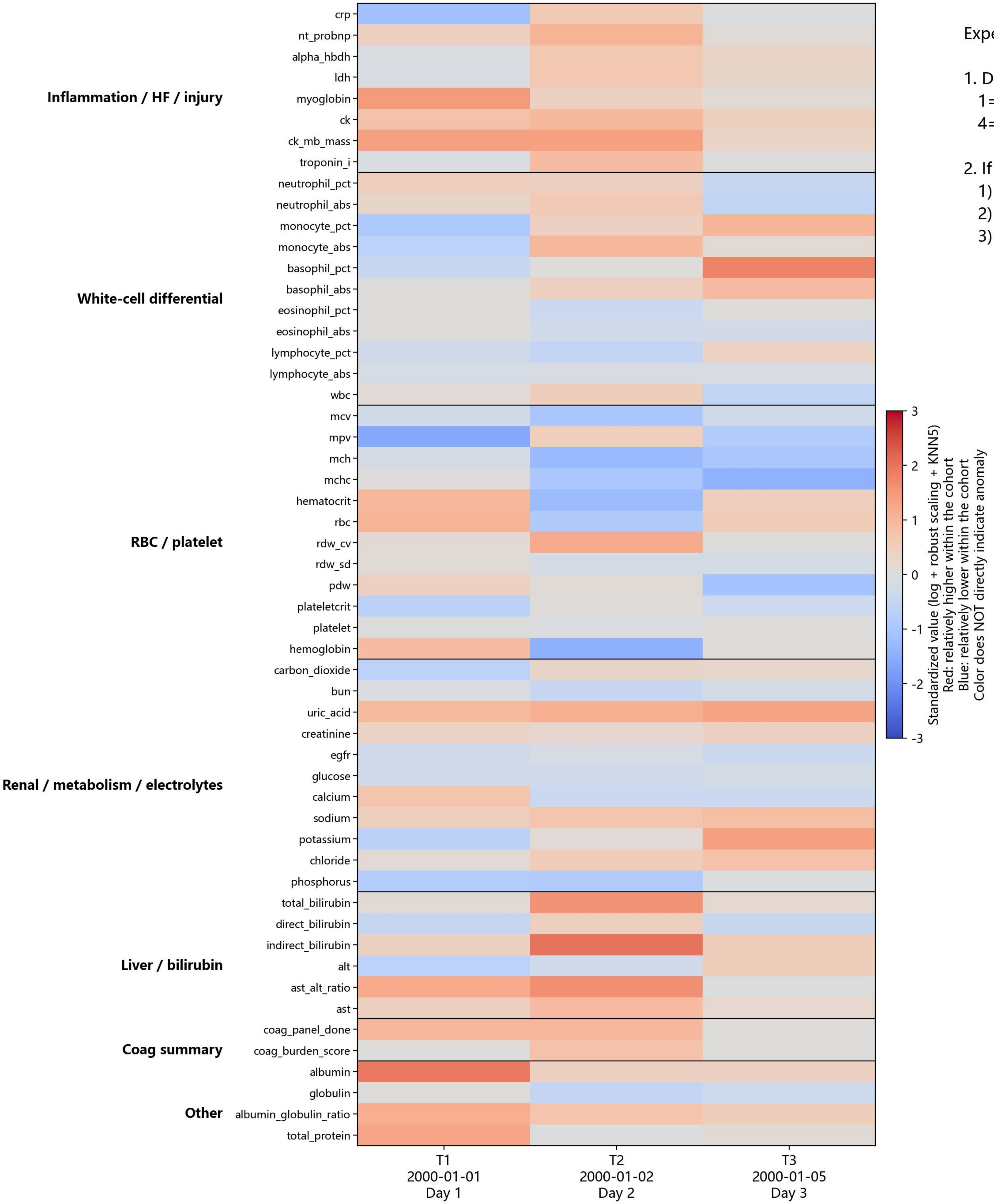

Expert review (blinded; no model score shown)

1. Degree of anomaly for this 3-point window (1-5):
   1=very typical; 2=relatively typical; 3=gray zone;
   4=relatively abnormal; 5=very abnormal

2. If scored 4-5, list the 3 most abnormal / noteworthy variables:
   1) ___________________________
   2) ___________________________
   3) ___________________________

Patient-window heatmap card for blinded expert review
ID: P130   Window: W01

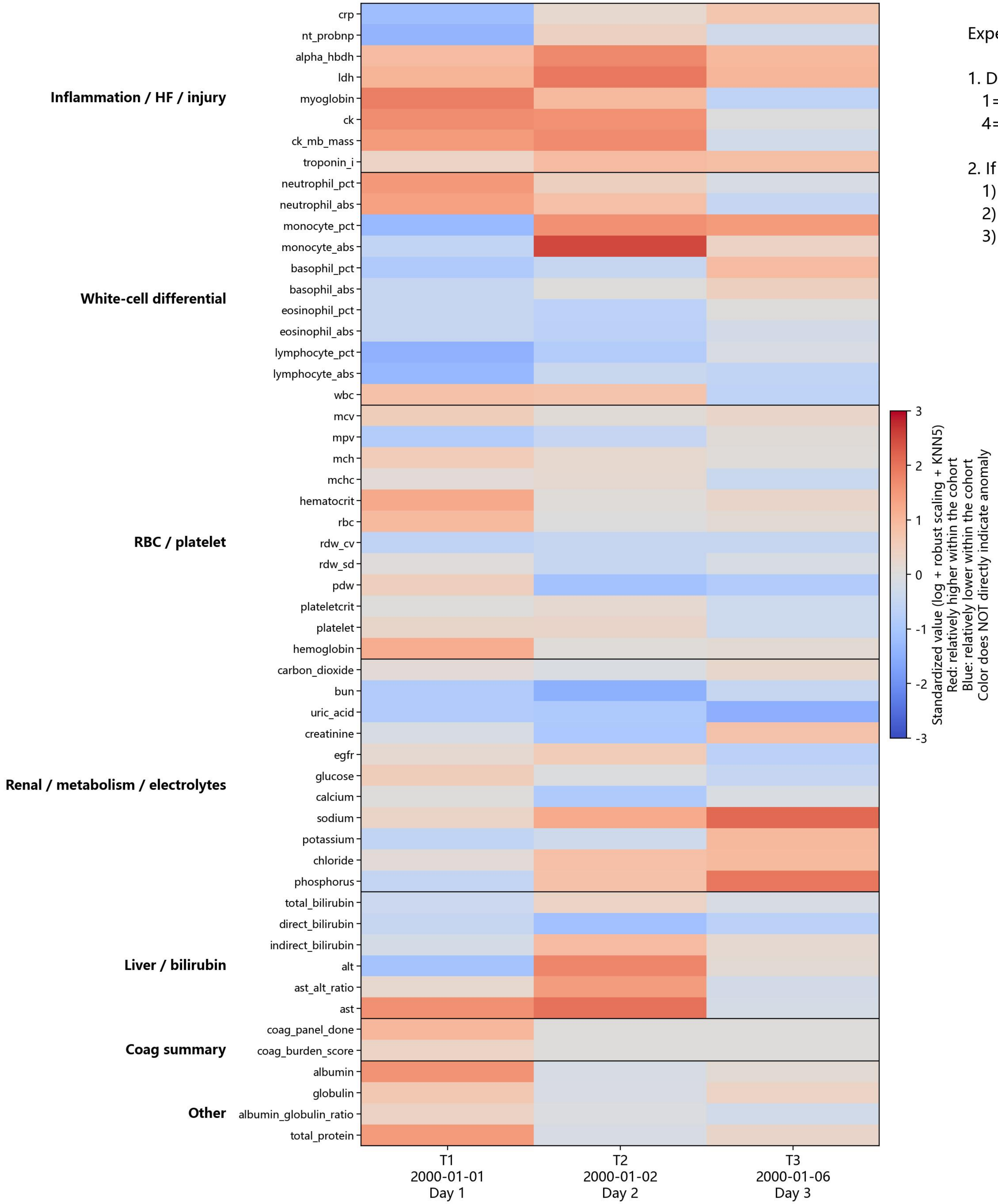

Expert review (blinded; no model score shown)

1. Degree of anomaly for this 3-point window (1-5):
   1=very typical; 2=relatively typical; 3=gray zone;
   4=relatively abnormal; 5=very abnormal

2. If scored 4-5, list the 3 most abnormal / noteworthy variables:
   1) ________________________
   2) ________________________
   3) ________________________

Patient-window heatmap card for blinded expert review
ID: P131   Window: W01

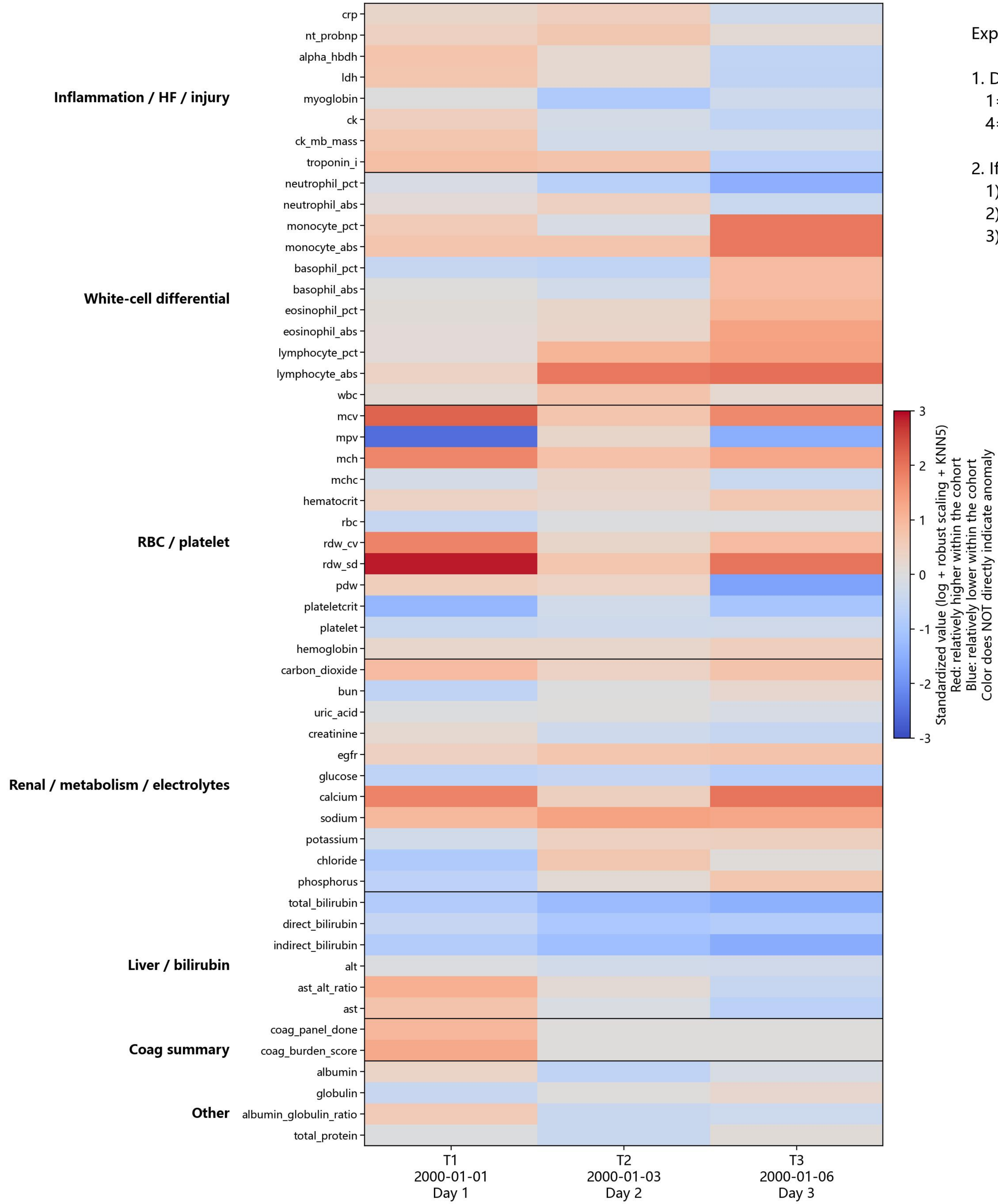

Expert review (blinded; no model score shown)

1. Degree of anomaly for this 3-point window (1-5):
   1=very typical; 2=relatively typical; 3=gray zone;
   4=relatively abnormal; 5=very abnormal

2. If scored 4-5, list the 3 most abnormal / noteworthy variables:
   1) ________________________
   2) ________________________
   3) ________________________

Patient-window heatmap card for blinded expert review
ID: P132   Window: W01

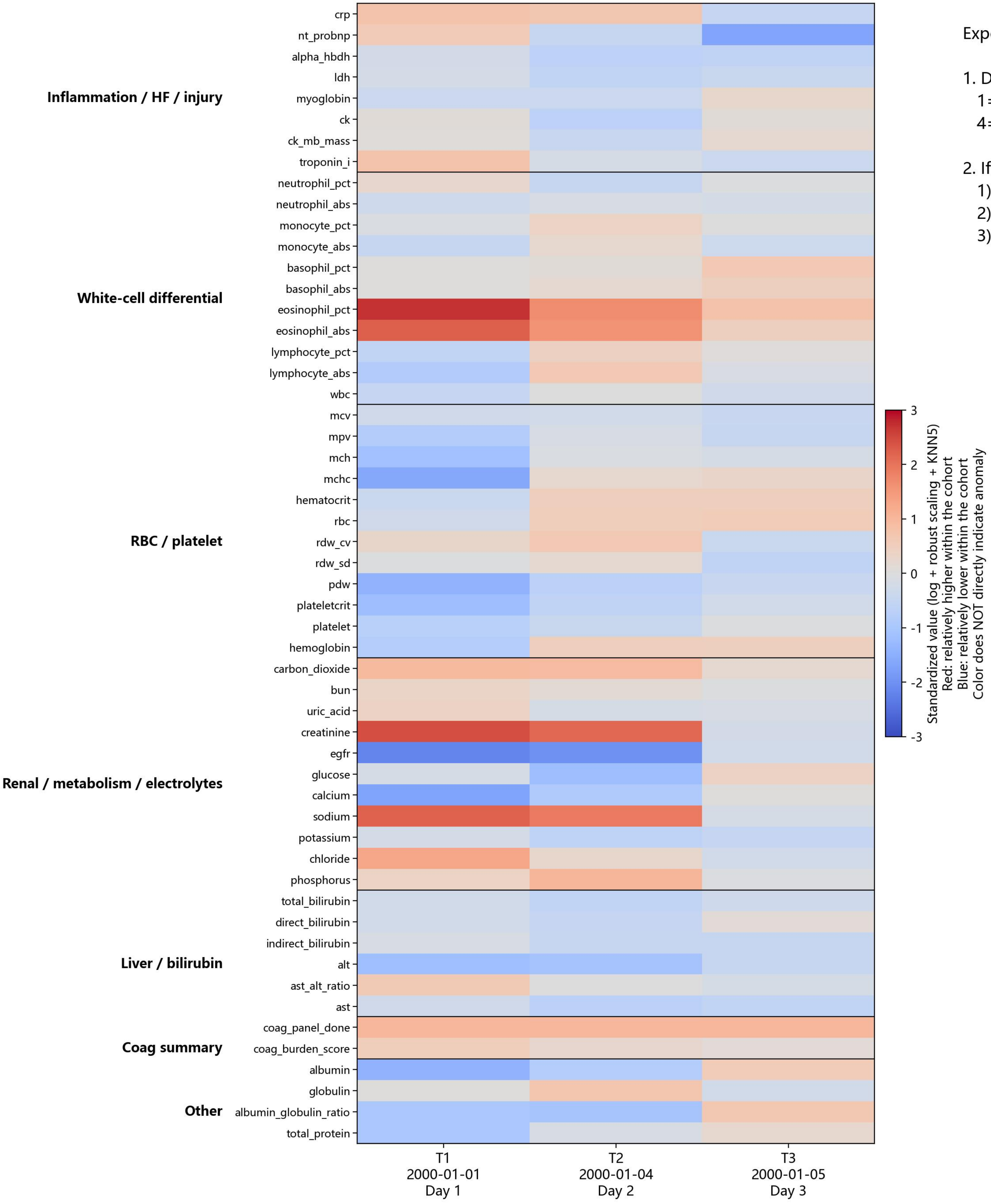

Expert review (blinded; no model score shown)

1. Degree of anomaly for this 3-point window (1-5):
   1=very typical; 2=relatively typical; 3=gray zone;
   4=relatively abnormal; 5=very abnormal

2. If scored 4-5, list the 3 most abnormal / noteworthy variables:
   1) ________________________
   2) ________________________
   3) ________________________

Patient-window heatmap card for blinded expert review
ID: P133   Window: W01

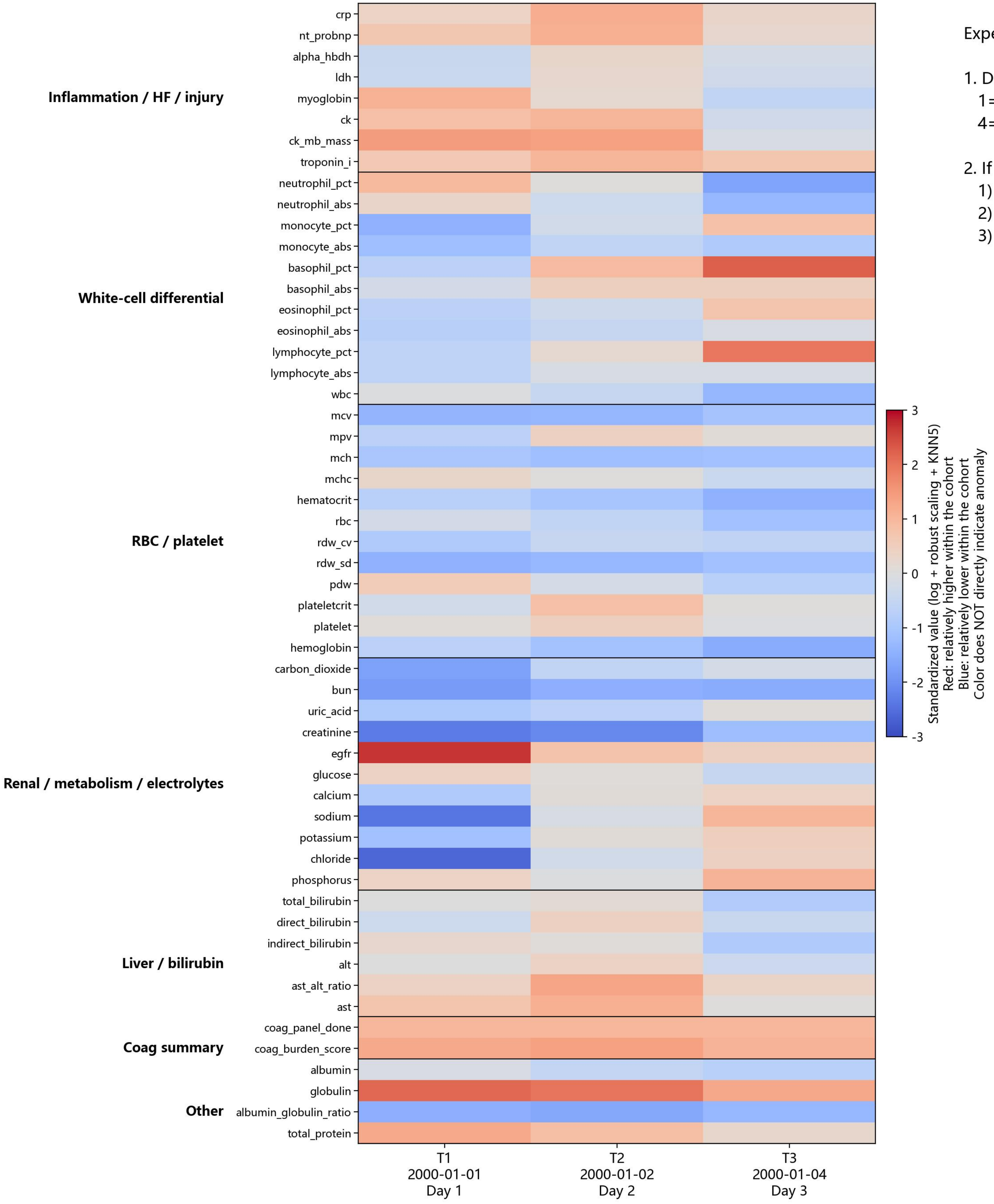

Expert review (blinded; no model score shown)

1. Degree of anomaly for this 3-point window (1-5):
   1=very typical; 2=relatively typical; 3=gray zone;
   4=relatively abnormal; 5=very abnormal

2. If scored 4-5, list the 3 most abnormal / noteworthy variables:
   1) ___________________________
   2) ___________________________
   3) ___________________________

Patient-window heatmap card for blinded expert review
ID: P134   Window: W01

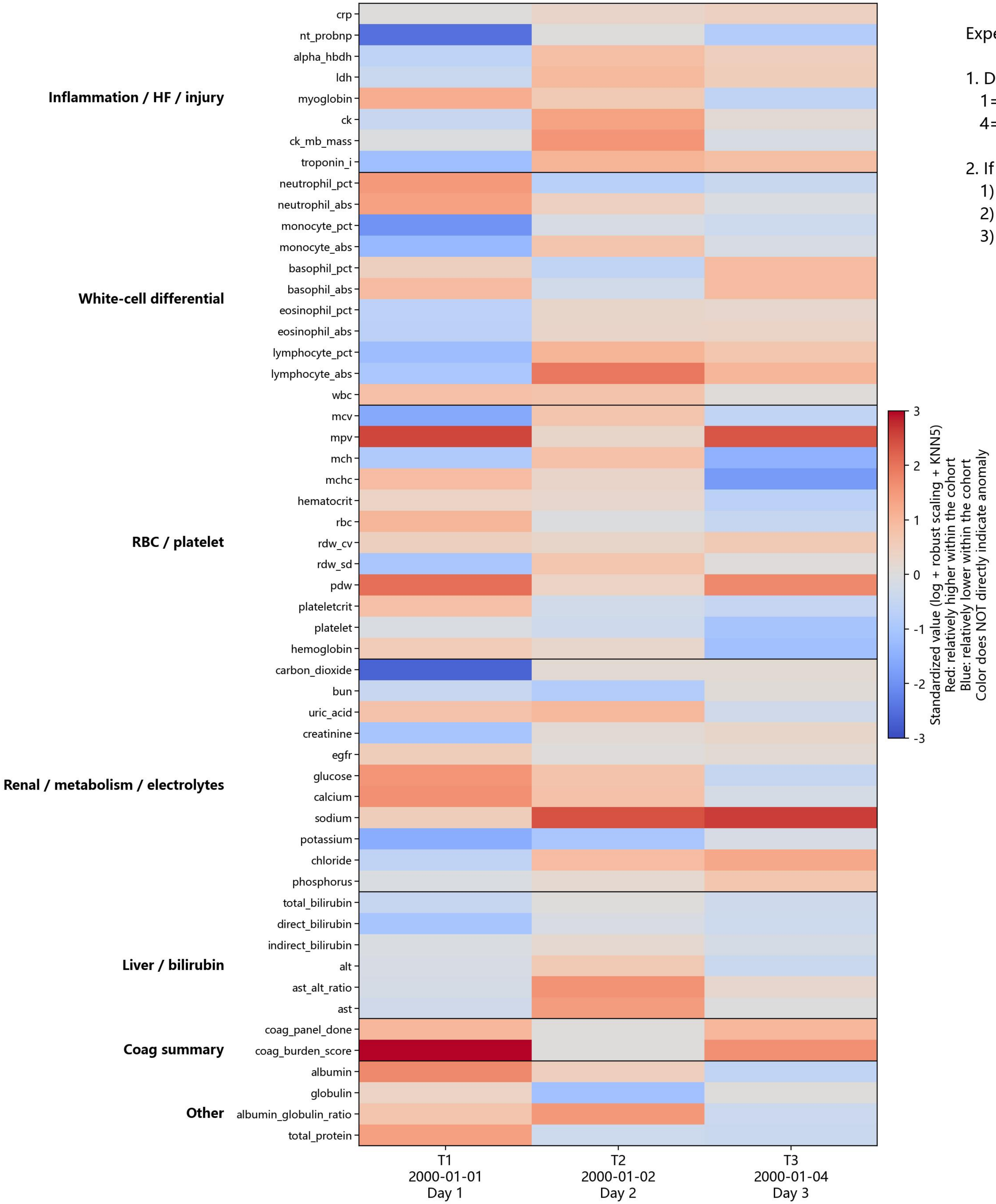

Expert review (blinded; no model score shown)

1. Degree of anomaly for this 3-point window (1-5):
   1=very typical; 2=relatively typical; 3=gray zone;
   4=relatively abnormal; 5=very abnormal

2. If scored 4-5, list the 3 most abnormal / noteworthy variables:
   1) ________________________
   2) ________________________
   3) ________________________

Patient-window heatmap card for blinded expert review
ID: P135   Window: W01

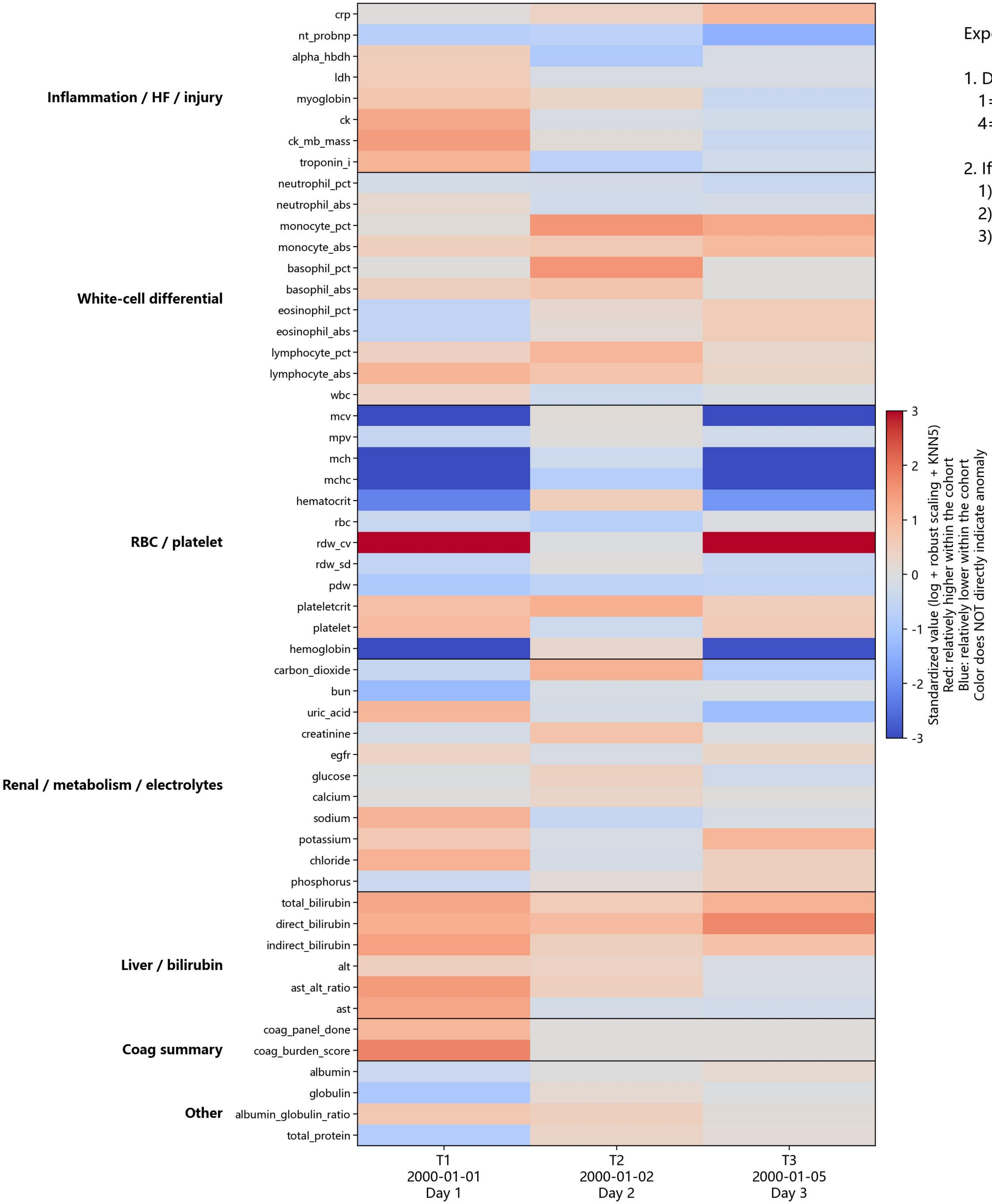

Expert review (blinded; no model score shown)

1. Degree of anomaly for this 3-point window (1-5):
   1=very typical; 2=relatively typical; 3=gray zone;
   4=relatively abnormal; 5=very abnormal

2. If scored 4-5, list the 3 most abnormal / noteworthy variables:
   1) ________________________
   2) ________________________
   3) ________________________

Patient-window heatmap card for blinded expert review
ID: P136   Window: W01

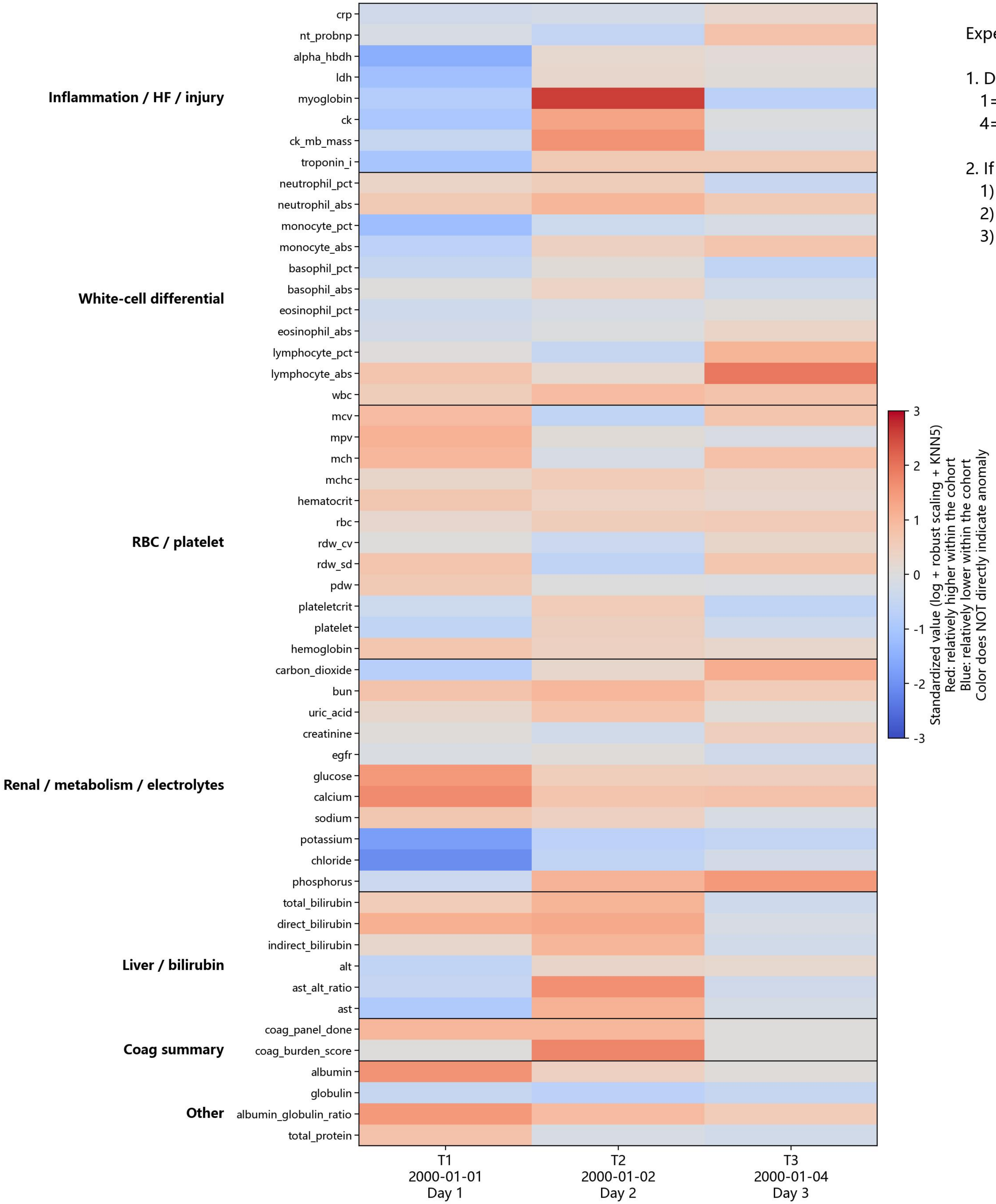

Expert review (blinded; no model score shown)

1. Degree of anomaly for this 3-point window (1-5):
   1=very typical; 2=relatively typical; 3=gray zone;
   4=relatively abnormal; 5=very abnormal

2. If scored 4-5, list the 3 most abnormal / noteworthy variables:
   1) ________________________
   2) ________________________
   3) ________________________

Patient-window heatmap card for blinded expert review
ID: P137   Window: W01

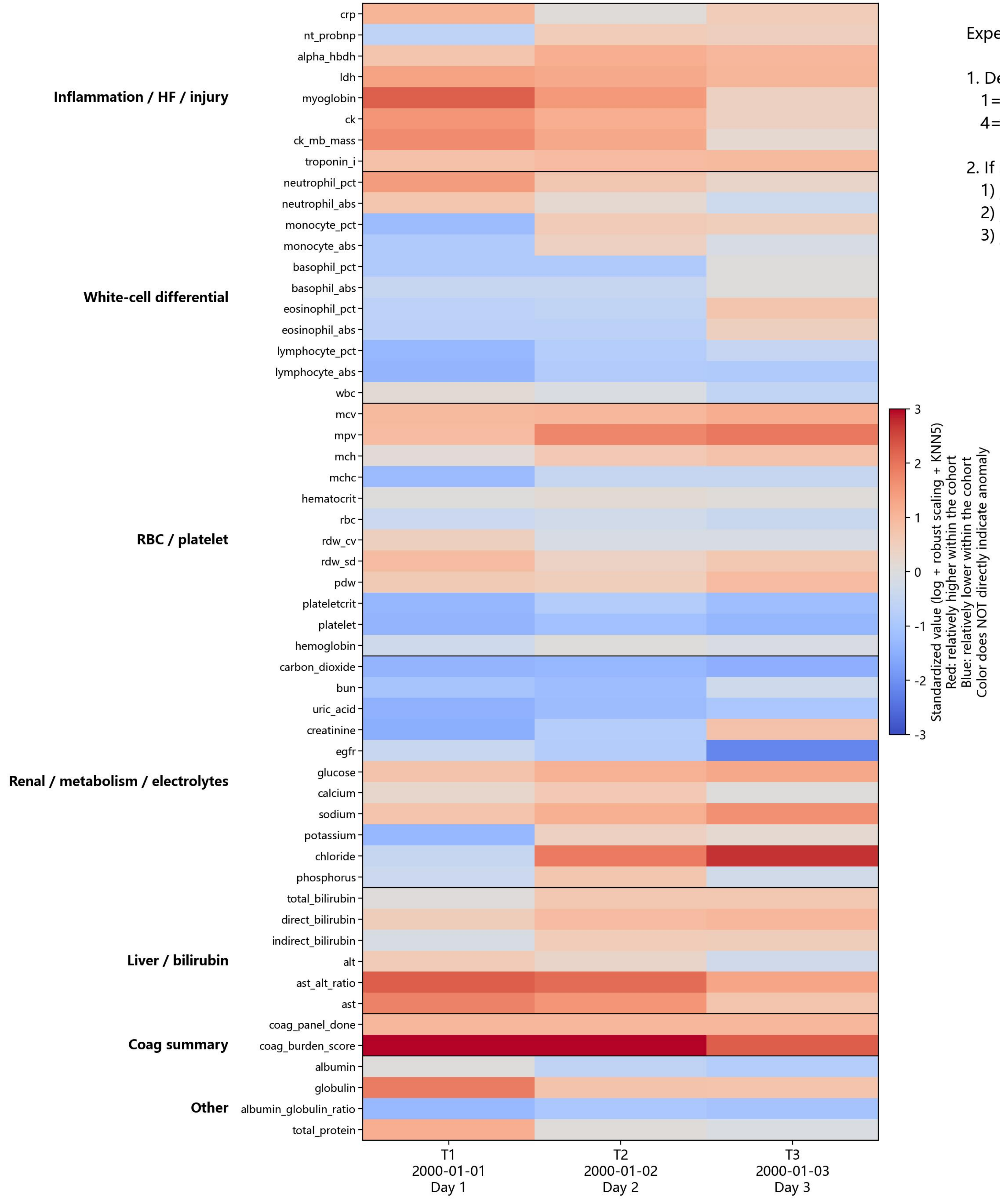

Expert review (blinded; no model score shown)

1. Degree of anomaly for this 3-point window (1-5):
   1=very typical; 2=relatively typical; 3=gray zone;
   4=relatively abnormal; 5=very abnormal

2. If scored 4-5, list the 3 most abnormal / noteworthy variables:
   1) ________________________
   2) ________________________
   3) ________________________

Patient-window heatmap card for blinded expert review
ID: P138   Window: W01

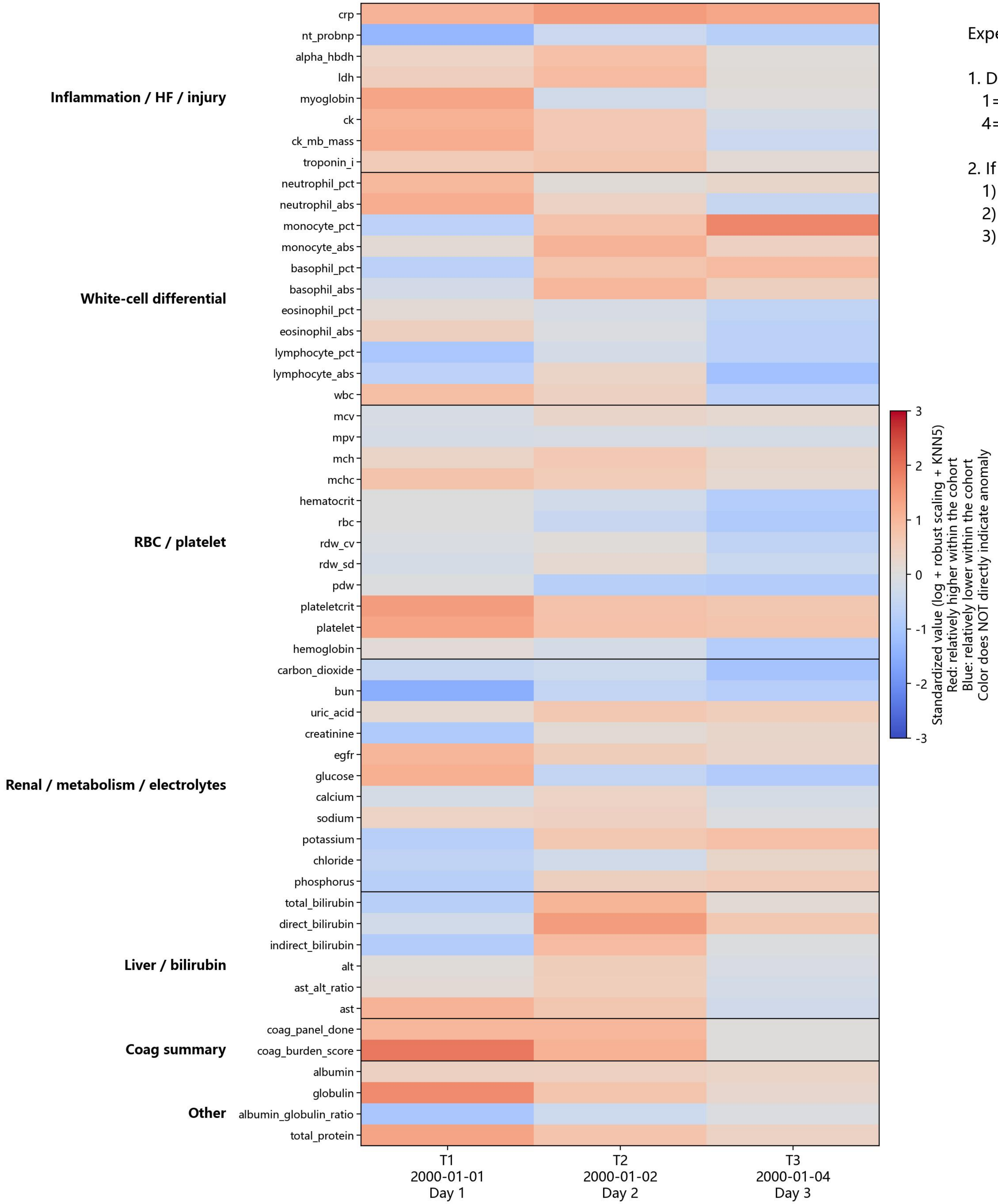

Expert review (blinded; no model score shown)

1. Degree of anomaly for this 3-point window (1-5):
   1=very typical; 2=relatively typical; 3=gray zone;
   4=relatively abnormal; 5=very abnormal

2. If scored 4-5, list the 3 most abnormal / noteworthy variables:
   1) ________________________
   2) ________________________
   3) ________________________

Patient-window heatmap card for blinded expert review
ID: P139   Window: W01

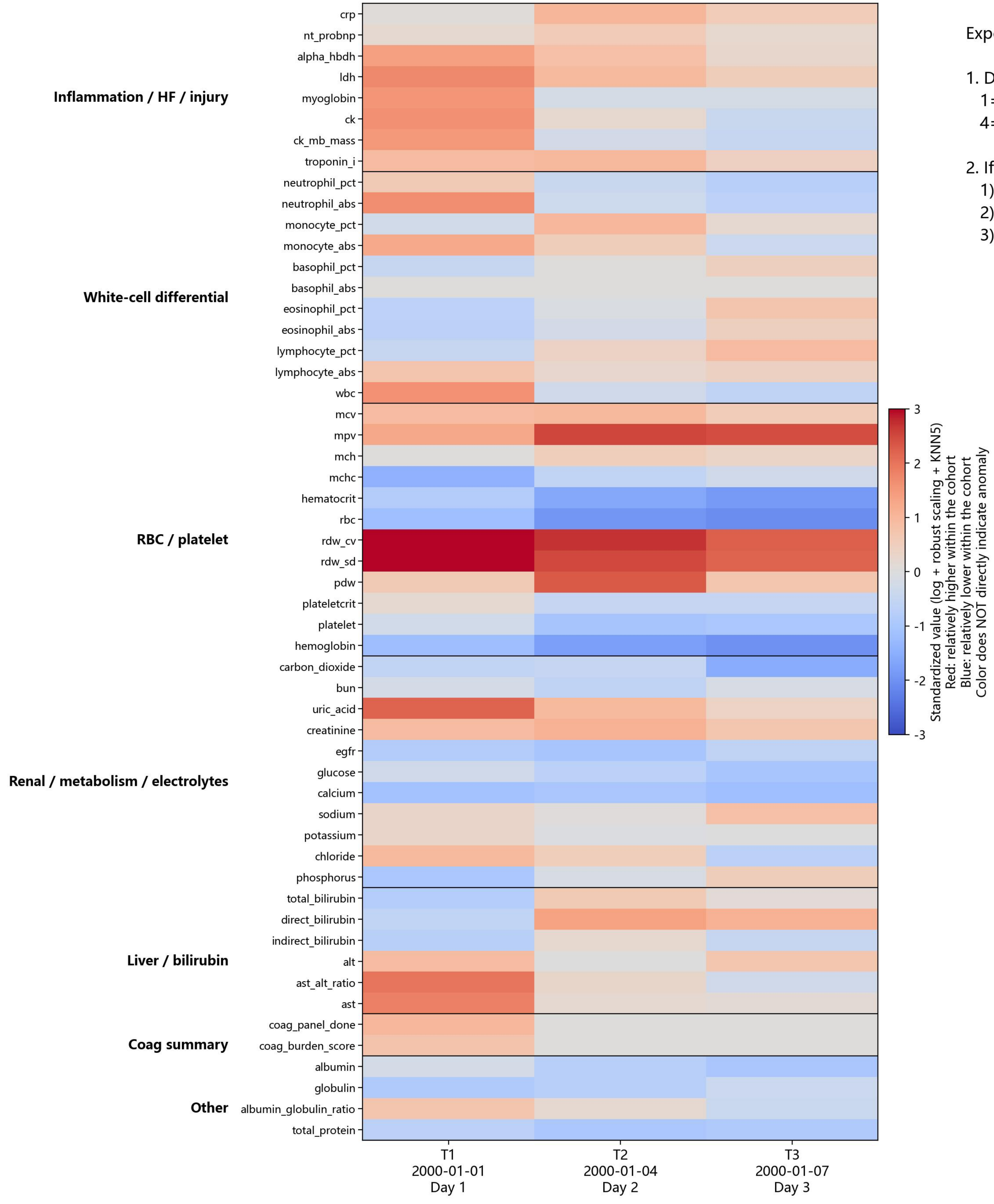

Expert review (blinded; no model score shown)

1. Degree of anomaly for this 3-point window (1-5):
   1=very typical; 2=relatively typical; 3=gray zone;
   4=relatively abnormal; 5=very abnormal

2. If scored 4-5, list the 3 most abnormal / noteworthy variables:
   1) ________________________
   2) ________________________
   3) ________________________

Patient-window heatmap card for blinded expert review
ID: P140   Window: W01

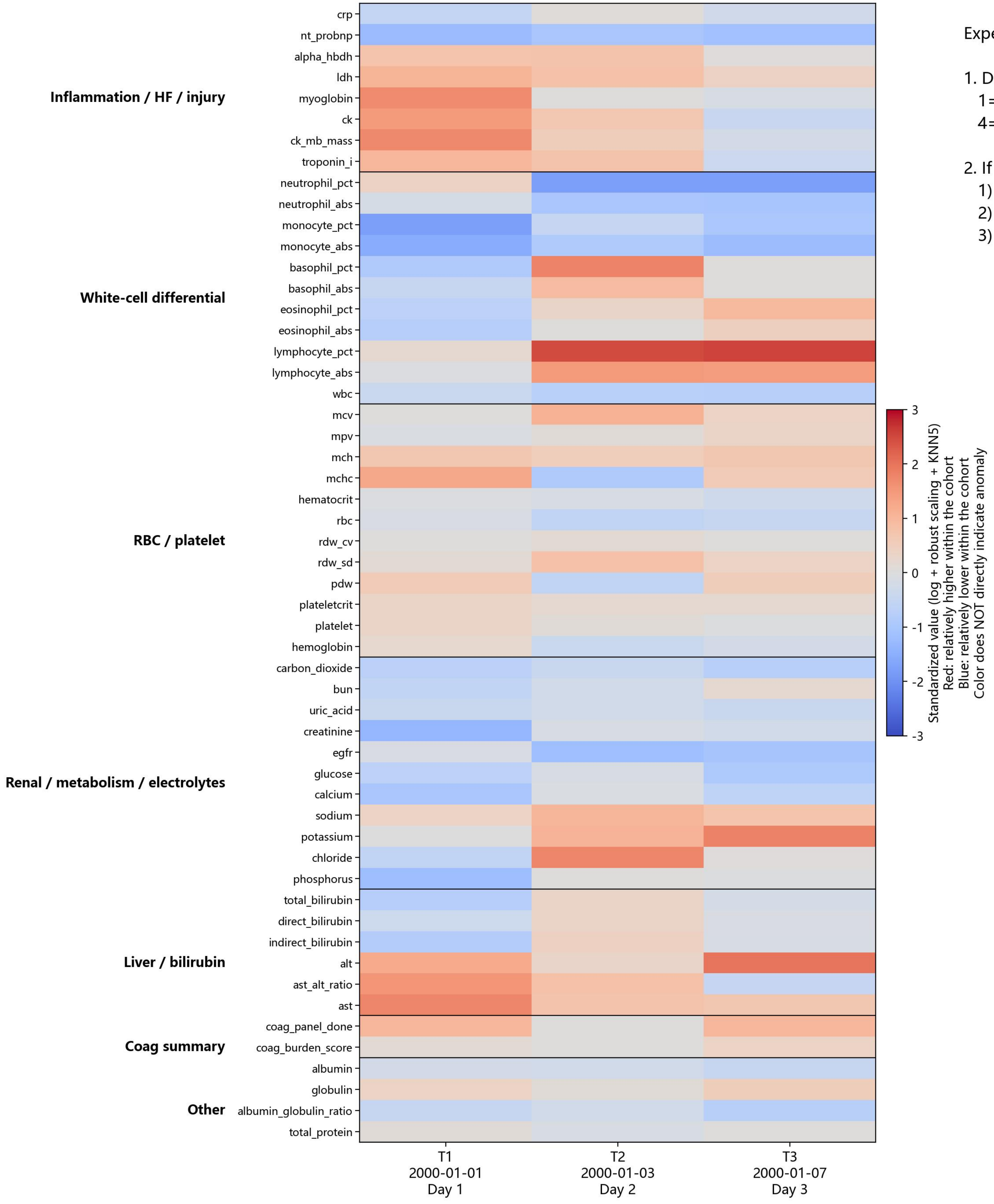

Expert review (blinded; no model score shown)

1. Degree of anomaly for this 3-point window (1-5):
   1=very typical; 2=relatively typical; 3=gray zone;
   4=relatively abnormal; 5=very abnormal

2. If scored 4-5, list the 3 most abnormal / noteworthy variables:
   1) __________________________
   2) __________________________
   3) __________________________

Patient-window heatmap card for blinded expert review
ID: P141   Window: W01

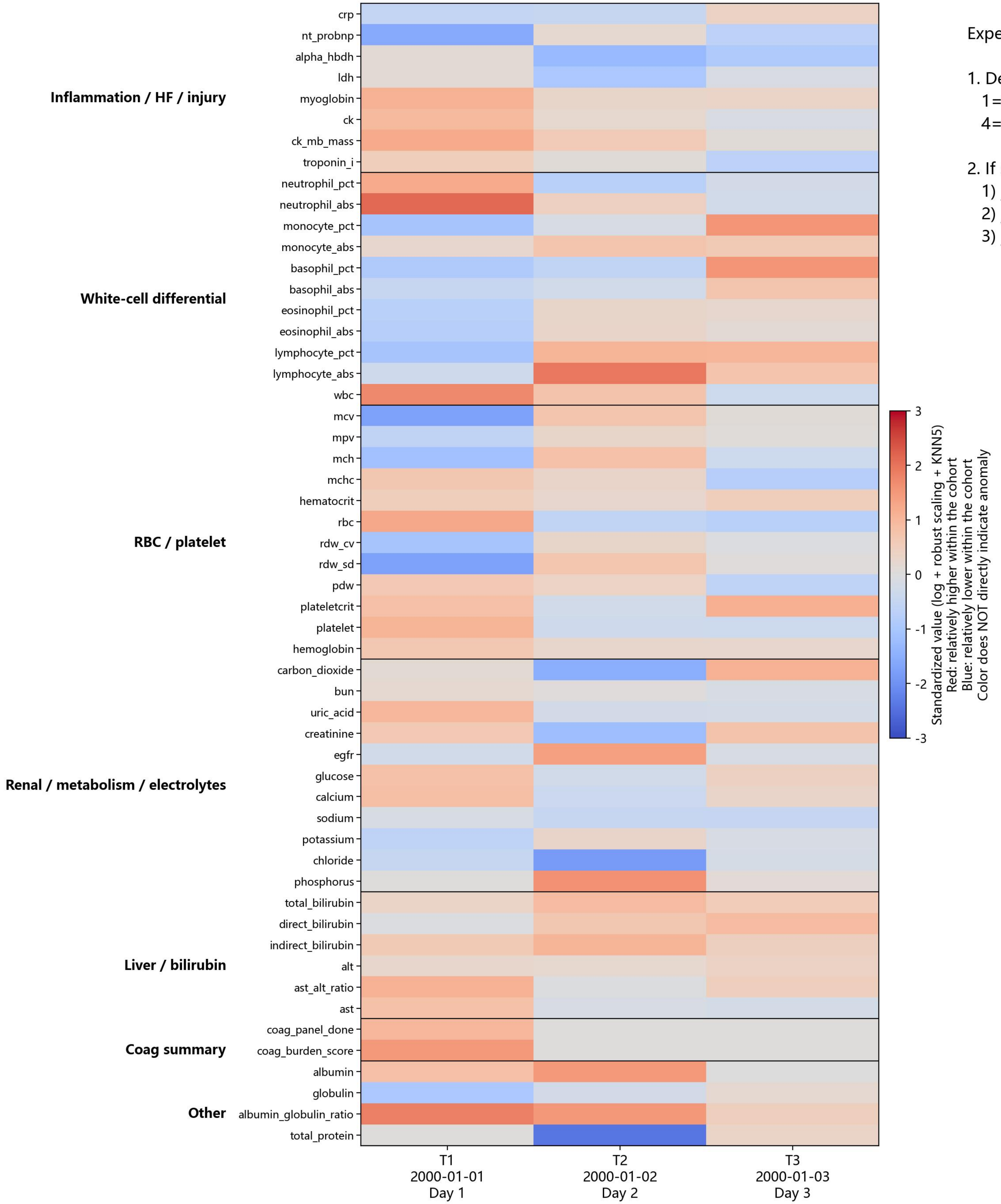

Expert review (blinded; no model score shown)

1. Degree of anomaly for this 3-point window (1-5):
   1=very typical; 2=relatively typical; 3=gray zone;
   4=relatively abnormal; 5=very abnormal

2. If scored 4-5, list the 3 most abnormal / noteworthy variables:
   1) ____________________________
   2) ____________________________
   3) ____________________________

Patient-window heatmap card for blinded expert review
ID: P142 Window: W01

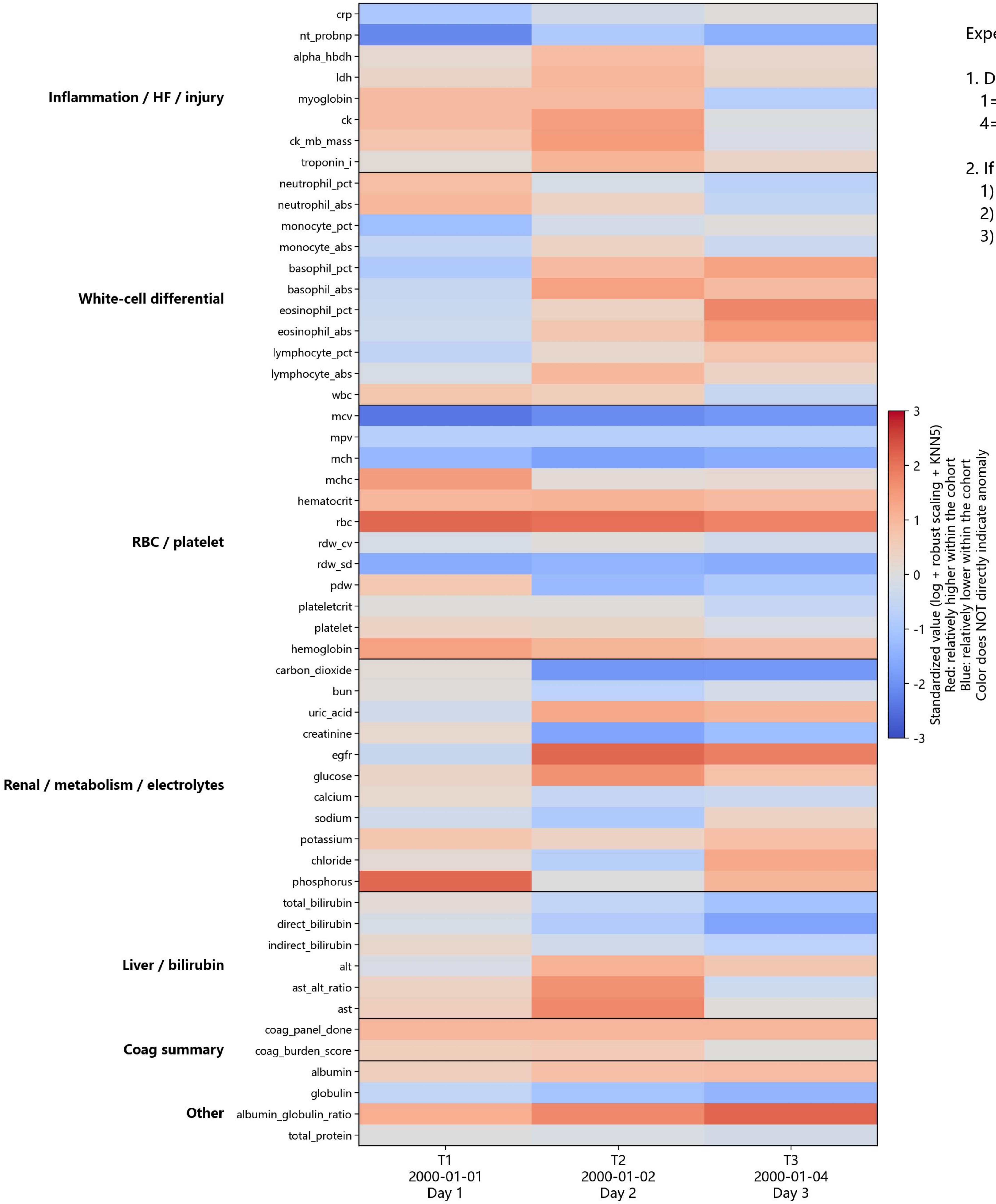

Expert review (blinded; no model score shown)

1. Degree of anomaly for this 3-point window (1-5):
   1=very typical; 2=relatively typical; 3=gray zone;
   4=relatively abnormal; 5=very abnormal

2. If scored 4-5, list the 3 most abnormal / noteworthy variables:
   1) ________________________
   2) ________________________
   3) ________________________

Patient-window heatmap card for blinded expert review
ID: P143   Window: W01

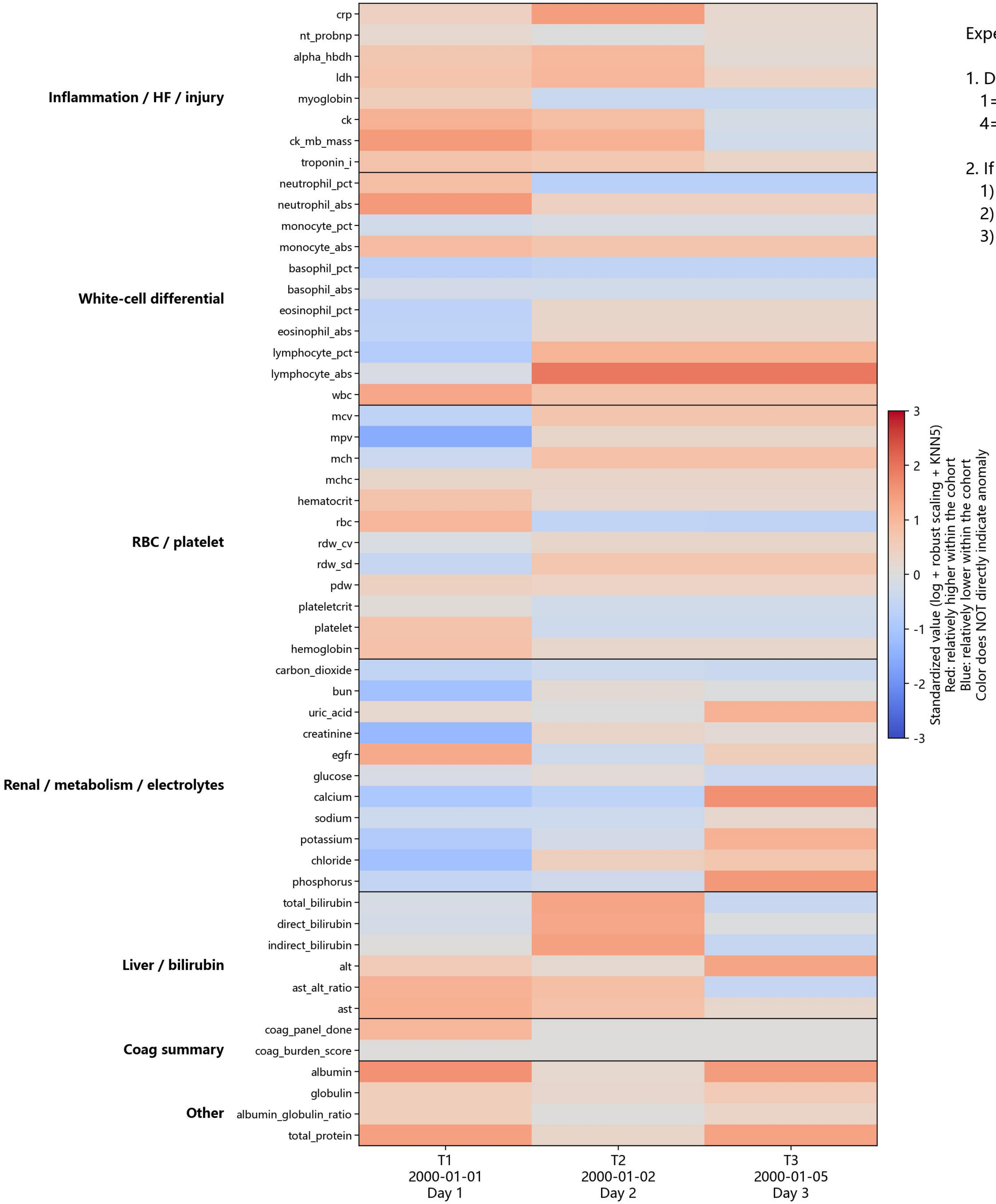

Expert review (blinded; no model score shown)

1. Degree of anomaly for this 3-point window (1-5):
   1=very typical; 2=relatively typical; 3=gray zone;
   4=relatively abnormal; 5=very abnormal

2. If scored 4-5, list the 3 most abnormal / noteworthy variables:
   1) ______________________
   2) ______________________
   3) ______________________

Patient-window heatmap card for blinded expert review
ID: P144   Window: W01

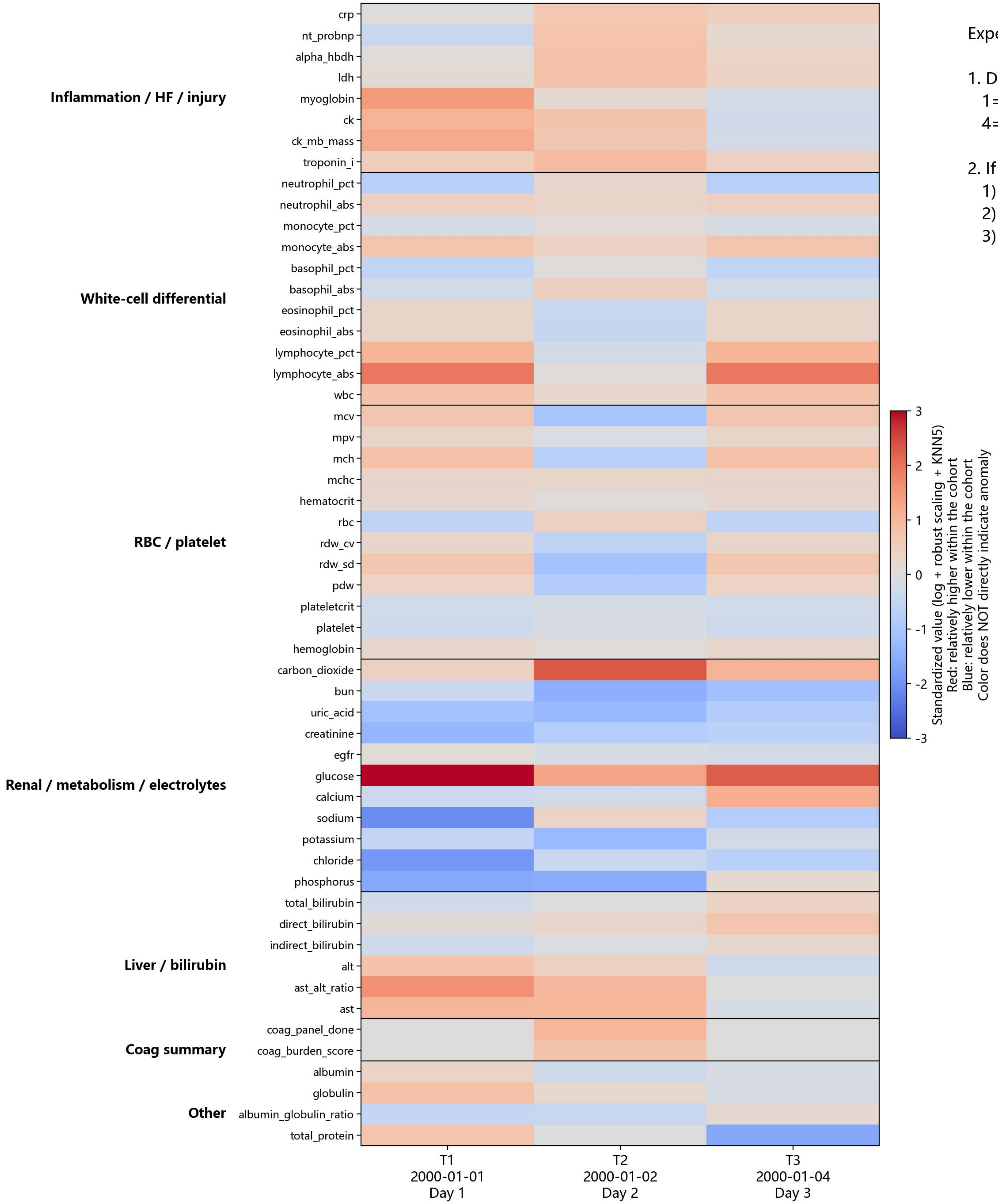

Expert review (blinded; no model score shown)

1. Degree of anomaly for this 3-point window (1-5):
   1=very typical; 2=relatively typical; 3=gray zone;
   4=relatively abnormal; 5=very abnormal

2. If scored 4-5, list the 3 most abnormal / noteworthy variables:
   1) ____________________________
   2) ____________________________
   3) ____________________________

Patient-window heatmap card for blinded expert review
ID: P145 Window: W01

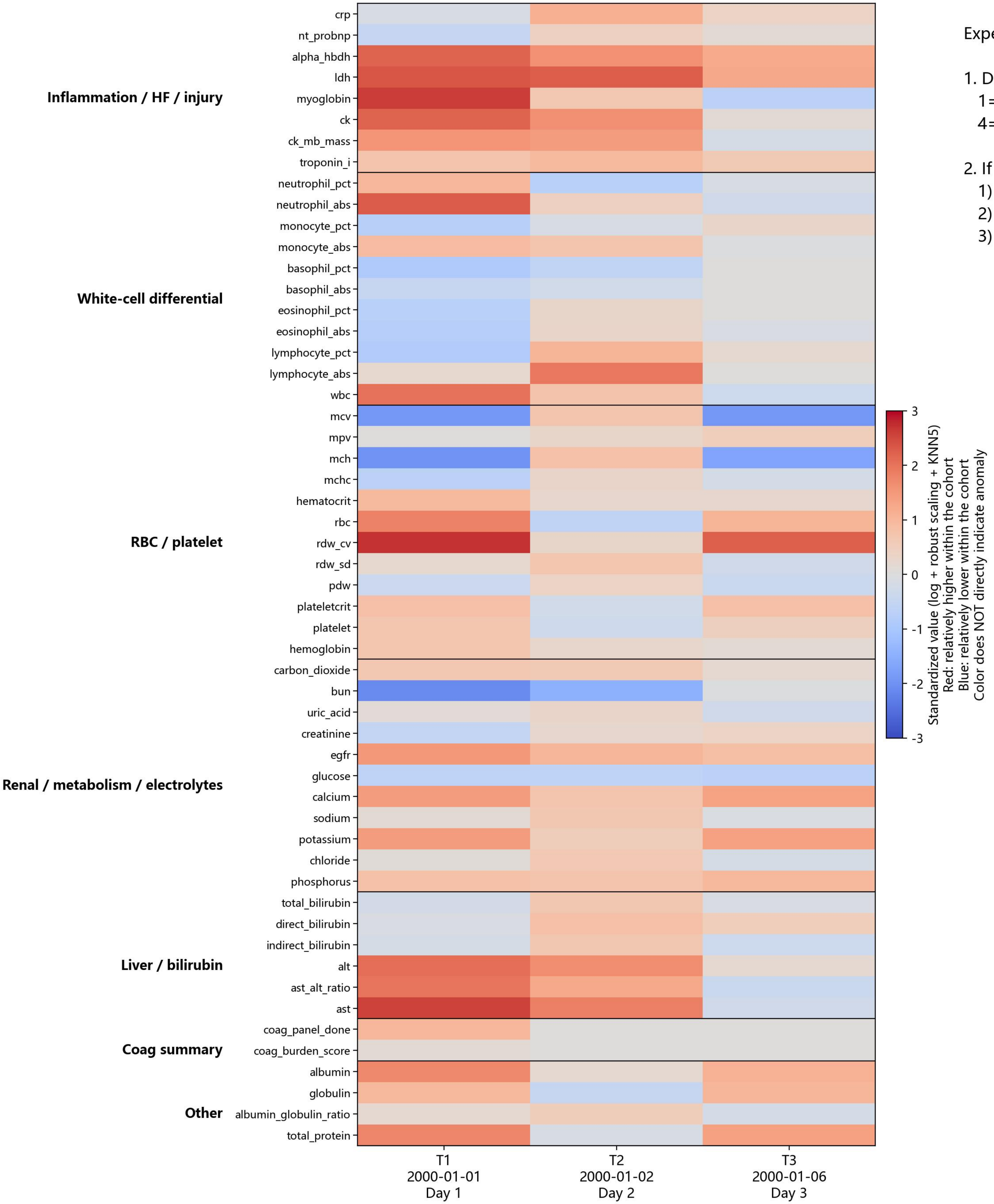

Expert review (blinded; no model score shown)

1. Degree of anomaly for this 3-point window (1-5):
   1=very typical; 2=relatively typical; 3=gray zone;
   4=relatively abnormal; 5=very abnormal

2. If scored 4-5, list the 3 most abnormal / noteworthy variables:
   1) ________________________
   2) ________________________
   3) ________________________

Patient-window heatmap card for blinded expert review
ID: P146   Window: W01

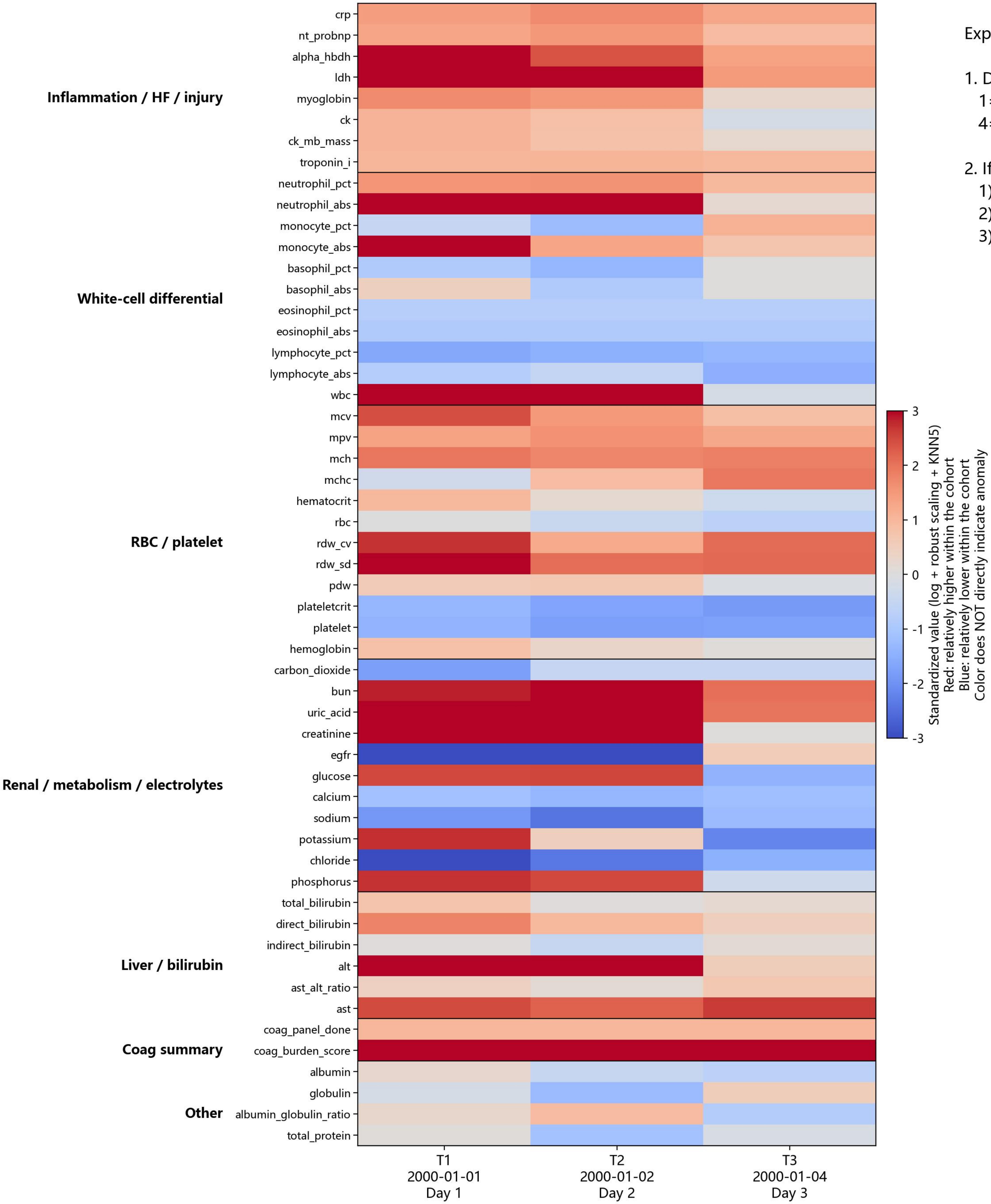

Expert review (blinded; no model score shown)

1. Degree of anomaly for this 3-point window (1-5):
   1=very typical; 2=relatively typical; 3=gray zone;
   4=relatively abnormal; 5=very abnormal

2. If scored 4-5, list the 3 most abnormal / noteworthy variables:
   1) ________________________
   2) ________________________
   3) ________________________

Patient-window heatmap card for blinded expert review
ID: P147   Window: W01

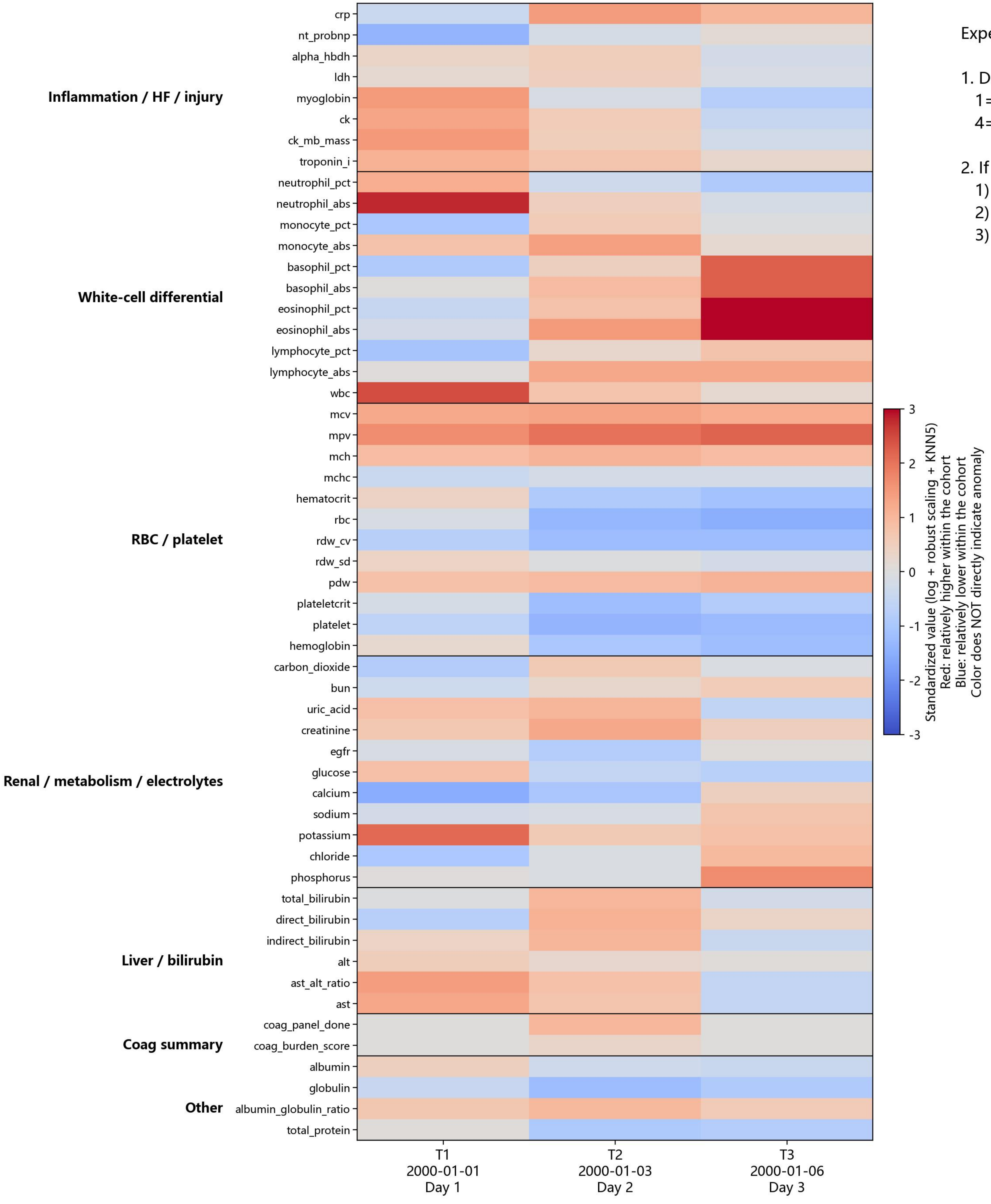

Expert review (blinded; no model score shown)

1. Degree of anomaly for this 3-point window (1-5):
   1=very typical; 2=relatively typical; 3=gray zone;
   4=relatively abnormal; 5=very abnormal

2. If scored 4-5, list the 3 most abnormal / noteworthy variables:
   1) ________________________
   2) ________________________
   3) ________________________

Patient-window heatmap card for blinded expert review
ID: P148   Window: W01

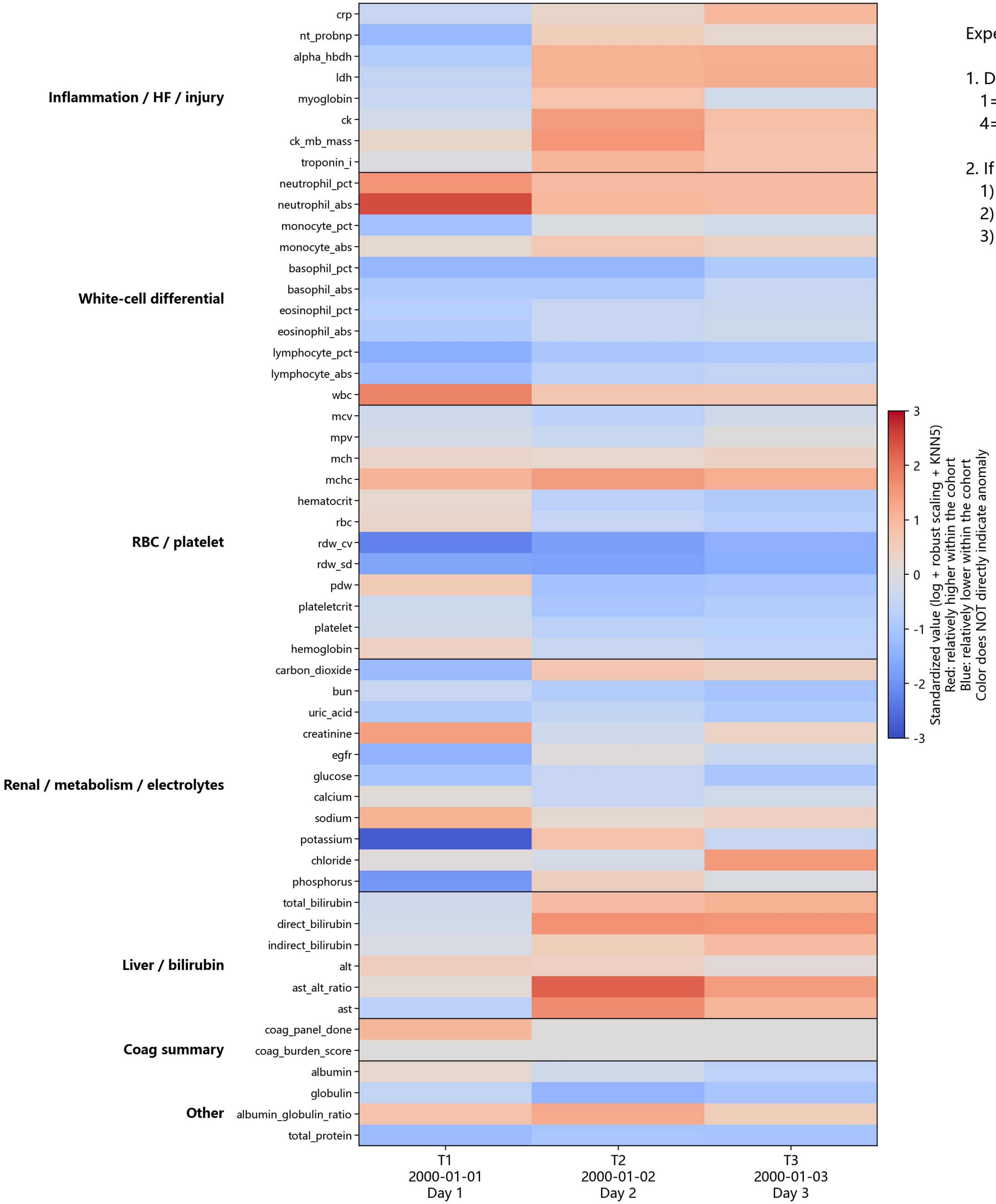

Expert review (blinded; no model score shown)

1. Degree of anomaly for this 3-point window (1-5):
   1=very typical; 2=relatively typical; 3=gray zone;
   4=relatively abnormal; 5=very abnormal

2. If scored 4-5, list the 3 most abnormal / noteworthy variables:
   1) ________________________
   2) ________________________
   3) ________________________

Patient-window heatmap card for blinded expert review
ID: P149   Window: W01

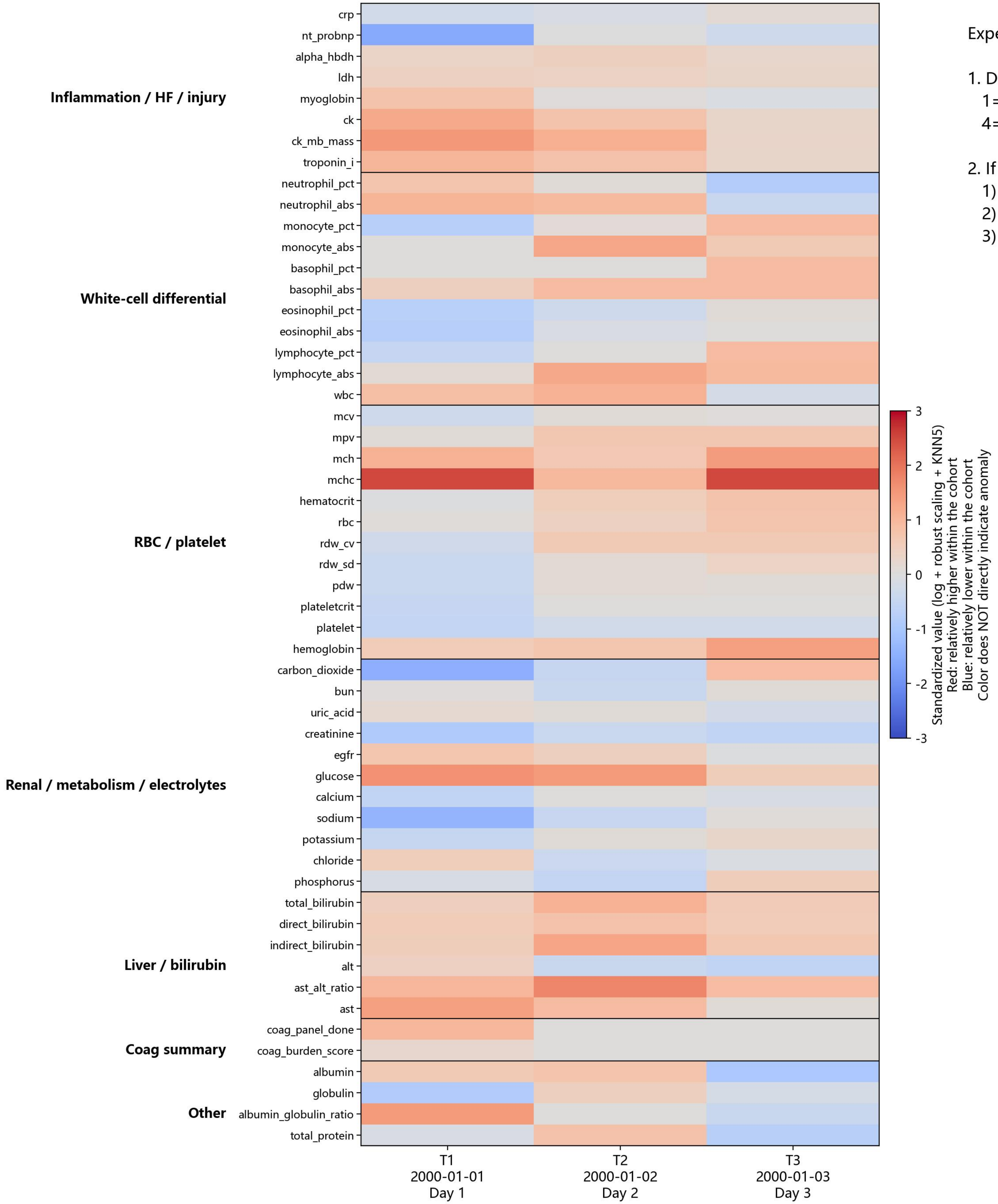

Expert review (blinded; no model score shown)

1. Degree of anomaly for this 3-point window (1-5):
   1=very typical; 2=relatively typical; 3=gray zone;
   4=relatively abnormal; 5=very abnormal

2. If scored 4-5, list the 3 most abnormal / noteworthy variables:
   1) ________________________
   2) ________________________
   3) ________________________

Patient-window heatmap card for blinded expert review
ID: P150   Window: W01

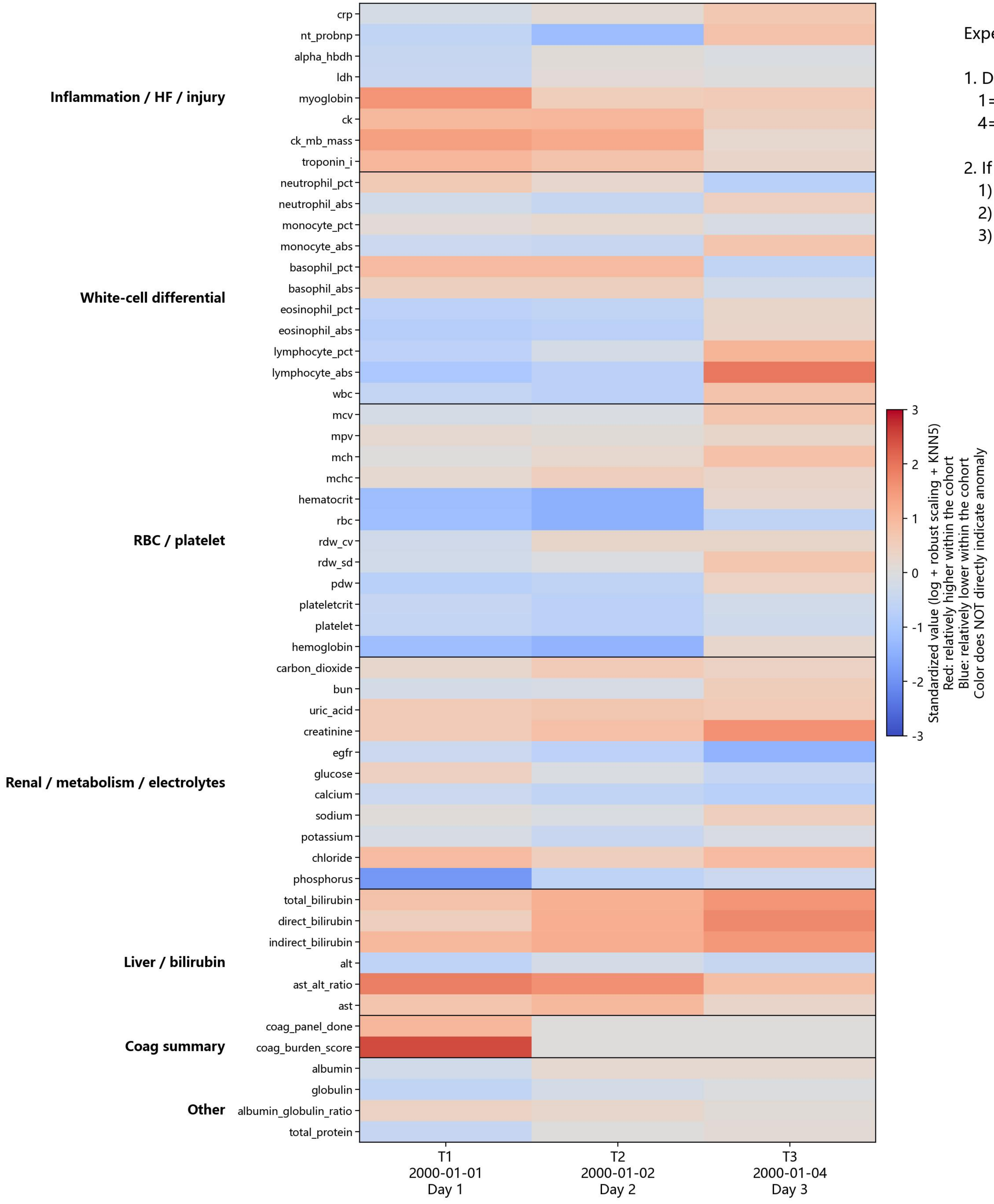

Expert review (blinded; no model score shown)

1. Degree of anomaly for this 3-point window (1-5):
   1=very typical; 2=relatively typical; 3=gray zone;
   4=relatively abnormal; 5=very abnormal

2. If scored 4-5, list the 3 most abnormal / noteworthy variables:
   1) ________________________
   2) ________________________
   3) ________________________

Patient-window heatmap card for blinded expert review
ID: P151 Window: W01

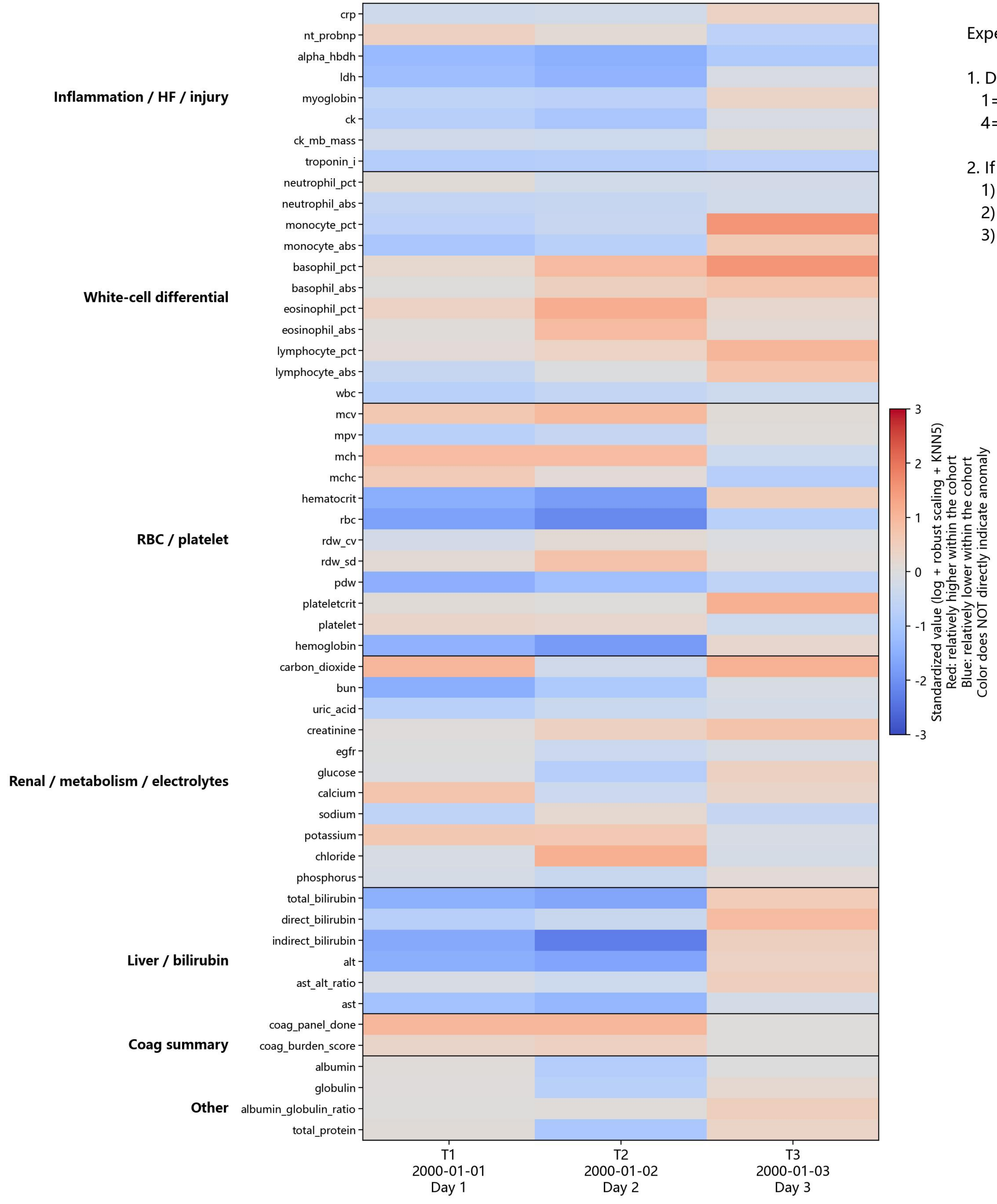

Expert review (blinded; no model score shown)

1. Degree of anomaly for this 3-point window (1-5):
1=very typical; 2=relatively typical; 3=gray zone;
4=relatively abnormal; 5=very abnormal

2. If scored 4-5, list the 3 most abnormal / noteworthy variables:
1) ____________________
2) ____________________
3) ____________________

Patient-window heatmap card for blinded expert review
ID: P152 Window: W01

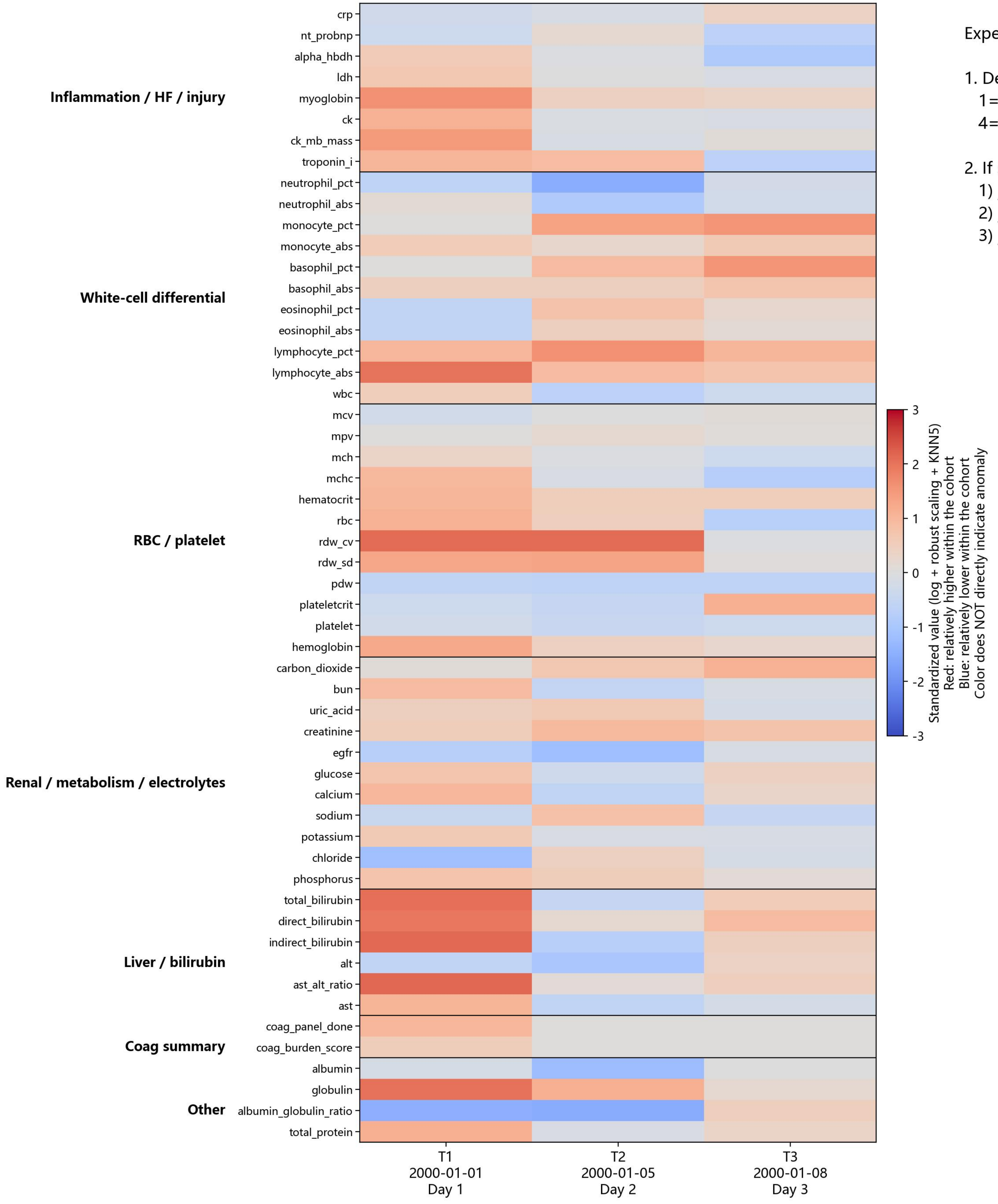

Expert review (blinded; no model score shown)

1. Degree of anomaly for this 3-point window (1-5):
1=very typical; 2=relatively typical; 3=gray zone;
4=relatively abnormal; 5=very abnormal

2. If scored 4-5, list the 3 most abnormal / noteworthy variables:
1) ________________________
2) ________________________
3) ________________________

Patient-window heatmap card for blinded expert review
ID: P153   Window: W01

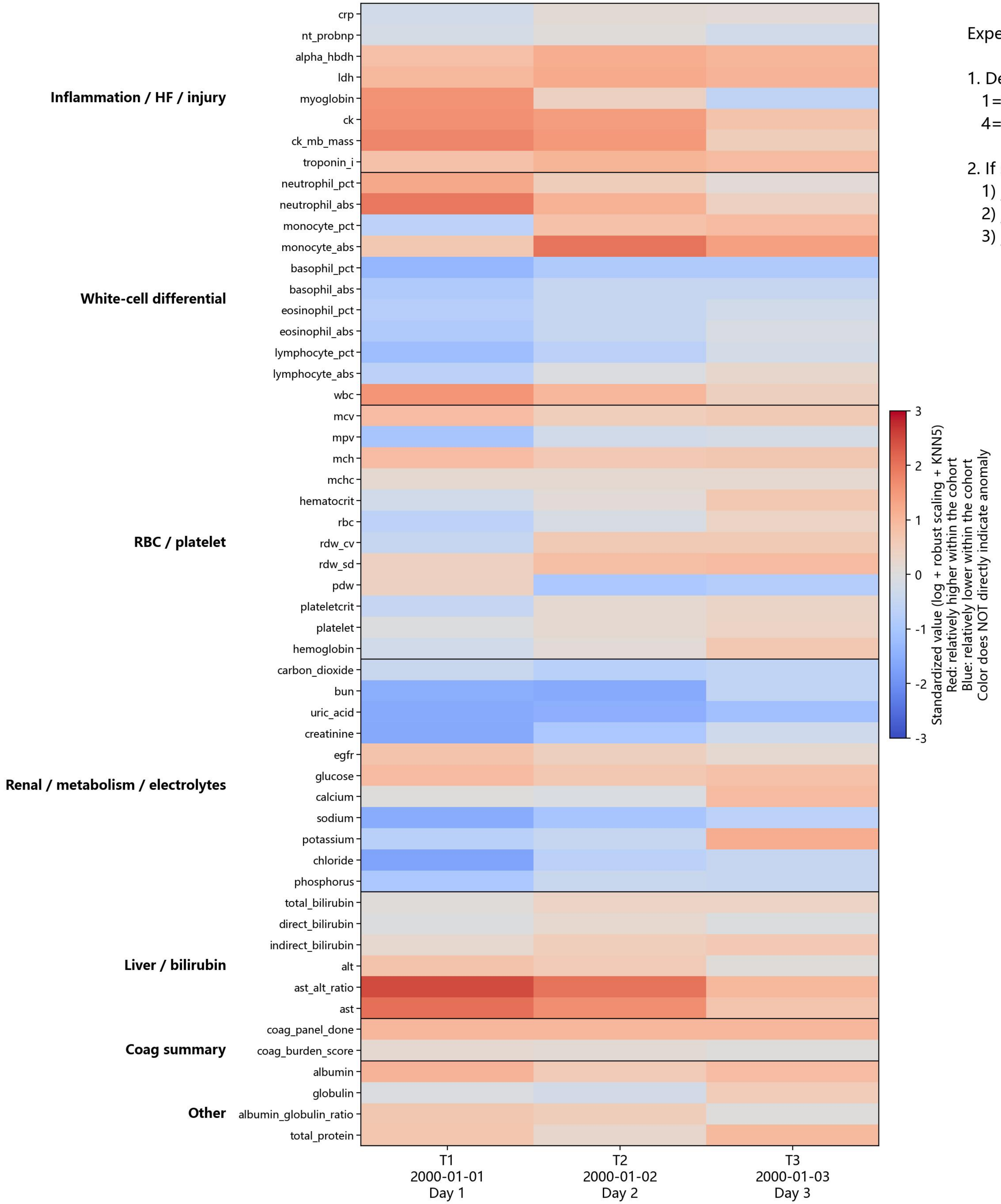

Expert review (blinded; no model score shown)

1. Degree of anomaly for this 3-point window (1-5):
   1=very typical; 2=relatively typical; 3=gray zone;
   4=relatively abnormal; 5=very abnormal

2. If scored 4-5, list the 3 most abnormal / noteworthy variables:
   1) ________________________
   2) ________________________
   3) ________________________

Patient-window heatmap card for blinded expert review
ID: P154   Window: W01

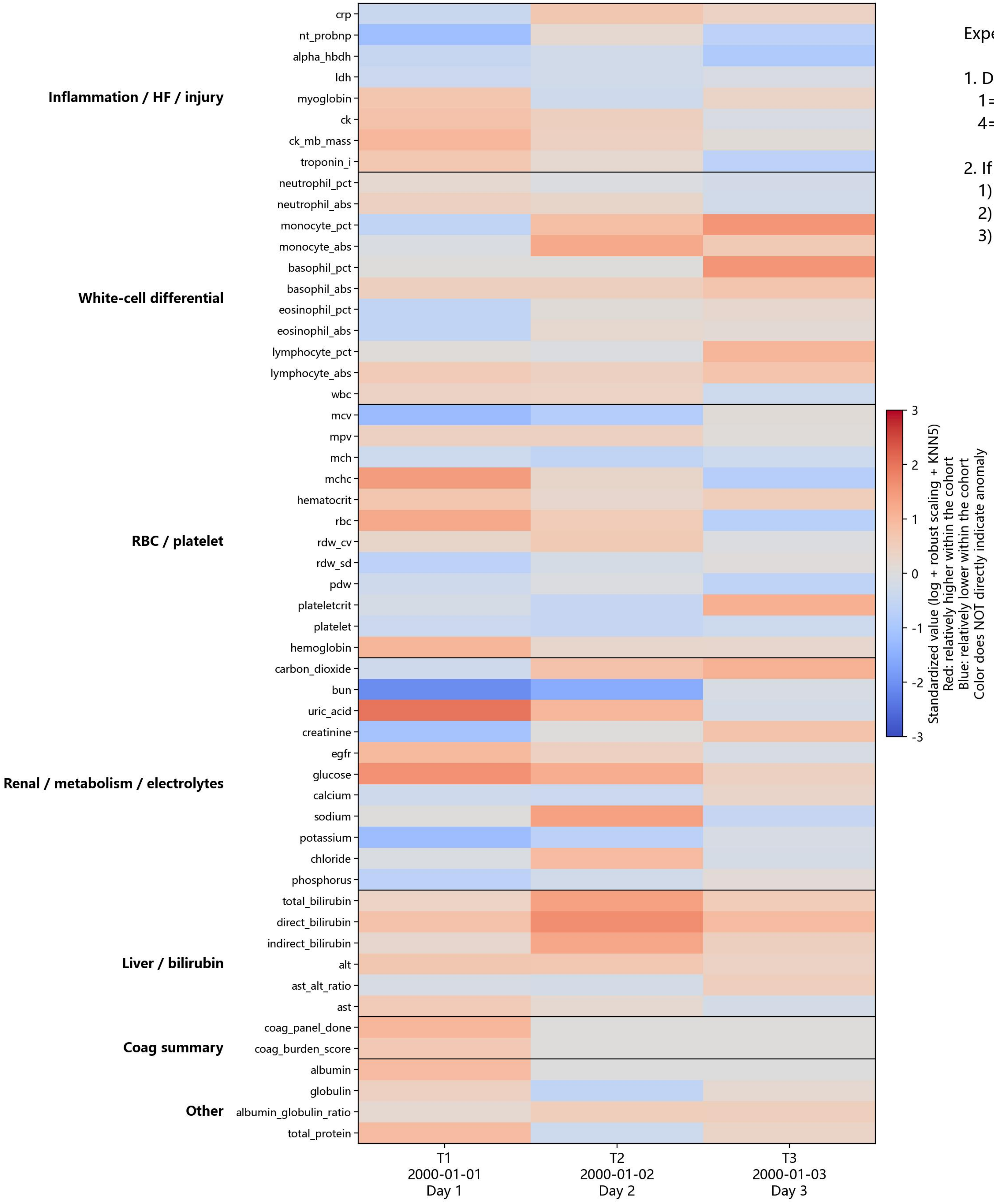

Expert review (blinded; no model score shown)

1. Degree of anomaly for this 3-point window (1-5):
   1=very typical; 2=relatively typical; 3=gray zone;
   4=relatively abnormal; 5=very abnormal

2. If scored 4-5, list the 3 most abnormal / noteworthy variables:
   1) ________________________
   2) ________________________
   3) ________________________

Patient-window heatmap card for blinded expert review
ID: P155   Window: W01

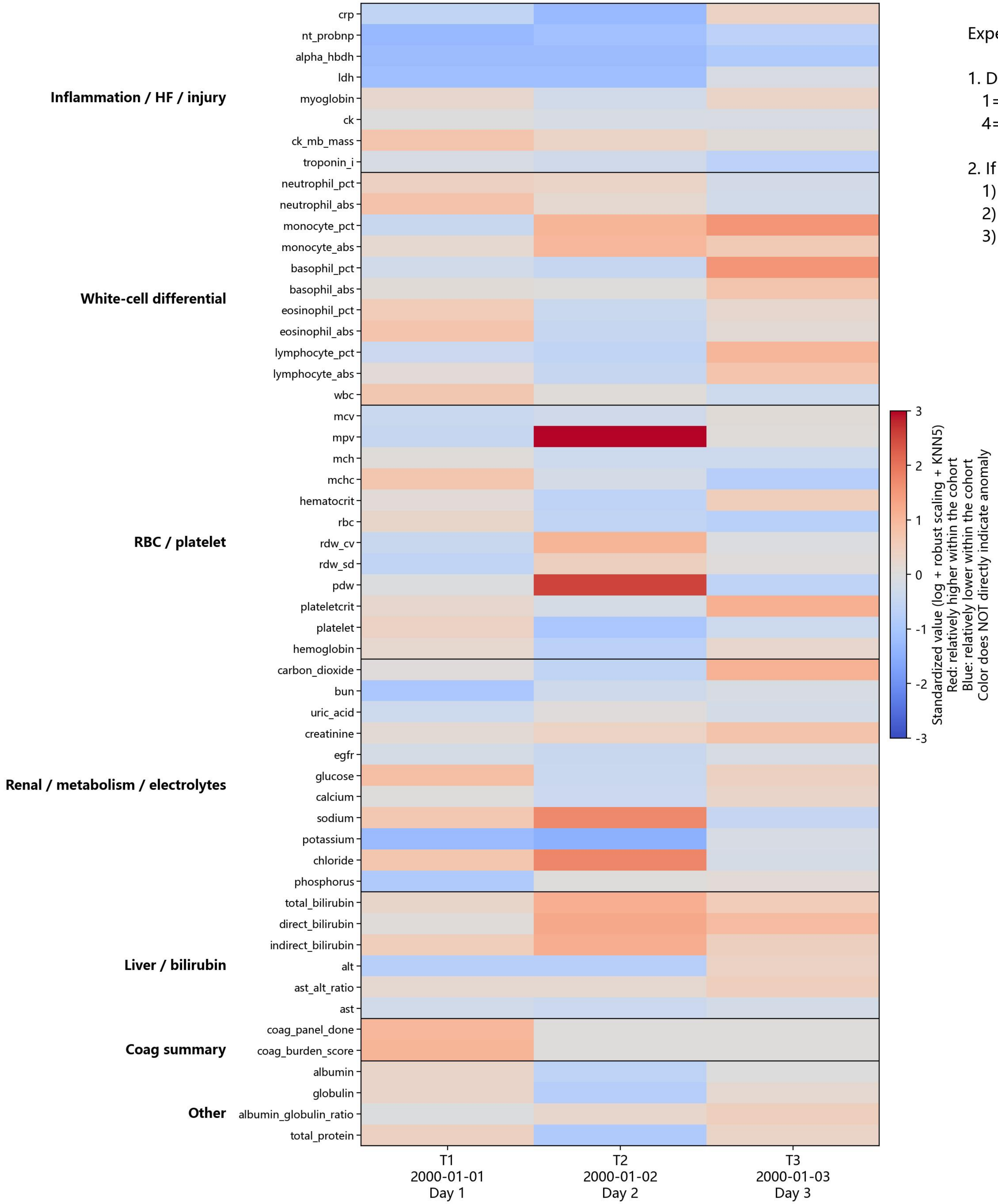

Expert review (blinded; no model score shown)

1. Degree of anomaly for this 3-point window (1-5):
   1=very typical; 2=relatively typical; 3=gray zone;
   4=relatively abnormal; 5=very abnormal

2. If scored 4-5, list the 3 most abnormal / noteworthy variables:
   1) ________________________
   2) ________________________
   3) ________________________

Patient-window heatmap card for blinded expert review
ID: P156   Window: W01

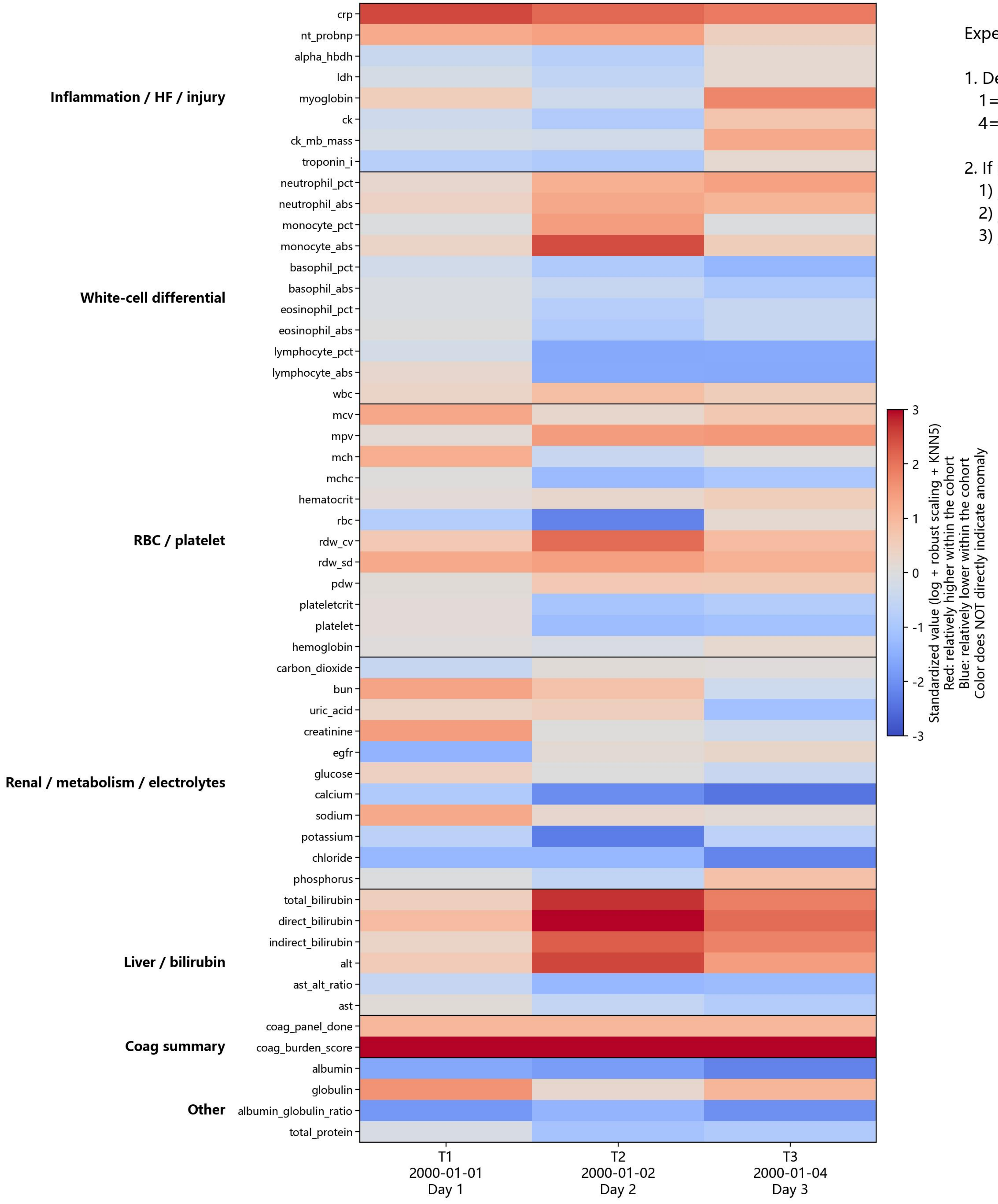

Expert review (blinded; no model score shown)

1. Degree of anomaly for this 3-point window (1-5):
   1=very typical; 2=relatively typical; 3=gray zone;
   4=relatively abnormal; 5=very abnormal

2. If scored 4-5, list the 3 most abnormal / noteworthy variables:
   1) ______________________
   2) ______________________
   3) ______________________

Patient-window heatmap card for blinded expert review
ID: P157   Window: W01

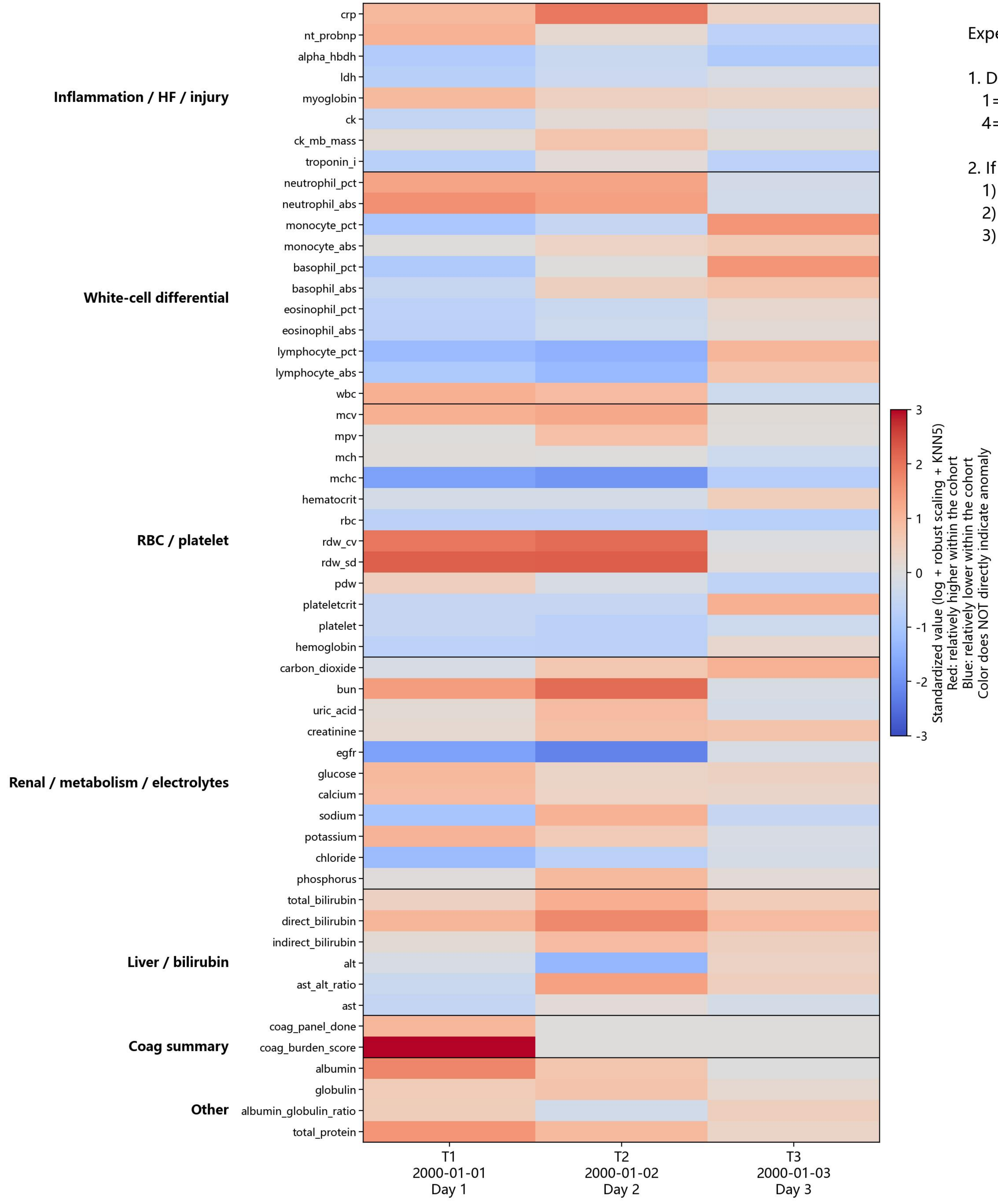

Expert review (blinded; no model score shown)

1. Degree of anomaly for this 3-point window (1-5):
   1=very typical; 2=relatively typical; 3=gray zone;
   4=relatively abnormal; 5=very abnormal

2. If scored 4-5, list the 3 most abnormal / noteworthy variables:
   1) ________________________
   2) ________________________
   3) ________________________

Patient-window heatmap card for blinded expert review
ID: P158   Window: W01

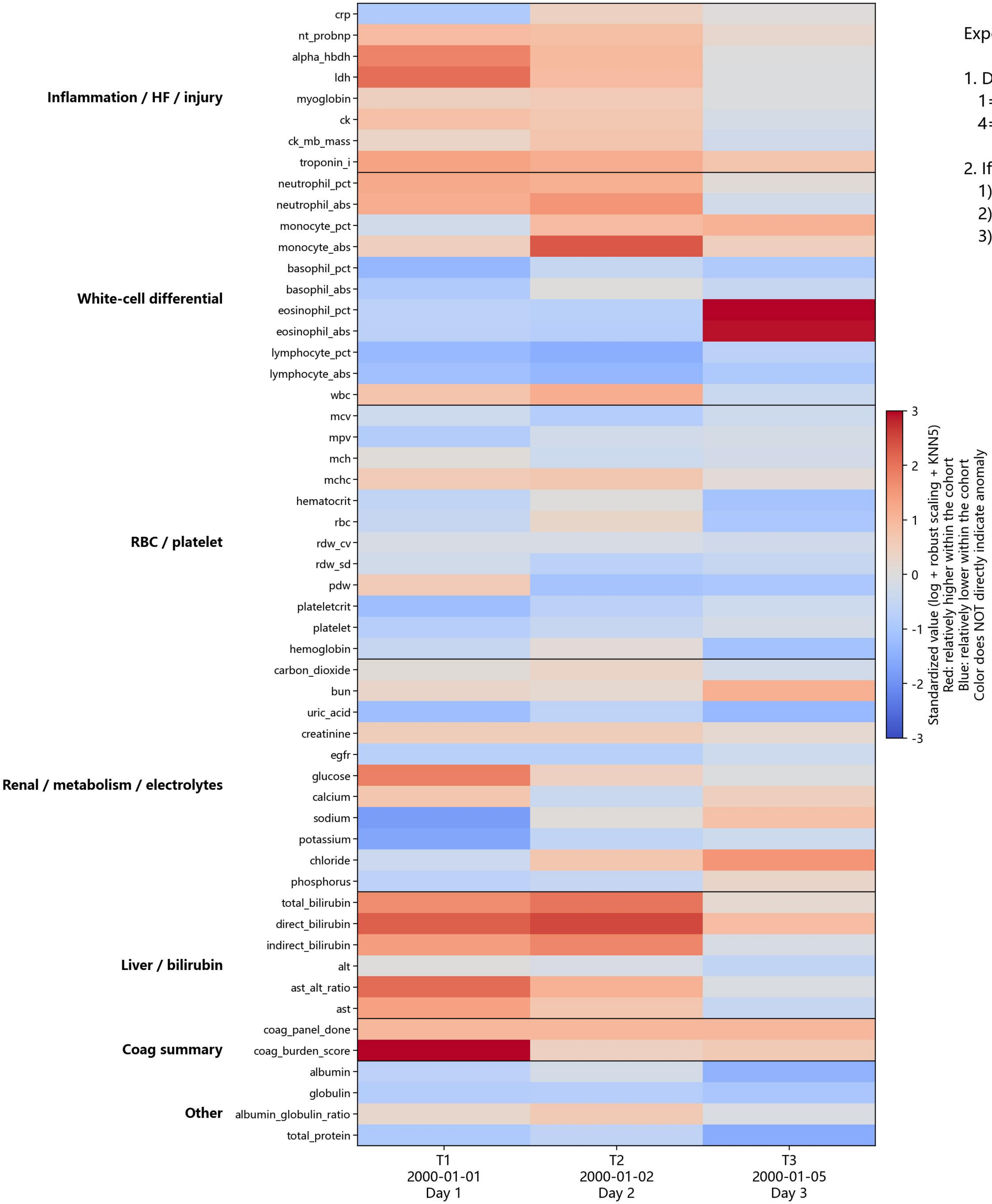

Expert review (blinded; no model score shown)

1. Degree of anomaly for this 3-point window (1-5):
   1=very typical; 2=relatively typical; 3=gray zone;
   4=relatively abnormal; 5=very abnormal

2. If scored 4-5, list the 3 most abnormal / noteworthy variables:
   1) ________________________
   2) ________________________
   3) ________________________

Patient-window heatmap card for blinded expert review
ID: P159   Window: W01

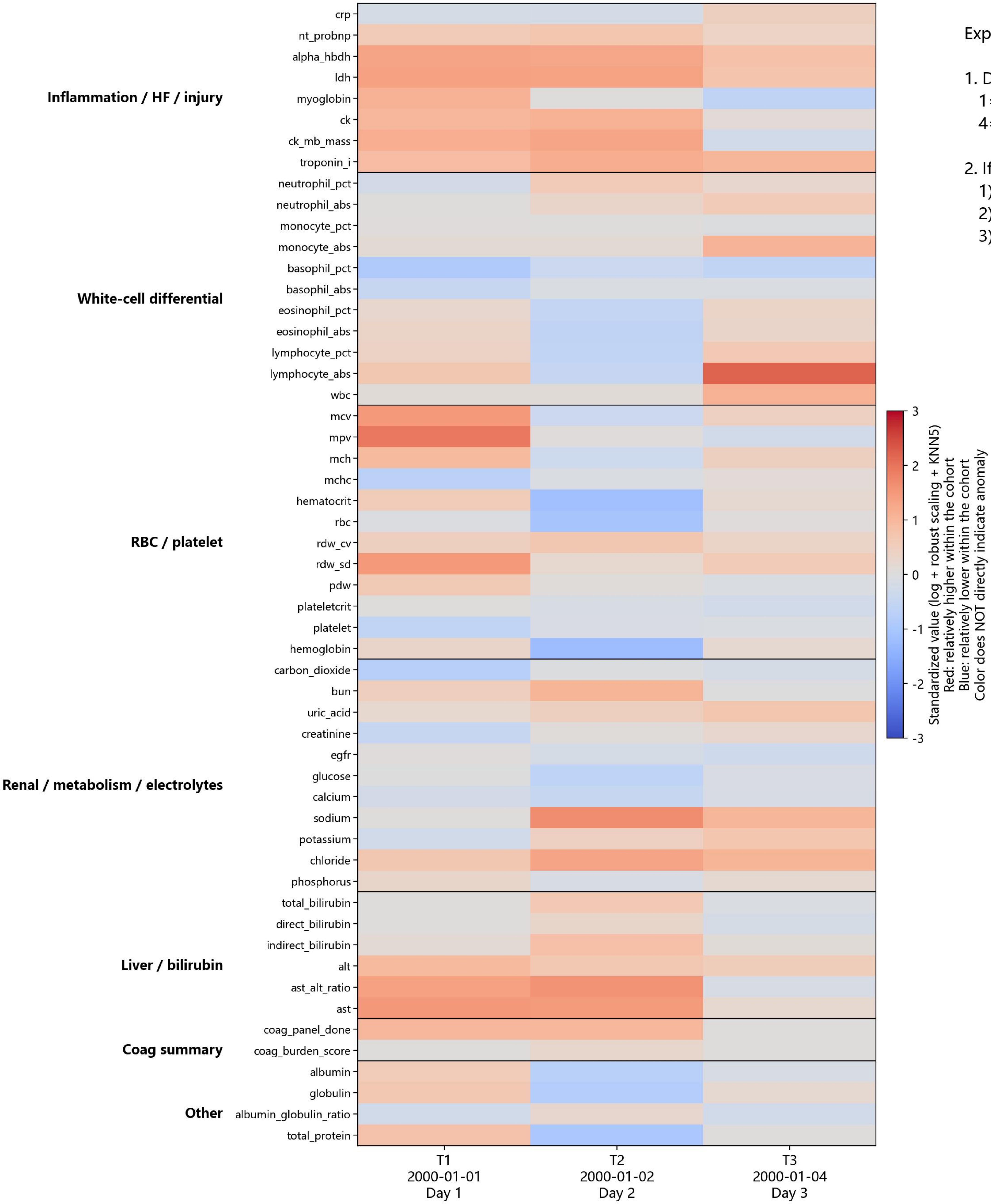

Expert review (blinded; no model score shown)

1. Degree of anomaly for this 3-point window (1-5):
   1=very typical; 2=relatively typical; 3=gray zone;
   4=relatively abnormal; 5=very abnormal

2. If scored 4-5, list the 3 most abnormal / noteworthy variables:
   1) ________________________
   2) ________________________
   3) ________________________

Patient-window heatmap card for blinded expert review
ID: P160   Window: W01

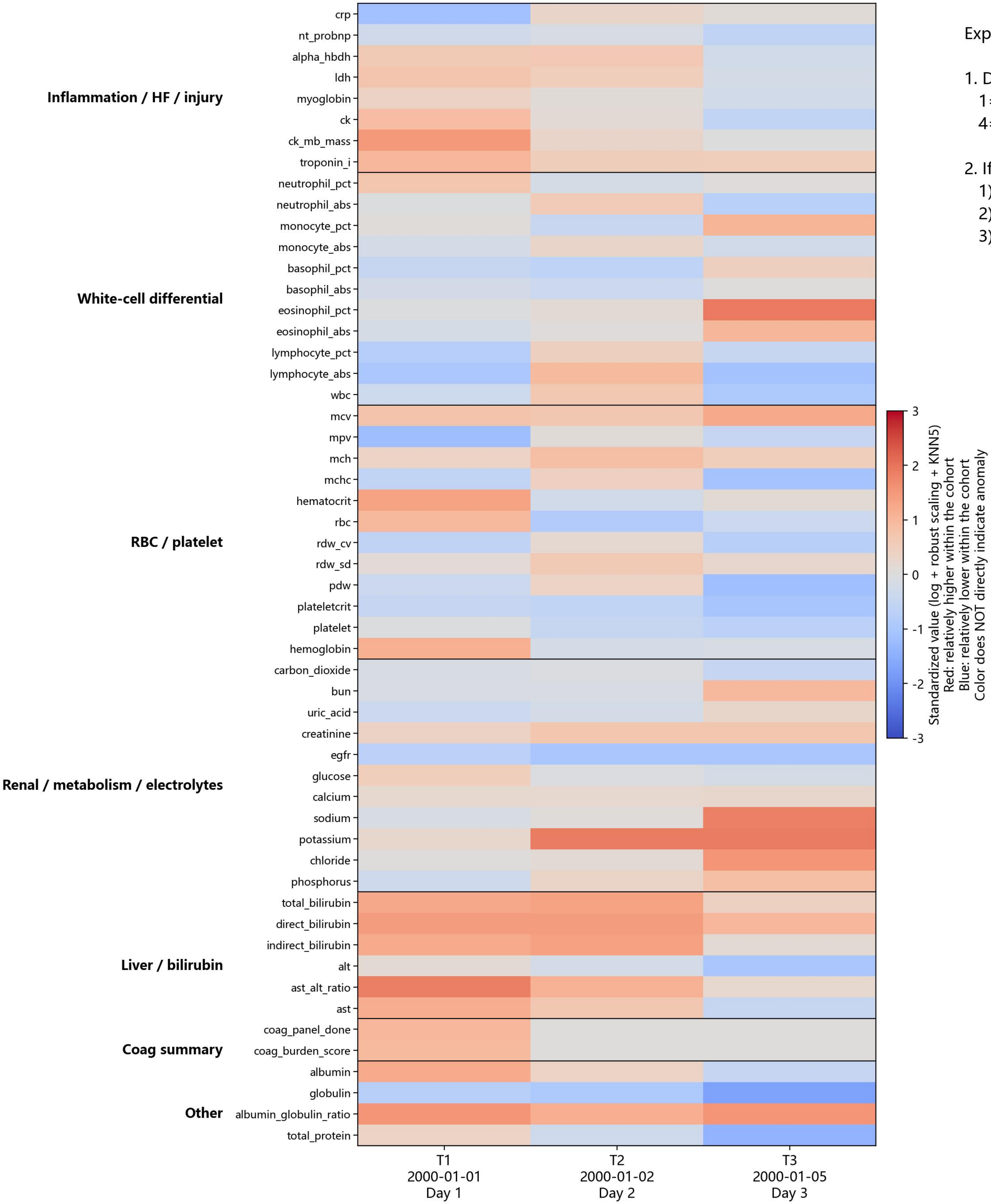

Expert review (blinded; no model score shown)

1. Degree of anomaly for this 3-point window (1-5):
   1=very typical; 2=relatively typical; 3=gray zone;
   4=relatively abnormal; 5=very abnormal

2. If scored 4-5, list the 3 most abnormal / noteworthy variables:
   1) ________________________
   2) ________________________
   3) ________________________

Patient-window heatmap card for blinded expert review
ID: P161   Window: W01

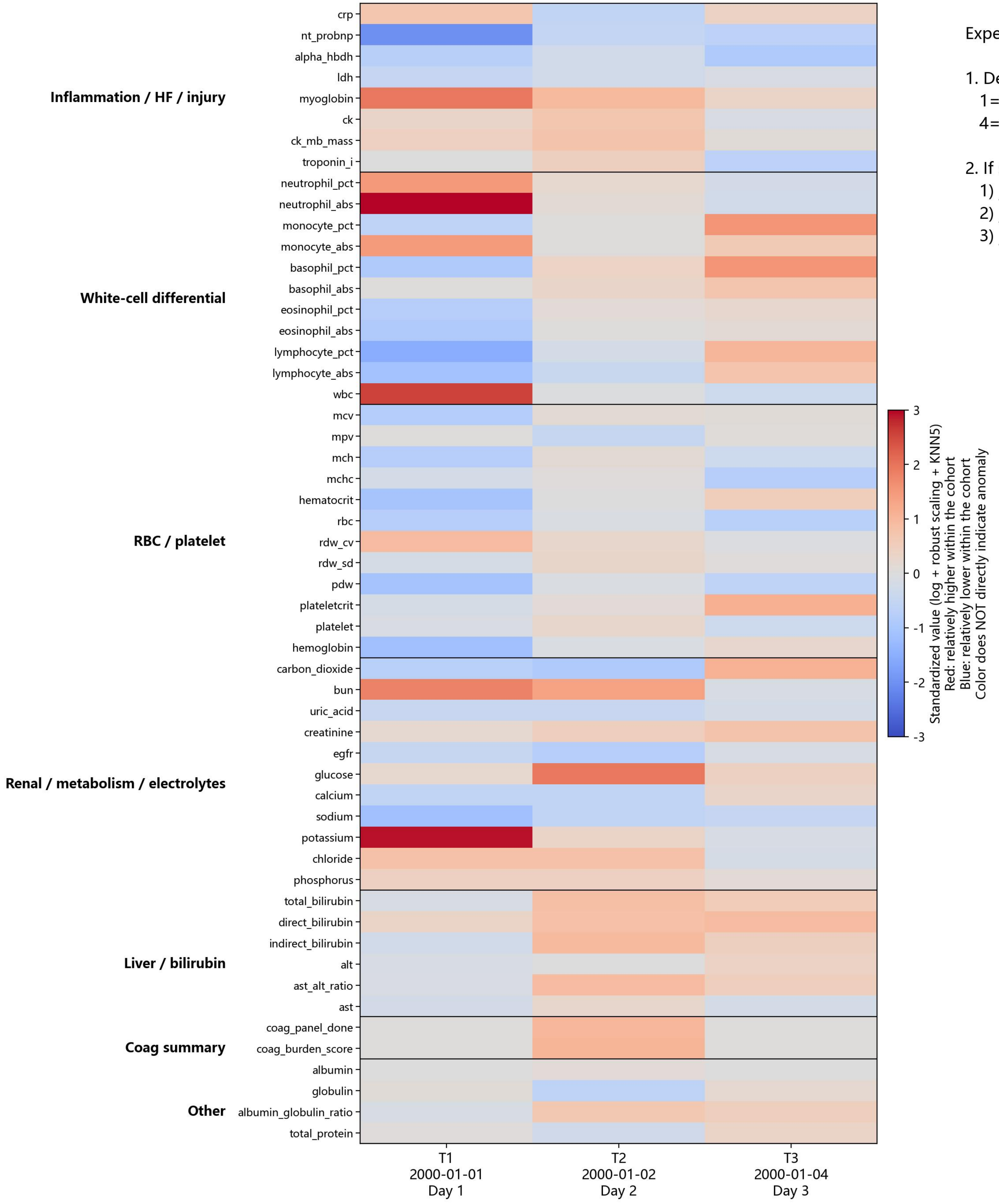

Expert review (blinded; no model score shown)

1. Degree of anomaly for this 3-point window (1-5):
   1=very typical; 2=relatively typical; 3=gray zone;
   4=relatively abnormal; 5=very abnormal

2. If scored 4-5, list the 3 most abnormal / noteworthy variables:
   1) ________________________
   2) ________________________
   3) ________________________

Patient-window heatmap card for blinded expert review
ID: P162   Window: W01

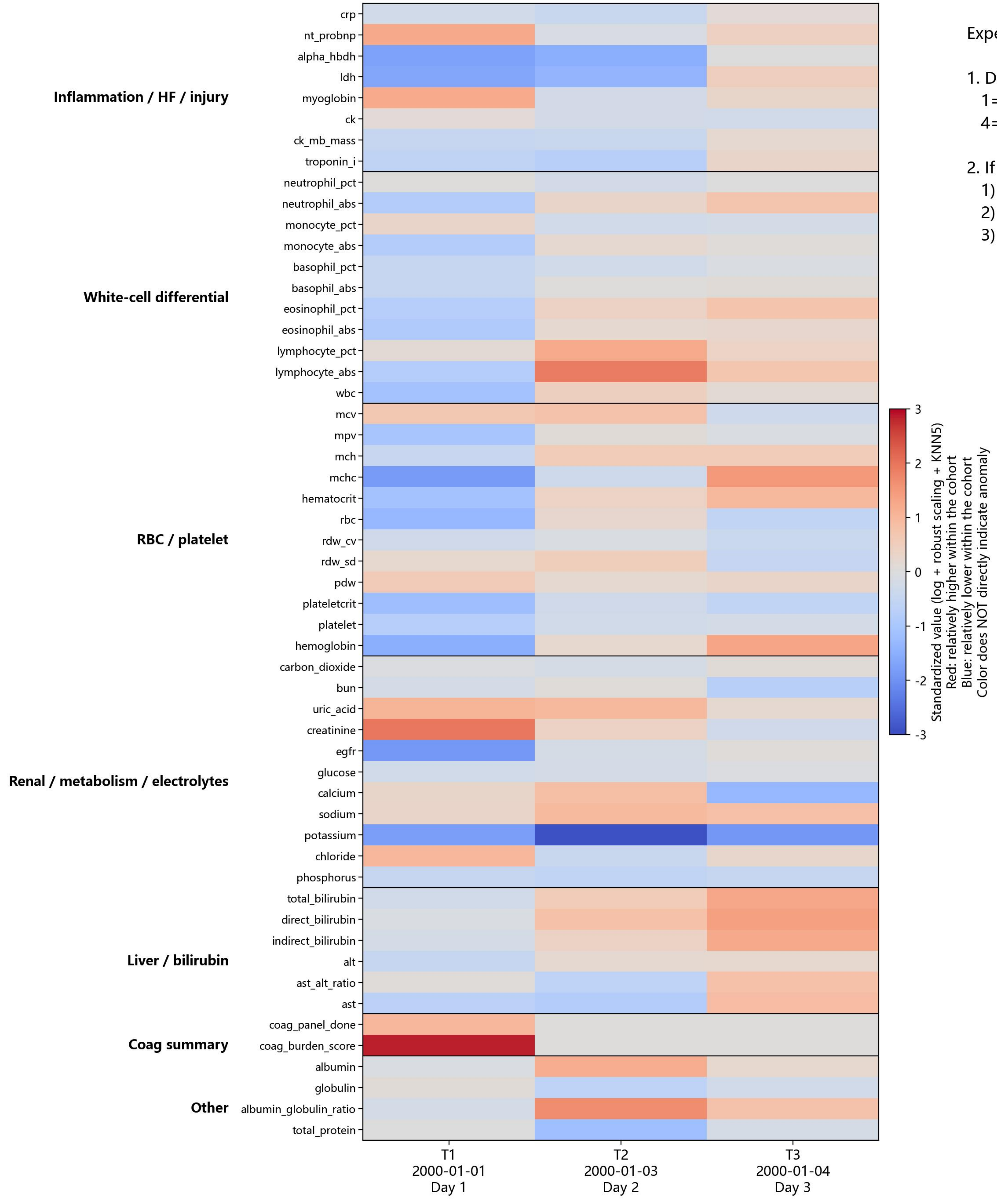

Expert review (blinded; no model score shown)

1. Degree of anomaly for this 3-point window (1-5):
   1=very typical; 2=relatively typical; 3=gray zone;
   4=relatively abnormal; 5=very abnormal

2. If scored 4-5, list the 3 most abnormal / noteworthy variables:
   1) ________________________
   2) ________________________
   3) ________________________

Patient-window heatmap card for blinded expert review
ID: P163   Window: W01

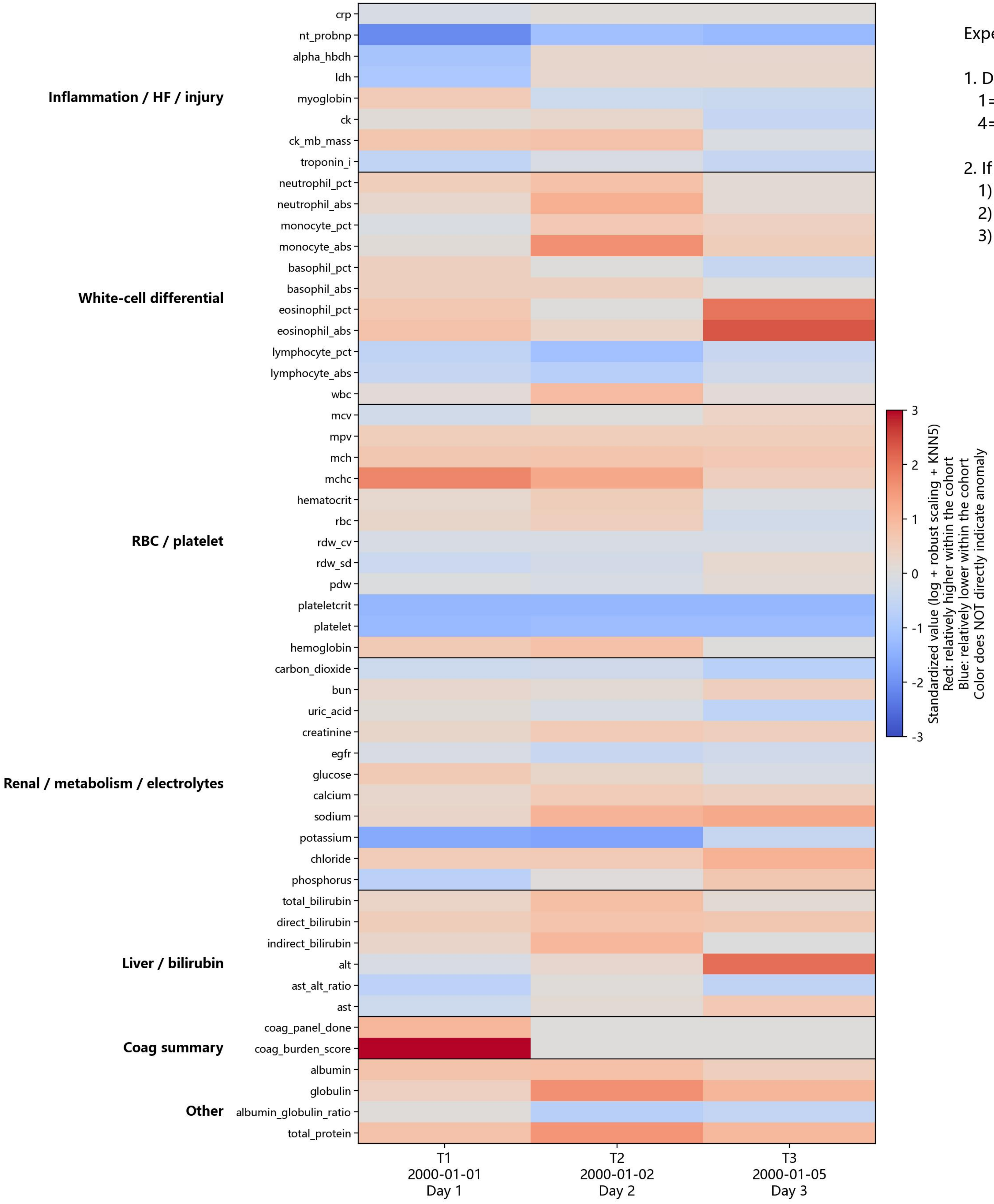

Expert review (blinded; no model score shown)

1. Degree of anomaly for this 3-point window (1-5):
   1=very typical; 2=relatively typical; 3=gray zone;
   4=relatively abnormal; 5=very abnormal

2. If scored 4-5, list the 3 most abnormal / noteworthy variables:
   1) ________________________
   2) ________________________
   3) ________________________

Patient-window heatmap card for blinded expert review
ID: P164   Window: W01

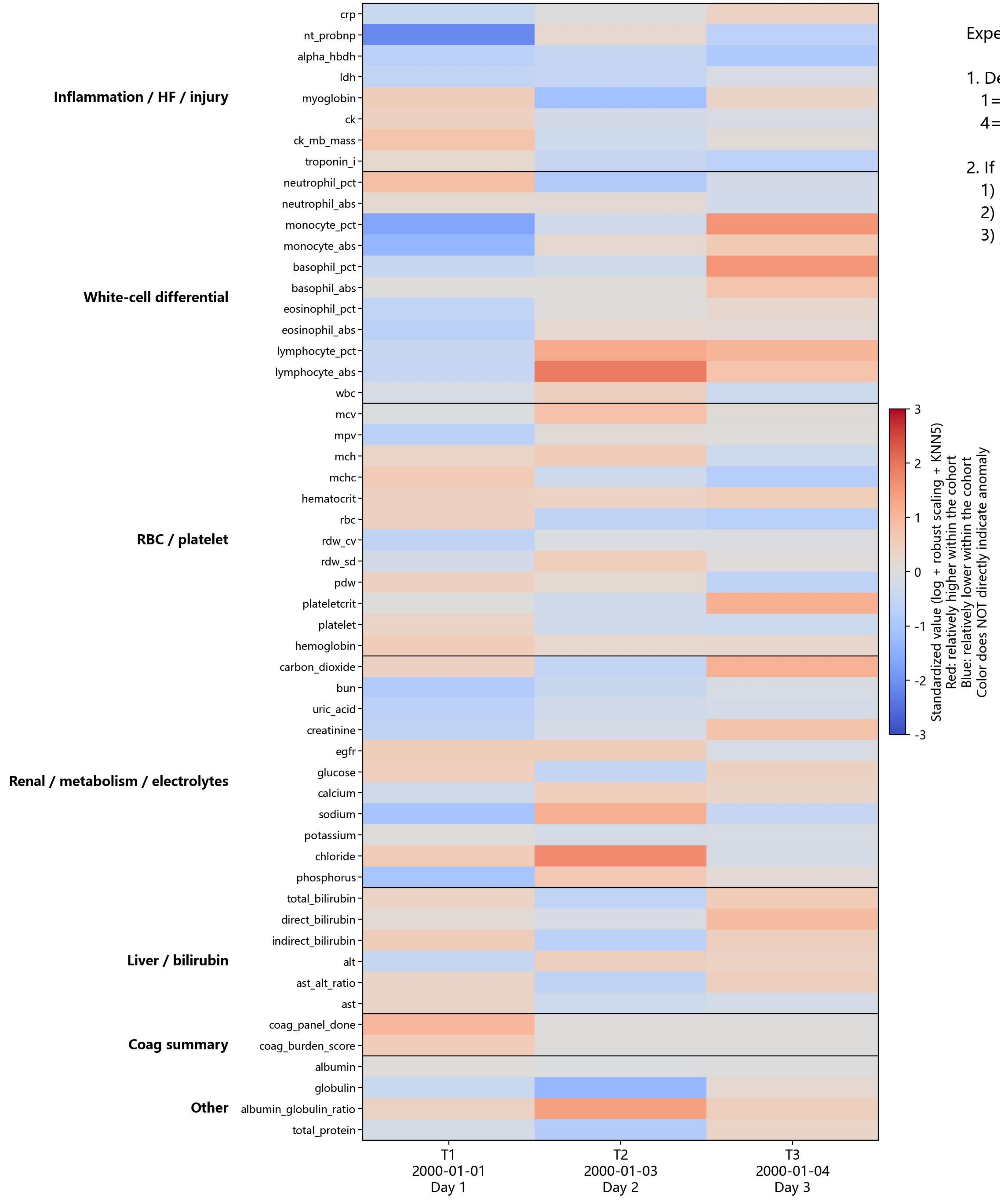

Expert review (blinded; no model score shown)

1. Degree of anomaly for this 3-point window (1-5):
   1=very typical; 2=relatively typical; 3=gray zone;
   4=relatively abnormal; 5=very abnormal

2. If scored 4-5, list the 3 most abnormal / noteworthy variables:
   1) ________________________
   2) ________________________
   3) ________________________

Patient-window heatmap card for blinded expert review
ID: P165   Window: W01

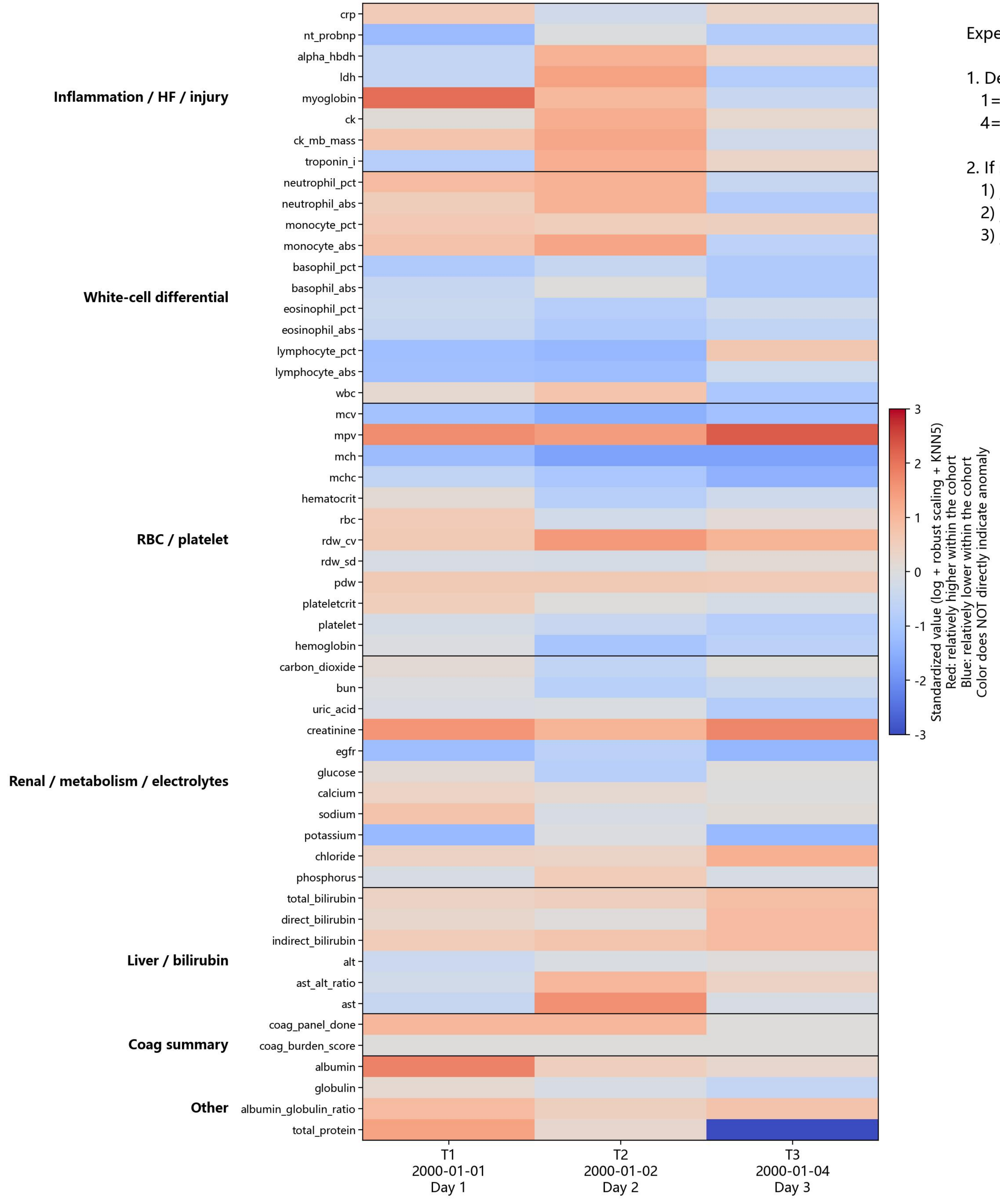

Expert review (blinded; no model score shown)

1. Degree of anomaly for this 3-point window (1-5):
   1=very typical; 2=relatively typical; 3=gray zone;
   4=relatively abnormal; 5=very abnormal

2. If scored 4-5, list the 3 most abnormal / noteworthy variables:
   1) ____________________
   2) ____________________
   3) ____________________

Patient-window heatmap card for blinded expert review
ID: P166   Window: W01

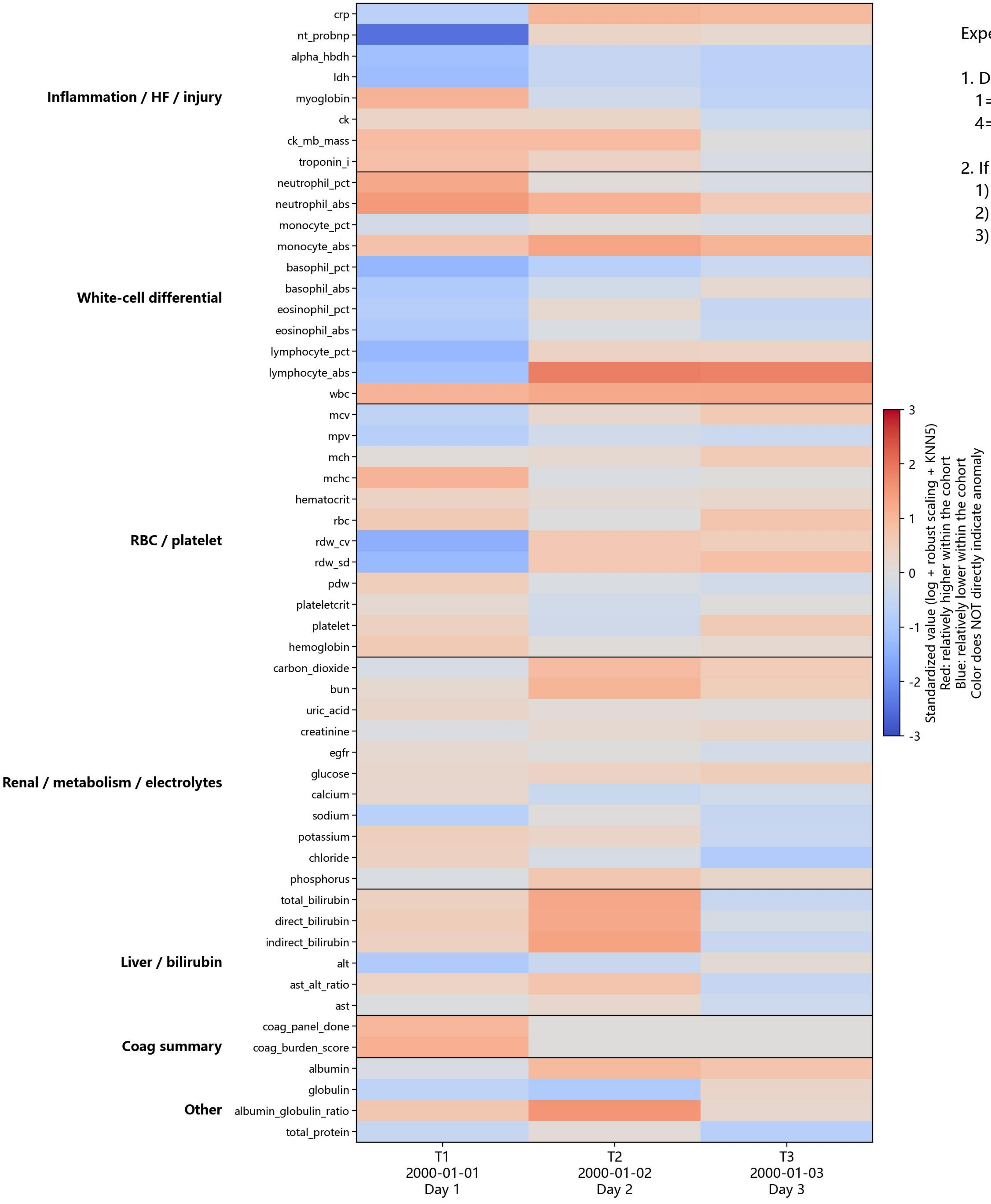

Expert review (blinded; no model score shown)

1. Degree of anomaly for this 3-point window (1-5):
   1=very typical; 2=relatively typical; 3=gray zone;
   4=relatively abnormal; 5=very abnormal

2. If scored 4-5, list the 3 most abnormal / noteworthy variables:
   1) ________________________
   2) ________________________
   3) ________________________

Patient-window heatmap card for blinded expert review
ID: P167   Window: W01

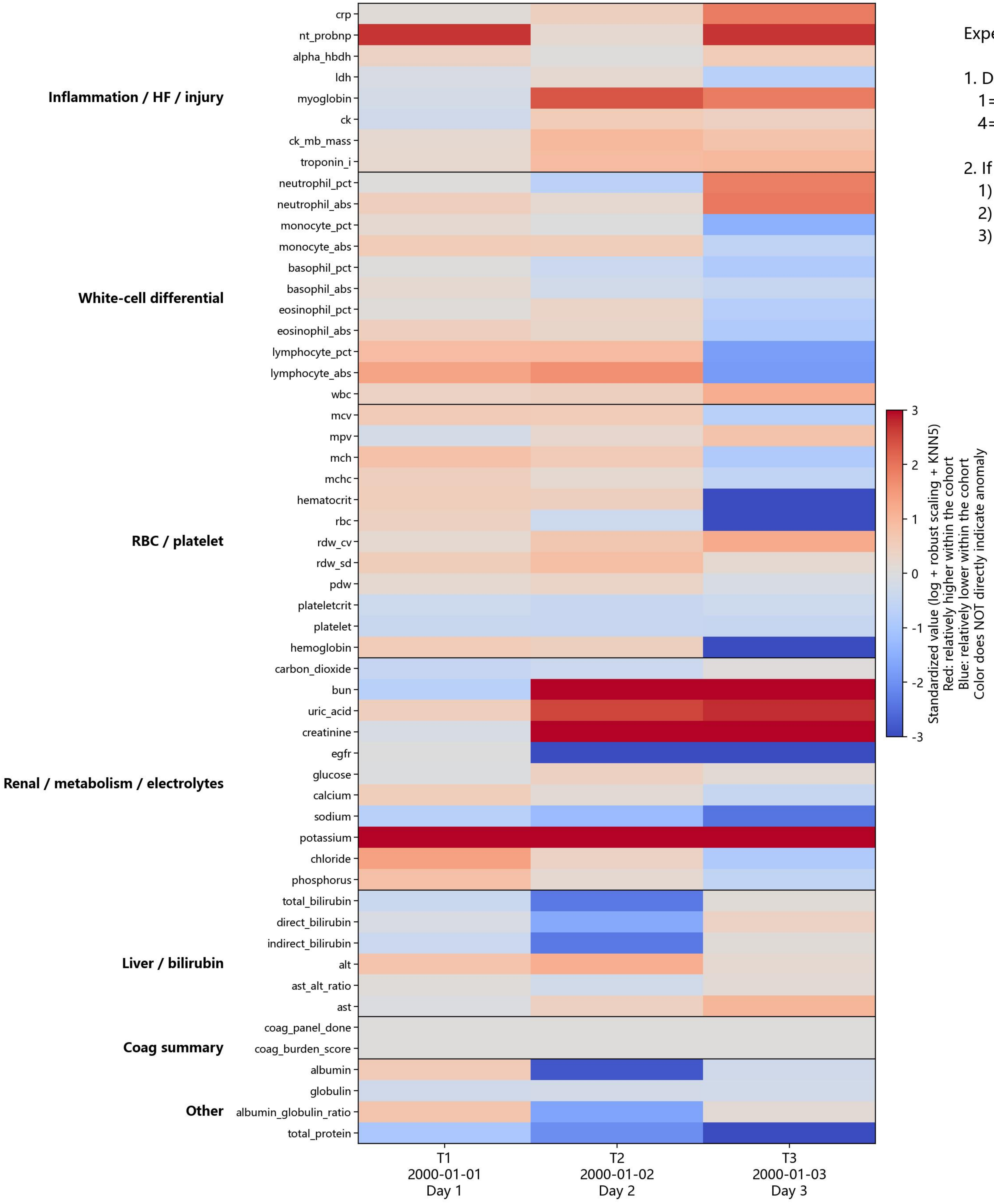

Expert review (blinded; no model score shown)

1. Degree of anomaly for this 3-point window (1-5):
   1=very typical; 2=relatively typical; 3=gray zone;
   4=relatively abnormal; 5=very abnormal

2. If scored 4-5, list the 3 most abnormal / noteworthy variables:
   1) ________________________
   2) ________________________
   3) ________________________

Patient-window heatmap card for blinded expert review
ID: P168   Window: W01

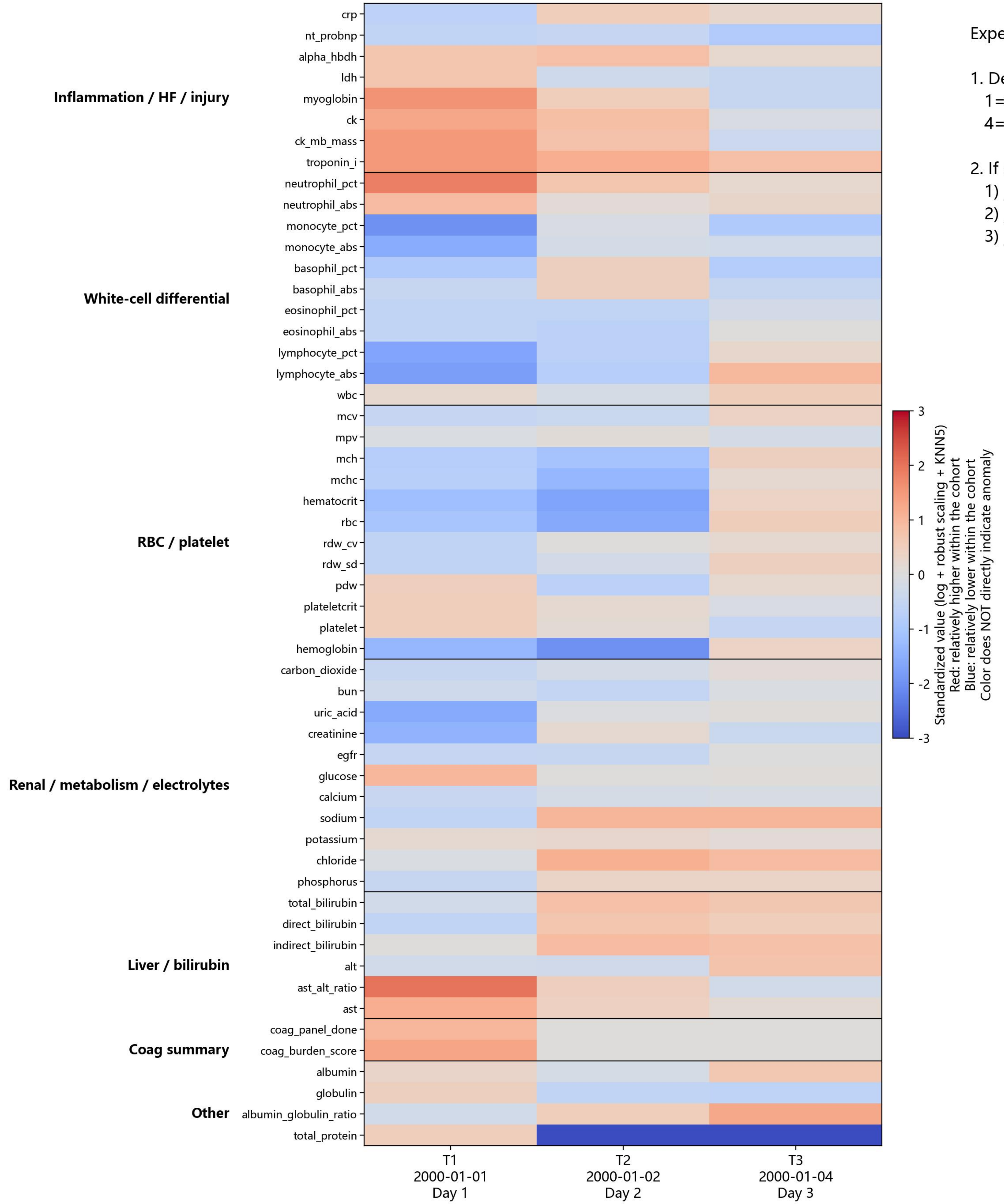

Expert review (blinded; no model score shown)

1. Degree of anomaly for this 3-point window (1-5):
   1=very typical; 2=relatively typical; 3=gray zone;
   4=relatively abnormal; 5=very abnormal

2. If scored 4-5, list the 3 most abnormal / noteworthy variables:
   1) ________________________
   2) ________________________
   3) ________________________

Patient-window heatmap card for blinded expert review
ID: P169   Window: W01

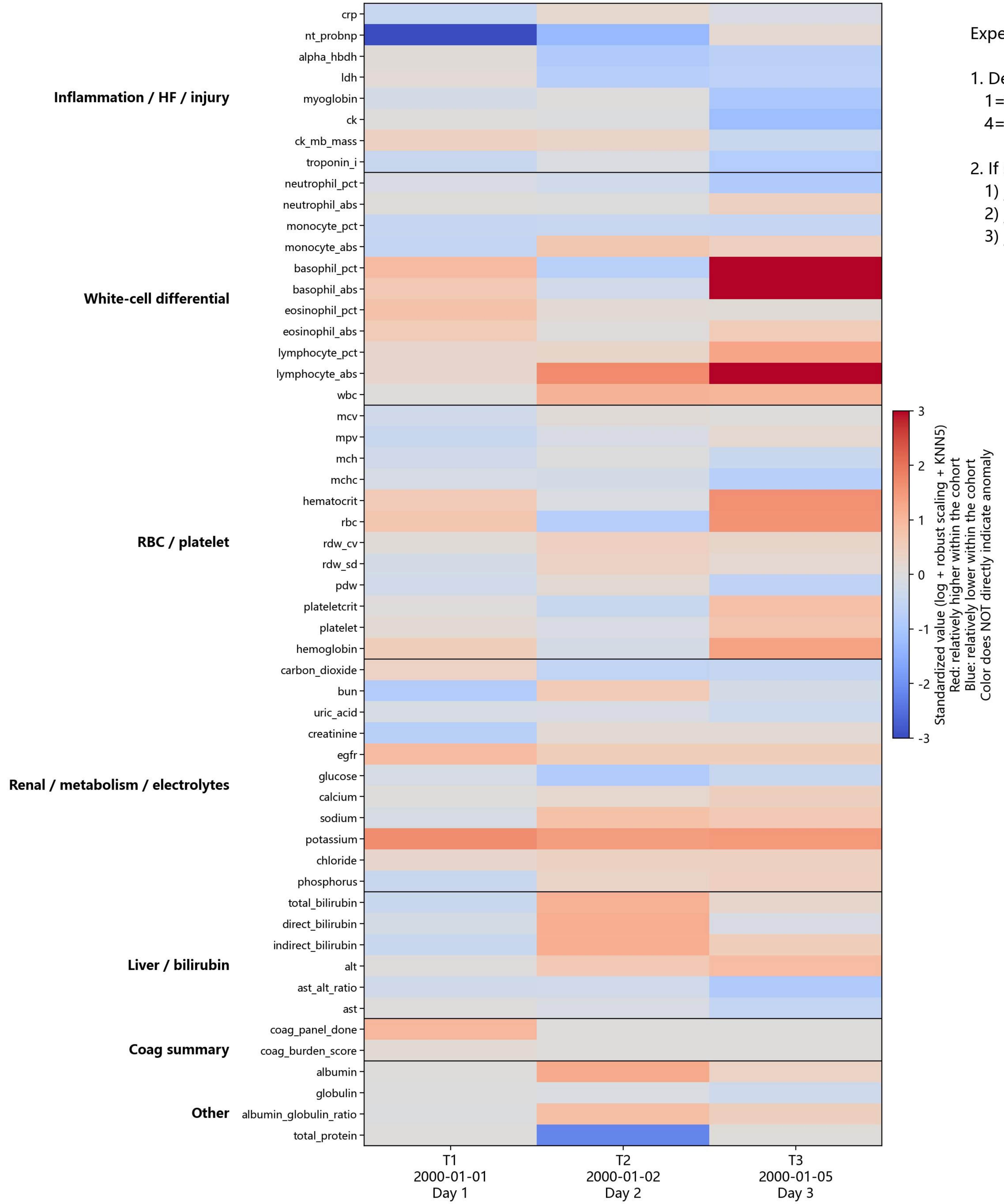

Expert review (blinded; no model score shown)

1. Degree of anomaly for this 3-point window (1-5):
   1=very typical; 2=relatively typical; 3=gray zone;
   4=relatively abnormal; 5=very abnormal

2. If scored 4-5, list the 3 most abnormal / noteworthy variables:
   1) ____________________________
   2) ____________________________
   3) ____________________________

Patient-window heatmap card for blinded expert review
ID: P170   Window: W01

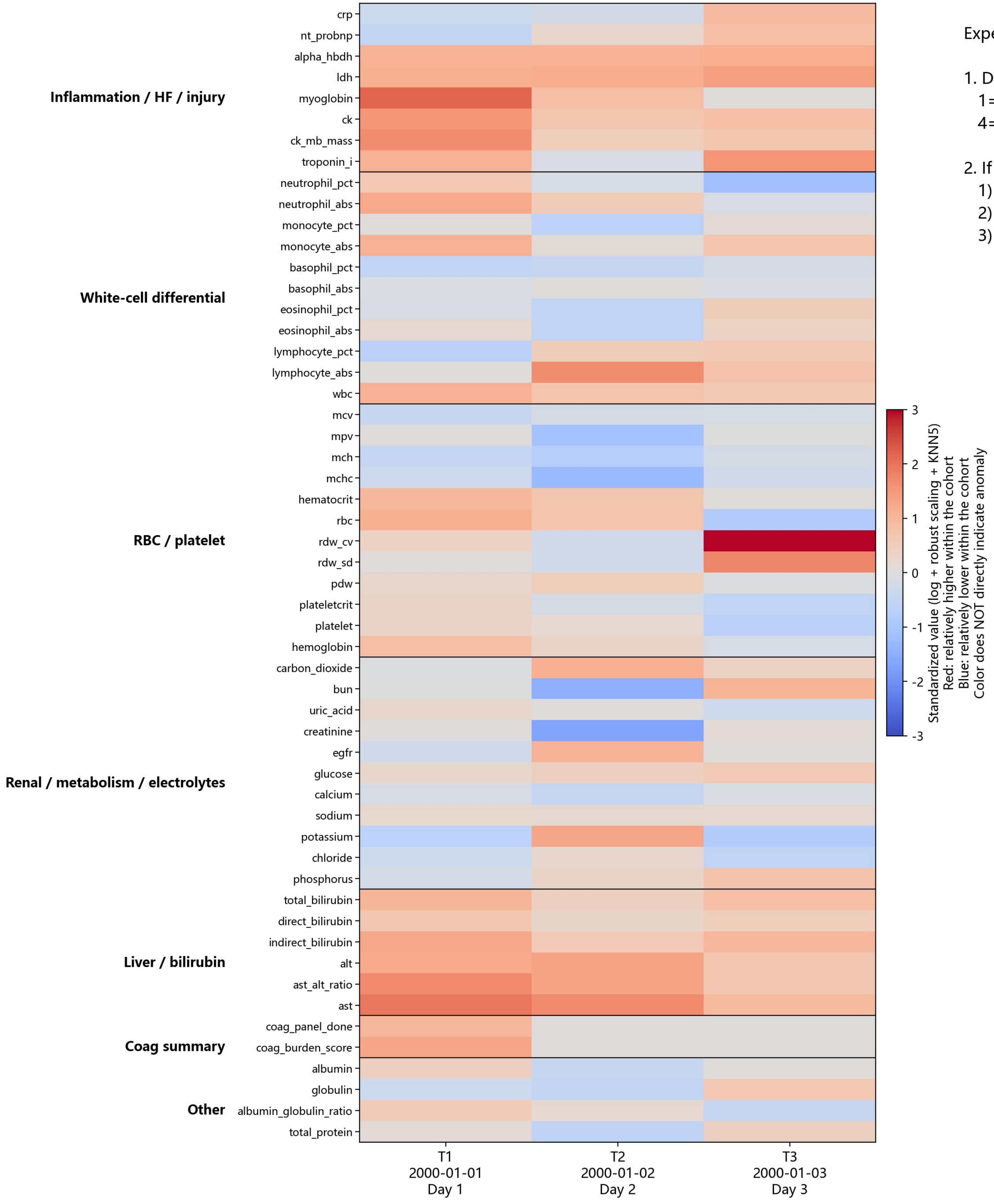

Expert review (blinded; no model score shown)

1. Degree of anomaly for this 3-point window (1-5):
   1=very typical; 2=relatively typical; 3=gray zone;
   4=relatively abnormal; 5=very abnormal

2. If scored 4-5, list the 3 most abnormal / noteworthy variables:
   1) ________________________
   2) ________________________
   3) ________________________

Patient-window heatmap card for blinded expert review
ID: P171   Window: W01

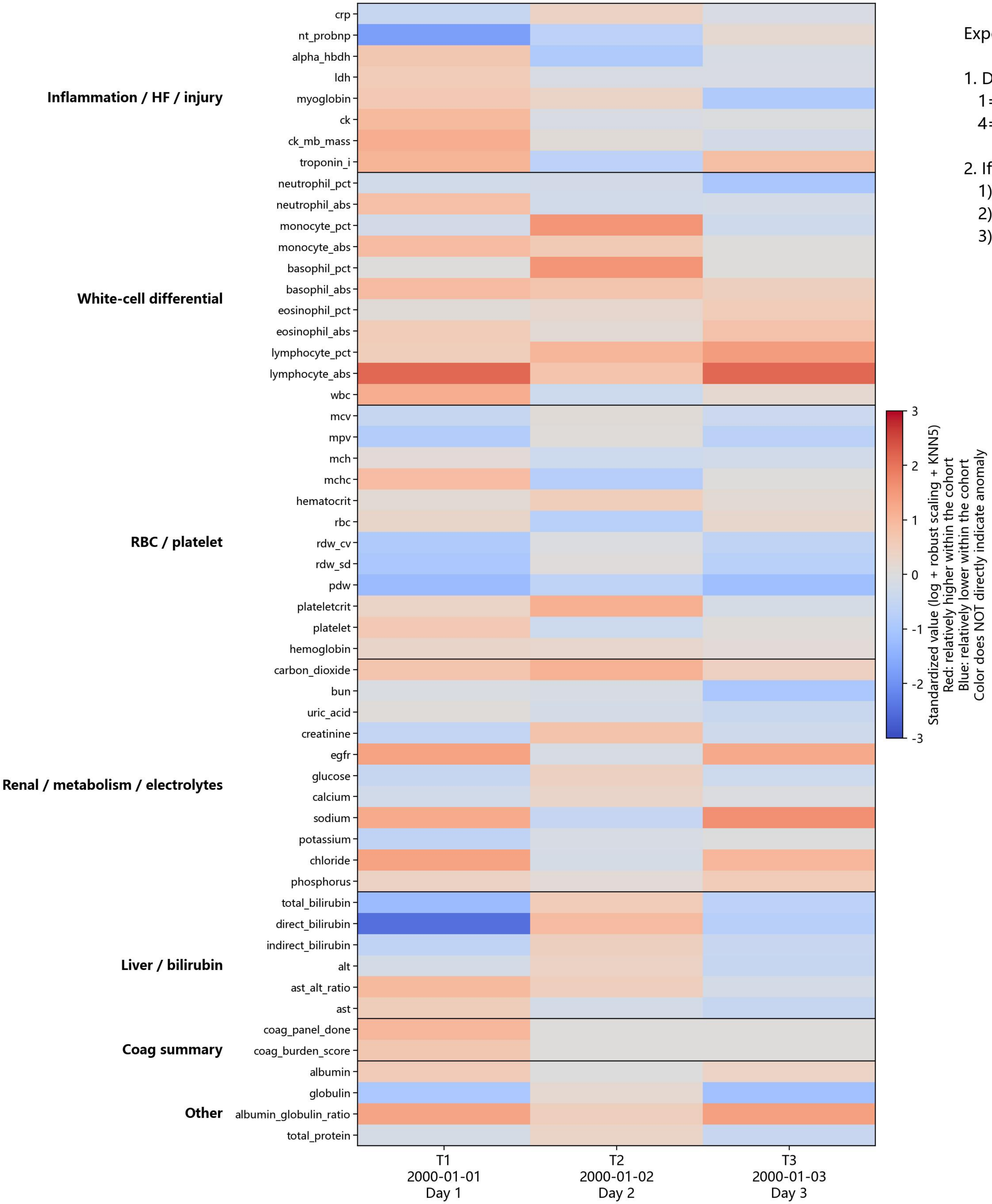

Expert review (blinded; no model score shown)

1. Degree of anomaly for this 3-point window (1-5):
   1=very typical; 2=relatively typical; 3=gray zone;
   4=relatively abnormal; 5=very abnormal

2. If scored 4-5, list the 3 most abnormal / noteworthy variables:
   1) ________________________
   2) ________________________
   3) ________________________

Patient-window heatmap card for blinded expert review
ID: P172   Window: W01

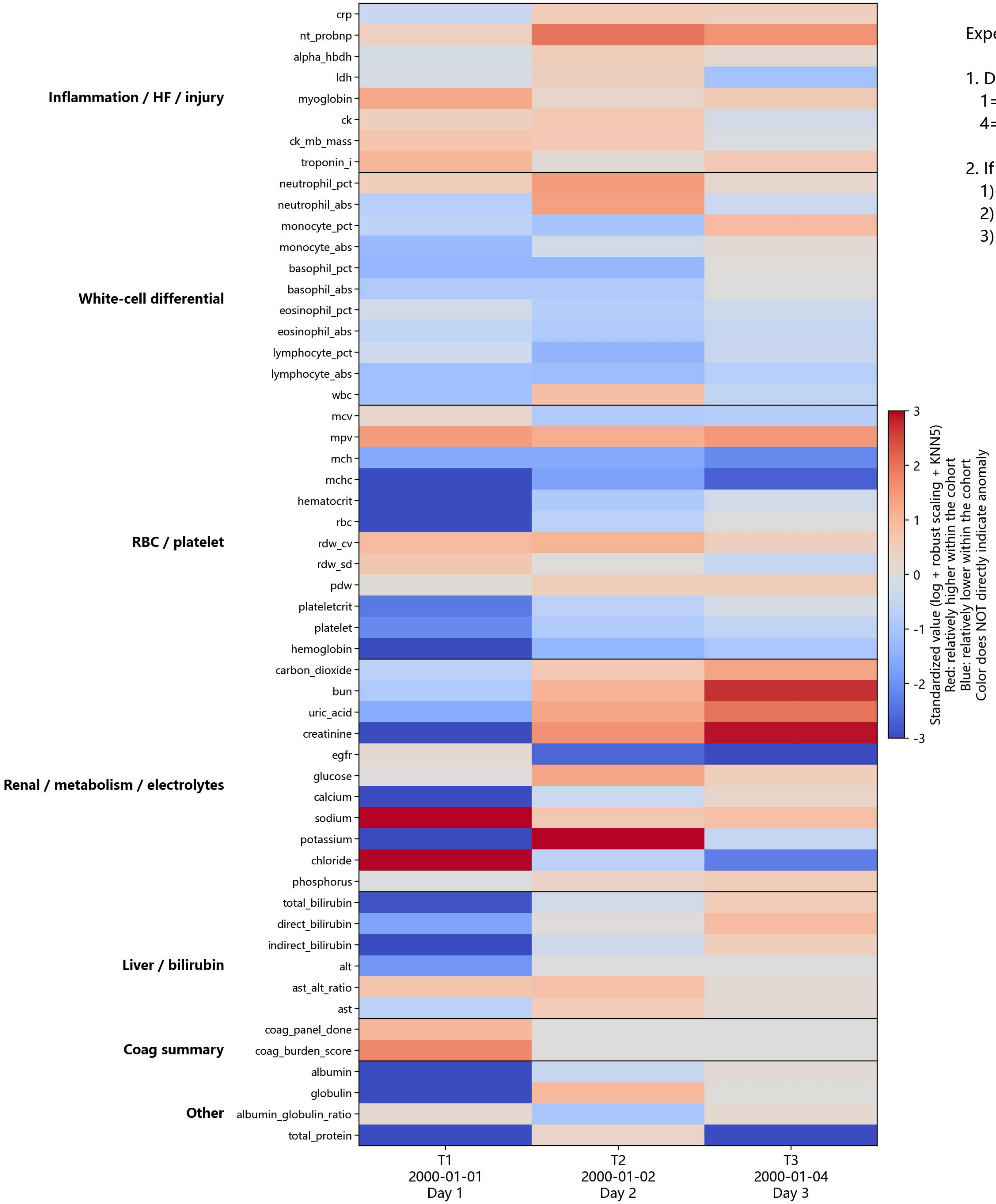

Expert review (blinded; no model score shown)

1. Degree of anomaly for this 3-point window (1-5):
   1=very typical; 2=relatively typical; 3=gray zone;
   4=relatively abnormal; 5=very abnormal

2. If scored 4-5, list the 3 most abnormal / noteworthy variables:
   1) ________________________
   2) ________________________
   3) ________________________

Patient-window heatmap card for blinded expert review
ID: P173   Window: W01

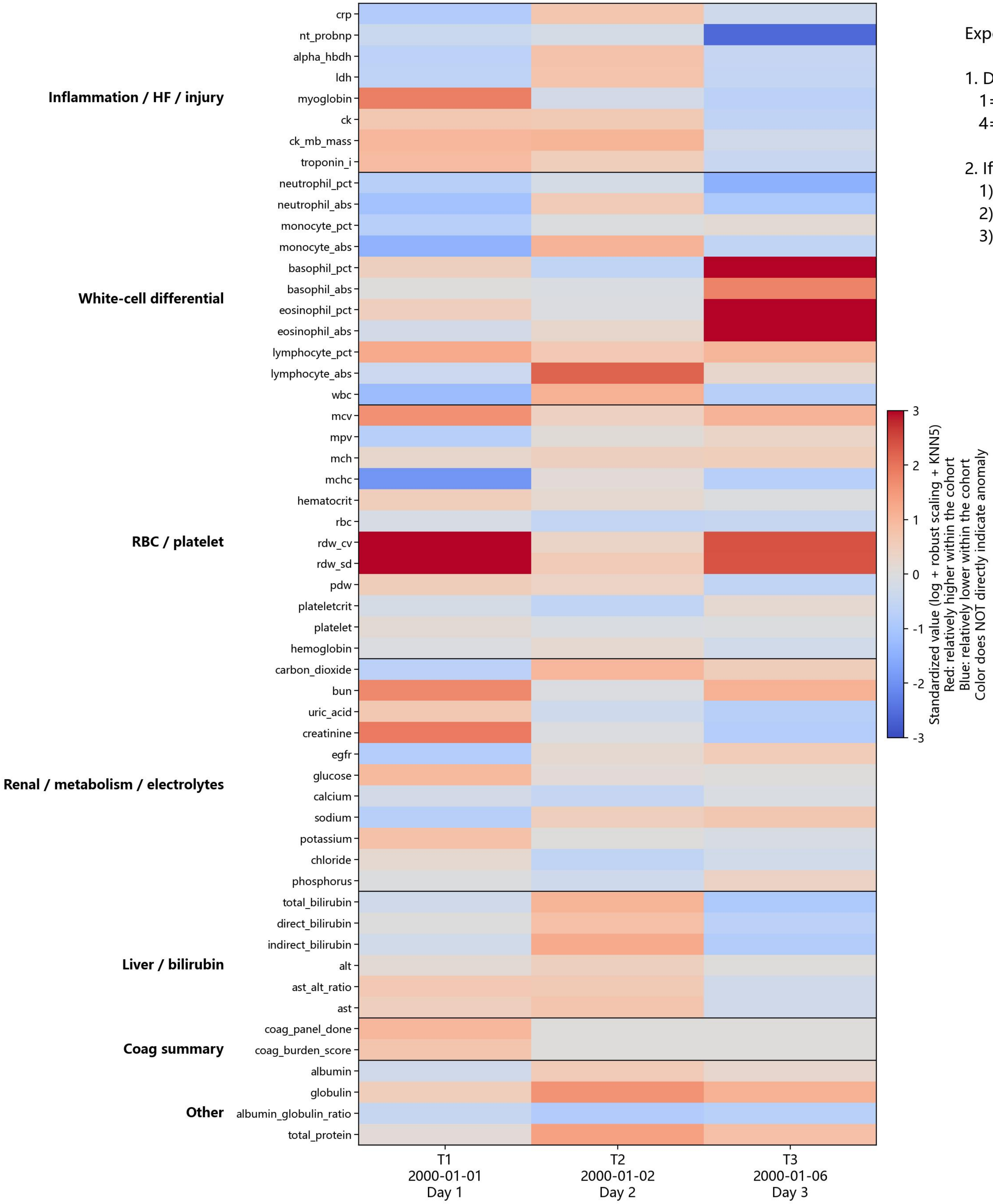

Expert review (blinded; no model score shown)

1. Degree of anomaly for this 3-point window (1-5):
1=very typical; 2=relatively typical; 3=gray zone;
4=relatively abnormal; 5=very abnormal

2. If scored 4-5, list the 3 most abnormal / noteworthy variables:
1) ____________________
2) ____________________
3) ____________________

Patient-window heatmap card for blinded expert review
ID: P174 Window: W01

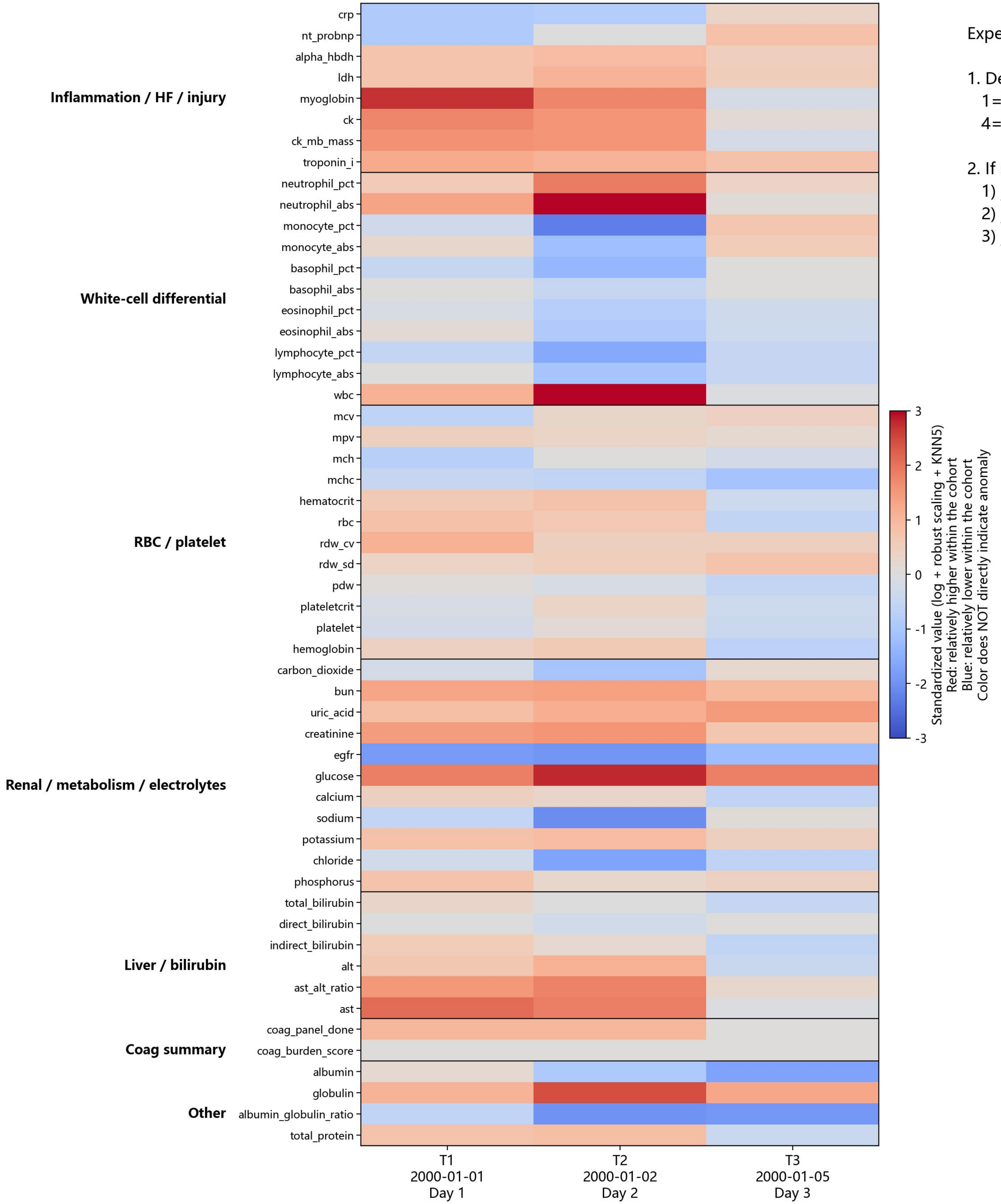

Expert review (blinded; no model score shown)

1. Degree of anomaly for this 3-point window (1-5):
1=very typical; 2=relatively typical; 3=gray zone;
4=relatively abnormal; 5=very abnormal

2. If scored 4-5, list the 3 most abnormal / noteworthy variables:
1) ________________________
2) ________________________
3) ________________________

Patient-window heatmap card for blinded expert review
ID: P175   Window: W01

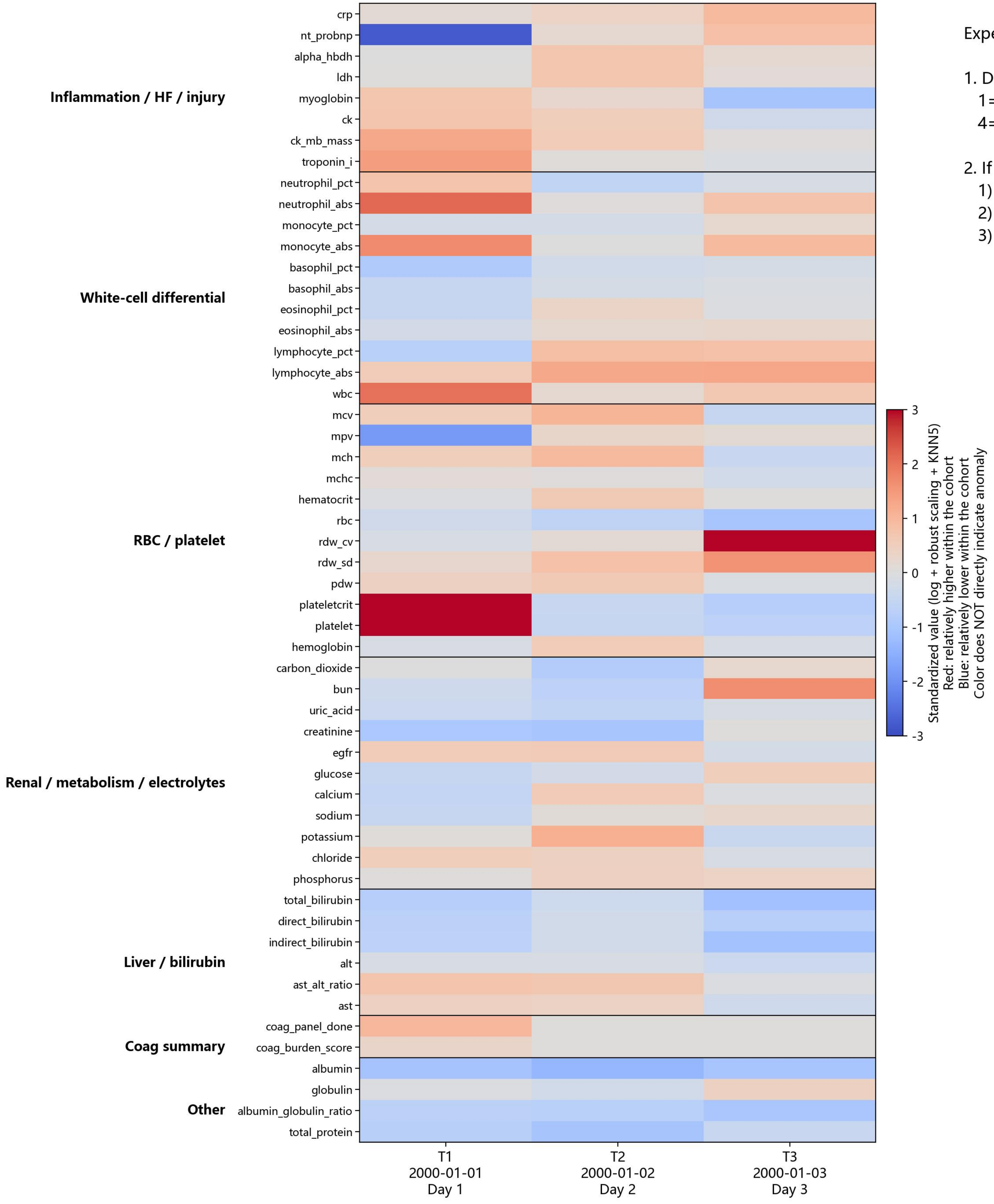

Expert review (blinded; no model score shown)

1. Degree of anomaly for this 3-point window (1-5):
   1=very typical; 2=relatively typical; 3=gray zone;
   4=relatively abnormal; 5=very abnormal

2. If scored 4-5, list the 3 most abnormal / noteworthy variables:
   1) ________________________
   2) ________________________
   3) ________________________

Patient-window heatmap card for blinded expert review
ID: P176 Window: W01

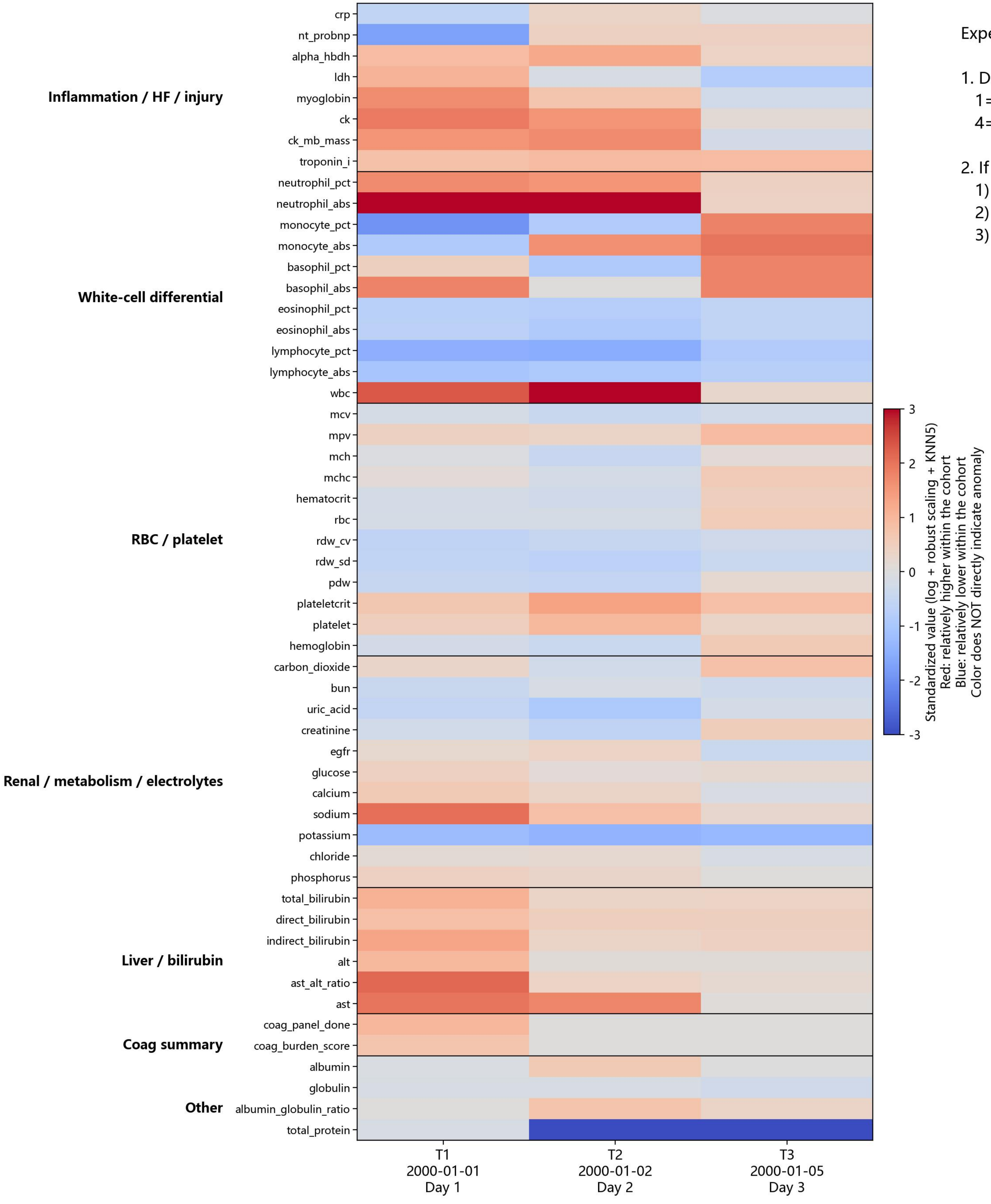

Expert review (blinded; no model score shown)

1. Degree of anomaly for this 3-point window (1-5):
   1=very typical; 2=relatively typical; 3=gray zone;
   4=relatively abnormal; 5=very abnormal

2. If scored 4-5, list the 3 most abnormal / noteworthy variables:
   1) __________________________
   2) __________________________
   3) __________________________

Patient-window heatmap card for blinded expert review
ID: P177   Window: W01

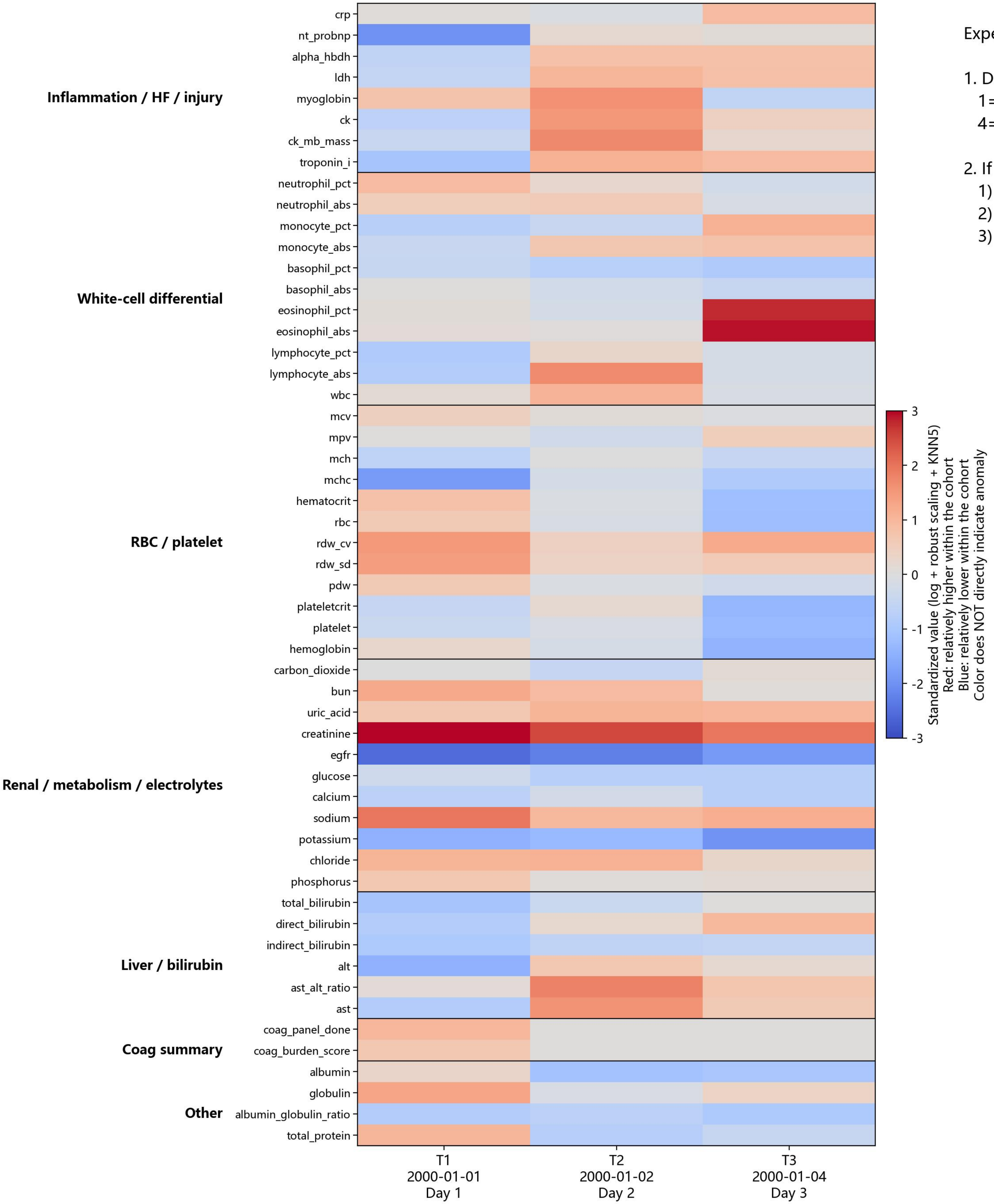

Expert review (blinded; no model score shown)

1. Degree of anomaly for this 3-point window (1-5):
   1=very typical; 2=relatively typical; 3=gray zone;
   4=relatively abnormal; 5=very abnormal

2. If scored 4-5, list the 3 most abnormal / noteworthy variables:
   1) ________________________
   2) ________________________
   3) ________________________

Patient-window heatmap card for blinded expert review
ID: P178   Window: W01

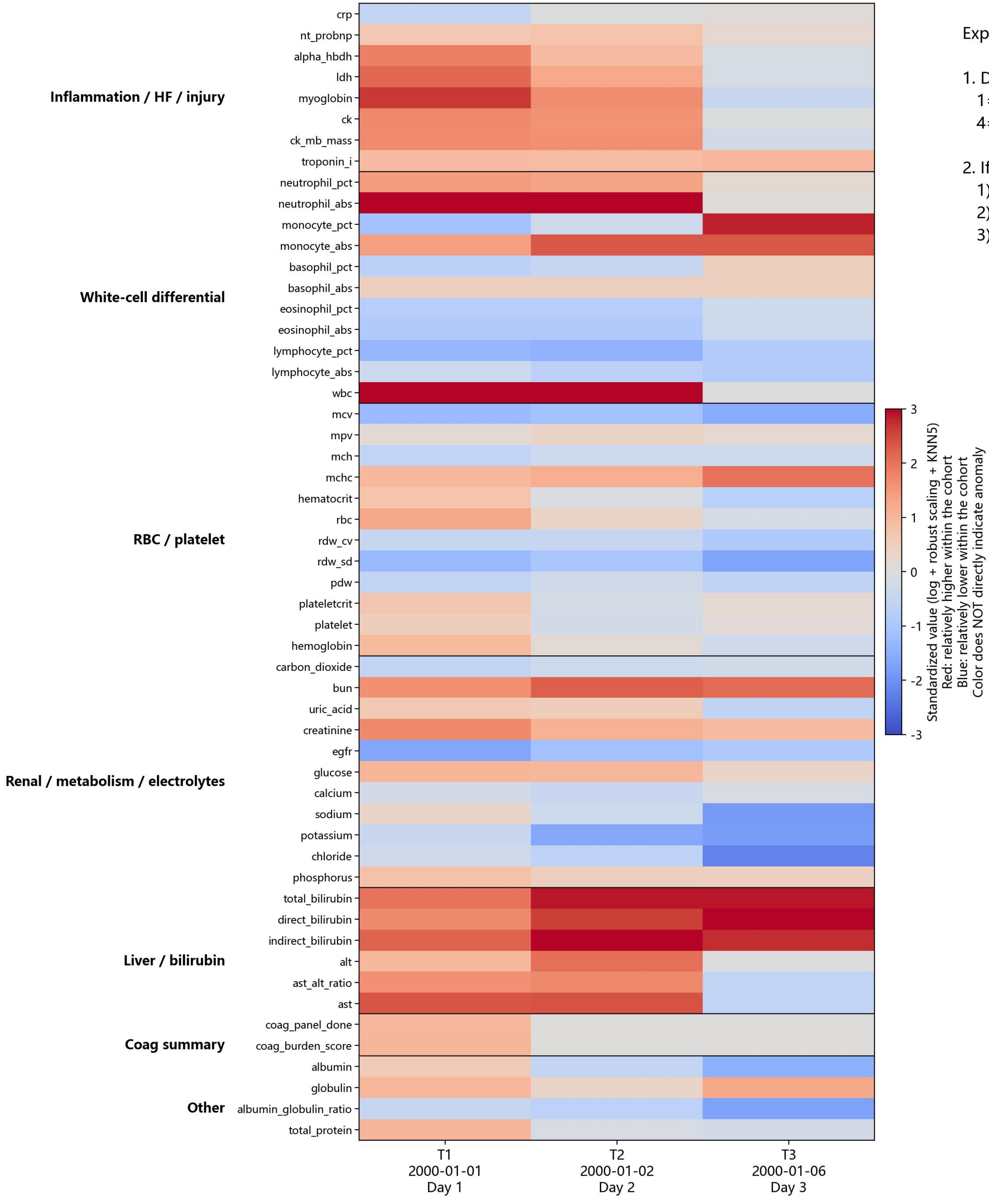

Expert review (blinded; no model score shown)

1. Degree of anomaly for this 3-point window (1-5):
1=very typical; 2=relatively typical; 3=gray zone;
4=relatively abnormal; 5=very abnormal

2. If scored 4-5, list the 3 most abnormal / noteworthy variables:
1) ________________________
2) ________________________
3) ________________________

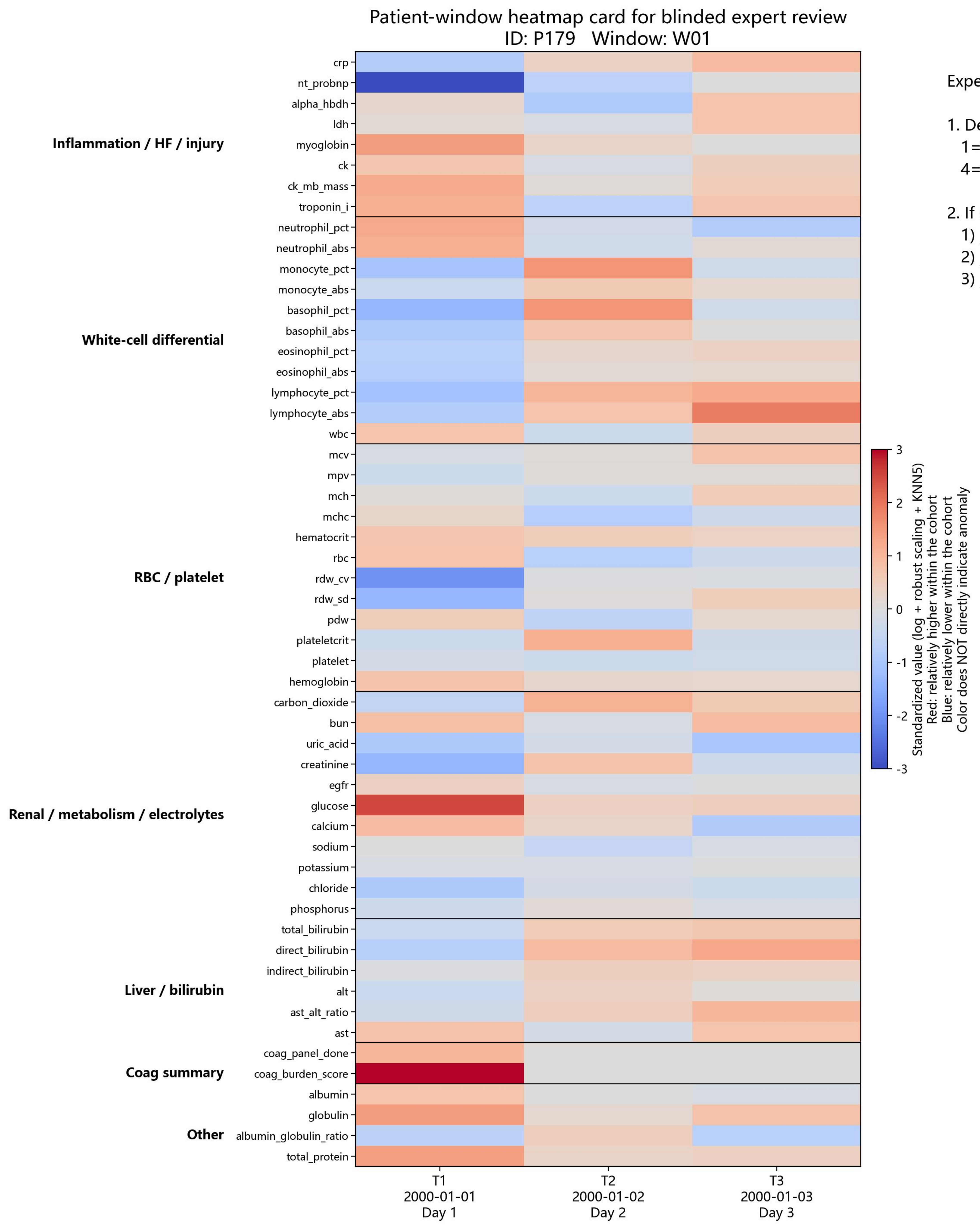

Expert review (blinded; no model score shown)

1. Degree of anomaly for this 3-point window (1-5):
   1=very typical; 2=relatively typical; 3=gray zone;
   4=relatively abnormal; 5=very abnormal

2. If scored 4-5, list the 3 most abnormal / noteworthy variables:
   1) ____________________
   2) ____________________
   3) ____________________

Patient-window heatmap card for blinded expert review
ID: P180   Window: W01

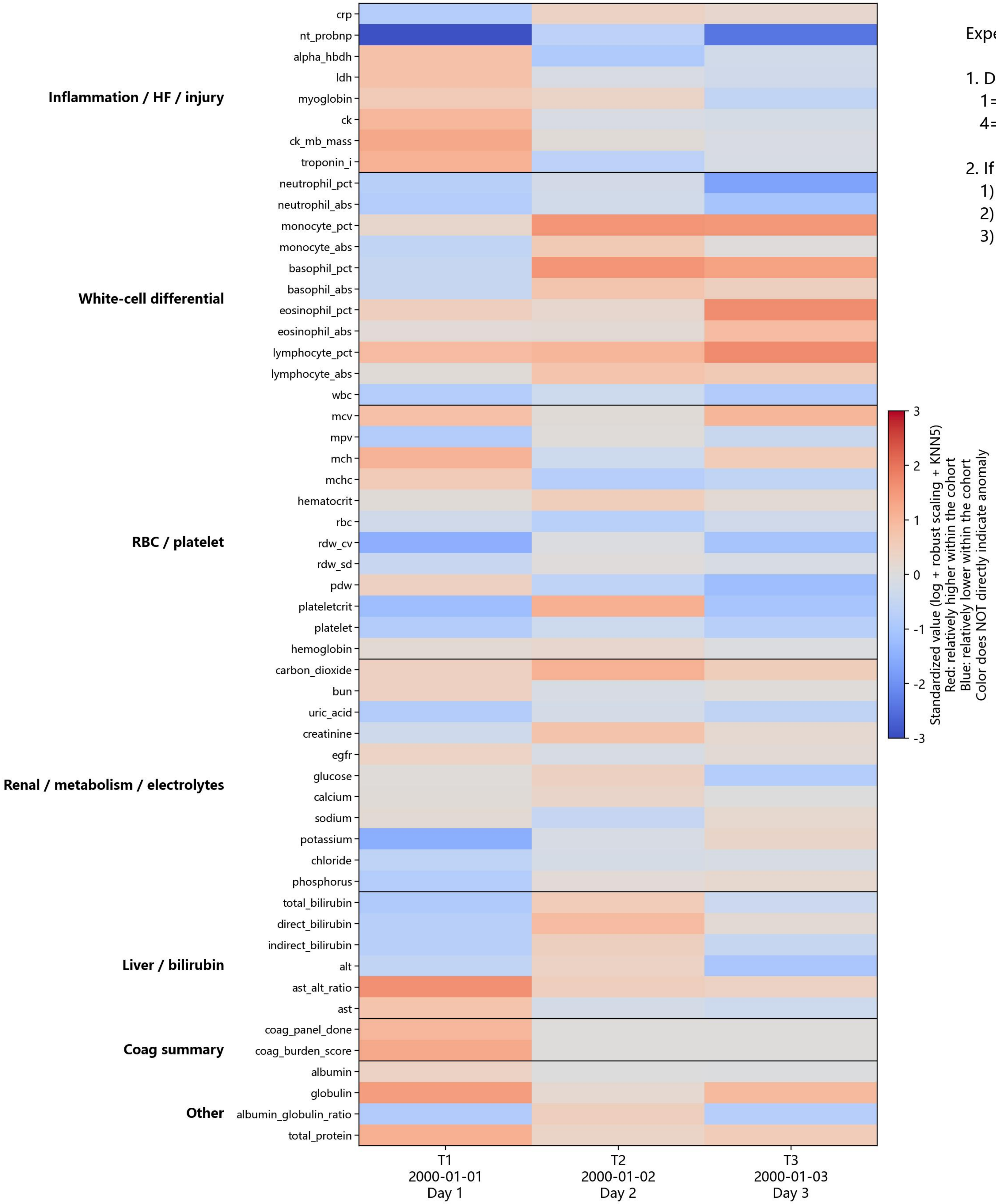

Expert review (blinded; no model score shown)

1. Degree of anomaly for this 3-point window (1-5):
   1=very typical; 2=relatively typical; 3=gray zone;
   4=relatively abnormal; 5=very abnormal

2. If scored 4-5, list the 3 most abnormal / noteworthy variables:
   1) ____________________________
   2) ____________________________
   3) ____________________________

Patient-window heatmap card for blinded expert review
ID: P181   Window: W01

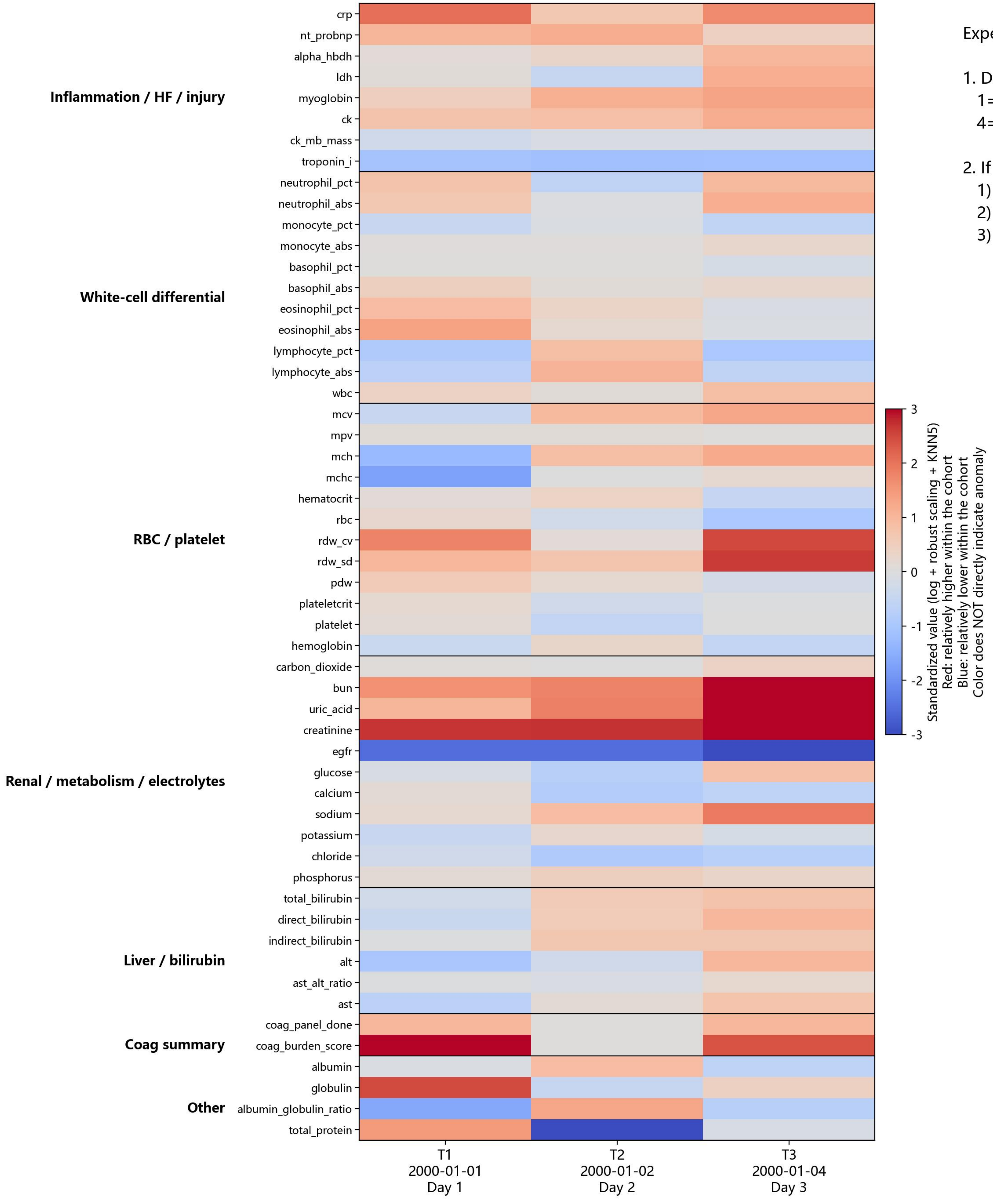

Expert review (blinded; no model score shown)

1. Degree of anomaly for this 3-point window (1-5):
   1=very typical; 2=relatively typical; 3=gray zone;
   4=relatively abnormal; 5=very abnormal

2. If scored 4-5, list the 3 most abnormal / noteworthy variables:
   1) ________________________
   2) ________________________
   3) ________________________

Patient-window heatmap card for blinded expert review
ID: P182   Window: W01

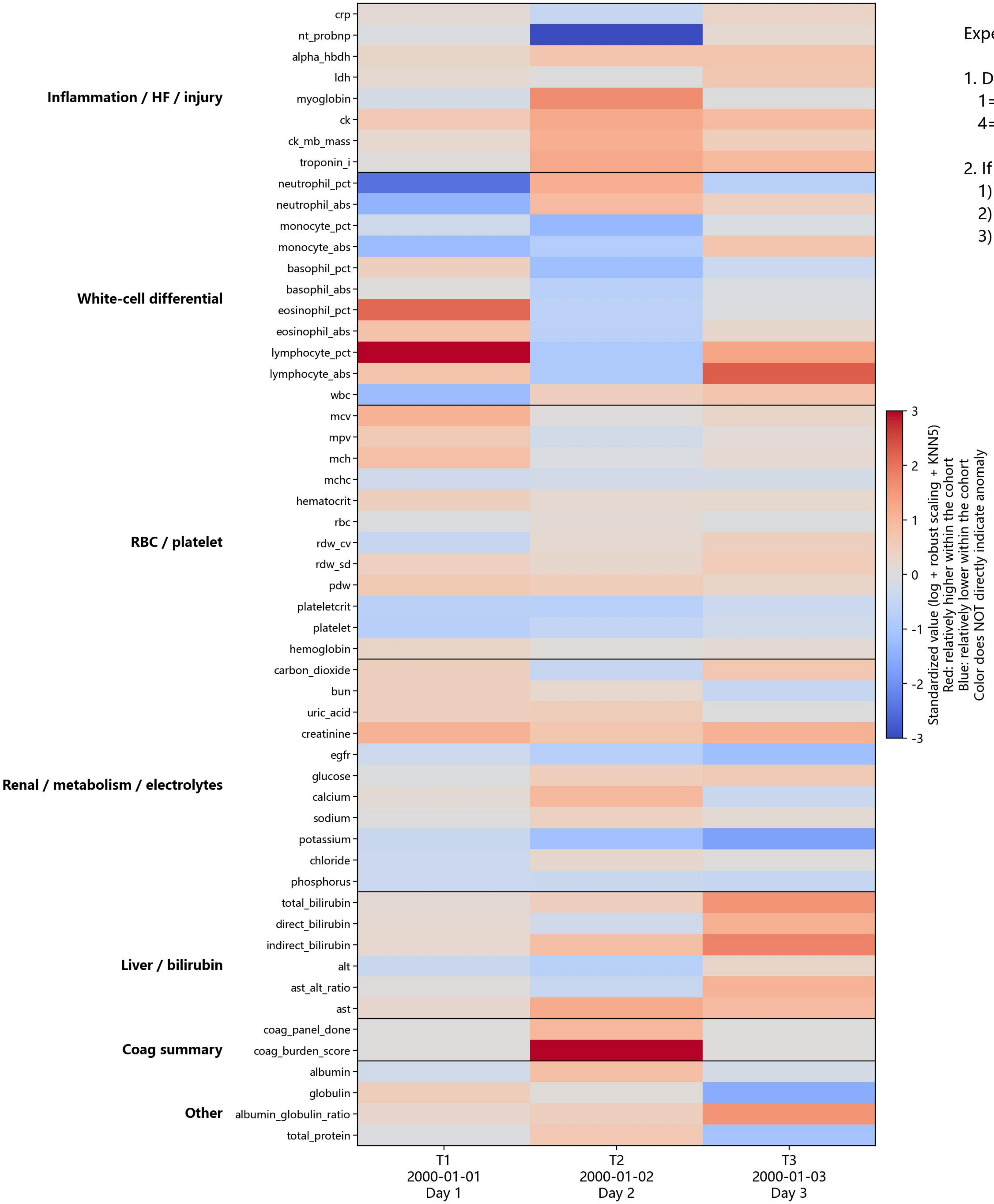

Expert review (blinded; no model score shown)

1. Degree of anomaly for this 3-point window (1-5):
   1=very typical; 2=relatively typical; 3=gray zone;
   4=relatively abnormal; 5=very abnormal

2. If scored 4-5, list the 3 most abnormal / noteworthy variables:
   1) ________________________
   2) ________________________
   3) ________________________

Patient-window heatmap card for blinded expert review
ID: P183   Window: W01

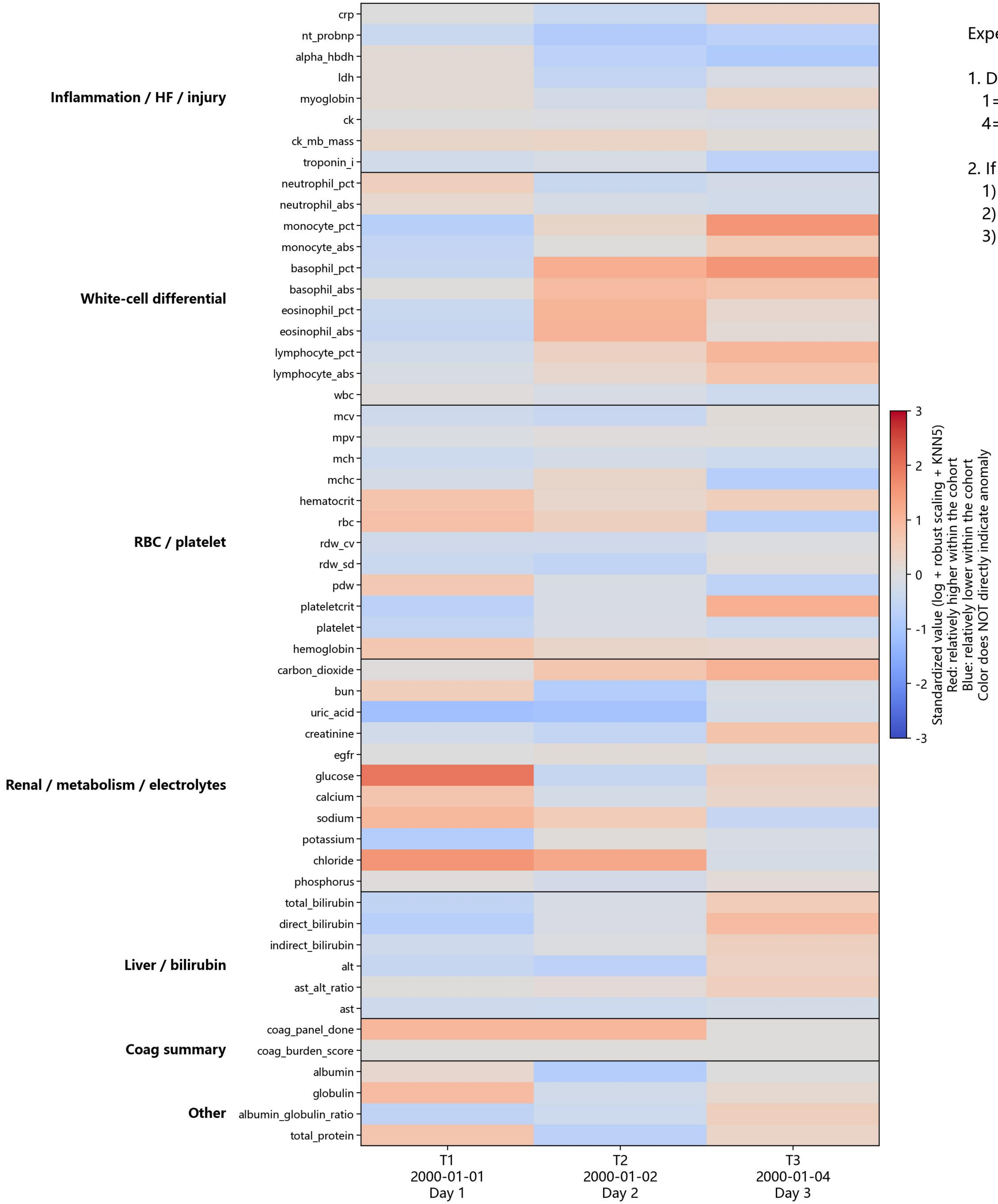

Expert review (blinded; no model score shown)

1. Degree of anomaly for this 3-point window (1-5):
   1=very typical; 2=relatively typical; 3=gray zone;
   4=relatively abnormal; 5=very abnormal

2. If scored 4-5, list the 3 most abnormal / noteworthy variables:
   1) ________________________
   2) ________________________
   3) ________________________
